# Supplementary material for: Publication speed in pharmacy practice journals: A comparative analysis
Source: PLoS One. 2021 Jun 29;16(6):e0253713. doi: 10.1371/journal.pone.0253713 (PMC8241115; doi:10.1371/journal.pone.0253713)
Supplement: S1 Appendix — (DOCX) [file pone.0253713.s001.docx]

**Publication speed in pharmacy practice journals: a comparative analysis**

**Supporting information 1. Comparison group: Results of the PMIDs random selection**

Antonio M. MENDES, Fernanda S. TONIN, Roberto PONTAROLO, Fernando FERNANDEZ-LLIMOS.

**Table 1. Research Randomizer Results: 1 Set of 3000 Yes Numbers Per Set**

| **Range: From 19209947 to 20237435 --** | **Range: From 20237436 to 21894257 --** | **Range: From 21894258 to 22631592 --** | **Range: From 22631593 to 23275957 --** | **Range: From 23275958 to 24380075 --** | **Range: From 24380076 to 26925884 --** | **Range: From 26925885 to 28163893 --** | **Range: From 28163894 to 29264908 --** | **Range: From 29264909 to 29289032 --** | **Range: From 29289033 to 30595005 --** |
| --- | --- | --- | --- | --- | --- | --- | --- | --- | --- |
| **2009** | **2010** | **2011** | **2012** | **2013** | **2014** | **2015** | **2016** | **2017** | **2018** |
| 19210225 | 20237803 | 21894472 | 22631880 | 23276474 | 24380923 | 26925902 | 28163955 | 29264912 | 29290787 |
| 19210531 | 20237886 | 21894542 | 22632234 | 23276596 | 24381736 | 26926468 | 28164509 | 29264920 | 29291655 |
| 19210980 | 20237900 | 21894740 | 22632247 | 23277245 | 24383224 | 26926610 | 28164666 | 29264921 | 29292022 |
| 19211078 | 20237905 | 21895092 | 22632313 | 23278305 | 24383424 | 26926929 | 28164722 | 29264924 | 29292138 |
| 19211503 | 20238146 | 21895208 | 22632447 | 23278650 | 24383613 | 26927501 | 28164794 | 29264937 | 29293009 |
| 19212720 | 20238863 | 21895423 | 22632524 | 23278990 | 24383688 | 26927749 | 28164935 | 29264939 | 29293112 |
| 19212844 | 20240587 | 21895521 | 22632565 | 23279138 | 24384868 | 26928085 | 28165990 | 29264960 | 29293211 |
| 19212851 | 20240768 | 21895777 | 22632638 | 23279251 | 24384873 | 26928719 | 28166444 | 29264974 | 29293296 |
| 19213090 | 20242041 | 21896139 | 22632715 | 23279795 | 24385411 | 26929381 | 28166741 | 29264986 | 29293355 |
| 19213192 | 20242615 | 21896245 | 22632888 | 23280372 | 24386458 | 26929856 | 28167006 | 29264996 | 29293556 |
| 19213336 | 20242882 | 21896317 | 22633374 | 23280494 | 24387830 | 26929902 | 28167391 | 29264997 | 29294046 |
| 19213617 | 20243569 | 21896328 | 22633950 | 23280971 | 24389342 | 26930250 | 28167707 | 29264998 | 29294105 |
| 19213735 | 20243746 | 21896599 | 22634718 | 23281167 | 24389895 | 26930462 | 28167766 | 29265007 | 29294410 |
| 19213789 | 20245293 | 21896942 | 22635119 | 23282370 | 24390500 | 26930733 | 28168052 | 29265010 | 29294633 |
| 19214952 | 20247229 | 21897243 | 22635221 | 23282509 | 24391128 | 26930902 | 28168390 | 29265024 | 29294785 |
| 19214971 | 20247468 | 21898318 | 22635285 | 23283578 | 24391484 | 26931027 | 28168601 | 29265029 | 29295014 |
| 19215507 | 20247545 | 21898435 | 22635326 | 23283593 | 24392724 | 26931131 | 28169870 | 29265031 | 29295512 |
| 19215516 | 20247564 | 21898482 | 22635670 | 23283865 | 24393388 | 26932005 | 28170781 | 29265044 | 29297467 |
| 19215931 | 20248252 | 21898488 | 22635810 | 23284224 | 24394423 | 26932151 | 28170870 | 29265047 | 29297548 |
| 19216184 | 20248949 | 21898769 | 22636101 | 23285352 | 24395474 | 26932252 | 28171925 | 29265053 | 29298180 |
| 19216217 | 20249298 | 21898946 | 22636176 | 23285883 | 24395690 | 26932425 | 28172056 | 29265064 | 29298234 |
| 19216219 | 20249351 | 21899465 | 22636291 | 23287120 | 24396386 | 26934480 | 28172534 | 29265070 | 29298465 |
| 19216702 | 20249783 | 21899472 | 22636295 | 23287191 | 24397060 | 26935168 | 28172669 | 29265072 | 29298512 |
| 19216754 | 20250207 | 21899505 | 22636368 | 23287602 | 24397715 | 26935196 | 28173287 | 29265073 | 29298719 |
| 19218327 | 20250566 | 21900084 | 22636574 | 23287746 | 24398199 | 26935487 | 28173294 | 29265096 | 29298727 |
| 19218911 | 20251180 | 21901469 | 22636907 | 23288061 | 24398277 | 26935923 | 28173412 | 29265109 | 29299032 |
| 19219107 | 20251391 | 21902394 | 22637138 | 23288314 | 24398712 | 26936337 | 28174403 | 29265117 | 29299039 |
| 19219891 | 20251687 | 21902765 | 22637586 | 23288499 | 24399107 | 26936687 | 28174557 | 29265119 | 29299641 |
| 19220020 | 20252825 | 21903058 | 22637698 | 23289990 | 24399517 | 26936743 | 28174637 | 29265130 | 29301044 |
| 19220149 | 20252844 | 21903067 | 22637775 | 23290485 | 24400083 | 26937093 | 28176084 | 29265139 | 29301091 |
| 19220231 | 20253209 | 21903207 | 22637964 | 23290858 | 24400817 | 26937530 | 28176891 | 29265140 | 29301646 |
| 19220390 | 20253337 | 21903269 | 22638075 | 23291218 | 24402912 | 26937793 | 28176954 | 29265144 | 29302050 |
| 19220877 | 20253634 | 21903337 | 22638310 | 23291612 | 24403650 | 26938166 | 28177313 | 29265147 | 29302569 |
| 19220893 | 20254152 | 21903427 | 22638353 | 23291988 | 24404319 | 26938518 | 28177739 | 29265155 | 29302943 |
| 19220946 | 20255052 | 21903526 | 22638391 | 23292480 | 24404463 | 26938580 | 28178009 | 29265156 | 29303301 |
| 19221198 | 20255571 | 21904459 | 22638425 | 23292706 | 24404539 | 26939203 | 28178059 | 29265164 | 29303910 |
| 19222505 | 20256948 | 21904531 | 22638543 | 23294168 | 24405484 | 26939312 | 28178875 | 29265168 | 29303955 |
| 19223001 | 20258682 | 21904797 | 22638578 | 23294199 | 24406592 | 26939621 | 28178901 | 29265169 | 29304190 |
| 19223232 | 20258782 | 21905005 | 22638819 | 23294432 | 24407310 | 26939748 | 28179022 | 29265170 | 29305655 |
| 19223238 | 20259333 | 21905026 | 22638927 | 23294643 | 24408249 | 26940062 | 28179348 | 29265179 | 29306249 |
| 19223298 | 20260007 | 21905254 | 22639111 | 23294970 | 24408472 | 26940363 | 28180039 | 29265187 | 29306446 |
| 19224479 | 20260510 | 21905617 | 22639130 | 23294980 | 24409255 | 26940783 | 28180388 | 29265203 | 29306540 |
| 19224729 | 20260564 | 21905866 | 22639625 | 23297930 | 24410654 | 26941169 | 28180567 | 29265209 | 29306943 |
| 19226355 | 20260609 | 21906155 | 22639925 | 23298726 | 24411444 | 26942018 | 28181190 | 29265218 | 29307197 |
| 19226439 | 20260811 | 21906203 | 22640437 | 23298759 | 24411951 | 26942900 | 28181361 | 29265241 | 29307229 |
| 19227012 | 20260961 | 21906458 | 22640697 | 23299609 | 24412997 | 26944906 | 28181417 | 29265243 | 29307262 |
| 19227219 | 20260988 | 21906711 | 22640781 | 23299613 | 24414203 | 26945266 | 28182417 | 29265245 | 29308084 |
| 19227934 | 20261423 | 21907072 | 22640813 | 23299662 | 24416062 | 26945900 | 28182633 | 29265246 | 29308918 |
| 19227935 | 20261872 | 21907128 | 22640846 | 23299949 | 24416810 | 26946941 | 28182958 | 29265247 | 29309187 |
| 19228718 | 20262833 | 21907306 | 22641321 | 23300331 | 24417040 | 26948017 | 28183096 | 29265292 | 29309439 |
| 19229116 | 20263055 | 21907532 | 22641613 | 23301081 | 24417483 | 26948030 | 28183204 | 29265294 | 29310781 |
| 19229455 | 20264052 | 21908210 | 22642095 | 23301771 | 24418351 | 26949040 | 28183552 | 29265303 | 29311018 |
| 19230059 | 20264450 | 21908733 | 22642489 | 23301841 | 24418531 | 26949046 | 28184132 | 29265306 | 29311448 |
| 19230112 | 20264607 | 21908813 | 22642542 | 23302030 | 24421192 | 26949426 | 28185760 | 29265322 | 29311704 |
| 19230262 | 20264851 | 21908864 | 22642585 | 23302138 | 24421776 | 26949981 | 28185982 | 29265323 | 29312177 |
| 19230443 | 20264996 | 21908872 | 22642677 | 23302324 | 24421977 | 26950010 | 28187033 | 29265330 | 29313314 |
| 19230941 | 20265166 | 21909051 | 22643220 | 23303695 | 24422304 | 26950040 | 28187146 | 29265331 | 29313452 |
| 19230985 | 20265527 | 21909501 | 22643472 | 23303727 | 24422968 | 26950047 | 28187442 | 29265342 | 29313520 |
| 19231109 | 20266226 | 21910314 | 22643505 | 23304175 | 24423449 | 26950096 | 28187724 | 29265345 | 29314555 |
| 19231115 | 20266356 | 21910447 | 22643574 | 23304182 | 24425444 | 26950735 | 28187926 | 29265352 | 29314618 |
| 19231367 | 20266662 | 21910595 | 22643610 | 23304258 | 24425511 | 26951042 | 28187973 | 29265365 | 29314707 |
| 19231385 | 20267263 | 21910603 | 22643977 | 23304522 | 24426473 | 26951357 | 28188174 | 29265366 | 29314758 |
| 19231641 | 20269323 | 21910818 | 22644377 | 23304561 | 24428812 | 26951824 | 28188329 | 29265369 | 29316232 |
| 19231755 | 20269577 | 21910932 | 22644409 | 23305043 | 24431376 | 26952190 | 28188419 | 29265374 | 29317004 |
| 19231868 | 20270554 | 21911352 | 22644540 | 23305620 | 24432924 | 26952272 | 28188666 | 29265381 | 29317788 |
| 19231945 | 20270772 | 21911627 | 22644749 | 23306585 | 24433476 | 26952527 | 28188776 | 29265387 | 29318006 |
| 19231950 | 20271340 | 21911818 | 22644800 | 23306788 | 24434152 | 26952645 | 28189328 | 29265418 | 29318021 |
| 19232086 | 20272310 | 21911879 | 22644925 | 23306888 | 24434322 | 26952992 | 28190205 | 29265445 | 29318483 |
| 19232429 | 20273066 | 21911928 | 22645217 | 23307139 | 24435469 | 26954725 | 28190411 | 29265455 | 29318789 |
| 19232565 | 20273383 | 21912606 | 22645295 | 23307260 | 24437211 | 26954726 | 28190824 | 29265459 | 29319007 |
| 19232771 | 20273722 | 21912628 | 22645323 | 23307466 | 24437830 | 26954755 | 28191220 | 29265467 | 29319140 |
| 19233626 | 20273832 | 21913161 | 22645653 | 23307795 | 24439067 | 26954944 | 28191407 | 29265468 | 29319426 |
| 19233841 | 20274570 | 21913372 | 22646233 | 23307875 | 24440076 | 26955257 | 28191417 | 29265473 | 29319436 |
| 19234128 | 20274759 | 21913624 | 22646767 | 23308301 | 24440468 | 26955672 | 28191927 | 29265480 | 29319869 |
| 19234230 | 20275214 | 21913746 | 22646980 | 23308505 | 24442149 | 26955838 | 28191972 | 29265484 | 29320008 |
| 19234688 | 20275579 | 21914042 | 22647044 | 23308544 | 24442348 | 26956542 | 28192942 | 29265487 | 29320121 |
| 19234700 | 20275877 | 21914185 | 22647093 | 23308617 | 24443378 | 26956875 | 28193133 | 29265489 | 29320328 |
| 19234843 | 20275987 | 21914249 | 22647294 | 23308672 | 24444090 | 26957004 | 28193283 | 29265510 | 29322161 |
| 19234861 | 20276134 | 21914907 | 22647383 | 23308733 | 24445254 | 26957089 | 28193725 | 29265513 | 29322335 |
| 19235560 | 20276174 | 21914941 | 22647502 | 23308754 | 24446020 | 26957522 | 28193754 | 29265533 | 29322884 |
| 19235839 | 20276670 | 21915665 | 22647530 | 23309229 | 24447542 | 26958973 | 28194258 | 29265544 | 29322989 |
| 19235849 | 20277242 | 21915723 | 22647886 | 23309876 | 24448363 | 26959422 | 28194483 | 29265547 | 29323139 |
| 19236382 | 20277637 | 21915930 | 22647905 | 23309912 | 24449663 | 26959663 | 28195253 | 29265552 | 29325303 |
| 19236455 | 20279234 | 21916058 | 22648066 | 23310077 | 24450401 | 26959765 | 28195458 | 29265553 | 29325317 |
| 19236680 | 20280120 | 21916091 | 22648403 | 23310162 | 24450801 | 26960131 | 28195754 | 29265569 | 29325408 |
| 19237662 | 20280886 | 21917089 | 22648562 | 23310336 | 24451318 | 26960233 | 28195805 | 29265570 | 29325828 |
| 19237995 | 20281925 | 21917103 | 22648667 | 23310940 | 24451561 | 26960266 | 28195998 | 29265571 | 29326914 |
| 19238045 | 20283898 | 21917383 | 22648806 | 23311539 | 24451776 | 26960406 | 28196162 | 29265573 | 29326967 |
| 19238352 | 20284127 | 21917441 | 22648816 | 23311740 | 24452125 | 26960560 | 28196637 | 29265575 | 29327153 |
| 19238645 | 20284728 | 21917490 | 22649226 | 23311879 | 24454213 | 26960618 | 28197219 | 29265584 | 29328449 |
| 19238896 | 20284993 | 21917743 | 22649527 | 23311937 | 24456194 | 26961485 | 28197287 | 29265593 | 29328532 |
| 19238920 | 20285269 | 21917876 | 22649879 | 23312150 | 24456445 | 26961869 | 28198968 | 29265597 | 29328981 |
| 19239021 | 20285788 | 21918139 | 22650118 | 23312806 | 24456979 | 26962701 | 28199594 | 29265609 | 29329901 |
| 19239092 | 20286424 | 21918241 | 22650198 | 23312853 | 24457063 | 26962883 | 28199868 | 29265611 | 29330176 |
| 19239213 | 20287391 | 21918365 | 22650809 | 23313732 | 24459183 | 26964198 | 28200037 | 29265637 | 29330332 |
| 19239682 | 20287682 | 21918392 | 22650850 | 23314455 | 24460453 | 26965279 | 28200172 | 29265640 | 29330770 |
| 19239822 | 20287747 | 21918579 | 22650972 | 23315259 | 24461636 | 26965600 | 28200454 | 29265643 | 29330897 |
| 19240241 | 20287861 | 21918587 | 22651158 | 23315446 | 24462381 | 26965937 | 28200609 | 29265648 | 29330995 |
| 19240433 | 20289781 | 21918784 | 22651181 | 23315459 | 24463416 | 26966428 | 28200912 | 29265649 | 29331143 |
| 19240836 | 20290084 | 21919157 | 22651891 | 23315730 | 24463633 | 26966466 | 28201025 | 29265651 | 29332256 |
| 19241344 | 20291323 | 21919395 | 22651907 | 23315822 | 24463859 | 26966502 | 28201134 | 29265669 | 29332620 |
| 19241434 | 20291679 | 21919904 | 22652679 | 23315908 | 24464307 | 26966617 | 28201944 | 29265671 | 29333076 |
| 19241784 | 20291698 | 21920019 | 22652938 | 23316439 | 24464414 | 26966802 | 28202204 | 29265694 | 29333324 |
| 19241809 | 20292159 | 21920132 | 22652972 | 23318319 | 24465478 | 26967009 | 28202497 | 29265705 | 29333770 |
| 19241816 | 20292427 | 21920377 | 22653052 | 23318642 | 24469761 | 26967271 | 28202639 | 29265714 | 29333984 |
| 19242285 | 20292974 | 21921219 | 22653062 | 23319601 | 24471400 | 26967294 | 28202764 | 29265722 | 29334740 |
| 19242390 | 20293008 | 21921301 | 22653408 | 23319931 | 24472729 | 26967407 | 28202877 | 29265736 | 29335370 |
| 19242700 | 20294074 | 21922413 | 22653478 | 23320640 | 24477192 | 26967540 | 28203947 | 29265738 | 29335502 |
| 19242895 | 20294618 | 21922739 | 22654074 | 23320836 | 24478538 | 26968790 | 28204366 | 29265742 | 29335528 |
| 19243061 | 20294977 | 21923453 | 22654146 | 23321908 | 24478893 | 26969188 | 28204420 | 29265745 | 29336174 |
| 19243212 | 20295917 | 21923724 | 22654221 | 23322738 | 24480018 | 26969836 | 28204527 | 29265752 | 29336186 |
| 19243554 | 20296256 | 21923779 | 22654627 | 23323182 | 24480062 | 26970179 | 28204552 | 29265772 | 29336259 |
| 19243978 | 20297128 | 21924035 | 22655144 | 23323186 | 24480160 | 26970711 | 28204880 | 29265776 | 29336686 |
| 19244342 | 20297498 | 21924748 | 22655340 | 23324234 | 24481864 | 26971361 | 28205065 | 29265783 | 29337052 |
| 19244397 | 20298471 | 21925933 | 22655800 | 23324292 | 24481970 | 26971682 | 28206080 | 29265785 | 29337844 |
| 19244722 | 20299500 | 21926062 | 22655859 | 23324732 | 24482183 | 26971713 | 28206349 | 29265801 | 29338552 |
| 19244883 | 20300367 | 21926265 | 22655935 | 23324861 | 24483197 | 26971719 | 28206355 | 29265805 | 29338650 |
| 19245106 | 20300608 | 21927141 | 22656080 | 23326039 | 24483339 | 26971844 | 28206522 | 29265810 | 29338756 |
| 19245410 | 20301632 | 21927444 | 22656364 | 23326064 | 24483648 | 26972299 | 28207145 | 29265812 | 29339024 |
| 19245432 | 20301741 | 21927750 | 22657280 | 23326200 | 24483810 | 26972338 | 28207275 | 29265814 | 29339040 |
| 19246281 | 20302352 | 21928166 | 22657313 | 23326230 | 24484448 | 26972988 | 28207282 | 29265815 | 29339153 |
| 19246505 | 20302600 | 21928280 | 22657345 | 23327062 | 24485194 | 26973544 | 28207411 | 29265818 | 29339511 |
| 19246578 | 20302921 | 21928497 | 22657545 | 23327356 | 24486273 | 26974516 | 28207566 | 29265819 | 29341275 |
| 19246610 | 20303386 | 21928721 | 22658018 | 23328814 | 24487016 | 26975202 | 28207888 | 29265820 | 29341869 |
| 19247102 | 20303845 | 21928816 | 22658027 | 23329291 | 24487302 | 26975939 | 28208133 | 29265827 | 29342600 |
| 19247160 | 20304788 | 21929222 | 22658307 | 23330946 | 24487908 | 26977244 | 28208633 | 29265830 | 29342791 |
| 19247349 | 20304841 | 21929316 | 22658752 | 23331424 | 24487982 | 26977265 | 28209392 | 29265836 | 29342975 |
| 19248938 | 20304870 | 21929554 | 22658845 | 23331942 | 24488911 | 26977867 | 28209552 | 29265837 | 29343008 |
| 19249038 | 20304891 | 21929578 | 22659042 | 23331980 | 24493259 | 26978764 | 28209566 | 29265856 | 29343339 |
| 19249143 | 20305049 | 21929625 | 22659323 | 23333738 | 24493456 | 26978940 | 28211352 | 29265857 | 29343507 |
| 19250184 | 20305266 | 21929662 | 22659356 | 23334162 | 24494064 | 26979451 | 28212534 | 29265862 | 29343514 |
| 19250290 | 20305381 | 21930079 | 22659391 | 23334871 | 24494219 | 26979593 | 28213298 | 29265864 | 29343758 |
| 19250515 | 20305839 | 21930081 | 22659496 | 23334946 | 24494470 | 26979666 | 28213927 | 29265868 | 29343965 |
| 19250616 | 20306573 | 21930821 | 22659524 | 23336101 | 24496672 | 26980373 | 28213969 | 29265871 | 29344067 |
| 19252395 | 20306670 | 21930963 | 22659539 | 23336211 | 24496951 | 26980672 | 28213973 | 29265876 | 29344298 |
| 19252406 | 20310305 | 21931006 | 22659541 | 23336490 | 24497010 | 26981676 | 28214749 | 29265896 | 29345326 |
| 19252647 | 20311006 | 21931280 | 22659569 | 23336501 | 24499041 | 26981717 | 28216303 | 29265905 | 29345533 |
| 19252649 | 20311093 | 21931305 | 22659691 | 23336743 | 24499951 | 26982189 | 28216586 | 29265916 | 29346199 |
| 19252836 | 20311158 | 21931376 | 22659798 | 23336800 | 24500718 | 26982229 | 28217311 | 29265921 | 29346438 |
| 19252889 | 20311857 | 21931381 | 22660116 | 23337430 | 24502361 | 26982858 | 28217507 | 29265930 | 29347032 |
| 19253400 | 20313247 | 21931625 | 22660531 | 23338196 | 24502687 | 26983160 | 28217588 | 29265936 | 29347249 |
| 19254884 | 20314168 | 21931635 | 22661224 | 23338264 | 24502853 | 26984554 | 28217614 | 29265940 | 29347296 |
| 19255445 | 20314720 | 21931788 | 22661349 | 23338359 | 24502926 | 26984830 | 28218054 | 29265942 | 29347713 |
| 19255508 | 20314730 | 21932039 | 22661545 | 23339214 | 24505827 | 26985254 | 28218554 | 29265944 | 29348008 |
| 19255599 | 20314994 | 21932258 | 22661653 | 23339245 | 24506657 | 26986525 | 28219168 | 29265958 | 29348067 |
| 19255677 | 20315023 | 21932857 | 22662197 | 23339551 | 24507558 | 26986921 | 28219810 | 29265970 | 29348395 |
| 19255789 | 20315306 | 21932885 | 22662420 | 23340086 | 24510202 | 26986946 | 28219963 | 29265973 | 29349431 |
| 19256076 | 20316924 | 21932988 | 22662794 | 23340698 | 24510448 | 26987180 | 28220446 | 29265991 | 29349576 |
| 19256215 | 20318345 | 21933060 | 22662817 | 23340979 | 24510742 | 26987249 | 28220761 | 29265995 | 29349876 |
| 19256438 | 20319691 | 21933815 | 22663453 | 23341263 | 24510937 | 26987268 | 28220805 | 29265997 | 29349891 |
| 19256471 | 20319791 | 21934073 | 22663480 | 23341888 | 24512262 | 26987315 | 28221329 | 29265998 | 29350028 |
| 19256534 | 20319924 | 21934812 | 22663481 | 23341971 | 24512462 | 26987634 | 28221454 | 29266004 | 29350530 |
| 19256555 | 20320406 | 21934997 | 22663547 | 23343330 | 24512472 | 26987799 | 28222417 | 29266012 | 29350704 |
| 19256823 | 20320870 | 21935427 | 22663788 | 23343354 | 24512935 | 26987856 | 28223089 | 29266016 | 29351393 |
| 19256951 | 20321927 | 21935466 | 22663799 | 23344093 | 24514448 | 26987933 | 28223876 | 29266020 | 29351750 |
| 19257094 | 20323565 | 21936206 | 22663857 | 23344219 | 24514796 | 26989364 | 28223899 | 29266024 | 29352230 |
| 19257233 | 20323588 | 21936661 | 22664161 | 23345112 | 24515069 | 26990209 | 28224556 | 29266045 | 29352330 |
| 19257532 | 20323686 | 21936764 | 22664301 | 23345199 | 24515346 | 26990412 | 28224889 | 29266052 | 29352759 |
| 19257613 | 20324003 | 21937456 | 22664378 | 23345233 | 24515566 | 26990422 | 28225192 | 29266053 | 29352791 |
| 19258314 | 20324483 | 21938107 | 22664429 | 23345419 | 24516191 | 26990668 | 28225241 | 29266055 | 29353511 |
| 19258792 | 20324595 | 21938176 | 22664611 | 23346665 | 24516398 | 26990825 | 28225354 | 29266066 | 29353694 |
| 19258926 | 20326502 | 21938192 | 22664820 | 23346911 | 24518548 | 26990917 | 28225505 | 29266074 | 29355130 |
| 19259208 | 20326583 | 21938281 | 22664914 | 23347404 | 24518708 | 26991857 | 28225510 | 29266082 | 29356475 |
| 19259371 | 20327491 | 21938640 | 22665032 | 23347473 | 24518841 | 26992363 | 28225869 | 29266088 | 29357907 |
| 19259566 | 20327686 | 21939198 | 22665115 | 23347779 | 24519711 | 26992627 | 28225877 | 29266091 | 29358460 |
| 19259614 | 20328751 | 21939266 | 22665224 | 23348000 | 24519847 | 26993322 | 28226441 | 29266092 | 29359408 |
| 19259771 | 20328776 | 21939539 | 22665384 | 23348117 | 24520082 | 26993690 | 28226818 | 29266096 | 29359441 |
| 19259801 | 20331000 | 21939644 | 22665495 | 23348604 | 24521122 | 26994060 | 28226937 | 29266097 | 29360015 |
| 19260304 | 20331460 | 21939703 | 22665563 | 23348927 | 24522869 | 26994188 | 28226948 | 29266110 | 29360116 |
| 19260650 | 20331657 | 21939730 | 22665934 | 23348968 | 24523077 | 26994318 | 28227314 | 29266114 | 29360238 |
| 19261584 | 20332534 | 21939765 | 22666322 | 23349145 | 24523727 | 26994329 | 28228598 | 29266122 | 29360283 |
| 19262108 | 20333694 | 21939809 | 22666707 | 23349563 | 24524354 | 26995562 | 28229237 | 29266126 | 29360918 |
| 19262134 | 20334042 | 21939848 | 22666974 | 23349748 | 24525116 | 26995649 | 28229655 | 29266147 | 29361665 |
| 19262163 | 20334674 | 21939920 | 22667655 | 23349821 | 24525145 | 26995695 | 28229693 | 29266151 | 29361721 |
| 19262397 | 20335056 | 21939999 | 22668092 | 23350717 | 24525803 | 26996633 | 28230043 | 29266166 | 29362117 |
| 19262559 | 20335187 | 21940356 | 22668373 | 23351441 | 24525883 | 26998171 | 28230200 | 29266167 | 29362467 |
| 19263495 | 20335579 | 21940357 | 22668398 | 23351700 | 24526886 | 26999332 | 28230333 | 29266179 | 29363031 |
| 19263637 | 20335781 | 21940558 | 22668428 | 23351971 | 24528324 | 26999593 | 28230443 | 29266196 | 29363436 |
| 19263984 | 20336218 | 21940600 | 22668771 | 23351984 | 24528444 | 27000619 | 28230626 | 29266229 | 29363528 |
| 19264250 | 20337249 | 21941186 | 22669362 | 23352115 | 24529305 | 27000812 | 28231064 | 29266240 | 29364026 |
| 19264943 | 20337331 | 21941592 | 22669594 | 23352378 | 24529759 | 27001235 | 28233889 | 29266262 | 29364486 |
| 19265317 | 20337655 | 21941730 | 22669778 | 23352889 | 24531144 | 27001302 | 28234711 | 29266268 | 29364993 |
| 19265349 | 20339394 | 21941962 | 22669905 | 23353151 | 24531439 | 27001346 | 28234998 | 29266271 | 29365626 |
| 19265538 | 20340992 | 21941971 | 22670409 | 23353284 | 24533265 | 27003005 | 28235147 | 29266275 | 29366410 |
| 19265579 | 20341009 | 21942539 | 22670521 | 23355124 | 24533855 | 27003023 | 28235789 | 29266277 | 29366433 |
| 19265809 | 20341046 | 21942673 | 22670623 | 23355379 | 24538387 | 27003280 | 28235991 | 29266280 | 29366514 |
| 19267713 | 20341666 | 21942871 | 22670643 | 23355406 | 24540516 | 27003283 | 28236533 | 29266281 | 29366602 |
| 19267991 | 20345239 | 21943028 | 22670923 | 23355436 | 24543673 | 27003706 | 28236627 | 29266282 | 29366622 |
| 19268155 | 20345334 | 21943558 | 22671244 | 23355771 | 24544591 | 27003839 | 28236928 | 29266288 | 29366921 |
| 19268844 | 20345341 | 21943638 | 22671446 | 23355822 | 24545301 | 27004617 | 28237792 | 29266299 | 29367361 |
| 19268978 | 20345548 | 21944142 | 22671620 | 23356239 | 24546386 | 27005004 | 28237900 | 29266300 | 29368155 |
| 19270550 | 20345957 | 21944861 | 22671647 | 23356442 | 24546842 | 27005849 | 28237943 | 29266305 | 29368257 |
| 19270911 | 20346267 | 21945328 | 22671759 | 23356796 | 24547953 | 27006108 | 28238571 | 29266310 | 29368965 |
| 19271338 | 20347777 | 21945876 | 22671888 | 23357566 | 24548730 | 27006636 | 28238699 | 29266319 | 29369221 |
| 19271435 | 20348562 | 21946188 | 22671971 | 23357923 | 24549651 | 27007162 | 28238865 | 29266331 | 29369911 |
| 19271444 | 20348823 | 21946991 | 22672357 | 23358272 | 24549670 | 27007188 | 28239017 | 29266334 | 29370051 |
| 19271957 | 20350137 | 21947073 | 22672447 | 23358769 | 24549833 | 27007285 | 28239576 | 29266353 | 29370149 |
| 19272680 | 20352009 | 21947197 | 22672490 | 23358782 | 24550958 | 27007613 | 28239849 | 29266359 | 29370364 |
| 19273183 | 20352370 | 21947204 | 22673095 | 23359012 | 24551927 | 27009103 | 28240023 | 29266362 | 29370827 |
| 19273619 | 20352460 | 21947313 | 22673099 | 23359332 | 24552755 | 27009259 | 28240454 | 29266365 | 29371566 |
| 19275347 | 20353148 | 21947449 | 22673122 | 23359627 | 24552866 | 27009577 | 28240526 | 29266377 | 29371608 |
| 19276394 | 20353471 | 21947676 | 22673694 | 23359632 | 24555248 | 27009872 | 28241505 | 29266382 | 29372017 |
| 19276721 | 20354183 | 21947684 | 22674242 | 23360049 | 24555326 | 27009946 | 28241518 | 29266398 | 29372724 |
| 19276838 | 20354211 | 21947796 | 22674998 | 23360594 | 24555388 | 27010113 | 28241538 | 29266429 | 29372892 |
| 19277124 | 20354466 | 21948084 | 22675184 | 23360602 | 24556516 | 27011656 | 28242367 | 29266431 | 29373056 |
| 19277777 | 20355168 | 21948404 | 22675209 | 23361455 | 24558408 | 27012397 | 28243148 | 29266436 | 29373144 |
| 19277825 | 20355304 | 21948576 | 22675718 | 23362025 | 24558789 | 27012902 | 28243344 | 29266443 | 29373220 |
| 19278567 | 20355511 | 21948777 | 22675748 | 23362048 | 24560843 | 27012930 | 28244245 | 29266449 | 29373274 |
| 19278619 | 20355902 | 21948844 | 22675842 | 23362114 | 24561556 | 27013991 | 28244445 | 29266472 | 29373405 |
| 19278758 | 20356206 | 21948956 | 22675988 | 23362891 | 24562077 | 27014007 | 28244950 | 29266475 | 29374530 |
| 19279130 | 20356746 | 21949704 | 22676003 | 23363260 | 24563708 | 27014653 | 28244958 | 29266485 | 29374580 |
| 19279226 | 20357095 | 21950364 | 22676049 | 23363287 | 24563883 | 27014715 | 28244960 | 29266493 | 29374937 |
| 19280740 | 20357669 | 21950372 | 22676252 | 23363342 | 24564673 | 27015420 | 28245241 | 29266494 | 29375622 |
| 19281566 | 20357970 | 21950476 | 22676497 | 23363396 | 24566664 | 27015623 | 28245527 | 29266496 | 29375868 |
| 19282592 | 20358199 | 21950765 | 22676772 | 23363676 | 24568997 | 27016228 | 28245534 | 29266502 | 29376068 |
| 19283925 | 20358429 | 21950954 | 22677094 | 23363747 | 24570247 | 27017058 | 28245607 | 29266528 | 29379477 |
| 19284020 | 20360365 | 21951018 | 22677179 | 23363755 | 24570937 | 27017092 | 28245855 | 29266535 | 29379981 |
| 19284348 | 20362594 | 21951527 | 22677529 | 23364608 | 24571699 | 27018708 | 28245923 | 29266540 | 29380655 |
| 19284437 | 20362600 | 21952092 | 22677959 | 23365217 | 24574211 | 27019347 | 28245974 | 29266543 | 29380691 |
| 19284623 | 20362713 | 21952229 | 22678011 | 23365295 | 24574475 | 27019453 | 28246086 | 29266548 | 29380818 |
| 19284738 | 20362817 | 21952459 | 22678086 | 23365722 | 24574502 | 27020253 | 28246419 | 29266568 | 29381138 |
| 19284979 | 20363082 | 21952716 | 22678232 | 23366723 | 24575985 | 27020955 | 28247349 | 29266575 | 29381681 |
| 19286160 | 20363545 | 21952818 | 22678277 | 23366729 | 24576285 | 27021069 | 28248652 | 29266577 | 29382077 |
| 19286821 | 20363956 | 21953232 | 22678376 | 23367086 | 24576646 | 27021651 | 28249761 | 29266578 | 29382205 |
| 19286867 | 20364227 | 21953831 | 22678387 | 23368220 | 24579971 | 27022376 | 28250041 | 29266580 | 29382326 |
| 19287343 | 20364655 | 21954098 | 22678409 | 23368532 | 24579992 | 27022935 | 28250478 | 29266593 | 29383446 |
| 19287367 | 20365849 | 21954714 | 22678419 | 23368594 | 24580379 | 27023313 | 28251373 | 29266599 | 29384364 |
| 19288010 | 20366809 | 21954879 | 22678476 | 23369613 | 24581270 | 27023480 | 28251773 | 29266612 | 29384713 |
| 19288319 | 20367243 | 21954902 | 22678687 | 23369818 | 24581428 | 27023645 | 28252120 | 29266617 | 29384853 |
| 19288428 | 20367354 | 21955097 | 22678739 | 23370108 | 24583159 | 27023676 | 28252198 | 29266626 | 29384898 |
| 19289160 | 20369234 | 21955200 | 22679384 | 23370822 | 24583365 | 27025059 | 28252391 | 29266635 | 29385289 |
| 19289570 | 20370194 | 21955242 | 22679466 | 23371177 | 24584332 | 27025860 | 28252544 | 29266642 | 29385481 |
| 19289836 | 20370337 | 21955275 | 22679488 | 23371327 | 24584610 | 27026486 | 28252745 | 29266670 | 29385564 |
| 19290180 | 20370864 | 21955324 | 22679668 | 23371390 | 24585544 | 27027466 | 28253074 | 29266683 | 29385632 |
| 19290945 | 20371304 | 21955353 | 22679879 | 23371945 | 24585744 | 27027777 | 28253604 | 29266707 | 29385663 |
| 19291145 | 20372902 | 21955737 | 22680845 | 23372043 | 24586386 | 27027791 | 28253788 | 29266712 | 29388233 |
| 19291486 | 20373246 | 21955949 | 22681100 | 23372298 | 24588513 | 27027802 | 28254243 | 29266715 | 29388425 |
| 19291501 | 20374582 | 21956056 | 22681223 | 23372305 | 24588760 | 27027914 | 28254435 | 29266720 | 29388823 |
| 19291865 | 20374889 | 21956159 | 22681345 | 23372560 | 24588970 | 27028370 | 28254495 | 29266723 | 29389233 |
| 19291902 | 20376303 | 21956347 | 22681363 | 23372907 | 24590775 | 27028644 | 28254657 | 29266739 | 29389409 |
| 19292226 | 20377660 | 21957056 | 22681705 | 23373317 | 24591242 | 27029007 | 28254827 | 29266740 | 29389710 |
| 19292683 | 20377924 | 21957795 | 22681849 | 23373616 | 24592583 | 27029048 | 28254905 | 29266742 | 29389844 |
| 19292714 | 20378040 | 21958140 | 22682147 | 23373861 | 24592624 | 27029616 | 28255267 | 29266746 | 29391710 |
| 19293081 | 20378134 | 21958305 | 22682250 | 23373959 | 24592743 | 27029836 | 28255360 | 29266760 | 29391816 |
| 19293491 | 20378490 | 21958577 | 22682513 | 23374338 | 24593186 | 27029943 | 28255407 | 29266763 | 29392032 |
| 19293595 | 20379450 | 21958706 | 22682638 | 23374432 | 24593244 | 27030011 | 28255616 | 29266767 | 29392210 |
| 19293712 | 20379935 | 21958949 | 22682751 | 23374542 | 24594419 | 27030914 | 28255877 | 29266774 | 29392465 |
| 19294674 | 20380058 | 21959255 | 22682835 | 23374932 | 24595484 | 27033685 | 28255942 | 29266781 | 29392496 |
| 19294824 | 20380957 | 21959329 | 22683129 | 23375663 | 24595638 | 27034075 | 28256425 | 29266783 | 29392703 |
| 19295008 | 20381568 | 21959357 | 22683405 | 23375674 | 24596618 | 27035102 | 28256709 | 29266789 | 29393443 |
| 19295432 | 20381949 | 21959610 | 22683741 | 23375904 | 24596787 | 27035289 | 28256890 | 29266794 | 29393682 |
| 19295546 | 20382787 | 21960140 | 22683813 | 23375985 | 24597006 | 27035379 | 28257333 | 29266797 | 29394163 |
| 19295732 | 20383870 | 21960541 | 22683945 | 23376926 | 24598832 | 27035424 | 28257643 | 29266803 | 29394563 |
| 19295742 | 20384078 | 21960853 | 22684008 | 23377676 | 24599584 | 27035987 | 28258285 | 29266812 | 29395203 |
| 19295763 | 20384968 | 21960920 | 22684023 | 23377856 | 24599607 | 27036251 | 28258384 | 29266813 | 29395451 |
| 19296183 | 20385295 | 21962569 | 22684154 | 23378488 | 24599761 | 27036407 | 28258898 | 29266816 | 29397295 |
| 19296260 | 20385326 | 21962633 | 22684637 | 23378624 | 24600774 | 27036527 | 28259038 | 29266840 | 29397945 |
| 19296813 | 20385513 | 21962696 | 22684642 | 23379681 | 24601046 | 27037481 | 28259200 | 29266851 | 29398485 |
| 19298290 | 20385705 | 21963152 | 22684830 | 23379814 | 24601244 | 27037710 | 28259568 | 29266863 | 29398685 |
| 19298526 | 20386662 | 21963201 | 22685227 | 23379832 | 24601444 | 27037807 | 28260026 | 29266871 | 29399648 |
| 19298597 | 20387040 | 21963395 | 22685424 | 23380505 | 24602068 | 27038997 | 28261234 | 29266874 | 29400628 |
| 19298738 | 20387113 | 21963937 | 22685673 | 23380996 | 24604149 | 27039307 | 28261476 | 29266875 | 29400901 |
| 19299671 | 20387414 | 21964057 | 22685892 | 23381383 | 24605202 | 27039464 | 28261796 | 29266880 | 29401155 |
| 19299938 | 20388661 | 21964094 | 22686477 | 23381769 | 24606407 | 27039796 | 28261931 | 29266882 | 29401391 |
| 19300027 | 20389914 | 21964157 | 22686512 | 23381911 | 24607286 | 27040231 | 28262218 | 29266883 | 29401561 |
| 19300193 | 20391338 | 21964363 | 22686553 | 23382939 | 24608420 | 27040235 | 28262297 | 29266884 | 29401668 |
| 19300765 | 20391638 | 21964488 | 22686591 | 23383147 | 24608495 | 27040464 | 28262744 | 29266885 | 29402138 |
| 19300876 | 20392143 | 21964859 | 22686678 | 23383297 | 24609111 | 27040526 | 28262949 | 29266888 | 29402532 |
| 19301176 | 20392730 | 21965282 | 22687203 | 23383758 | 24609257 | 27040768 | 28263318 | 29266901 | 29403141 |
| 19302407 | 20392759 | 21965567 | 22687270 | 23384054 | 24609379 | 27042008 | 28263359 | 29266915 | 29403450 |
| 19302494 | 20393189 | 21965839 | 22687325 | 23384356 | 24609628 | 27042018 | 28263423 | 29266929 | 29403934 |
| 19302956 | 20393540 | 21965904 | 22687624 | 23384575 | 24610823 | 27042194 | 28263465 | 29266937 | 29403984 |
| 19303101 | 20393794 | 21966204 | 22687648 | 23384716 | 24611042 | 27042875 | 28263489 | 29266941 | 29404080 |
| 19303531 | 20393940 | 21966506 | 22687845 | 23385039 | 24611447 | 27043423 | 28263659 | 29266966 | 29404141 |
| 19304378 | 20395511 | 21966834 | 22688108 | 23386417 | 24611964 | 27043479 | 28264442 | 29266984 | 29404438 |
| 19304383 | 20395566 | 21966916 | 22688691 | 23386517 | 24612675 | 27043849 | 28265401 | 29267001 | 29404491 |
| 19305544 | 20397035 | 21967039 | 22688825 | 23386948 | 24613779 | 27044500 | 28265696 | 29267015 | 29404909 |
| 19305644 | 20397571 | 21967412 | 22688856 | 23387203 | 24614022 | 27045555 | 28266446 | 29267021 | 29404929 |
| 19305743 | 20397662 | 21967954 | 22688872 | 23387492 | 24615323 | 27045674 | 28266458 | 29267026 | 29405579 |
| 19305912 | 20399462 | 21968192 | 22688949 | 23387750 | 24616568 | 27045839 | 28266564 | 29267048 | 29406023 |
| 19306168 | 20399886 | 21968268 | 22688991 | 23387814 | 24617055 | 27045999 | 28266570 | 29267072 | 29406140 |
| 19306190 | 20400434 | 21968380 | 22689007 | 23387890 | 24617149 | 27046136 | 28266978 | 29267080 | 29406488 |
| 19306360 | 20400599 | 21968834 | 22689273 | 23388925 | 24617458 | 27046936 | 28267650 | 29267091 | 29406526 |
| 19306826 | 20400612 | 21968961 | 22689840 | 23389381 | 24620422 | 27047210 | 28268173 | 29267126 | 29407627 |
| 19306893 | 20400649 | 21969179 | 22690075 | 23389446 | 24620703 | 27047597 | 28268285 | 29267144 | 29407954 |
| 19307126 | 20400917 | 21969243 | 22690184 | 23389820 | 24621027 | 27047675 | 28268504 | 29267147 | 29408657 |
| 19307430 | 20401300 | 21969599 | 22690542 | 23390124 | 24621384 | 27047924 | 28268536 | 29267151 | 29409272 |
| 19308167 | 20401789 | 21969700 | 22690585 | 23392148 | 24621488 | 27048168 | 28268630 | 29267153 | 29409411 |
| 19308216 | 20402109 | 21969837 | 22690651 | 23392332 | 24622550 | 27048649 | 28268953 | 29267154 | 29409957 |
| 19308224 | 20403491 | 21969916 | 22690710 | 23392496 | 24623722 | 27048920 | 28269649 | 29267160 | 29410241 |
| 19308383 | 20404693 | 21970018 | 22690901 | 23392650 | 24624653 | 27048998 | 28270907 | 29267165 | 29411655 |
| 19308845 | 20404763 | 21970582 | 22690910 | 23392762 | 24624696 | 27049575 | 28271386 | 29267168 | 29412181 |
| 19309023 | 20404920 | 21970679 | 22691480 | 23392832 | 24625038 | 27050306 | 28271841 | 29267185 | 29412348 |
| 19309461 | 20405983 | 21971092 | 22691504 | 23392948 | 24626154 | 27050642 | 28272052 | 29267196 | 29412567 |
| 19309779 | 20408067 | 21971181 | 22691658 | 23392997 | 24626290 | 27050712 | 28272109 | 29267198 | 29413117 |
| 19309803 | 20408876 | 21971324 | 22691828 | 23393398 | 24627042 | 27051033 | 28272447 | 29267226 | 29413587 |
| 19310248 | 20409136 | 21971450 | 22692042 | 23393464 | 24627960 | 27052224 | 28272475 | 29267235 | 29414119 |
| 19310967 | 20409277 | 21972211 | 22692552 | 23393572 | 24628779 | 27052878 | 28273182 | 29267236 | 29414174 |
| 19311249 | 20409607 | 21972988 | 22693111 | 23393645 | 24629203 | 27053061 | 28273700 | 29267241 | 29414466 |
| 19311518 | 20410066 | 21973016 | 22693848 | 23394410 | 24630946 | 27053406 | 28274005 | 29267244 | 29414677 |
| 19311685 | 20410365 | 21973115 | 22694129 | 23396023 | 24631267 | 27053734 | 28274464 | 29267245 | 29414923 |
| 19311784 | 20410544 | 21973766 | 22694434 | 23396101 | 24632692 | 27053844 | 28274694 | 29267251 | 29415187 |
| 19311906 | 20411003 | 21973845 | 22694466 | 23396170 | 24633851 | 27053996 | 28275402 | 29267260 | 29415225 |
| 19312362 | 20411774 | 21974270 | 22694837 | 23396483 | 24637010 | 27054746 | 28275435 | 29267270 | 29415902 |
| 19312537 | 20414008 | 21974907 | 22694950 | 23397284 | 24637191 | 27055806 | 28276073 | 29267283 | 29416131 |
| 19312655 | 20414156 | 21975001 | 22695144 | 23398140 | 24637235 | 27056370 | 28276361 | 29267285 | 29416239 |
| 19312872 | 20414397 | 21975281 | 22695263 | 23398566 | 24637702 | 27056635 | 28277003 | 29267286 | 29416269 |
| 19313245 | 20415022 | 21975435 | 22695366 | 23398982 | 24638914 | 27056674 | 28277106 | 29267295 | 29416597 |
| 19313260 | 20415531 | 21975460 | 22695480 | 23398996 | 24638934 | 27056808 | 28277239 | 29267301 | 29417060 |
| 19313560 | 20415606 | 21975588 | 22695481 | 23399067 | 24639845 | 27057275 | 28277300 | 29267312 | 29417174 |
| 19313761 | 20415932 | 21975633 | 22695527 | 23399330 | 24640341 | 27057584 | 28277428 | 29267316 | 29417317 |
| 19313873 | 20416328 | 21975670 | 22695666 | 23399374 | 24641470 | 27057886 | 28277726 | 29267323 | 29418928 |
| 19313976 | 20416533 | 21976231 | 22695885 | 23399492 | 24642404 | 27058398 | 28277851 | 29267326 | 29421088 |
| 19314157 | 20416646 | 21976319 | 22696157 | 23400444 | 24642477 | 27058846 | 28277974 | 29267328 | 29421173 |
| 19314222 | 20417570 | 21976394 | 22696290 | 23400492 | 24644494 | 27059105 | 28278091 | 29267331 | 29421997 |
| 19314249 | 20418020 | 21976513 | 22696485 | 23400857 | 24644637 | 27059576 | 28278096 | 29267335 | 29423374 |
| 19316114 | 20418884 | 21976657 | 22696828 | 23401418 | 24645034 | 27059767 | 28278240 | 29267337 | 29423510 |
| 19317180 | 20419146 | 21976711 | 22696974 | 23402122 | 24645566 | 27060541 | 28278311 | 29267338 | 29423799 |
| 19317308 | 20419424 | 21976882 | 22697572 | 23402729 | 24646109 | 27060651 | 28278661 | 29267340 | 29424191 |
| 19317683 | 20419487 | 21977087 | 22697690 | 23402770 | 24646341 | 27060882 | 28279263 | 29267341 | 29424833 |
| 19318403 | 20419810 | 21977158 | 22697829 | 23403240 | 24648761 | 27061130 | 28279380 | 29267344 | 29424939 |
| 19318999 | 20419916 | 21977200 | 22697974 | 23403501 | 24649879 | 27061287 | 28279712 | 29267348 | 29425188 |
| 19319169 | 20422516 | 21977358 | 22698089 | 23403583 | 24650696 | 27061491 | 28279922 | 29267361 | 29425402 |
| 19319364 | 20422706 | 21978044 | 22698219 | 23403772 | 24650728 | 27061530 | 28280041 | 29267379 | 29425945 |
| 19319516 | 20426484 | 21978049 | 22698286 | 23403886 | 24652396 | 27062060 | 28280146 | 29267380 | 29425973 |
| 19320100 | 20426520 | 21978136 | 22698302 | 23404957 | 24653238 | 27062675 | 28280450 | 29267414 | 29426343 |
| 19320386 | 20426576 | 21978279 | 22698353 | 23405166 | 24654243 | 27062848 | 28280573 | 29267437 | 29426425 |
| 19320569 | 20426617 | 21978281 | 22698671 | 23405598 | 24654248 | 27063133 | 28280684 | 29267451 | 29427004 |
| 19320760 | 20427154 | 21978386 | 22698866 | 23405650 | 24655370 | 27063261 | 28280724 | 29267465 | 29427167 |
| 19321305 | 20427260 | 21978605 | 22698891 | 23407313 | 24655801 | 27063269 | 28280749 | 29267466 | 29427530 |
| 19321500 | 20427705 | 21978919 | 22699510 | 23408046 | 24656393 | 27063338 | 28280817 | 29267467 | 29427676 |
| 19321870 | 20427745 | 21979046 | 22699762 | 23408082 | 24658113 | 27063579 | 28280887 | 29267483 | 29428103 |
| 19322697 | 20428037 | 21980268 | 22699794 | 23408164 | 24658239 | 27063946 | 28281423 | 29267488 | 29428289 |
| 19323124 | 20428121 | 21980457 | 22699978 | 23408179 | 24658758 | 27064013 | 28281514 | 29267501 | 29428430 |
| 19323478 | 20428410 | 21981297 | 22700457 | 23408413 | 24659408 | 27064405 | 28281792 | 29267517 | 29429130 |
| 19323501 | 20429586 | 21981362 | 22700813 | 23408432 | 24660199 | 27064709 | 28282150 | 29267519 | 29429219 |
| 19324192 | 20430121 | 21981389 | 22701481 | 23408794 | 24660832 | 27065031 | 28282244 | 29267520 | 29430433 |
| 19324560 | 20430174 | 21981521 | 22701537 | 23409093 | 24661950 | 27065837 | 28282322 | 29267524 | 29430468 |
| 19324702 | 20430205 | 21982472 | 22701670 | 23409207 | 24663722 | 27067034 | 28282437 | 29267538 | 29430932 |
| 19324986 | 20430892 | 21982619 | 22701960 | 23409210 | 24664196 | 27067371 | 28282456 | 29267544 | 29431277 |
| 19325561 | 20431431 | 21982681 | 22702091 | 23409392 | 24664261 | 27067379 | 28283100 | 29267565 | 29431489 |
| 19325839 | 20431529 | 21982954 | 22702291 | 23409627 | 24667288 | 27067576 | 28283482 | 29267577 | 29432321 |
| 19325895 | 20431675 | 21983045 | 22702366 | 23410365 | 24668204 | 27068358 | 28284224 | 29267580 | 29432513 |
| 19326741 | 20433242 | 21983184 | 22702607 | 23410554 | 24669646 | 27068577 | 28284419 | 29267589 | 29433191 |
| 19327059 | 20433547 | 21983334 | 22703522 | 23410625 | 24669720 | 27068938 | 28285426 | 29267590 | 29433203 |
| 19327415 | 20434101 | 21983566 | 22703622 | 23411939 | 24670444 | 27069013 | 28285559 | 29267596 | 29433234 |
| 19327485 | 20434197 | 21983824 | 22703839 | 23412117 | 24672503 | 27069111 | 28285777 | 29267603 | 29433286 |
| 19327971 | 20435433 | 21983872 | 22704081 | 23412417 | 24672767 | 27070137 | 28286374 | 29267605 | 29433642 |
| 19328198 | 20435728 | 21984177 | 22704096 | 23412736 | 24673013 | 27070150 | 28286627 | 29267635 | 29433697 |
| 19328681 | 20436496 | 21984197 | 22704158 | 23412856 | 24673240 | 27070690 | 28286951 | 29267644 | 29434010 |
| 19329122 | 20437700 | 21984225 | 22704188 | 23412911 | 24673530 | 27072151 | 28287248 | 29267660 | 29434103 |
| 19329156 | 20438289 | 21984330 | 22704267 | 23413911 | 24674187 | 27072424 | 28287948 | 29267662 | 29434369 |
| 19329548 | 20438389 | 21984346 | 22704422 | 23414302 | 24675350 | 27073306 | 28288715 | 29267665 | 29435841 |
| 19330488 | 20438782 | 21985193 | 22704537 | 23414470 | 24676915 | 27074321 | 28288985 | 29267671 | 29436648 |
| 19330830 | 20439309 | 21985728 | 22704577 | 23414471 | 24677235 | 27075115 | 28288999 | 29267675 | 29437246 |
| 19331517 | 20439534 | 21985815 | 22705057 | 23414791 | 24677480 | 27075503 | 28289312 | 29267678 | 29437922 |
| 19331665 | 20440241 | 21985920 | 22705294 | 23415259 | 24680266 | 27076489 | 28289453 | 29267687 | 29438066 |
| 19331760 | 20441082 | 21986017 | 22705371 | 23415327 | 24682971 | 27077139 | 28289531 | 29267710 | 29438617 |
| 19332064 | 20441997 | 21986178 | 22705611 | 23415363 | 24686044 | 27077230 | 28289539 | 29267723 | 29438661 |
| 19332103 | 20442315 | 21986766 | 22705970 | 23416040 | 24686440 | 27077620 | 28289871 | 29267730 | 29438733 |
| 19332126 | 20442745 | 21986989 | 22706787 | 23416209 | 24687078 | 27078089 | 28289927 | 29267733 | 29438903 |
| 19332213 | 20443265 | 21987155 | 22706938 | 23416335 | 24688885 | 27079099 | 28289988 | 29267738 | 29439173 |
| 19332401 | 20443511 | 21987432 | 22706970 | 23416539 | 24689653 | 27079955 | 28290711 | 29267739 | 29440674 |
| 19332533 | 20444560 | 21988110 | 22707251 | 23416754 | 24689999 | 27079971 | 28291179 | 29267748 | 29441374 |
| 19333458 | 20445817 | 21988274 | 22708547 | 23416783 | 24690335 | 27080677 | 28291324 | 29267777 | 29441963 |
| 19334319 | 20445943 | 21988315 | 22708589 | 23416944 | 24691152 | 27081644 | 28291433 | 29267781 | 29442743 |
| 19334369 | 20446245 | 21988421 | 22709073 | 23417681 | 24691717 | 27081830 | 28291687 | 29267788 | 29442840 |
| 19334903 | 20447996 | 21988838 | 22709740 | 23418070 | 24692459 | 27082935 | 28291741 | 29267796 | 29442926 |
| 19335045 | 20448841 | 21988911 | 22709778 | 23420030 | 24693604 | 27083026 | 28291820 | 29267822 | 29443101 |
| 19335556 | 20449478 | 21989599 | 22709785 | 23420257 | 24695003 | 27084098 | 28292211 | 29267823 | 29443451 |
| 19335979 | 20449868 | 21989614 | 22709971 | 23420550 | 24695153 | 27084189 | 28292517 | 29267841 | 29443763 |
| 19335997 | 20450216 | 21989645 | 22710879 | 23420606 | 24696187 | 27084264 | 28292580 | 29267867 | 29443808 |
| 19337501 | 20451041 | 21989961 | 22710991 | 23420779 | 24698623 | 27084615 | 28292758 | 29267876 | 29443829 |
| 19337714 | 20451276 | 21990135 | 22711071 | 23420805 | 24699852 | 27084693 | 28293607 | 29267878 | 29444529 |
| 19337986 | 20451584 | 21990253 | 22711200 | 23420848 | 24700130 | 27085034 | 28293662 | 29267884 | 29444679 |
| 19338094 | 20452341 | 21990941 | 22711416 | 23422789 | 24703612 | 27085047 | 28294153 | 29267894 | 29444998 |
| 19339177 | 20452900 | 21991153 | 22711462 | 23422791 | 24704174 | 27085259 | 28294369 | 29267902 | 29445181 |
| 19340413 | 20454134 | 21991433 | 22711889 | 23422905 | 24704534 | 27085442 | 28295294 | 29267906 | 29445199 |
| 19340481 | 20454195 | 21991838 | 22711901 | 23423229 | 24705501 | 27085833 | 28295935 | 29267909 | 29446332 |
| 19341052 | 20455147 | 21991960 | 22712408 | 23423684 | 24706143 | 27085973 | 28296747 | 29267913 | 29446991 |
| 19341075 | 20455177 | 21992157 | 22712515 | 23423916 | 24706241 | 27086318 | 28296821 | 29267936 | 29447501 |
| 19342085 | 20455250 | 21992448 | 22712737 | 23423996 | 24709862 | 27086323 | 28297240 | 29267950 | 29447743 |
| 19342415 | 20456779 | 21992450 | 22712760 | 23424112 | 24710273 | 27086630 | 28297617 | 29267963 | 29448180 |
| 19342647 | 20456811 | 21992468 | 22713151 | 23424932 | 24710424 | 27086805 | 28298330 | 29267966 | 29448264 |
| 19343095 | 20457123 | 21992472 | 22713452 | 23424950 | 24710898 | 27087274 | 28299039 | 29267974 | 29448363 |
| 19343298 | 20457548 | 21992580 | 22713484 | 23424996 | 24711450 | 27087589 | 28300542 | 29267985 | 29448772 |
| 19343650 | 20457662 | 21992608 | 22713771 | 23425118 | 24713038 | 27087596 | 28301074 | 29267995 | 29448984 |
| 19343729 | 20458359 | 21992743 | 22713803 | 23425989 | 24714618 | 27087840 | 28301510 | 29268007 | 29449874 |
| 19344246 | 20458373 | 21992997 | 22713805 | 23426163 | 24714826 | 27088658 | 28301582 | 29268010 | 29450236 |
| 19344708 | 20458912 | 21993433 | 22714748 | 23426476 | 24715040 | 27088666 | 28301672 | 29268011 | 29450417 |
| 19344730 | 20460223 | 21993505 | 22715010 | 23426845 | 24715187 | 27088825 | 28301778 | 29268015 | 29450422 |
| 19344882 | 20460345 | 21993666 | 22715033 | 23426997 | 24715939 | 27088907 | 28301850 | 29268017 | 29450635 |
| 19345301 | 20460918 | 21993978 | 22715051 | 23427174 | 24716400 | 27089201 | 28302346 | 29268021 | 29451111 |
| 19345960 | 20461058 | 21994350 | 22715821 | 23427523 | 24716452 | 27091079 | 28302516 | 29268030 | 29451324 |
| 19346441 | 20462676 | 21994369 | 22715835 | 23428805 | 24716517 | 27091345 | 28302869 | 29268035 | 29452648 |
| 19346669 | 20463190 | 21994802 | 22716044 | 23428862 | 24717975 | 27091604 | 28302983 | 29268048 | 29453485 |
| 19347636 | 20464016 | 21995236 | 22716621 | 23429024 | 24719344 | 27091640 | 28303699 | 29268052 | 29453595 |
| 19348059 | 20464052 | 21995452 | 22717059 | 23429072 | 24720927 | 27091752 | 28304220 | 29268053 | 29453993 |
| 19348215 | 20464239 | 21995504 | 22717145 | 23429081 | 24721546 | 27091942 | 28304755 | 29268058 | 29454086 |
| 19348777 | 20464806 | 21995643 | 22717267 | 23429325 | 24723928 | 27092067 | 28304800 | 29268063 | 29454375 |
| 19349045 | 20465552 | 21996043 | 22717541 | 23429330 | 24723957 | 27092070 | 28304900 | 29268074 | 29454537 |
| 19349783 | 20465947 | 21996089 | 22717568 | 23429439 | 24725453 | 27093114 | 28305283 | 29268081 | 29454546 |
| 19351098 | 20466442 | 21996255 | 22718258 | 23430127 | 24727383 | 27093310 | 28305574 | 29268085 | 29454738 |
| 19351149 | 20466664 | 21996326 | 22718369 | 23430369 | 24728352 | 27094372 | 28305591 | 29268096 | 29454913 |
| 19351651 | 20467847 | 21996360 | 22718683 | 23430729 | 24728517 | 27095185 | 28305968 | 29268097 | 29454992 |
| 19351982 | 20468922 | 21996750 | 22718689 | 23431362 | 24730376 | 27095669 | 28306278 | 29268106 | 29455425 |
| 19352063 | 20469886 | 21997163 | 22718884 | 23431427 | 24730419 | 27096011 | 28306343 | 29268110 | 29455884 |
| 19352258 | 20470508 | 21997394 | 22718936 | 23432468 | 24730904 | 27096459 | 28306721 | 29268112 | 29456293 |
| 19353139 | 20470603 | 21997402 | 22719528 | 23433769 | 24731742 | 27097089 | 28306755 | 29268117 | 29456623 |
| 19353493 | 20471196 | 21997681 | 22719578 | 23434296 | 24733112 | 27097119 | 28306824 | 29268122 | 29456678 |
| 19353882 | 20471817 | 21997847 | 22719645 | 23434800 | 24733158 | 27097786 | 28307007 | 29268126 | 29457853 |
| 19354655 | 20471997 | 21997862 | 22719874 | 23435926 | 24733979 | 27098028 | 28307455 | 29268127 | 29458330 |
| 19355123 | 20472119 | 21998781 | 22720139 | 23436505 | 24734198 | 27098419 | 28307559 | 29268134 | 29459340 |
| 19355254 | 20473832 | 21998964 | 22720351 | 23436787 | 24735135 | 27099349 | 28308301 | 29268135 | 29459350 |
| 19355341 | 20475101 | 21999151 | 22720464 | 23437035 | 24736197 | 27099858 | 28309148 | 29268144 | 29459410 |
| 19355393 | 20475974 | 21999352 | 22720740 | 23437502 | 24737057 | 27099892 | 28309354 | 29268147 | 29460063 |
| 19355986 | 20476283 | 21999380 | 22720784 | 23438367 | 24737184 | 27101016 | 28309830 | 29268148 | 29460739 |
| 19356052 | 20476763 | 21999397 | 22721074 | 23438634 | 24738191 | 27101061 | 28310535 | 29268149 | 29461191 |
| 19356446 | 20477130 | 21999689 | 22721102 | 23438663 | 24739163 | 27102010 | 28310583 | 29268156 | 29461436 |
| 19356826 | 20478867 | 21999758 | 22721199 | 23439703 | 24739354 | 27102198 | 28310708 | 29268157 | 29461549 |
| 19357405 | 20479662 | 21999896 | 22721312 | 23439980 | 24740410 | 27102510 | 28310968 | 29268166 | 29461593 |
| 19357862 | 20480195 | 21999974 | 22721375 | 23439999 | 24741220 | 27102538 | 28311613 | 29268168 | 29462227 |
| 19358218 | 20481163 | 22000459 | 22721638 | 23440490 | 24742003 | 27103345 | 28313150 | 29268169 | 29462245 |
| 19358395 | 20481802 | 22000498 | 22721707 | 23440507 | 24742894 | 27103476 | 28314046 | 29268172 | 29462320 |
| 19358612 | 20482998 | 22001546 | 22721761 | 23441297 | 24743439 | 27104030 | 28314074 | 29268175 | 29462627 |
| 19359187 | 20484258 | 22001847 | 22722083 | 23441344 | 24745935 | 27104414 | 28314180 | 29268176 | 29462796 |
| 19359292 | 20485121 | 22002116 | 22722133 | 23442245 | 24746021 | 27104614 | 28314466 | 29268186 | 29463022 |
| 19359405 | 20485330 | 22002194 | 22722184 | 23442644 | 24747556 | 27104747 | 28314600 | 29268212 | 29463279 |
| 19359500 | 20485343 | 22002206 | 22722308 | 23442681 | 24749673 | 27105211 | 28314700 | 29268229 | 29463574 |
| 19360083 | 20485650 | 22002561 | 22722421 | 23442921 | 24750699 | 27105561 | 28315620 | 29268231 | 29464030 |
| 19360813 | 20485844 | 22002683 | 22722432 | 23443031 | 24752439 | 27105916 | 28316116 | 29268233 | 29464197 |
| 19361152 | 20485975 | 22002783 | 22722582 | 23443698 | 24757398 | 27105989 | 28316349 | 29268234 | 29464966 |
| 19361187 | 20487468 | 22002870 | 22722698 | 23444312 | 24760176 | 27106070 | 28317168 | 29268239 | 29465754 |
| 19361698 | 20487769 | 22002968 | 22722803 | 23444632 | 24760216 | 27106929 | 28317454 | 29268258 | 29465943 |
| 19361984 | 20487973 | 22003199 | 22723067 | 23444751 | 24760656 | 27106979 | 28317476 | 29268267 | 29467016 |
| 19361990 | 20489640 | 22003248 | 22723090 | 23445049 | 24760701 | 27107072 | 28317478 | 29268270 | 29467189 |
| 19362181 | 20490299 | 22003269 | 22723123 | 23445478 | 24760750 | 27107341 | 28317981 | 29268271 | 29467200 |
| 19362500 | 20490425 | 22003604 | 22723577 | 23446428 | 24760876 | 27107491 | 28318081 | 29268274 | 29468547 |
| 19362628 | 20490856 | 22003719 | 22723626 | 23446516 | 24761745 | 27108891 | 28318118 | 29268279 | 29468905 |
| 19362736 | 20491065 | 22004068 | 22723750 | 23446652 | 24762342 | 27108960 | 28318617 | 29268283 | 29468951 |
| 19362849 | 20491701 | 22004331 | 22724251 | 23447107 | 24763349 | 27109464 | 28318629 | 29268287 | 29469099 |
| 19362959 | 20491816 | 22004349 | 22724339 | 23447516 | 24763784 | 27109494 | 28318679 | 29268315 | 29470289 |
| 19363209 | 20491830 | 22005059 | 22724399 | 23447586 | 24763891 | 27109822 | 28319418 | 29268322 | 29470569 |
| 19363588 | 20491862 | 22005225 | 22724487 | 23447960 | 24764891 | 27109987 | 28319606 | 29268324 | 29470602 |
| 19363873 | 20491993 | 22005763 | 22724710 | 23448088 | 24765498 | 27110370 | 28321025 | 29268342 | 29471786 |
| 19363997 | 20492495 | 22006031 | 22724770 | 23448385 | 24765825 | 27111446 | 28321049 | 29268343 | 29472020 |
| 19364314 | 20492708 | 22006210 | 22724806 | 23448488 | 24766083 | 27111493 | 28321185 | 29268353 | 29472327 |
| 19364585 | 20493127 | 22006676 | 22724924 | 23448806 | 24769671 | 27111766 | 28321255 | 29268360 | 29472471 |
| 19364724 | 20493354 | 22007118 | 22724963 | 23449012 | 24770766 | 27111832 | 28321495 | 29268379 | 29472847 |
| 19365693 | 20493366 | 22007322 | 22725289 | 23449176 | 24772452 | 27112225 | 28322588 | 29268383 | 29473173 |
| 19365736 | 20493610 | 22007426 | 22725302 | 23449242 | 24773410 | 27112556 | 28323535 | 29268384 | 29473434 |
| 19366299 | 20494147 | 22007448 | 22725512 | 23449308 | 24774421 | 27113968 | 28323783 | 29268387 | 29474545 |
| 19366650 | 20495601 | 22007944 | 22725675 | 23449631 | 24775315 | 27114967 | 28324178 | 29268392 | 29474677 |
| 19366934 | 20495669 | 22007976 | 22725986 | 23449832 | 24776546 | 27115456 | 28324413 | 29268402 | 29475381 |
| 19367330 | 20495780 | 22008261 | 22726087 | 23450990 | 24776577 | 27116459 | 28324996 | 29268404 | 29475746 |
| 19367642 | 20496101 | 22008634 | 22726391 | 23451345 | 24778073 | 27116467 | 28325430 | 29268413 | 29476113 |
| 19367696 | 20496890 | 22008730 | 22726628 | 23451683 | 24778248 | 27117323 | 28325631 | 29268423 | 29476870 |
| 19367932 | 20497396 | 22008772 | 22726793 | 23451862 | 24778408 | 27117354 | 28325932 | 29268441 | 29476952 |
| 19367992 | 20497403 | 22009040 | 22727170 | 23452355 | 24780274 | 27117966 | 28326031 | 29268446 | 29478017 |
| 19368000 | 20497440 | 22009297 | 22727183 | 23452427 | 24781860 | 27118816 | 28326713 | 29268452 | 29478404 |
| 19369291 | 20498511 | 22009536 | 22727291 | 23452720 | 24782661 | 27118841 | 28327084 | 29268459 | 29478492 |
| 19369367 | 20498839 | 22009678 | 22727642 | 23453609 | 24782705 | 27120218 | 28327391 | 29268463 | 29479330 |
| 19369593 | 20499321 | 22010150 | 22728004 | 23455059 | 24783410 | 27121028 | 28328001 | 29268470 | 29479559 |
| 19369634 | 20500909 | 22010161 | 22728154 | 23455087 | 24783659 | 27121267 | 28328077 | 29268476 | 29479598 |
| 19369782 | 20502179 | 22010760 | 22728260 | 23455103 | 24784179 | 27121481 | 28328518 | 29268484 | 29480006 |
| 19369811 | 20502386 | 22010763 | 22728390 | 23455482 | 24785963 | 27121558 | 28328961 | 29268488 | 29481270 |
| 19369933 | 20502398 | 22011564 | 22728652 | 23455637 | 24787377 | 27121857 | 28329155 | 29268501 | 29481517 |
| 19370338 | 20503409 | 22011690 | 22728715 | 23455707 | 24787715 | 27122427 | 28329393 | 29268503 | 29481826 |
| 19370906 | 20503746 | 22011789 | 22728928 | 23456050 | 24787775 | 27123111 | 28330127 | 29268514 | 29482132 |
| 19371140 | 20504145 | 22011858 | 22729160 | 23456074 | 24788431 | 27123568 | 28330407 | 29268523 | 29482804 |
| 19371273 | 20504424 | 22011871 | 22729368 | 23456687 | 24789276 | 27124089 | 28330789 | 29268525 | 29482913 |
| 19372619 | 20504723 | 22012011 | 22729645 | 23457082 | 24790979 | 27125869 | 28331302 | 29268534 | 29483120 |
| 19372828 | 20505214 | 22012025 | 22729911 | 23457297 | 24791184 | 27126084 | 28331519 | 29268547 | 29483251 |
| 19372864 | 20505319 | 22012370 | 22729986 | 23458045 | 24791231 | 27126534 | 28331727 | 29268549 | 29483350 |
| 19373328 | 20505624 | 22012444 | 22730050 | 23458702 | 24792075 | 27126587 | 28331873 | 29268552 | 29484107 |
| 19373573 | 20505913 | 22012468 | 22730176 | 23458905 | 24792672 | 27126959 | 28331975 | 29268564 | 29484583 |
| 19374380 | 20506518 | 22012530 | 22730267 | 23459036 | 24792742 | 27126997 | 28332126 | 29268569 | 29484732 |
| 19374902 | 20506876 | 22012665 | 22730380 | 23459143 | 24796285 | 27127740 | 28332131 | 29268601 | 29485508 |
| 19375347 | 20507665 | 22012694 | 22730570 | 23459515 | 24796353 | 27127829 | 28332431 | 29268609 | 29485866 |
| 19375481 | 20509340 | 22012707 | 22731067 | 23459652 | 24796581 | 27127859 | 28333092 | 29268610 | 29486104 |
| 19376029 | 20509479 | 22013751 | 22731242 | 23460115 | 24797315 | 27127901 | 28333396 | 29268612 | 29486296 |
| 19376080 | 20509844 | 22013908 | 22731476 | 23460352 | 24797768 | 27128114 | 28333702 | 29268629 | 29487773 |
| 19376303 | 20510516 | 22013970 | 22731554 | 23461065 | 24797952 | 27129807 | 28334009 | 29268641 | 29488287 |
| 19376306 | 20511561 | 22014043 | 22731585 | 23461646 | 24798279 | 27130221 | 28334112 | 29268643 | 29488652 |
| 19376316 | 20512478 | 22014280 | 22731616 | 23462063 | 24799685 | 27130861 | 28334520 | 29268648 | 29488920 |
| 19376469 | 20512501 | 22014477 | 22731976 | 23462241 | 24799893 | 27131133 | 28334721 | 29268651 | 29489451 |
| 19377428 | 20512711 | 22014644 | 22732039 | 23462301 | 24800933 | 27131718 | 28335631 | 29268664 | 29489895 |
| 19377531 | 20513108 | 22015158 | 22732061 | 23462690 | 24801511 | 27132467 | 28335901 | 29268670 | 29491891 |
| 19377636 | 20513516 | 22015164 | 22732693 | 23463135 | 24801965 | 27132968 | 28337013 | 29268679 | 29492263 |
| 19378540 | 20514207 | 22015318 | 22733032 | 23463223 | 24802606 | 27133789 | 28337115 | 29268691 | 29493481 |
| 19379194 | 20515423 | 22015789 | 22733108 | 23463330 | 24802898 | 27134120 | 28337250 | 29268694 | 29494015 |
| 19379453 | 20515537 | 22016117 | 22733221 | 23463337 | 24803243 | 27134532 | 28337361 | 29268710 | 29494767 |
| 19379707 | 20516140 | 22017074 | 22733255 | 23463873 | 24804685 | 27134908 | 28337385 | 29268730 | 29494854 |
| 19380242 | 20516591 | 22017155 | 22733408 | 23464179 | 24805037 | 27135234 | 28337559 | 29268731 | 29495678 |
| 19380315 | 20516991 | 22017169 | 22733774 | 23464321 | 24805959 | 27135840 | 28337683 | 29268732 | 29496324 |
| 19380337 | 20517562 | 22017189 | 22733868 | 23464720 | 24809075 | 27136399 | 28337881 | 29268745 | 29496435 |
| 19380618 | 20518577 | 22017344 | 22733898 | 23464787 | 24809748 | 27136865 | 28338556 | 29268756 | 29496588 |
| 19380666 | 20519180 | 22017988 | 22734366 | 23465350 | 24811687 | 27138590 | 28338798 | 29268763 | 29496657 |
| 19380801 | 20519374 | 22019070 | 22734423 | 23465568 | 24813054 | 27138757 | 28340148 | 29268765 | 29497257 |
| 19381246 | 20519485 | 22019084 | 22734517 | 23465781 | 24816902 | 27139843 | 28340550 | 29268775 | 29497419 |
| 19381287 | 20520138 | 22019514 | 22734603 | 23465985 | 24817547 | 27139863 | 28340732 | 29268782 | 29497566 |
| 19381757 | 20520942 | 22019699 | 22734830 | 23466113 | 24818057 | 27140081 | 28341053 | 29268786 | 29498177 |
| 19381801 | 20522867 | 22019764 | 22735264 | 23466134 | 24820731 | 27140279 | 28341374 | 29268793 | 29498400 |
| 19381989 | 20522975 | 22019956 | 22735906 | 23466715 | 24820740 | 27140779 | 28341679 | 29268803 | 29499168 |
| 19382214 | 20523969 | 22020401 | 22735938 | 23466933 | 24821675 | 27140824 | 28341937 | 29268805 | 29499332 |
| 19382437 | 20525684 | 22020978 | 22737291 | 23467395 | 24822005 | 27141193 | 28342209 | 29268817 | 29499398 |
| 19383013 | 20526612 | 22021702 | 22737371 | 23467492 | 24822709 | 27143060 | 28342383 | 29268845 | 29499702 |
| 19385889 | 20526766 | 22022009 | 22737643 | 23467558 | 24822879 | 27143490 | 28342390 | 29268853 | 29499859 |
| 19385988 | 20527193 | 22022011 | 22737744 | 23467950 | 24823761 | 27143522 | 28343055 | 29268862 | 29500099 |
| 19386006 | 20527403 | 22022592 | 22737989 | 23467971 | 24825127 | 27143615 | 28343074 | 29268872 | 29500681 |
| 19386455 | 20527679 | 22022595 | 22738030 | 23468044 | 24825350 | 27143755 | 28343534 | 29268877 | 29502798 |
| 19386493 | 20528734 | 22022995 | 22738164 | 23468058 | 24826706 | 27143823 | 28343627 | 29268884 | 29503017 |
| 19386499 | 20530017 | 22023330 | 22738807 | 23468157 | 24827322 | 27144008 | 28344142 | 29268885 | 29504449 |
| 19387028 | 20530975 | 22023480 | 22739091 | 23468411 | 24827490 | 27144244 | 28344825 | 29268890 | 29504981 |
| 19387072 | 20531487 | 22023738 | 22739236 | 23468748 | 24828235 | 27145120 | 28344858 | 29268894 | 29505040 |
| 19387227 | 20531691 | 22024218 | 22739361 | 23468904 | 24828624 | 27145768 | 28344874 | 29268909 | 29506352 |
| 19387318 | 20531891 | 22024381 | 22739559 | 23469809 | 24829093 | 27146311 | 28345935 | 29268923 | 29506851 |
| 19387471 | 20532179 | 22024391 | 22739638 | 23470447 | 24829132 | 27146351 | 28346715 | 29268926 | 29507350 |
| 19387587 | 20532328 | 22024477 | 22740462 | 23470688 | 24829609 | 27146363 | 28346892 | 29268929 | 29508782 |
| 19387748 | 20533183 | 22024603 | 22740749 | 23470979 | 24830303 | 27146372 | 28346926 | 29268936 | 29508830 |
| 19387977 | 20533414 | 22024834 | 22740765 | 23471143 | 24831749 | 27146850 | 28347149 | 29268940 | 29509450 |
| 19388252 | 20533602 | 22025180 | 22741617 | 23471274 | 24832725 | 27148017 | 28348196 | 29268946 | 29509469 |
| 19388326 | 20533890 | 22025758 | 22741848 | 23472379 | 24833180 | 27148563 | 28348704 | 29268948 | 29510269 |
| 19388670 | 20535145 | 22025977 | 22741911 | 23472471 | 24833417 | 27148933 | 28349368 | 29268953 | 29510450 |
| 19388779 | 20535509 | 22026214 | 22741996 | 23472622 | 24835974 | 27149116 | 28350139 | 29268966 | 29510799 |
| 19388991 | 20535930 | 22026987 | 22742012 | 23472695 | 24836193 | 27149703 | 28350262 | 29268967 | 29510836 |
| 19389280 | 20536287 | 22027112 | 22742076 | 23473096 | 24836431 | 27150211 | 28350664 | 29268985 | 29511504 |
| 19389291 | 20536837 | 22027301 | 22742383 | 23473356 | 24837389 | 27150631 | 28350839 | 29268996 | 29511514 |
| 19389357 | 20537018 | 22027553 | 22742732 | 23473392 | 24837472 | 27150756 | 28351089 | 29269005 | 29511530 |
| 19389677 | 20537554 | 22027681 | 22743007 | 23473962 | 24837921 | 27150786 | 28351176 | 29269006 | 29511774 |
| 19389916 | 20537990 | 22027702 | 22743293 | 23474351 | 24838987 | 27150968 | 28352150 | 29269009 | 29512071 |
| 19390075 | 20538443 | 22028170 | 22743475 | 23474409 | 24840782 | 27151163 | 28352346 | 29269017 | 29513353 |
| 19390262 | 20538467 | 22028316 | 22743819 | 23474452 | 24841543 | 27151621 | 28352474 | 29269026 | 29513885 |
| 19390587 | 20538805 | 22028689 | 22744340 | 23474993 | 24841964 | 27151706 | 28352548 | 29269037 | 29514267 |
| 19390621 | 20539445 | 22028724 | 22744464 | 23475081 | 24842437 | 27152352 | 28352585 | 29269063 | 29514402 |
| 19391076 | 20540533 | 22028867 | 22744465 | 23475435 | 24842927 | 27152482 | 28352838 | 29269068 | 29514600 |
| 19391166 | 20541825 | 22029546 | 22744937 | 23475699 | 24844666 | 27153062 | 28353137 | 29269083 | 29515595 |
| 19391485 | 20542588 | 22029603 | 22745169 | 23476750 | 24846445 | 27153176 | 28353298 | 29269092 | 29515670 |
| 19391651 | 20542923 | 22029791 | 22745230 | 23476880 | 24846492 | 27153492 | 28353660 | 29269095 | 29516019 |
| 19392570 | 20543041 | 22029914 | 22745543 | 23477005 | 24846765 | 27153983 | 28355847 | 29269104 | 29516671 |
| 19392908 | 20543521 | 22030678 | 22745758 | 23477205 | 24846791 | 27154192 | 28356028 | 29269111 | 29516803 |
| 19393037 | 20543839 | 22031400 | 22745798 | 23477770 | 24848179 | 27154278 | 28356251 | 29269112 | 29517271 |
| 19393386 | 20543936 | 22031562 | 22746013 | 23480156 | 24848339 | 27154704 | 28356623 | 29269116 | 29517526 |
| 19394405 | 20544680 | 22031915 | 22747251 | 23480336 | 24850812 | 27155040 | 28358085 | 29269126 | 29517699 |
| 19395101 | 20544957 | 22032266 | 22747293 | 23480432 | 24850816 | 27156684 | 28358611 | 29269133 | 29517773 |
| 19395144 | 20545207 | 22032475 | 22747777 | 23480499 | 24850892 | 27157104 | 28359231 | 29269135 | 29517922 |
| 19395289 | 20546189 | 22032578 | 22747807 | 23481083 | 24851574 | 27157401 | 28359469 | 29269137 | 29518132 |
| 19395448 | 20548381 | 22032860 | 22748009 | 23481239 | 24851865 | 27159444 | 28359532 | 29269149 | 29518557 |
| 19395645 | 20548390 | 22032918 | 22748093 | 23481312 | 24852646 | 27159813 | 28359665 | 29269160 | 29519070 |
| 19395964 | 20548601 | 22033047 | 22748094 | 23481821 | 24853432 | 27159884 | 28360091 | 29269167 | 29519216 |
| 19396369 | 20549166 | 22034061 | 22748959 | 23482450 | 24854099 | 27160276 | 28360127 | 29269177 | 29520088 |
| 19396718 | 20549900 | 22034231 | 22749037 | 23482879 | 24855209 | 27160491 | 28360241 | 29269183 | 29521243 |
| 19397239 | 20550847 | 22034319 | 22749330 | 23483102 | 24859512 | 27160872 | 28360458 | 29269184 | 29521754 |
| 19398533 | 20550921 | 22034533 | 22749356 | 23483109 | 24859682 | 27161032 | 28360609 | 29269185 | 29522378 |
| 19399099 | 20551017 | 22035024 | 22749952 | 23483357 | 24859984 | 27161691 | 28361244 | 29269193 | 29523563 |
| 19399666 | 20551837 | 22035093 | 22749982 | 23483397 | 24860534 | 27161872 | 28361386 | 29269200 | 29524303 |
| 19399834 | 20552950 | 22035514 | 22750033 | 23483666 | 24861257 | 27161883 | 28361396 | 29269210 | 29524436 |
| 19400309 | 20553064 | 22035681 | 22750035 | 23483946 | 24862062 | 27162718 | 28363254 | 29269211 | 29525403 |
| 19400487 | 20553399 | 22035815 | 22750215 | 23484057 | 24862772 | 27162756 | 28363644 | 29269222 | 29525456 |
| 19400793 | 20554630 | 22035827 | 22750800 | 23484484 | 24866091 | 27163021 | 28363789 | 29269228 | 29525536 |
| 19401181 | 20555467 | 22036072 | 22750874 | 23484492 | 24866586 | 27163636 | 28363882 | 29269232 | 29526056 |
| 19401326 | 20556045 | 22036111 | 22750905 | 23484525 | 24867107 | 27163734 | 28364397 | 29269234 | 29526516 |
| 19401377 | 20558041 | 22036224 | 22750907 | 23485699 | 24867207 | 27163882 | 28364625 | 29269242 | 29526681 |
| 19401425 | 20559582 | 22036230 | 22751160 | 23486072 | 24869652 | 27165172 | 28365305 | 29269258 | 29528168 |
| 19401438 | 20561206 | 22036424 | 22751197 | 23486830 | 24871265 | 27165204 | 28365710 | 29269259 | 29528944 |
| 19401456 | 20562088 | 22036496 | 22751201 | 23487223 | 24873729 | 27166390 | 28365999 | 29269262 | 29529087 |
| 19401519 | 20563493 | 22036574 | 22751245 | 23487246 | 24873844 | 27167517 | 28366910 | 29269266 | 29530223 |
| 19402282 | 20563893 | 22037219 | 22751285 | 23487701 | 24874388 | 27167803 | 28367178 | 29269269 | 29530303 |
| 19402357 | 20564754 | 22037488 | 22751425 | 23488041 | 24875214 | 27167994 | 28367220 | 29269295 | 29530936 |
| 19402363 | 20565281 | 22037994 | 22751542 | 23488617 | 24875586 | 27168009 | 28367294 | 29269297 | 29531148 |
| 19402512 | 20566036 | 22038139 | 22751750 | 23488725 | 24875740 | 27168509 | 28367516 | 29269307 | 29531204 |
| 19403116 | 20566325 | 22038221 | 22752202 | 23488877 | 24875768 | 27169217 | 28367662 | 29269328 | 29531405 |
| 19403200 | 20568140 | 22038498 | 22752265 | 23488911 | 24875794 | 27169766 | 28367782 | 29269333 | 29531472 |
| 19403687 | 20568655 | 22038774 | 22752299 | 23489036 | 24876650 | 27170340 | 28368198 | 29269337 | 29531542 |
| 19403837 | 20569431 | 22038889 | 22752327 | 23489087 | 24876685 | 27170653 | 28369476 | 29269341 | 29531796 |
| 19403899 | 20570905 | 22039032 | 22752475 | 23489419 | 24877312 | 27171082 | 28369790 | 29269344 | 29533517 |
| 19404404 | 20571257 | 22039458 | 22752734 | 23490336 | 24877377 | 27172204 | 28370720 | 29269345 | 29535389 |
| 19405030 | 20573084 | 22039698 | 22753078 | 23490570 | 24883193 | 27172532 | 28371249 | 29269364 | 29535938 |
| 19405066 | 20573283 | 22039748 | 22753158 | 23490841 | 24884408 | 27173675 | 28371378 | 29269367 | 29536153 |
| 19405517 | 20573547 | 22040037 | 22753184 | 23491337 | 24884628 | 27174056 | 28372416 | 29269368 | 29536441 |
| 19406217 | 20573860 | 22040201 | 22753341 | 23492894 | 24885815 | 27174299 | 28372417 | 29269371 | 29536536 |
| 19406284 | 20574817 | 22040278 | 22753635 | 23493593 | 24887089 | 27174397 | 28373101 | 29269377 | 29537118 |
| 19406636 | 20575794 | 22040510 | 22753704 | 23493721 | 24887413 | 27174515 | 28373613 | 29269380 | 29537227 |
| 19408297 | 20577365 | 22041049 | 22753805 | 23494005 | 24887744 | 27174795 | 28374013 | 29269386 | 29537682 |
| 19408598 | 20577530 | 22041057 | 22753929 | 23494051 | 24888453 | 27174871 | 28374045 | 29269390 | 29537857 |
| 19409725 | 20577920 | 22041101 | 22754091 | 23494110 | 24888512 | 27175185 | 28374063 | 29269391 | 29538520 |
| 19410276 | 20577933 | 22041129 | 22754182 | 23494354 | 24888791 | 27175344 | 28375144 | 29269394 | 29538669 |
| 19410624 | 20577946 | 22041581 | 22754221 | 23494614 | 24889433 | 27175636 | 28375231 | 29269404 | 29538766 |
| 19411813 | 20578058 | 22041741 | 22754577 | 23495624 | 24889712 | 27175936 | 28375419 | 29269410 | 29539123 |
| 19412148 | 20578307 | 22042209 | 22755053 | 23498895 | 24890054 | 27176408 | 28375451 | 29269413 | 29539143 |
| 19412625 | 20578584 | 22042243 | 22755181 | 23499612 | 24890735 | 27176525 | 28375705 | 29269419 | 29539305 |
| 19412705 | 20578874 | 22042274 | 22755331 | 23499951 | 24891092 | 27177269 | 28376488 | 29269451 | 29539701 |
| 19412929 | 20579160 | 22042299 | 22755561 | 23500048 | 24893101 | 27178253 | 28377139 | 29269455 | 29540016 |
| 19413186 | 20580455 | 22042367 | 22756375 | 23500226 | 24893456 | 27178653 | 28377229 | 29269459 | 29540066 |
| 19414524 | 20581334 | 22042386 | 22756462 | 23501265 | 24894371 | 27178857 | 28377531 | 29269464 | 29540359 |
| 19414668 | 20582136 | 22042414 | 22756522 | 23501396 | 24894822 | 27179045 | 28377842 | 29269470 | 29540673 |
| 19414683 | 20582780 | 22042420 | 22756826 | 23501481 | 24894955 | 27179400 | 28379103 | 29269474 | 29540827 |
| 19414704 | 20582844 | 22042633 | 22756953 | 23502048 | 24896444 | 27179728 | 28379728 | 29269484 | 29540999 |
| 19414758 | 20583659 | 22043167 | 22757036 | 23502234 | 24897531 | 27179828 | 28379769 | 29269486 | 29542098 |
| 19415090 | 20584151 | 22043297 | 22757416 | 23502855 | 24898723 | 27180127 | 28380082 | 29269489 | 29542711 |
| 19415155 | 20584224 | 22043330 | 22757717 | 23503822 | 24900307 | 27180222 | 28381194 | 29269493 | 29542847 |
| 19415841 | 20585153 | 22043862 | 22757841 | 23504000 | 24900413 | 27180846 | 28381319 | 29269515 | 29542939 |
| 19415937 | 20586051 | 22044190 | 22758048 | 23504401 | 24900603 | 27180905 | 28381465 | 29269516 | 29544766 |
| 19416058 | 20586100 | 22044348 | 22758157 | 23504632 | 24900661 | 27181108 | 28381720 | 29269517 | 29546331 |
| 19416165 | 20586316 | 22044392 | 22758260 | 23504999 | 24900962 | 27181250 | 28381860 | 29269533 | 29546608 |
| 19416405 | 20586358 | 22044751 | 22758690 | 23505122 | 24901983 | 27182272 | 28382181 | 29269553 | 29546688 |
| 19416433 | 20587268 | 22044821 | 22758831 | 23505409 | 24904208 | 27182467 | 28382301 | 29269580 | 29546971 |
| 19416632 | 20587350 | 22044894 | 22758962 | 23506267 | 24904481 | 27182794 | 28383041 | 29269582 | 29547358 |
| 19417283 | 20587459 | 22045016 | 22759004 | 23506441 | 24904951 | 27182876 | 28383299 | 29269590 | 29547384 |
| 19417339 | 20587716 | 22045465 | 22759593 | 23506814 | 24910016 | 27183302 | 28383855 | 29269593 | 29547728 |
| 19417462 | 20587801 | 22045560 | 22759881 | 23507181 | 24910427 | 27183446 | 28384670 | 29269607 | 29547827 |
| 19417483 | 20590016 | 22045717 | 22759924 | 23507543 | 24910433 | 27184060 | 28385075 | 29269611 | 29548447 |
| 19417543 | 20590285 | 22045872 | 22759998 | 23507875 | 24910969 | 27184828 | 28385233 | 29269613 | 29548689 |
| 19418122 | 20590412 | 22046407 | 22761027 | 23507887 | 24913230 | 27185153 | 28385626 | 29269617 | 29548912 |
| 19418148 | 20590958 | 22046731 | 22761174 | 23508234 | 24913493 | 27185288 | 28387578 | 29269619 | 29549098 |
| 19418386 | 20592990 | 22048595 | 22761403 | 23508872 | 24913957 | 27185304 | 28387723 | 29269639 | 29549385 |
| 19418468 | 20593064 | 22048811 | 22761438 | 23509118 | 24914957 | 27185424 | 28388027 | 29269646 | 29549408 |
| 19418620 | 20593069 | 22049035 | 22761480 | 23509550 | 24916589 | 27185696 | 28388424 | 29269656 | 29549696 |
| 19418667 | 20593310 | 22049084 | 22761584 | 23509597 | 24917194 | 27186753 | 28388797 | 29269661 | 29550996 |
| 19418823 | 20593648 | 22049194 | 22761627 | 23510359 | 24917258 | 27186976 | 28388814 | 29269668 | 29551042 |
| 19418825 | 20593746 | 22049372 | 22761688 | 23510519 | 24917742 | 27187541 | 28389035 | 29269678 | 29552101 |
| 19419438 | 20594178 | 22049905 | 22762037 | 23510566 | 24918457 | 27187821 | 28389069 | 29269685 | 29552284 |
| 19419567 | 20596115 | 22049976 | 22762159 | 23511262 | 24918997 | 27187986 | 28389244 | 29269694 | 29552444 |
| 19419629 | 20596258 | 22050125 | 22762959 | 23511421 | 24919361 | 27188413 | 28389344 | 29269712 | 29553586 |
| 19419866 | 20596564 | 22050867 | 22762965 | 23511682 | 24919708 | 27189216 | 28389961 | 29269721 | 29553616 |
| 19419952 | 20597707 | 22051242 | 22762968 | 23511958 | 24920314 | 27189614 | 28389989 | 29269727 | 29554153 |
| 19420045 | 20597857 | 22051414 | 22763012 | 23512054 | 24924114 | 27189709 | 28390079 | 29269734 | 29554684 |
| 19420068 | 20598123 | 22051759 | 22763191 | 23512096 | 24924192 | 27189749 | 28391476 | 29269772 | 29554812 |
| 19421019 | 20599144 | 22051897 | 22763330 | 23512710 | 24924308 | 27189998 | 28391615 | 29269780 | 29556147 |
| 19421122 | 20599938 | 22052003 | 22763376 | 23512892 | 24925627 | 27190772 | 28392057 | 29269781 | 29556200 |
| 19421518 | 20600087 | 22052303 | 22763689 | 23513174 | 24926062 | 27191517 | 28392219 | 29269793 | 29556557 |
| 19421816 | 20600192 | 22052606 | 22763722 | 23513255 | 24926174 | 27191842 | 28392314 | 29269800 | 29557008 |
| 19421882 | 20600225 | 22052725 | 22763815 | 23513771 | 24927021 | 27193023 | 28392800 | 29269803 | 29557018 |
| 19421888 | 20600253 | 22053401 | 22763834 | 23514274 | 24927171 | 27193050 | 28394172 | 29269807 | 29557206 |
| 19423045 | 20600638 | 22053511 | 22764146 | 23514276 | 24927213 | 27193120 | 28394390 | 29269808 | 29557249 |
| 19423335 | 20601611 | 22053545 | 22764462 | 23515461 | 24928263 | 27193598 | 28395234 | 29269827 | 29557448 |
| 19423489 | 20601744 | 22053928 | 22764493 | 23515964 | 24928484 | 27194124 | 28395308 | 29269862 | 29557560 |
| 19423641 | 20602420 | 22054095 | 22764508 | 23516592 | 24929414 | 27194294 | 28395486 | 29269863 | 29557871 |
| 19423682 | 20602924 | 22054503 | 22764578 | 23516650 | 24929655 | 27194497 | 28396200 | 29269866 | 29558091 |
| 19423938 | 20603135 | 22054633 | 22764680 | 23517863 | 24929697 | 27194759 | 28396571 | 29269877 | 29558701 |
| 19423954 | 20605145 | 22054960 | 22765269 | 23517940 | 24930556 | 27195532 | 28396702 | 29269884 | 29558729 |
| 19424450 | 20605640 | 22055880 | 22765645 | 23518641 | 24931214 | 27196943 | 28397745 | 29269892 | 29558796 |
| 19424475 | 20608360 | 22056081 | 22765882 | 23518856 | 24931496 | 27197557 | 28398221 | 29269895 | 29559132 |
| 19424546 | 20608461 | 22056302 | 22766215 | 23519688 | 24932111 | 27198076 | 28398316 | 29269897 | 29560241 |
| 19424602 | 20609295 | 22056381 | 22766369 | 23520223 | 24932327 | 27198097 | 28398356 | 29269902 | 29562025 |
| 19424707 | 20609323 | 22056387 | 22766454 | 23520349 | 24934722 | 27198409 | 28399283 | 29269914 | 29562547 |
| 19425279 | 20610821 | 22056414 | 22766530 | 23520364 | 24935350 | 27198501 | 28399622 | 29269916 | 29562700 |
| 19425510 | 20611061 | 22056503 | 22766546 | 23520891 | 24936624 | 27198747 | 28399883 | 29269917 | 29562883 |
| 19425809 | 20611425 | 22057224 | 22767127 | 23521616 | 24938102 | 27200569 | 28401149 | 29269933 | 29563099 |
| 19426480 | 20612530 | 22057402 | 22767254 | 23521675 | 24938296 | 27200695 | 28401453 | 29269938 | 29563849 |
| 19426953 | 20613242 | 22057600 | 22767468 | 23522134 | 24940284 | 27200838 | 28402100 | 29269945 | 29564024 |
| 19427300 | 20613289 | 22057984 | 22767715 | 23523277 | 24941615 | 27201000 | 28402177 | 29269963 | 29564146 |
| 19427666 | 20613964 | 22058069 | 22767798 | 23523892 | 24941685 | 27201088 | 28402359 | 29269989 | 29564148 |
| 19428211 | 20614574 | 22058108 | 22767825 | 23524458 | 24942039 | 27201105 | 28402524 | 29269992 | 29566208 |
| 19428493 | 20615404 | 22058418 | 22768107 | 23524754 | 24942886 | 27201635 | 28403763 | 29270003 | 29566402 |
| 19428535 | 20617480 | 22059039 | 22768233 | 23524806 | 24943072 | 27202292 | 28403886 | 29270014 | 29566648 |
| 19428668 | 20618145 | 22059212 | 22768671 | 23525713 | 24943291 | 27202608 | 28404036 | 29270019 | 29566760 |
| 19428930 | 20618607 | 22059319 | 22768928 | 23525719 | 24945450 | 27202901 | 28404431 | 29270025 | 29567004 |
| 19429075 | 20619369 | 22059454 | 22769157 | 23526072 | 24945458 | 27203170 | 28405073 | 29270055 | 29567625 |
| 19429177 | 20620816 | 22059768 | 22769250 | 23527340 | 24945857 | 27203499 | 28405114 | 29270063 | 29567944 |
| 19429266 | 20621033 | 22060138 | 22769409 | 23527683 | 24947720 | 27203663 | 28405166 | 29270070 | 29568090 |
| 19429440 | 20621295 | 22060472 | 22769854 | 23528081 | 24950708 | 27204211 | 28405245 | 29270100 | 29569575 |
| 19429760 | 20621918 | 22060854 | 22769944 | 23528415 | 24950748 | 27204949 | 28406171 | 29270104 | 29569795 |
| 19430014 | 20622356 | 22061038 | 22770233 | 23528710 | 24950883 | 27205220 | 28406196 | 29270115 | 29570667 |
| 19430023 | 20622383 | 22061122 | 22770500 | 23529289 | 24953300 | 27205495 | 28406418 | 29270117 | 29571102 |
| 19430033 | 20623377 | 22061148 | 22770503 | 23529866 | 24955285 | 27205737 | 28407031 | 29270119 | 29573461 |
| 19430061 | 20624336 | 22061342 | 22770997 | 23531085 | 24955947 | 27205930 | 28407071 | 29270124 | 29573790 |
| 19430238 | 20624493 | 22061569 | 22771017 | 23531264 | 24956086 | 27206223 | 28407378 | 29270137 | 29575098 |
| 19430255 | 20625312 | 22061989 | 22771554 | 23531581 | 24956882 | 27206261 | 28407525 | 29270141 | 29575219 |
| 19430896 | 20626640 | 22062014 | 22771689 | 23531946 | 24958598 | 27206667 | 28407829 | 29270145 | 29575430 |
| 19431244 | 20627187 | 22062128 | 22771827 | 23532635 | 24960506 | 27207184 | 28408994 | 29270146 | 29575684 |
| 19431352 | 20627433 | 22062277 | 22772083 | 23532826 | 24961965 | 27207186 | 28409104 | 29270151 | 29575862 |
| 19431487 | 20627611 | 22062397 | 22772263 | 23532924 | 24963421 | 27208181 | 28409200 | 29270152 | 29576099 |
| 19431641 | 20627620 | 22062783 | 22772631 | 23533566 | 24963544 | 27208624 | 28409740 | 29270153 | 29576370 |
| 19432140 | 20627812 | 22063021 | 22772744 | 23533645 | 24964447 | 27208897 | 28410175 | 29270156 | 29576599 |
| 19432555 | 20628576 | 22063101 | 22772916 | 23533682 | 24967494 | 27209312 | 28410584 | 29270158 | 29576622 |
| 19432771 | 20628846 | 22063810 | 22773377 | 23533770 | 24968798 | 27209418 | 28410949 | 29270163 | 29576990 |
| 19433037 | 20630128 | 22063826 | 22773641 | 23534065 | 24969052 | 27210630 | 28411001 | 29270177 | 29577219 |
| 19433183 | 20630745 | 22063979 | 22773894 | 23534216 | 24969802 | 27211646 | 28411078 | 29270183 | 29577359 |
| 19433225 | 20631536 | 22064674 | 22774469 | 23534733 | 24970264 | 27211711 | 28412320 | 29270188 | 29578088 |
| 19433233 | 20632292 | 22065131 | 22774567 | 23534935 | 24973346 | 27212271 | 28412908 | 29270200 | 29578879 |
| 19433259 | 20632386 | 22065376 | 22774587 | 23534936 | 24973939 | 27212393 | 28413507 | 29270201 | 29579508 |
| 19433748 | 20633331 | 22065395 | 22774623 | 23535752 | 24974983 | 27212539 | 28414083 | 29270205 | 29580022 |
| 19433845 | 20633453 | 22065643 | 22774677 | 23536636 | 24976739 | 27212681 | 28414532 | 29270233 | 29580103 |
| 19434051 | 20634469 | 22065811 | 22774681 | 23536912 | 24977585 | 27213336 | 28414936 | 29270254 | 29580126 |
| 19434081 | 20635342 | 22065883 | 22774832 | 23537376 | 24978178 | 27213943 | 28415097 | 29270267 | 29580164 |
| 19434085 | 20635460 | 22065961 | 22774992 | 23537627 | 24983937 | 27214022 | 28415303 | 29270281 | 29580172 |
| 19434342 | 20635550 | 22067118 | 22775257 | 23537732 | 24984301 | 27214066 | 28415602 | 29270282 | 29580642 |
| 19434382 | 20635637 | 22067897 | 22775420 | 23538022 | 24984733 | 27214495 | 28415824 | 29270292 | 29580647 |
| 19434709 | 20636648 | 22067936 | 22775482 | 23538423 | 24986051 | 27214586 | 28415838 | 29270293 | 29580726 |
| 19435410 | 20637117 | 22068959 | 22775621 | 23538671 | 24987009 | 27215174 | 28416033 | 29270294 | 29581686 |
| 19435686 | 20638498 | 22070441 | 22775770 | 23538848 | 24987409 | 27215259 | 28416488 | 29270300 | 29581986 |
| 19435813 | 20638571 | 22070516 | 22775808 | 23539054 | 24987816 | 27215647 | 28416592 | 29270301 | 29582144 |
| 19436025 | 20639117 | 22070654 | 22775927 | 23539466 | 24989154 | 27215819 | 28417426 | 29270305 | 29582188 |
| 19436457 | 20639160 | 22070673 | 22776379 | 23539586 | 24990740 | 27216050 | 28417996 | 29270307 | 29582213 |
| 19437113 | 20640963 | 22071437 | 22777111 | 23539744 | 24991291 | 27217353 | 28418220 | 29270317 | 29583885 |
| 19437433 | 20641678 | 22071666 | 22777500 | 23540442 | 24991499 | 27217408 | 28418668 | 29270328 | 29584287 |
| 19438274 | 20642643 | 22072051 | 22777552 | 23541067 | 24992039 | 27217501 | 28418863 | 29270344 | 29584480 |
| 19438970 | 20642940 | 22072294 | 22777632 | 23541559 | 24992622 | 27217608 | 28419223 | 29270361 | 29584719 |
| 19439127 | 20642984 | 22072563 | 22778245 | 23542293 | 24992864 | 27218119 | 28419548 | 29270368 | 29584964 |
| 19439445 | 20643501 | 22072669 | 22778550 | 23542509 | 24993625 | 27219080 | 28419556 | 29270371 | 29585356 |
| 19439739 | 20643537 | 22072717 | 22778724 | 23542583 | 24993703 | 27219641 | 28419679 | 29270385 | 29586379 |
| 19439783 | 20644002 | 22073687 | 22778841 | 23542746 | 24995906 | 27219726 | 28419926 | 29270386 | 29587170 |
| 19439825 | 20644680 | 22073999 | 22779056 | 23542965 | 24996738 | 27220051 | 28420494 | 29270400 | 29587310 |
| 19440190 | 20645087 | 22074204 | 22779162 | 23543046 | 24996763 | 27220101 | 28420653 | 29270401 | 29587346 |
| 19440843 | 20645206 | 22074339 | 22779209 | 23543354 | 24997643 | 27222240 | 28421271 | 29270410 | 29587454 |
| 19440862 | 20645682 | 22074371 | 22779220 | 23543466 | 24998817 | 27224417 | 28421277 | 29270427 | 29589684 |
| 19441026 | 20645718 | 22075099 | 22779365 | 23544156 | 24998927 | 27224438 | 28422127 | 29270428 | 29590248 |
| 19441405 | 20645732 | 22075132 | 22779479 | 23544886 | 25000643 | 27224530 | 28422217 | 29270436 | 29590477 |
| 19441885 | 20646350 | 22075651 | 22779560 | 23545234 | 25000995 | 27225360 | 28422579 | 29270452 | 29592790 |
| 19442574 | 20646919 | 22075798 | 22779748 | 23545297 | 25001565 | 27226483 | 28422651 | 29270462 | 29592868 |
| 19442755 | 20647737 | 22075837 | 22779800 | 23545447 | 25003789 | 27226786 | 28423022 | 29270480 | 29593198 |
| 19443527 | 20648703 | 22075931 | 22780069 | 23546216 | 25004428 | 27226971 | 28423686 | 29270497 | 29593586 |
| 19443713 | 20649306 | 22076043 | 22780167 | 23546406 | 25004678 | 27227629 | 28423857 | 29270498 | 29593868 |
| 19444140 | 20649394 | 22076337 | 22780387 | 23546820 | 25005118 | 27227730 | 28424112 | 29270518 | 29593939 |
| 19444368 | 20649672 | 22076672 | 22780465 | 23547928 | 25005916 | 27229550 | 28424339 | 29270526 | 29594354 |
| 19445099 | 20650541 | 22076724 | 22781014 | 23548544 | 25006362 | 27229914 | 28424382 | 29270530 | 29595767 |
| 19445409 | 20650627 | 22076990 | 22781052 | 23549172 | 25006465 | 27230260 | 28424613 | 29270536 | 29597142 |
| 19445464 | 20651863 | 22077698 | 22781077 | 23549220 | 25006701 | 27230312 | 28424860 | 29270542 | 29598279 |
| 19445662 | 20652286 | 22077751 | 22781292 | 23549466 | 25006968 | 27230903 | 28425395 | 29270549 | 29598621 |
| 19445820 | 20652790 | 22078563 | 22781334 | 23549554 | 25007621 | 27231004 | 28425669 | 29270550 | 29598925 |
| 19446760 | 20654470 | 22078655 | 22781714 | 23550208 | 25009505 | 27231068 | 28425682 | 29270555 | 29599420 |
| 19446850 | 20655662 | 22078755 | 22781789 | 23551254 | 25009882 | 27231169 | 28426076 | 29270556 | 29599496 |
| 19446878 | 20655812 | 22078857 | 22782247 | 23552060 | 25011251 | 27231290 | 28426479 | 29270557 | 29599651 |
| 19447007 | 20656271 | 22079334 | 22782435 | 23552423 | 25011428 | 27231405 | 28426698 | 29270560 | 29599712 |
| 19447098 | 20657226 | 22079527 | 22782479 | 23552444 | 25011903 | 27231775 | 28426879 | 29270565 | 29599729 |
| 19447584 | 20657766 | 22079588 | 22782497 | 23552487 | 25012799 | 27231816 | 28426920 | 29270573 | 29600448 |
| 19448603 | 20658272 | 22080129 | 22782593 | 23552727 | 25013332 | 27232637 | 28426950 | 29270580 | 29601615 |
| 19449263 | 20658406 | 22080169 | 22782605 | 23553110 | 25015861 | 27232652 | 28427066 | 29270602 | 29602872 |
| 19449934 | 20658511 | 22080340 | 22782767 | 23553755 | 25016192 | 27232686 | 28428098 | 29270619 | 29604393 |
| 19450076 | 20659300 | 22080386 | 22782780 | 23553802 | 25017611 | 27234683 | 28428512 | 29270622 | 29604413 |
| 19450563 | 20660295 | 22080451 | 22782783 | 23553840 | 25018588 | 27235059 | 28428577 | 29270639 | 29604506 |
| 19451122 | 20662705 | 22080555 | 22782925 | 23554817 | 25020395 | 27235089 | 28429515 | 29270648 | 29604946 |
| 19451368 | 20663373 | 22080958 | 22783034 | 23556737 | 25020675 | 27235496 | 28429706 | 29270668 | 29605391 |
| 19451450 | 20663495 | 22081075 | 22783172 | 23556813 | 25021376 | 27235616 | 28430014 | 29270669 | 29605664 |
| 19451465 | 20663587 | 22081339 | 22783271 | 23556915 | 25023104 | 27235814 | 28430686 | 29270684 | 29606038 |
| 19451809 | 20663909 | 22081579 | 22783569 | 23558057 | 25023653 | 27236113 | 28430712 | 29270692 | 29606440 |
| 19452100 | 20664148 | 22081852 | 22784096 | 23558320 | 25024046 | 27236125 | 28431270 | 29270715 | 29606772 |
| 19452301 | 20666875 | 22082026 | 22784265 | 23558588 | 25024231 | 27236155 | 28431819 | 29270726 | 29607112 |
| 19452320 | 20666970 | 22082133 | 22784783 | 23559060 | 25024797 | 27236566 | 28432131 | 29270763 | 29607572 |
| 19452473 | 20667043 | 22082140 | 22784796 | 23559195 | 25028190 | 27236586 | 28432308 | 29270764 | 29608235 |
| 19452528 | 20667833 | 22083204 | 22785001 | 23559221 | 25028245 | 27236806 | 28432725 | 29270772 | 29609773 |
| 19452667 | 20668954 | 22083453 | 22785345 | 23559384 | 25029116 | 27237309 | 28432763 | 29270778 | 29610157 |
| 19452680 | 20669026 | 22083998 | 22785370 | 23559808 | 25029931 | 27237377 | 28432888 | 29270783 | 29610432 |
| 19452911 | 20669281 | 22084771 | 22785382 | 23560197 | 25030043 | 27237718 | 28433153 | 29270788 | 29610865 |
| 19453817 | 20669723 | 22085099 | 22785655 | 23560231 | 25030641 | 27237896 | 28433376 | 29270799 | 29612183 |
| 19453936 | 20670055 | 22085144 | 22785715 | 23560322 | 25030751 | 27238318 | 28434325 | 29270806 | 29612858 |
| 19454050 | 20670596 | 22085928 | 22785727 | 23560385 | 25031225 | 27238320 | 28434649 | 29270834 | 29613929 |
| 19454146 | 20670754 | 22086151 | 22785878 | 23560580 | 25033905 | 27238403 | 28435204 | 29270842 | 29613959 |
| 19454516 | 20671885 | 22086400 | 22786406 | 23560999 | 25035123 | 27238593 | 28435716 | 29270858 | 29614519 |
| 19455085 | 20672395 | 22086551 | 22787207 | 23561148 | 25035175 | 27238850 | 28435887 | 29270861 | 29614757 |
| 19455129 | 20675945 | 22086889 | 22787392 | 23561152 | 25035865 | 27239744 | 28435934 | 29270868 | 29616532 |
| 19455818 | 20675981 | 22086989 | 22787433 | 23561387 | 25036975 | 27240323 | 28436008 | 29270872 | 29616808 |
| 19455986 | 20676480 | 22087176 | 22787496 | 23561426 | 25037411 | 27240504 | 28436243 | 29270881 | 29616843 |
| 19457581 | 20676825 | 22087371 | 22787629 | 23561624 | 25037451 | 27241512 | 28436259 | 29270889 | 29616861 |
| 19457662 | 20677005 | 22087399 | 22787754 | 23562031 | 25037580 | 27241666 | 28437512 | 29270890 | 29617591 |
| 19457867 | 20677482 | 22087414 | 22788128 | 23562272 | 25037681 | 27241731 | 28437973 | 29270897 | 29618484 |
| 19457917 | 20678003 | 22087490 | 22788220 | 23562617 | 25038372 | 27242094 | 28438500 | 29270921 | 29618489 |
| 19458684 | 20678547 | 22087493 | 22788310 | 23562627 | 25038601 | 27242214 | 28438898 | 29270932 | 29618588 |
| 19458701 | 20679023 | 22087583 | 22788657 | 23562940 | 25039309 | 27242827 | 28439034 | 29270933 | 29620302 |
| 19458923 | 20679407 | 22087904 | 22788685 | 23562993 | 25039767 | 27242982 | 28439070 | 29270935 | 29620764 |
| 19459320 | 20679665 | 22088163 | 22788810 | 23563012 | 25039876 | 27243088 | 28439237 | 29270938 | 29621131 |
| 19459565 | 20680802 | 22088343 | 22789194 | 23563080 | 25040783 | 27243713 | 28439365 | 29270952 | 29621703 |
| 19460806 | 20682389 | 22088427 | 22789393 | 23564174 | 25041242 | 27244536 | 28439863 | 29270963 | 29622149 |
| 19460907 | 20683331 | 22088480 | 22789562 | 23564552 | 25043574 | 27244962 | 28440506 | 29271000 | 29622178 |
| 19461100 | 20684951 | 22088788 | 22790075 | 23564604 | 25044034 | 27245083 | 28440770 | 29271009 | 29622895 |
| 19461252 | 20686143 | 22088809 | 22790109 | 23564725 | 25044064 | 27245226 | 28440779 | 29271016 | 29623326 |
| 19461913 | 20686430 | 22089081 | 22790333 | 23564896 | 25045513 | 27245967 | 28441812 | 29271017 | 29624026 |
| 19462362 | 20686864 | 22089171 | 22790420 | 23565012 | 25045590 | 27247214 | 28441982 | 29271019 | 29624322 |
| 19462437 | 20687582 | 22089323 | 22790657 | 23565209 | 25048342 | 27247300 | 28442115 | 29271032 | 29624355 |
| 19463115 | 20688161 | 22090410 | 22791051 | 23566276 | 25050685 | 27247593 | 28442243 | 29271033 | 29624389 |
| 19463427 | 20688326 | 22090482 | 22791539 | 23567495 | 25055080 | 27247665 | 28443126 | 29271042 | 29624684 |
| 19463947 | 20688593 | 22090531 | 22791706 | 23568310 | 25055851 | 27247774 | 28443198 | 29271058 | 29625091 |
| 19464684 | 20689237 | 22091406 | 22791996 | 23568695 | 25056200 | 27247881 | 28443486 | 29271059 | 29625176 |
| 19464825 | 20690074 | 22091894 | 22792236 | 23569125 | 25056208 | 27248930 | 28443647 | 29271062 | 29625505 |
| 19464854 | 20690220 | 22091929 | 22792490 | 23570240 | 25057957 | 27248979 | 28443902 | 29271063 | 29625514 |
| 19465135 | 20690950 | 22092015 | 22793079 | 23570451 | 25059856 | 27249413 | 28444119 | 29271064 | 29625520 |
| 19465185 | 20690965 | 22092084 | 22793098 | 23570995 | 25059868 | 27249494 | 28444180 | 29271091 | 29626241 |
| 19465231 | 20691348 | 22092098 | 22793249 | 23571123 | 25061237 | 27249657 | 28445156 | 29271110 | 29627265 |
| 19465251 | 20692668 | 22092452 | 22794732 | 23572443 | 25063246 | 27249965 | 28445349 | 29271120 | 29627747 |
| 19465335 | 20692822 | 22092696 | 22795043 | 23573184 | 25063860 | 27250370 | 28445444 | 29271126 | 29627775 |
| 19465903 | 20693115 | 22093075 | 22795232 | 23573500 | 25064024 | 27250811 | 28445658 | 29271132 | 29627930 |
| 19466589 | 20693589 | 22093848 | 22795577 | 23574226 | 25064036 | 27250848 | 28445805 | 29271134 | 29629499 |
| 19466709 | 20693704 | 22095713 | 22796020 | 23574299 | 25064572 | 27251136 | 28446188 | 29271151 | 29629530 |
| 19466802 | 20693854 | 22095880 | 22796132 | 23575308 | 25066521 | 27251286 | 28446670 | 29271152 | 29631244 |
| 19467186 | 20693897 | 22096015 | 22796386 | 23575704 | 25066819 | 27251362 | 28446812 | 29271160 | 29631551 |
| 19467335 | 20694780 | 22096218 | 22796665 | 23575906 | 25067291 | 27251504 | 28446915 | 29271179 | 29631557 |
| 19468026 | 20695241 | 22096453 | 22796791 | 23576113 | 25069058 | 27251831 | 28447010 | 29271180 | 29632819 |
| 19468327 | 20696309 | 22096515 | 22796917 | 23576189 | 25069350 | 27251849 | 28447517 | 29271186 | 29632977 |
| 19468434 | 20696743 | 22096793 | 22796931 | 23576714 | 25069461 | 27252163 | 28447877 | 29271199 | 29633341 |
| 19469247 | 20697869 | 22097006 | 22797136 | 23577574 | 25070231 | 27252395 | 28448177 | 29271200 | 29633539 |
| 19469375 | 20698470 | 22097499 | 22797464 | 23578127 | 25071540 | 27252873 | 28448390 | 29271203 | 29633970 |
| 19469823 | 20699102 | 22097560 | 22797654 | 23578158 | 25072135 | 27253167 | 28449496 | 29271208 | 29634025 |
| 19470159 | 20700132 | 22097598 | 22797715 | 23578249 | 25073179 | 27253279 | 28449989 | 29271237 | 29636123 |
| 19470399 | 20702252 | 22098176 | 22797808 | 23579276 | 25073276 | 27253743 | 28450022 | 29271238 | 29636968 |
| 19470749 | 20703373 | 22098718 | 22797944 | 23579438 | 25074002 | 27255310 | 28450359 | 29271263 | 29636972 |
| 19470803 | 20704384 | 22098971 | 22798103 | 23579441 | 25074623 | 27255601 | 28450605 | 29271270 | 29637484 |
| 19470982 | 20705689 | 22099085 | 22798315 | 23579509 | 25074631 | 27255689 | 28450608 | 29271273 | 29637677 |
| 19471457 | 20706440 | 22099712 | 22798459 | 23579738 | 25074961 | 27257674 | 28450688 | 29271286 | 29637842 |
| 19471667 | 20706493 | 22100195 | 22798547 | 23579774 | 25075652 | 27258032 | 28451415 | 29271289 | 29638117 |
| 19472010 | 20706526 | 22100242 | 22798573 | 23580002 | 25076140 | 27258235 | 28451457 | 29271295 | 29638218 |
| 19472101 | 20706588 | 22100278 | 22798581 | 23580395 | 25076150 | 27259290 | 28452142 | 29271297 | 29638928 |
| 19472250 | 20707849 | 22100417 | 22798779 | 23580472 | 25076530 | 27259365 | 28452612 | 29271303 | 29639975 |
| 19472552 | 20707979 | 22100452 | 22798839 | 23581079 | 25076947 | 27259572 | 28452770 | 29271320 | 29640203 |
| 19473452 | 20708257 | 22100561 | 22798877 | 23581474 | 25077671 | 27259773 | 28453680 | 29271330 | 29640356 |
| 19473753 | 20708498 | 22100851 | 22798905 | 23582152 | 25077909 | 27261495 | 28453749 | 29271364 | 29640533 |
| 19474398 | 20708819 | 22100938 | 22799092 | 23583768 | 25078087 | 27261717 | 28453827 | 29271369 | 29641307 |
| 19474610 | 20710238 | 22101008 | 22799733 | 23583877 | 25078102 | 27262668 | 28453961 | 29271376 | 29641331 |
| 19474696 | 20710909 | 22101913 | 22800867 | 23584027 | 25079157 | 27262857 | 28454944 | 29271379 | 29643124 |
| 19474941 | 20711250 | 22101923 | 22801090 | 23584156 | 25081199 | 27263526 | 28455350 | 29271395 | 29643285 |
| 19477147 | 20711296 | 22102118 | 22801120 | 23584866 | 25081273 | 27263766 | 28455686 | 29271398 | 29644060 |
| 19477436 | 20711432 | 22102762 | 22801483 | 23585182 | 25082296 | 27263991 | 28455895 | 29271404 | 29645552 |
| 19477524 | 20713682 | 22103109 | 22801557 | 23585512 | 25082432 | 27264193 | 28456600 | 29271418 | 29645661 |
| 19477562 | 20714345 | 22103160 | 22801978 | 23585881 | 25082634 | 27265702 | 28457066 | 29271422 | 29645786 |
| 19478113 | 20715015 | 22103322 | 22802163 | 23586378 | 25084002 | 27265895 | 28457228 | 29271426 | 29646164 |
| 19478197 | 20715427 | 22103452 | 22802212 | 23586661 | 25084280 | 27266397 | 28457465 | 29271433 | 29646331 |
| 19478538 | 20715562 | 22103527 | 22802216 | 23586796 | 25084593 | 27266590 | 28457728 | 29271444 | 29646532 |
| 19478616 | 20716111 | 22103566 | 22802888 | 23587650 | 25086177 | 27266884 | 28458085 | 29271449 | 29647224 |
| 19479072 | 20717095 | 22103638 | 22802936 | 23587678 | 25086494 | 27267501 | 28458432 | 29271453 | 29648021 |
| 19479701 | 20717151 | 22104231 | 22803585 | 23587979 | 25086883 | 27268346 | 28461130 | 29271456 | 29648615 |
| 19479831 | 20718600 | 22104704 | 22803671 | 23588104 | 25087867 | 27268482 | 28461131 | 29271471 | 29649485 |
| 19480378 | 20720269 | 22104807 | 22804088 | 23588211 | 25088003 | 27268784 | 28461574 | 29271481 | 29649663 |
| 19481245 | 20720425 | 22104817 | 22804096 | 23588411 | 25089995 | 27269526 | 28461942 | 29271486 | 29650185 |
| 19481471 | 20720973 | 22105608 | 22804771 | 23588793 | 25092445 | 27269783 | 28462326 | 29271491 | 29650290 |
| 19482065 | 20721039 | 22105623 | 22805009 | 23588874 | 25092935 | 27269817 | 28463038 | 29271496 | 29650535 |
| 19482387 | 20722038 | 22105640 | 22805658 | 23589238 | 25093809 | 27270011 | 28463371 | 29271499 | 29651475 |
| 19482573 | 20722491 | 22105670 | 22805878 | 23589362 | 25094303 | 27270057 | 28463392 | 29271502 | 29651748 |
| 19482616 | 20722531 | 22106136 | 22806114 | 23589442 | 25095100 | 27270549 | 28463514 | 29271510 | 29651830 |
| 19482964 | 20723115 | 22106537 | 22806273 | 23590150 | 25095358 | 27270681 | 28463794 | 29271517 | 29651917 |
| 19483036 | 20723681 | 22106933 | 22806511 | 23590754 | 25096129 | 27270835 | 28463953 | 29271531 | 29652142 |
| 19483419 | 20723807 | 22107099 | 22806734 | 23591202 | 25096224 | 27271426 | 28464036 | 29271538 | 29652357 |
| 19483644 | 20724531 | 22107324 | 22806844 | 23591329 | 25096300 | 27272456 | 28464125 | 29271560 | 29652728 |
| 19484202 | 20725415 | 22107614 | 22806905 | 23591473 | 25096757 | 27272492 | 28464350 | 29271566 | 29652950 |
| 19484281 | 20725599 | 22107626 | 22807008 | 23591529 | 25100318 | 27272659 | 28464396 | 29271584 | 29653232 |
| 19484723 | 20726279 | 22107929 | 22807141 | 23591560 | 25100895 | 27272993 | 28465590 | 29271591 | 29653274 |
| 19485031 | 20726292 | 22108011 | 22807379 | 23591968 | 25102254 | 27273092 | 28465595 | 29271592 | 29654094 |
| 19485135 | 20726592 | 22108257 | 22807592 | 23592375 | 25102862 | 27273372 | 28466006 | 29271593 | 29654209 |
| 19485774 | 20726671 | 22109556 | 22807690 | 23592877 | 25104186 | 27274203 | 28466251 | 29271594 | 29654975 |
| 19486262 | 20727309 | 22109688 | 22807833 | 23593008 | 25104917 | 27274906 | 28466368 | 29271595 | 29656514 |
| 19486697 | 20727715 | 22109830 | 22808242 | 23593265 | 25105659 | 27275137 | 28467132 | 29271596 | 29656679 |
| 19487215 | 20728766 | 22110011 | 22808342 | 23593473 | 25105692 | 27275209 | 28467352 | 29271598 | 29657531 |
| 19487334 | 20730400 | 22110383 | 22808684 | 23593837 | 25106458 | 27275381 | 28467571 | 29271603 | 29658002 |
| 19487533 | 20730633 | 22111201 | 22808765 | 23594199 | 25106577 | 27275465 | 28468148 | 29271609 | 29658258 |
| 19487898 | 20731417 | 22111390 | 22809183 | 23594267 | 25107670 | 27275716 | 28468586 | 29271617 | 29658264 |
| 19487940 | 20732131 | 22111559 | 22809411 | 23594748 | 25108394 | 27276088 | 28469067 | 29271619 | 29659915 |
| 19487985 | 20733150 | 22111920 | 22810292 | 23594926 | 25108456 | 27276222 | 28469207 | 29271638 | 29660101 |
| 19488545 | 20733210 | 22112051 | 22810346 | 23595619 | 25108913 | 27276874 | 28469258 | 29271651 | 29660358 |
| 19488669 | 20733334 | 22112093 | 22810397 | 23596181 | 25110530 | 27277704 | 28469710 | 29271653 | 29662045 |
| 19489136 | 20733484 | 22112270 | 22810965 | 23596182 | 25112570 | 27278086 | 28469834 | 29271681 | 29662146 |
| 19489902 | 20733532 | 22112587 | 22811227 | 23596295 | 25113179 | 27278201 | 28470665 | 29271696 | 29662149 |
| 19490540 | 20733913 | 22112625 | 22811422 | 23596343 | 25114100 | 27278264 | 28470805 | 29271700 | 29662801 |
| 19491047 | 20734108 | 22112880 | 22811525 | 23596410 | 25114568 | 27279165 | 28470853 | 29271705 | 29663324 |
| 19491099 | 20734265 | 22112883 | 22811708 | 23596583 | 25116293 | 27279869 | 28471002 | 29271706 | 29663690 |
| 19491636 | 20734660 | 22113029 | 22811817 | 23596997 | 25117556 | 27280195 | 28471410 | 29271722 | 29664250 |
| 19491746 | 20735625 | 22113173 | 22811842 | 23597382 | 25118326 | 27280277 | 28471955 | 29271731 | 29664300 |
| 19491752 | 20736213 | 22113536 | 22811866 | 23597504 | 25119111 | 27280637 | 28472515 | 29271742 | 29664398 |
| 19492308 | 20736903 | 22114545 | 22812077 | 23598133 | 25119247 | 27280638 | 28472625 | 29271748 | 29664467 |
| 19493278 | 20737114 | 22114713 | 22812427 | 23598684 | 25120595 | 27280726 | 28472920 | 29271756 | 29665358 |
| 19493878 | 20737512 | 22114930 | 22812725 | 23598978 | 25121552 | 27281336 | 28473547 | 29271758 | 29665805 |
| 19494202 | 20737866 | 22115007 | 22812812 | 23599098 | 25121737 | 27281372 | 28473634 | 29271778 | 29667097 |
| 19495165 | 20738103 | 22115148 | 22813085 | 23600494 | 25122281 | 27281727 | 28473680 | 29271779 | 29667240 |
| 19495211 | 20738289 | 22115691 | 22813142 | 23600924 | 25122961 | 27281745 | 28474092 | 29271799 | 29667309 |
| 19495294 | 20738365 | 22115848 | 22813160 | 23602361 | 25122973 | 27281768 | 28474159 | 29271814 | 29667693 |
| 19495326 | 20739802 | 22115878 | 22813234 | 23603107 | 25123311 | 27281880 | 28475450 | 29271819 | 29667799 |
| 19495340 | 20740378 | 22115917 | 22813757 | 23603416 | 25124183 | 27282358 | 28475603 | 29271840 | 29668702 |
| 19495677 | 20740506 | 22115929 | 22813817 | 23603447 | 25124934 | 27282747 | 28475953 | 29271848 | 29668729 |
| 19497245 | 20741112 | 22116057 | 22813998 | 23603699 | 25125576 | 27282845 | 28476355 | 29271853 | 29669613 |
| 19497664 | 20741317 | 22116072 | 22814042 | 23604062 | 25126699 | 27282889 | 28477202 | 29271857 | 29670736 |
| 19497854 | 20741963 | 22116213 | 22814236 | 23605415 | 25129409 | 27283385 | 28477590 | 29271861 | 29671110 |
| 19498358 | 20742059 | 22116262 | 22814416 | 23605616 | 25131181 | 27283434 | 28477705 | 29271865 | 29671701 |
| 19498563 | 20742267 | 22116568 | 22814762 | 23605882 | 25131754 | 27283753 | 28478219 | 29271887 | 29672061 |
| 19498830 | 20742779 | 22116651 | 22815092 | 23606244 | 25132400 | 27284382 | 28478383 | 29271898 | 29672162 |
| 19498998 | 20742966 | 22116835 | 22815371 | 23606332 | 25132853 | 27284460 | 28478901 | 29271899 | 29672718 |
| 19499400 | 20744000 | 22117034 | 22815439 | 23606771 | 25134165 | 27284462 | 28480308 | 29271914 | 29673104 |
| 19500230 | 20744424 | 22117121 | 22816070 | 23606888 | 25134198 | 27284912 | 28480520 | 29271920 | 29674590 |
| 19500815 | 20745104 | 22117282 | 22816397 | 23607719 | 25134498 | 27285093 | 28480579 | 29271928 | 29677716 |
| 19500831 | 20745819 | 22117437 | 22816535 | 23607847 | 25135121 | 27285698 | 28480596 | 29271929 | 29677756 |
| 19500893 | 20747054 | 22117450 | 22816784 | 23607976 | 25136393 | 27285733 | 28480729 | 29271934 | 29680076 |
| 19501227 | 20747135 | 22117474 | 22816789 | 23608028 | 25136976 | 27285751 | 28480826 | 29271944 | 29680279 |
| 19501504 | 20748207 | 22117942 | 22816885 | 23608557 | 25137457 | 27286270 | 28480835 | 29271980 | 29680340 |
| 19502389 | 20748228 | 22118090 | 22816942 | 23608732 | 25137461 | 27286272 | 28480878 | 29271984 | 29680472 |
| 19502735 | 20748939 | 22118196 | 22817227 | 23610021 | 25139724 | 27286451 | 28480883 | 29271992 | 29680827 |
| 19502782 | 20749256 | 22118782 | 22817280 | 23610609 | 25139963 | 27286456 | 28481154 | 29272000 | 29681126 |
| 19503181 | 20751257 | 22118862 | 22817434 | 23611043 | 25140825 | 27286725 | 28482072 | 29272005 | 29681478 |
| 19504211 | 20751384 | 22119102 | 22817924 | 23611633 | 25141457 | 27287951 | 28482230 | 29272010 | 29682190 |
| 19504278 | 20752204 | 22119281 | 22818046 | 23612222 | 25141503 | 27288386 | 28482414 | 29272019 | 29683060 |
| 19504314 | 20753496 | 22119292 | 22818147 | 23612425 | 25142480 | 27288797 | 28482659 | 29272041 | 29684166 |
| 19504576 | 20754168 | 22119393 | 22818481 | 23612760 | 25145441 | 27288984 | 28483409 | 29272045 | 29684350 |
| 19505169 | 20754506 | 22119635 | 22818649 | 23613975 | 25146289 | 27289633 | 28483895 | 29272050 | 29684573 |
| 19506027 | 20755110 | 22119687 | 22819066 | 23614364 | 25146473 | 27289828 | 28484564 | 29272051 | 29684694 |
| 19506318 | 20755677 | 22120411 | 22819206 | 23615200 | 25148621 | 27290201 | 28484655 | 29272058 | 29685050 |
| 19506659 | 20756196 | 22120652 | 22819491 | 23615417 | 25149441 | 27291386 | 28484844 | 29272078 | 29685217 |
| 19507570 | 20757981 | 22121001 | 22819855 | 23615941 | 25149698 | 27291538 | 28485958 | 29272112 | 29686491 |
| 19507632 | 20758406 | 22121229 | 22820721 | 23616001 | 25150878 | 27291717 | 28486868 | 29272117 | 29686631 |
| 19507794 | 20758676 | 22121296 | 22821157 | 23616245 | 25154311 | 27291948 | 28487439 | 29272122 | 29686722 |
| 19507827 | 20758901 | 22121470 | 22821497 | 23616255 | 25154475 | 27292602 | 28487605 | 29272130 | 29687710 |
| 19507934 | 20760221 | 22121881 | 22821515 | 23616293 | 25155036 | 27292677 | 28487888 | 29272133 | 29687774 |
| 19508904 | 20760687 | 22122095 | 22821552 | 23616726 | 25155141 | 27293256 | 28487910 | 29272134 | 29688141 |
| 19509221 | 20761267 | 22122496 | 22821670 | 23617261 | 25155624 | 27295063 | 28488495 | 29272146 | 29688722 |
| 19509573 | 20761285 | 22122528 | 22821751 | 23617289 | 25156249 | 27295455 | 28488628 | 29272153 | 29689049 |
| 19509732 | 20761563 | 22122828 | 22822458 | 23617652 | 25156684 | 27296690 | 28488817 | 29272162 | 29689931 |
| 19509839 | 20762319 | 22122927 | 22822518 | 23617676 | 25156803 | 27297124 | 28489081 | 29272169 | 29690257 |
| 19510051 | 20762362 | 22123099 | 22822991 | 23618161 | 25156915 | 27297508 | 28489674 | 29272172 | 29690330 |
| 19510166 | 20762780 | 22123211 | 22823435 | 23618406 | 25158249 | 27297783 | 28489800 | 29272173 | 29690857 |
| 19510775 | 20762995 | 22123609 | 22823735 | 23618928 | 25159661 | 27297879 | 28489893 | 29272188 | 29690989 |
| 19510874 | 20763825 | 22123699 | 22823864 | 23619243 | 25159705 | 27298201 | 28490043 | 29272194 | 29691324 |
| 19510909 | 20764320 | 22123750 | 22824227 | 23619373 | 25161254 | 27299480 | 28490479 | 29272203 | 29691564 |
| 19511620 | 20764329 | 22123790 | 22824676 | 23619516 | 25161823 | 27299715 | 28490783 | 29272206 | 29691695 |
| 19512938 | 20765500 | 22124411 | 22825281 | 23620135 | 25161885 | 27299732 | 28491212 | 29272208 | 29691797 |
| 19513056 | 20766769 | 22124592 | 22825737 | 23620283 | 25162216 | 27299911 | 28491713 | 29272211 | 29691861 |
| 19513196 | 20767154 | 22124918 | 22825992 | 23620405 | 25163974 | 27299915 | 28491746 | 29272214 | 29691947 |
| 19513461 | 20767457 | 22125259 | 22826033 | 23620466 | 25165652 | 27299924 | 28491829 | 29272218 | 29692126 |
| 19513628 | 20768130 | 22125547 | 22826367 | 23620528 | 25166349 | 27299949 | 28491939 | 29272220 | 29692147 |
| 19514173 | 20768564 | 22125677 | 22826388 | 23620677 | 25167355 | 27300196 | 28492258 | 29272237 | 29693242 |
| 19514208 | 20769607 | 22125870 | 22826684 | 23621200 | 25167655 | 27300475 | 28492331 | 29272249 | 29693619 |
| 19514501 | 20770072 | 22126583 | 22826705 | 23621254 | 25167686 | 27300817 | 28492370 | 29272252 | 29694567 |
| 19514681 | 20770117 | 22126801 | 22827069 | 23622656 | 25169878 | 27302217 | 28492511 | 29272266 | 29695420 |
| 19516029 | 20770526 | 22126894 | 22827420 | 23623777 | 25170468 | 27302549 | 28492937 | 29272282 | 29696970 |
| 19516052 | 20770659 | 22126899 | 22827964 | 23623979 | 25170559 | 27302804 | 28493108 | 29272290 | 29697117 |
| 19516297 | 20771967 | 22126908 | 22828296 | 23624079 | 25170935 | 27302853 | 28493693 | 29272295 | 29698121 |
| 19516305 | 20771987 | 22127381 | 22828522 | 23624993 | 25173153 | 27303013 | 28494103 | 29272297 | 29698573 |
| 19516546 | 20773398 | 22127733 | 22828986 | 23624996 | 25173333 | 27303090 | 28495626 | 29272300 | 29698722 |
| 19516563 | 20773898 | 22127765 | 22829146 | 23625368 | 25173872 | 27303541 | 28496043 | 29272305 | 29698820 |
| 19516869 | 20774333 | 22127991 | 22829197 | 23625685 | 25175038 | 27305070 | 28496064 | 29272310 | 29699226 |
| 19516937 | 20774625 | 22128084 | 22829204 | 23625951 | 25175524 | 27305283 | 28496322 | 29272314 | 29699744 |
| 19517306 | 20775773 | 22128191 | 22829320 | 23626484 | 25176190 | 27306411 | 28496475 | 29272320 | 29700051 |
| 19517327 | 20776456 | 22128240 | 22829526 | 23626718 | 25176370 | 27306592 | 28498575 | 29272324 | 29700588 |
| 19517329 | 20777880 | 22128348 | 22829615 | 23626726 | 25177566 | 27306605 | 28499178 | 29272325 | 29700894 |
| 19517429 | 20777996 | 22128353 | 22830303 | 23626801 | 25179510 | 27307132 | 28499253 | 29272341 | 29701180 |
| 19517527 | 20778708 | 22128459 | 22830740 | 23627058 | 25183170 | 27307678 | 28499436 | 29272360 | 29701213 |
| 19518407 | 20778886 | 22129079 | 22830939 | 23627151 | 25183189 | 27307772 | 28499666 | 29272371 | 29702921 |
| 19518637 | 20779579 | 22129422 | 22831062 | 23627549 | 25184253 | 27307930 | 28500476 | 29272376 | 29702942 |
| 19518747 | 20779860 | 22129447 | 22831161 | 23627568 | 25184627 | 27308282 | 28500536 | 29272385 | 29702952 |
| 19519310 | 20779919 | 22130154 | 22831277 | 23627586 | 25185936 | 27308640 | 28501337 | 29272395 | 29703363 |
| 19520949 | 20780575 | 22130232 | 22831399 | 23627590 | 25188807 | 27310093 | 28501716 | 29272417 | 29703755 |
| 19520982 | 20780877 | 22130311 | 22831847 | 23627864 | 25190180 | 27310208 | 28501919 | 29272418 | 29703846 |
| 19521463 | 20781649 | 22130646 | 22832429 | 23627901 | 25190210 | 27311308 | 28503302 | 29272423 | 29704528 |
| 19521626 | 20781763 | 22130665 | 22832809 | 23628347 | 25190301 | 27311347 | 28503347 | 29272430 | 29704609 |
| 19521914 | 20782429 | 22130872 | 22832914 | 23628693 | 25191205 | 27311622 | 28503390 | 29272435 | 29704869 |
| 19522096 | 20783877 | 22131105 | 22833106 | 23629084 | 25192732 | 27312876 | 28504285 | 29272443 | 29705322 |
| 19522335 | 20784005 | 22131573 | 22833289 | 23629088 | 25193139 | 27312915 | 28504534 | 29272446 | 29705797 |
| 19522389 | 20784093 | 22131912 | 22833292 | 23629215 | 25193279 | 27313626 | 28504561 | 29272461 | 29705890 |
| 19522646 | 20784916 | 22131944 | 22833806 | 23629298 | 25193333 | 27314214 | 28504786 | 29272465 | 29706558 |
| 19524454 | 20785465 | 22131998 | 22833916 | 23629334 | 25194443 | 27314257 | 28504885 | 29272473 | 29706599 |
| 19524596 | 20785695 | 22132055 | 22833934 | 23629524 | 25194551 | 27314365 | 28504909 | 29272482 | 29706608 |
| 19524779 | 20786222 | 22132160 | 22834602 | 23629554 | 25195659 | 27314632 | 28505322 | 29272484 | 29706740 |
| 19524837 | 20786720 | 22132209 | 22834619 | 23629617 | 25196058 | 27314991 | 28505680 | 29272496 | 29707054 |
| 19525028 | 20787773 | 22132873 | 22835138 | 23629942 | 25196599 | 27315165 | 28505932 | 29272500 | 29707231 |
| 19525230 | 20788233 | 22132928 | 22835505 | 23630053 | 25197628 | 27315298 | 28505990 | 29272523 | 29707311 |
| 19525604 | 20788308 | 22133006 | 22835750 | 23630236 | 25199319 | 27315307 | 28506232 | 29272540 | 29708126 |
| 19526029 | 20788401 | 22133085 | 22835776 | 23631594 | 25199579 | 27315673 | 28507034 | 29272550 | 29709780 |
| 19526041 | 20790003 | 22133129 | 22835902 | 23632261 | 25201668 | 27316054 | 28507472 | 29272551 | 29710256 |
| 19526109 | 20790232 | 22133146 | 22836446 | 23632479 | 25201751 | 27316247 | 28507625 | 29272560 | 29710695 |
| 19526349 | 20790270 | 22133482 | 22836649 | 23632612 | 25202117 | 27316321 | 28507811 | 29272563 | 29710959 |
| 19526549 | 20790332 | 22133636 | 22836779 | 23632928 | 25202477 | 27316346 | 28507825 | 29272572 | 29711050 |
| 19526619 | 20791331 | 22133637 | 22836884 | 23633190 | 25205247 | 27316428 | 28509198 | 29272584 | 29711141 |
| 19526939 | 20792054 | 22133814 | 22837008 | 23635095 | 25205586 | 27317009 | 28509721 | 29272587 | 29711281 |
| 19527065 | 20793077 | 22134129 | 22837103 | 23635414 | 25205737 | 27317255 | 28509760 | 29272588 | 29712532 |
| 19527613 | 20793401 | 22134144 | 22837434 | 23635733 | 25205860 | 27317725 | 28510125 | 29272589 | 29712590 |
| 19528076 | 20795282 | 22134410 | 22837538 | 23635920 | 25206282 | 27318102 | 28510312 | 29272593 | 29712730 |
| 19528603 | 20795550 | 22134524 | 22837643 | 23635963 | 25207280 | 27318663 | 28510391 | 29272595 | 29712854 |
| 19528896 | 20795560 | 22134610 | 22837885 | 23635992 | 25209860 | 27318732 | 28511173 | 29272609 | 29712938 |
| 19529430 | 20795918 | 22134778 | 22838055 | 23636040 | 25212079 | 27319084 | 28512093 | 29272627 | 29713289 |
| 19529500 | 20795938 | 22135286 | 22838293 | 23636162 | 25213981 | 27321048 | 28512490 | 29272643 | 29714792 |
| 19529531 | 20796848 | 22135322 | 22838420 | 23636195 | 25214030 | 27323167 | 28512930 | 29272646 | 29715322 |
| 19529839 | 20797137 | 22135372 | 22838517 | 23638088 | 25214277 | 27324081 | 28512953 | 29272649 | 29716233 |
| 19529898 | 20797492 | 22135449 | 22839515 | 23638096 | 25215791 | 27324117 | 28513018 | 29272654 | 29716850 |
| 19529996 | 20797936 | 22135501 | 22839764 | 23638901 | 25216145 | 27324704 | 28513098 | 29272655 | 29716894 |
| 19530541 | 20798435 | 22136247 | 22839958 | 23638990 | 25216199 | 27324899 | 28513307 | 29272657 | 29717264 |
| 19530861 | 20798801 | 22136896 | 22839981 | 23639559 | 25216619 | 27325177 | 28513744 | 29272658 | 29717894 |
| 19530871 | 20800157 | 22136899 | 22840035 | 23639624 | 25219745 | 27325896 | 28513873 | 29272659 | 29718214 |
| 19531648 | 20800432 | 22136984 | 22840120 | 23639684 | 25219752 | 27326374 | 28514115 | 29272671 | 29718230 |
| 19531871 | 20800641 | 22137131 | 22840715 | 23639919 | 25221526 | 27326698 | 28514677 | 29272672 | 29718585 |
| 19531995 | 20801235 | 22137162 | 22840774 | 23640226 | 25224412 | 27327572 | 28514732 | 29272673 | 29718877 |
| 19532007 | 20801582 | 22137312 | 22840783 | 23641299 | 25224952 | 27327760 | 28515722 | 29272676 | 29719716 |
| 19532016 | 20802679 | 22137341 | 22841306 | 23641551 | 25225397 | 27327989 | 28516010 | 29272685 | 29719919 |
| 19532060 | 20803554 | 22137811 | 22841482 | 23641556 | 25225632 | 27328083 | 28516255 | 29272688 | 29720025 |
| 19532381 | 20804576 | 22138374 | 22841604 | 23641825 | 25226102 | 27328206 | 28516611 | 29272716 | 29720215 |
| 19532504 | 20804868 | 22138542 | 22841673 | 23641950 | 25227340 | 27328545 | 28516799 | 29272729 | 29720334 |
| 19532735 | 20805407 | 22138630 | 22841737 | 23642048 | 25228334 | 27328961 | 28517020 | 29272730 | 29720628 |
| 19532838 | 20805690 | 22138881 | 22841881 | 23642312 | 25228895 | 27329939 | 28517145 | 29272749 | 29720650 |
| 19534285 | 20805936 | 22139398 | 22841882 | 23642431 | 25231465 | 27331296 | 28518447 | 29272750 | 29720675 |
| 19534356 | 20807139 | 22139623 | 22841905 | 23642444 | 25233498 | 27331845 | 28518554 | 29272754 | 29720750 |
| 19534362 | 20807399 | 22139674 | 22842094 | 23642581 | 25233814 | 27332303 | 28518897 | 29272779 | 29721129 |
| 19534550 | 20808606 | 22139830 | 22842342 | 23642619 | 25234001 | 27332799 | 28519513 | 29272781 | 29721557 |
| 19534711 | 20810931 | 22140130 | 22842345 | 23642666 | 25234057 | 27333927 | 28520050 | 29272786 | 29721568 |
| 19534767 | 20811153 | 22141834 | 22842451 | 23642882 | 25235402 | 27334059 | 28520057 | 29272820 | 29722871 |
| 19534858 | 20811797 | 22141886 | 22842591 | 23643000 | 25236893 | 27334674 | 28520207 | 29272821 | 29722880 |
| 19535063 | 20812565 | 22142095 | 22842615 | 23643187 | 25239611 | 27334912 | 28520926 | 29272829 | 29723051 |
| 19535570 | 20814269 | 22142440 | 22842678 | 23643606 | 25239709 | 27335241 | 28521476 | 29272837 | 29723236 |
| 19535610 | 20814372 | 22142469 | 22842733 | 23643696 | 25240612 | 27335505 | 28522277 | 29272846 | 29723366 |
| 19535660 | 20814907 | 22142896 | 22842984 | 23644165 | 25242170 | 27335549 | 28522621 | 29272852 | 29723415 |
| 19535735 | 20814996 | 22143246 | 22843292 | 23645568 | 25242456 | 27336171 | 28522671 | 29272853 | 29723431 |
| 19535775 | 20815900 | 22143285 | 22843776 | 23645626 | 25242885 | 27336270 | 28523467 | 29272860 | 29723671 |
| 19535836 | 20815993 | 22143419 | 22843853 | 23646518 | 25244535 | 27337857 | 28523581 | 29272877 | 29724625 |
| 19535893 | 20816284 | 22143486 | 22843932 | 23646609 | 25244813 | 27338221 | 28524516 | 29272892 | 29725389 |
| 19536082 | 20816833 | 22143558 | 22844009 | 23646759 | 25246150 | 27338900 | 28526855 | 29272895 | 29725645 |
| 19536702 | 20816848 | 22143980 | 22844257 | 23646966 | 25246919 | 27338975 | 28526883 | 29272897 | 29725746 |
| 19536965 | 20816987 | 22144328 | 22844943 | 23646989 | 25247927 | 27339137 | 28526906 | 29272912 | 29726475 |
| 19537117 | 20816998 | 22144456 | 22845302 | 23647251 | 25248072 | 27339139 | 28527242 | 29272918 | 29726902 |
| 19537478 | 20817348 | 22144592 | 22845459 | 23647305 | 25248340 | 27339145 | 28527243 | 29272924 | 29727162 |
| 19537553 | 20817676 | 22144729 | 22846806 | 23647406 | 25248610 | 27339169 | 28527424 | 29272936 | 29727364 |
| 19537582 | 20818493 | 22144873 | 22846817 | 23647969 | 25251458 | 27339516 | 28527493 | 29272938 | 29727716 |
| 19537793 | 20818691 | 22144899 | 22847240 | 23648034 | 25254387 | 27339518 | 28527828 | 29272939 | 29727913 |
| 19538144 | 20819777 | 22145120 | 22847254 | 23648147 | 25254821 | 27339743 | 28528227 | 29272946 | 29728164 |
| 19538178 | 20819881 | 22145258 | 22847483 | 23648362 | 25255505 | 27339750 | 28528406 | 29272962 | 29728776 |
| 19538506 | 20819944 | 22145298 | 22847494 | 23648453 | 25256558 | 27340162 | 28528703 | 29272964 | 29729006 |
| 19538912 | 20820597 | 22145718 | 22847663 | 23648766 | 25256627 | 27340512 | 28529120 | 29272972 | 29729052 |
| 19539210 | 20821256 | 22145739 | 22848037 | 23648782 | 25256928 | 27340981 | 28529606 | 29272976 | 29730164 |
| 19539279 | 20821318 | 22146061 | 22848207 | 23648971 | 25257704 | 27341283 | 28530393 | 29273007 | 29730212 |
| 19539432 | 20821861 | 22146727 | 22848545 | 23649123 | 25259692 | 27341298 | 28530448 | 29273008 | 29730468 |
| 19539531 | 20822467 | 22146867 | 22848625 | 23650097 | 25260760 | 27341920 | 28530940 | 29273013 | 29730477 |
| 19539683 | 20822768 | 22147085 | 22848711 | 23650252 | 25261183 | 27341971 | 28531471 | 29273022 | 29730558 |
| 19539880 | 20823209 | 22147164 | 22848947 | 23650433 | 25264345 | 27343315 | 28531783 | 29273027 | 29731001 |
| 19540394 | 20824229 | 22147254 | 22849188 | 23650571 | 25266046 | 27343834 | 28531831 | 29273030 | 29731926 |
| 19540526 | 20824544 | 22147404 | 22849233 | 23651143 | 25267336 | 27344375 | 28531946 | 29273034 | 29732013 |
| 19541207 | 20824897 | 22147538 | 22849291 | 23651208 | 25267552 | 27345820 | 28532019 | 29273045 | 29732469 |
| 19541295 | 20825243 | 22147628 | 22849454 | 23651327 | 25267577 | 27345882 | 28532432 | 29273046 | 29732823 |
| 19541469 | 20825795 | 22148048 | 22849556 | 23651710 | 25268060 | 27346031 | 28532507 | 29273058 | 29733915 |
| 19541787 | 20826051 | 22148089 | 22850160 | 23651858 | 25270890 | 27346038 | 28533284 | 29273066 | 29734104 |
| 19542530 | 20827266 | 22148348 | 22850186 | 23651952 | 25271005 | 27346123 | 28534064 | 29273087 | 29734242 |
| 19542554 | 20828064 | 22148411 | 22850188 | 23652448 | 25274061 | 27346299 | 28534463 | 29273088 | 29734496 |
| 19542621 | 20828548 | 22148656 | 22850476 | 23652620 | 25274127 | 27348436 | 28534514 | 29273140 | 29734747 |
| 19543600 | 20828698 | 22148708 | 22850671 | 23652728 | 25274200 | 27348446 | 28534712 | 29273143 | 29735588 |
| 19543748 | 20829148 | 22148932 | 22850679 | 23652804 | 25274780 | 27348694 | 28535111 | 29273145 | 29735910 |
| 19543936 | 20829569 | 22148972 | 22850830 | 23653014 | 25275296 | 27348824 | 28535146 | 29273154 | 29736482 |
| 19544963 | 20829911 | 22149247 | 22850915 | 23654120 | 25275955 | 27349135 | 28535337 | 29273163 | 29737962 |
| 19544968 | 20830273 | 22149797 | 22851002 | 23654591 | 25276383 | 27349254 | 28535436 | 29273166 | 29738453 |
| 19545302 | 20830864 | 22149802 | 22851459 | 23654772 | 25278650 | 27350089 | 28535513 | 29273173 | 29739863 |
| 19545476 | 20831155 | 22149878 | 22851612 | 23655699 | 25278921 | 27350772 | 28535564 | 29273174 | 29740156 |
| 19545501 | 20833503 | 22150052 | 22852410 | 23656018 | 25280344 | 27351132 | 28536366 | 29273192 | 29740235 |
| 19545744 | 20834092 | 22150594 | 22852682 | 23656302 | 25282059 | 27352137 | 28536511 | 29273199 | 29740406 |
| 19545796 | 20834198 | 22150651 | 22852685 | 23656500 | 25282181 | 27353340 | 28536517 | 29273202 | 29740660 |
| 19545928 | 20834290 | 22150794 | 22852776 | 23656736 | 25283990 | 27353531 | 28536554 | 29273208 | 29741684 |
| 19546104 | 20835151 | 22150825 | 22852875 | 23656921 | 25284195 | 27353552 | 28537134 | 29273229 | 29742138 |
| 19547111 | 20835304 | 22150892 | 22853226 | 23657056 | 25284628 | 27353741 | 28537272 | 29273249 | 29742230 |
| 19547624 | 20835960 | 22150981 | 22853303 | 23657785 | 25284945 | 27353845 | 28537719 | 29273253 | 29742402 |
| 19547824 | 20836117 | 22151270 | 22853392 | 23658010 | 25285055 | 27353914 | 28538041 | 29273254 | 29742997 |
| 19548330 | 20836245 | 22151732 | 22853455 | 23658280 | 25285119 | 27354093 | 28539846 | 29273255 | 29743411 |
| 19548387 | 20836327 | 22151779 | 22853511 | 23658871 | 25285670 | 27354222 | 28540269 | 29273259 | 29744196 |
| 19548390 | 20836770 | 22151873 | 22853655 | 23658973 | 25286738 | 27354293 | 28540761 | 29273270 | 29744308 |
| 19548653 | 20837181 | 22151889 | 22853656 | 23659271 | 25287499 | 27354407 | 28541077 | 29273292 | 29744382 |
| 19548722 | 20837642 | 22152395 | 22854054 | 23659413 | 25288792 | 27354436 | 28541095 | 29273311 | 29744783 |
| 19548751 | 20837824 | 22152673 | 22854651 | 23660954 | 25288837 | 27354669 | 28541524 | 29273320 | 29744794 |
| 19548991 | 20838700 | 22152997 | 22854753 | 23661018 | 25289132 | 27355743 | 28542497 | 29273335 | 29745196 |
| 19549072 | 20838774 | 22153033 | 22855058 | 23661028 | 25289521 | 27355883 | 28543034 | 29273344 | 29745359 |
| 19549132 | 20839538 | 22153257 | 22855453 | 23661915 | 25290093 | 27356422 | 28543096 | 29273356 | 29746010 |
| 19550091 | 20839587 | 22153410 | 22855851 | 23662483 | 25291158 | 27356882 | 28543263 | 29273360 | 29746222 |
| 19550108 | 20839628 | 22153593 | 22856047 | 23662795 | 25291422 | 27357053 | 28544605 | 29273369 | 29748561 |
| 19552145 | 20839932 | 22153615 | 22856685 | 23663341 | 25291872 | 27357328 | 28544963 | 29273371 | 29748585 |
| 19552684 | 20841009 | 22153963 | 22856823 | 23663574 | 25292330 | 27358150 | 28545026 | 29273383 | 29749046 |
| 19552729 | 20841222 | 22154038 | 22856995 | 23664314 | 25292336 | 27358271 | 28545156 | 29273390 | 29749254 |
| 19552963 | 20841331 | 22154043 | 22857242 | 23664632 | 25292431 | 27359729 | 28545512 | 29273391 | 29749543 |
| 19553696 | 20841337 | 22154388 | 22857549 | 23664650 | 25293202 | 27359734 | 28546798 | 29273438 | 29750576 |
| 19553794 | 20842987 | 22154891 | 22857593 | 23664846 | 25293546 | 27360034 | 28546988 | 29273445 | 29750844 |
| 19554022 | 20843815 | 22154920 | 22858252 | 23665205 | 25294749 | 27360238 | 28547238 | 29273446 | 29750924 |
| 19554154 | 20843829 | 22155253 | 22858363 | 23665697 | 25294809 | 27360977 | 28547367 | 29273447 | 29751151 |
| 19554632 | 20844310 | 22155384 | 22858864 | 23666594 | 25296165 | 27361288 | 28547378 | 29273456 | 29751371 |
| 19554960 | 20844566 | 22155470 | 22859424 | 23666615 | 25296623 | 27361660 | 28547527 | 29273457 | 29751566 |
| 19555196 | 20845263 | 22155936 | 22859569 | 23666773 | 25297534 | 27361895 | 28547592 | 29273463 | 29751979 |
| 19555287 | 20845354 | 22156177 | 22859618 | 23667322 | 25300296 | 27362059 | 28548030 | 29273466 | 29752289 |
| 19556059 | 20846449 | 22156481 | 22859723 | 23667491 | 25300792 | 27362199 | 28548225 | 29273472 | 29752950 |
| 19556087 | 20846701 | 22156634 | 22859957 | 23668039 | 25300895 | 27362739 | 28548494 | 29273480 | 29753043 |
| 19556666 | 20846944 | 22156639 | 22860036 | 23668534 | 25303271 | 27362908 | 28548577 | 29273482 | 29753045 |
| 19557017 | 20847263 | 22156964 | 22860359 | 23668779 | 25303560 | 27362938 | 28549266 | 29273515 | 29753304 |
| 19557039 | 20848685 | 22157439 | 22860569 | 23668949 | 25304503 | 27363521 | 28549469 | 29273531 | 29753526 |
| 19557496 | 20849772 | 22157727 | 22861318 | 23669322 | 25304953 | 27363660 | 28550001 | 29273537 | 29753810 |
| 19557876 | 20850487 | 22158171 | 22861589 | 23669450 | 25306914 | 27363927 | 28550014 | 29273539 | 29754110 |
| 19558297 | 20850885 | 22158655 | 22861650 | 23669827 | 25309366 | 27364234 | 28550787 | 29273549 | 29755385 |
| 19558470 | 20851086 | 22159019 | 22861722 | 23670020 | 25310275 | 27365114 | 28551204 | 29273553 | 29755526 |
| 19558659 | 20851198 | 22159179 | 22861964 | 23670588 | 25312079 | 27365251 | 28551872 | 29273563 | 29755982 |
| 19558844 | 20851642 | 22159957 | 22862092 | 23670662 | 25313033 | 27365893 | 28551877 | 29273573 | 29756135 |
| 19558904 | 20851730 | 22160034 | 22862151 | 23671227 | 25314776 | 27366113 | 28551940 | 29273585 | 29756314 |
| 19558940 | 20852292 | 22160274 | 22862375 | 23672435 | 25315110 | 27366564 | 28552477 | 29273594 | 29756868 |
| 19559528 | 20852724 | 22160341 | 22862530 | 23672697 | 25315489 | 27366947 | 28553262 | 29273595 | 29756876 |
| 19559632 | 20852742 | 22160639 | 22862781 | 23673615 | 25315800 | 27367231 | 28553337 | 29273600 | 29756935 |
| 19559889 | 20853103 | 22160679 | 22862822 | 23673629 | 25318209 | 27368100 | 28553400 | 29273604 | 29757734 |
| 19561113 | 20854894 | 22160795 | 22862877 | 23673689 | 25321344 | 27368372 | 28554358 | 29273609 | 29758080 |
| 19561226 | 20856145 | 22160869 | 22863269 | 23674291 | 25323191 | 27368484 | 28554406 | 29273627 | 29758280 |
| 19561374 | 20856215 | 22161447 | 22863460 | 23674800 | 25323333 | 27368675 | 28555467 | 29273628 | 29758711 |
| 19561387 | 20856233 | 22161963 | 22863797 | 23674859 | 25323445 | 27368998 | 28555617 | 29273662 | 29759244 |
| 19562648 | 20856235 | 22162218 | 22863859 | 23675072 | 25326416 | 27369304 | 28555675 | 29273664 | 29759440 |
| 19562669 | 20856538 | 22162530 | 22864254 | 23675374 | 25327687 | 27369568 | 28555695 | 29273684 | 29760212 |
| 19562679 | 20856797 | 22163241 | 22864762 | 23675475 | 25329144 | 27369596 | 28555803 | 29273686 | 29760277 |
| 19562834 | 20857252 | 22163427 | 22864792 | 23675635 | 25329991 | 27371685 | 28556039 | 29273688 | 29761675 |
| 19563503 | 20858218 | 22163494 | 22864822 | 23676147 | 25330338 | 27371876 | 28556129 | 29273695 | 29761879 |
| 19564424 | 20859055 | 22163929 | 22864934 | 23676666 | 25331049 | 27372529 | 28556688 | 29273700 | 29762168 |
| 19565934 | 20859829 | 22164200 | 22865148 | 23676809 | 25332645 | 27372661 | 28556884 | 29273712 | 29762826 |
| 19566040 | 20860189 | 22164364 | 22865525 | 23676905 | 25333483 | 27372954 | 28556961 | 29273726 | 29762940 |
| 19566162 | 20861450 | 22164419 | 22865916 | 23677290 | 25333609 | 27372990 | 28557157 | 29273728 | 29763034 |
| 19566304 | 20862208 | 22164678 | 22866510 | 23677626 | 25334506 | 27373752 | 28557373 | 29273732 | 29763318 |
| 19566463 | 20862639 | 22164828 | 22866525 | 23677787 | 25335074 | 27374037 | 28557463 | 29273738 | 29763328 |
| 19567197 | 20863120 | 22164859 | 22866743 | 23678026 | 25335095 | 27375026 | 28558602 | 29273752 | 29763599 |
| 19567551 | 20863773 | 22164948 | 22866784 | 23678069 | 25335385 | 27375120 | 28558858 | 29273753 | 29764045 |
| 19567693 | 20864813 | 22165026 | 22867038 | 23678648 | 25335635 | 27375905 | 28559454 | 29273755 | 29764233 |
| 19568442 | 20864820 | 22165396 | 22867067 | 23678779 | 25335794 | 27376234 | 28559957 | 29273765 | 29764869 |
| 19568458 | 20864999 | 22165491 | 22867403 | 23678788 | 25337028 | 27376765 | 28560096 | 29273770 | 29764884 |
| 19568607 | 20865292 | 22165551 | 22867509 | 23678866 | 25339812 | 27376787 | 28560206 | 29273771 | 29765230 |
| 19568647 | 20865753 | 22166661 | 22867624 | 23678938 | 25340293 | 27376854 | 28560650 | 29273774 | 29765310 |
| 19568794 | 20866072 | 22166868 | 22868047 | 23679022 | 25342671 | 27377235 | 28560653 | 29273778 | 29765774 |
| 19568795 | 20866078 | 22166934 | 22868123 | 23679385 | 25343572 | 27378848 | 28560724 | 29273786 | 29767465 |
| 19569787 | 20868751 | 22166977 | 22868130 | 23679438 | 25344389 | 27378865 | 28560946 | 29273791 | 29767692 |
| 19570835 | 20869576 | 22167033 | 22868819 | 23679956 | 25345016 | 27379141 | 28561012 | 29273792 | 29767768 |
| 19571427 | 20871755 | 22167053 | 22869017 | 23680082 | 25345210 | 27379689 | 28562728 | 29273803 | 29767804 |
| 19572114 | 20871824 | 22167131 | 22869090 | 23680273 | 25347015 | 27380303 | 28562843 | 29273812 | 29768061 |
| 19572943 | 20872063 | 22167422 | 22869163 | 23680305 | 25347278 | 27380634 | 28563635 | 29273820 | 29768263 |
| 19573283 | 20873188 | 22167728 | 22869238 | 23680458 | 25348108 | 27380810 | 28564042 | 29273825 | 29769076 |
| 19573344 | 20873405 | 22167950 | 22869812 | 23680891 | 25348561 | 27381042 | 28564338 | 29273826 | 29769911 |
| 19574126 | 20873588 | 22168111 | 22869914 | 23681966 | 25348644 | 27381128 | 28564424 | 29273831 | 29770613 |
| 19574535 | 20873676 | 22168471 | 22870304 | 23682542 | 25350985 | 27381485 | 28564440 | 29273833 | 29770766 |
| 19574709 | 20873776 | 22168597 | 22870382 | 23682677 | 25352436 | 27381508 | 28566052 | 29273844 | 29770980 |
| 19575073 | 20874088 | 22169047 | 22870530 | 23684115 | 25352923 | 27381977 | 28566354 | 29273859 | 29771356 |
| 19575549 | 20874585 | 22169051 | 22870773 | 23684182 | 25353775 | 27382240 | 28567039 | 29273864 | 29771367 |
| 19575864 | 20874907 | 22169079 | 22870784 | 23684409 | 25354683 | 27382698 | 28567506 | 29273865 | 29773722 |
| 19576013 | 20875239 | 22169157 | 22871038 | 23684876 | 25354709 | 27382831 | 28567779 | 29273867 | 29774773 |
| 19576405 | 20876010 | 22169526 | 22871467 | 23684896 | 25355171 | 27382926 | 28567839 | 29273880 | 29774949 |
| 19576926 | 20876272 | 22170151 | 22871484 | 23685486 | 25355335 | 27383277 | 28568331 | 29273889 | 29775010 |
| 19577175 | 20876575 | 22170391 | 22871505 | 23685842 | 25355792 | 27383305 | 28568514 | 29273899 | 29775077 |
| 19577451 | 20876600 | 22170547 | 22871597 | 23686460 | 25356123 | 27384051 | 28569062 | 29273901 | 29775182 |
| 19577721 | 20876721 | 22170649 | 22871698 | 23686656 | 25356399 | 27384200 | 28569546 | 29273905 | 29775192 |
| 19578442 | 20878129 | 22170762 | 22872116 | 23687205 | 25356833 | 27384549 | 28569599 | 29273919 | 29776206 |
| 19579359 | 20878876 | 22170874 | 22872776 | 23687886 | 25357718 | 27385621 | 28569807 | 29273927 | 29776632 |
| 19579431 | 20879791 | 22171570 | 22872832 | 23688769 | 25359407 | 27386678 | 28570706 | 29273928 | 29776703 |
| 19579595 | 20879907 | 22171626 | 22872853 | 23689052 | 25359439 | 27386929 | 28571146 | 29273930 | 29776708 |
| 19579614 | 20880417 | 22172157 | 22873041 | 23689162 | 25360576 | 27386961 | 28571166 | 29273945 | 29777181 |
| 19580006 | 20880927 | 22172310 | 22873112 | 23689350 | 25360700 | 27386970 | 28572077 | 29273946 | 29777406 |
| 19581070 | 20882655 | 22172718 | 22873155 | 23689555 | 25361153 | 27387256 | 28572283 | 29273948 | 29778098 |
| 19581162 | 20882669 | 22172762 | 22874027 | 23689662 | 25363655 | 27387400 | 28572516 | 29273950 | 29778189 |
| 19581830 | 20883861 | 22173684 | 22874035 | 23689890 | 25366699 | 27387581 | 28573379 | 29273959 | 29778191 |
| 19582074 | 20884415 | 22173878 | 22874202 | 23690462 | 25368674 | 27388097 | 28573763 | 29273995 | 29778649 |
| 19582689 | 20886853 | 22174001 | 22874277 | 23690980 | 25369700 | 27388355 | 28573899 | 29274000 | 29779503 |
| 19583205 | 20887235 | 22174100 | 22874317 | 23691062 | 25372316 | 27388367 | 28574415 | 29274002 | 29779705 |
| 19583213 | 20887317 | 22174141 | 22874454 | 23691170 | 25372449 | 27388513 | 28574569 | 29274008 | 29779931 |
| 19583276 | 20887447 | 22174333 | 22874485 | 23691212 | 25373905 | 27388786 | 28574621 | 29274013 | 29781243 |
| 19583667 | 20888782 | 22174359 | 22874531 | 23691216 | 25376226 | 27389582 | 28574913 | 29274026 | 29781277 |
| 19584125 | 20889088 | 22174455 | 22874539 | 23691335 | 25380559 | 27389789 | 28575204 | 29274045 | 29781643 |
| 19584232 | 20890101 | 22174575 | 22874784 | 23691434 | 25380751 | 27389879 | 28575324 | 29274055 | 29781692 |
| 19585085 | 20890231 | 22174661 | 22875965 | 23692294 | 25380885 | 27389897 | 28575960 | 29274056 | 29781728 |
| 19585885 | 20891459 | 22174926 | 22876556 | 23692670 | 25381757 | 27390109 | 28576096 | 29274060 | 29782487 |
| 19586558 | 20891815 | 22176519 | 22876735 | 23692818 | 25382665 | 27390567 | 28576157 | 29274061 | 29783700 |
| 19586976 | 20892046 | 22176712 | 22876943 | 23692937 | 25382858 | 27390577 | 28576176 | 29274064 | 29783871 |
| 19587645 | 20892585 | 22176713 | 22877195 | 23693046 | 25384151 | 27390863 | 28576354 | 29274067 | 29784021 |
| 19587952 | 20893049 | 22176959 | 22877347 | 23693636 | 25386407 | 27391254 | 28576864 | 29274073 | 29784169 |
| 19588123 | 20893124 | 22177283 | 22877640 | 23693711 | 25386421 | 27391501 | 28577557 | 29274077 | 29784223 |
| 19589176 | 20893133 | 22177827 | 22877747 | 23693843 | 25387424 | 27391693 | 28577875 | 29274088 | 29784721 |
| 19589533 | 20893239 | 22179607 | 22878110 | 23693948 | 25388375 | 27392063 | 28578144 | 29274098 | 29784984 |
| 19589650 | 20893378 | 22179858 | 22878784 | 23694425 | 25388494 | 27393756 | 28578192 | 29274101 | 29786129 |
| 19590031 | 20893692 | 22180093 | 22879084 | 23694572 | 25389188 | 27394348 | 28578364 | 29274108 | 29786525 |
| 19591318 | 20894882 | 22180165 | 22879195 | 23694575 | 25391324 | 27394360 | 28578406 | 29274109 | 29786994 |
| 19591541 | 20896365 | 22180489 | 22879809 | 23694605 | 25392843 | 27394708 | 28578537 | 29274110 | 29787067 |
| 19591696 | 20896549 | 22180779 | 22880034 | 23694756 | 25393047 | 27394713 | 28579140 | 29274120 | 29788115 |
| 19591821 | 20896961 | 22180965 | 22880671 | 23695518 | 25394580 | 27395160 | 28579218 | 29274127 | 29789381 |
| 19592016 | 20897016 | 22181405 | 22880814 | 23696722 | 25396885 | 27395720 | 28579309 | 29274133 | 29789517 |
| 19592020 | 20897420 | 22181503 | 22880993 | 23696988 | 25397115 | 27396993 | 28580074 | 29274147 | 29789649 |
| 19592108 | 20898275 | 22181538 | 22881084 | 23697053 | 25397350 | 27398447 | 28580301 | 29274151 | 29790239 |
| 19592460 | 20898379 | 22181655 | 22881283 | 23697499 | 25397392 | 27399443 | 28580362 | 29274152 | 29790837 |
| 19592530 | 20898434 | 22181937 | 22881794 | 23697724 | 25397708 | 27399668 | 28580411 | 29274164 | 29790848 |
| 19592617 | 20898570 | 22181985 | 22882040 | 23697737 | 25397788 | 27399959 | 28580498 | 29274197 | 29791115 |
| 19592884 | 20898755 | 22182067 | 22882892 | 23698032 | 25398193 | 27400362 | 28581298 | 29274202 | 29791216 |
| 19592895 | 20898785 | 22182343 | 22882920 | 23699455 | 25398357 | 27400854 | 28581527 | 29274208 | 29791225 |
| 19593338 | 20898987 | 22182968 | 22882986 | 23699593 | 25400670 | 27400932 | 28582370 | 29274220 | 29791466 |
| 19593357 | 20899881 | 22183416 | 22883004 | 23699742 | 25401944 | 27401920 | 28582376 | 29274226 | 29791714 |
| 19593513 | 20900321 | 22183850 | 22883057 | 23699778 | 25402186 | 27402364 | 28582535 | 29274264 | 29791796 |
| 19593678 | 20901124 | 22184012 | 22883263 | 23700116 | 25402812 | 27403029 | 28582557 | 29274268 | 29791960 |
| 19593783 | 20901387 | 22184027 | 22883406 | 23700218 | 25402993 | 27403094 | 28582797 | 29274287 | 29792133 |
| 19593843 | 20901409 | 22184063 | 22883471 | 23700265 | 25403111 | 27403279 | 28583579 | 29274289 | 29792501 |
| 19594282 | 20902046 | 22184067 | 22883662 | 23700971 | 25403715 | 27403291 | 28583730 | 29274294 | 29792853 |
| 19594445 | 20902332 | 22184139 | 22883814 | 23701496 | 25403772 | 27403534 | 28583957 | 29274298 | 29792940 |
| 19594473 | 20902859 | 22184142 | 22884004 | 23701877 | 25404556 | 27403735 | 28585216 | 29274309 | 29793416 |
| 19594679 | 20904697 | 22184330 | 22884190 | 23701940 | 25405081 | 27403834 | 28585720 | 29274313 | 29793567 |
| 19594787 | 20904704 | 22184534 | 22884244 | 23702201 | 25406989 | 27403853 | 28586188 | 29274323 | 29794247 |
| 19594813 | 20904718 | 22185473 | 22884423 | 23702226 | 25407252 | 27404093 | 28586581 | 29274327 | 29794405 |
| 19594908 | 20905357 | 22185642 | 22884860 | 23702294 | 25408138 | 27404895 | 28587213 | 29274334 | 29795989 |
| 19595446 | 20906028 | 22185649 | 22885167 | 23702372 | 25408224 | 27405111 | 28587801 | 29274337 | 29796122 |
| 19595479 | 20906206 | 22185688 | 22885192 | 23702406 | 25408251 | 27405662 | 28588191 | 29274348 | 29796714 |
| 19595645 | 20906367 | 22186181 | 22885211 | 23702452 | 25411895 | 27405746 | 28588361 | 29274360 | 29796737 |
| 19595841 | 20906637 | 22186479 | 22885218 | 23703877 | 25413095 | 27406070 | 28588400 | 29274365 | 29797602 |
| 19596125 | 20908432 | 22186767 | 22885312 | 23703986 | 25413233 | 27406916 | 28588544 | 29274370 | 29797712 |
| 19596186 | 20910520 | 22187026 | 22885626 | 23704191 | 25413581 | 27406964 | 28589630 | 29274385 | 29797774 |
| 19596434 | 20911055 | 22187828 | 22886176 | 23705434 | 25413944 | 27407054 | 28590611 | 29274392 | 29798009 |
| 19596454 | 20912169 | 22188157 | 22886435 | 23705744 | 25415652 | 27407478 | 28591233 | 29274397 | 29798484 |
| 19596859 | 20912665 | 22188520 | 22886666 | 23705769 | 25417035 | 27408635 | 28591355 | 29274399 | 29798732 |
| 19596884 | 20912804 | 22188710 | 22886668 | 23705834 | 25418605 | 27411026 | 28591856 | 29274400 | 29799123 |
| 19596933 | 20913779 | 22189410 | 22886888 | 23705844 | 25419594 | 27411161 | 28591927 | 29274406 | 29799241 |
| 19597716 | 20913876 | 22189616 | 22886892 | 23706630 | 25421998 | 27412451 | 28592010 | 29274422 | 29799303 |
| 19597896 | 20914448 | 22189763 | 22887111 | 23706836 | 25422164 | 27412584 | 28593128 | 29274424 | 29799697 |
| 19598290 | 20915463 | 22189766 | 22887525 | 23707733 | 25422372 | 27412771 | 28593364 | 29274425 | 29799798 |
| 19599573 | 20915644 | 22190042 | 22887547 | 23707764 | 25423103 | 27412811 | 28594238 | 29274437 | 29800010 |
| 19599705 | 20917449 | 22190192 | 22887749 | 23707908 | 25423250 | 27413075 | 28594872 | 29274445 | 29801292 |
| 19599765 | 20917778 | 22190331 | 22887899 | 23708596 | 25423550 | 27413728 | 28594932 | 29274449 | 29801790 |
| 19599839 | 20917897 | 22190370 | 22888294 | 23708922 | 25424539 | 27413992 | 28596118 | 29274451 | 29802405 |
| 19599845 | 20917986 | 22190940 | 22888691 | 23709130 | 25426364 | 27414358 | 28596716 | 29274458 | 29802538 |
| 19600567 | 20918034 | 22191269 | 22888757 | 23709399 | 25427454 | 27415570 | 28596800 | 29274459 | 29803618 |
| 19600661 | 20918257 | 22191284 | 22889005 | 23709783 | 25427494 | 27415740 | 28597046 | 29274468 | 29804131 |
| 19601170 | 20918448 | 22191786 | 22889178 | 23711405 | 25427860 | 27415827 | 28597232 | 29274475 | 29804845 |
| 19601554 | 20918733 | 22191926 | 22889295 | 23711743 | 25428735 | 27416038 | 28597245 | 29274500 | 29805558 |
| 19601663 | 20919035 | 22192067 | 22889860 | 23712472 | 25429608 | 27416243 | 28597510 | 29274515 | 29806218 |
| 19601962 | 20920345 | 22192166 | 22889914 | 23712801 | 25431343 | 27416928 | 28597546 | 29274521 | 29806440 |
| 19601965 | 20921056 | 22192212 | 22890131 | 23712851 | 25432102 | 27417067 | 28597587 | 29274522 | 29809327 |
| 19602806 | 20922019 | 22192258 | 22890448 | 23712999 | 25432143 | 27418006 | 28597592 | 29274523 | 29809362 |
| 19603044 | 20922434 | 22192296 | 22890649 | 23713148 | 25432259 | 27418620 | 28597711 | 29274565 | 29810178 |
| 19603420 | 20922567 | 22192384 | 22890958 | 23713361 | 25436836 | 27418985 | 28598105 | 29274569 | 29810202 |
| 19603762 | 20924280 | 22192406 | 22890978 | 23713979 | 25436858 | 27419553 | 28598225 | 29274595 | 29811662 |
| 19604781 | 20924356 | 22192532 | 22890993 | 23714587 | 25439669 | 27420397 | 28598412 | 29274597 | 29811689 |
| 19605651 | 20924836 | 22192606 | 22891596 | 23714659 | 25439810 | 27420896 | 28598990 | 29274601 | 29815075 |
| 19605917 | 20925454 | 22192711 | 22891629 | 23714902 | 25440193 | 27421183 | 28599058 | 29274609 | 29815321 |
| 19605971 | 20925762 | 22192748 | 22891790 | 23715611 | 25441040 | 27421439 | 28599681 | 29274613 | 29815903 |
| 19606025 | 20925785 | 22192897 | 22892518 | 23715957 | 25441863 | 27421496 | 28599866 | 29274626 | 29816168 |
| 19607313 | 20927051 | 22192980 | 22893340 | 23716224 | 25442113 | 27422703 | 28601265 | 29274651 | 29816404 |
| 19608158 | 20927080 | 22193437 | 22893911 | 23717370 | 25442385 | 27422774 | 28603424 | 29274657 | 29816735 |
| 19608303 | 20927712 | 22194151 | 22893938 | 23717850 | 25443508 | 27425082 | 28603570 | 29274663 | 29816986 |
| 19608557 | 20927713 | 22194515 | 22894127 | 23717944 | 25443786 | 27425314 | 28603827 | 29274671 | 29817830 |
| 19608692 | 20929171 | 22195484 | 22894408 | 23717952 | 25443914 | 27425721 | 28604119 | 29274672 | 29818308 |
| 19609152 | 20929397 | 22195558 | 22894539 | 23718165 | 25447272 | 27426033 | 28604603 | 29274674 | 29818679 |
| 19609456 | 20929538 | 22195619 | 22894834 | 23718298 | 25447610 | 27426189 | 28605206 | 29274690 | 29819484 |
| 19610093 | 20929883 | 22196351 | 22894896 | 23719295 | 25447615 | 27427282 | 28605216 | 29274693 | 29819840 |
| 19610297 | 20930172 | 22196485 | 22895247 | 23719344 | 25448285 | 27428679 | 28605561 | 29274695 | 29820265 |
| 19610826 | 20930311 | 22196683 | 22895308 | 23719691 | 25448669 | 27428713 | 28605654 | 29274715 | 29820285 |
| 19610876 | 20930533 | 22196755 | 22896126 | 23720031 | 25449550 | 27429606 | 28606295 | 29274717 | 29820562 |
| 19612513 | 20930663 | 22197046 | 22896610 | 23720517 | 25450106 | 27430786 | 28606811 | 29274729 | 29821736 |
| 19614020 | 20930860 | 22197261 | 22896691 | 23720887 | 25450334 | 27430823 | 28607049 | 29274731 | 29822488 |
| 19615019 | 20931066 | 22197477 | 22896750 | 23721605 | 25451138 | 27431455 | 28607076 | 29274736 | 29822544 |
| 19615162 | 20931456 | 22197928 | 22897210 | 23721925 | 25452932 | 27431977 | 28607352 | 29274750 | 29823082 |
| 19615378 | 20931694 | 22198623 | 22897262 | 23721966 | 25454361 | 27432099 | 28608230 | 29274753 | 29823111 |
| 19615542 | 20932170 | 22198971 | 22897427 | 23722052 | 25456788 | 27432438 | 28608237 | 29274754 | 29823167 |
| 19615744 | 20932276 | 22199380 | 22897569 | 23722981 | 25456876 | 27433498 | 28609119 | 29274756 | 29823462 |
| 19616184 | 20935045 | 22199471 | 22897830 | 23723049 | 25457597 | 27433643 | 28609516 | 29274761 | 29823483 |
| 19616638 | 20935086 | 22200008 | 22897916 | 23723371 | 25458068 | 27434043 | 28609798 | 29274771 | 29823704 |
| 19616793 | 20935420 | 22200277 | 22898191 | 23723494 | 25458542 | 27434080 | 28611022 | 29274776 | 29823912 |
| 19617555 | 20936525 | 22202053 | 22898330 | 23724224 | 25458957 | 27434181 | 28611031 | 29274789 | 29823920 |
| 19617839 | 20937261 | 22202153 | 22898513 | 23724317 | 25459382 | 27435294 | 28611079 | 29274794 | 29824072 |
| 19618254 | 20937355 | 22202248 | 22898598 | 23724584 | 25459690 | 27435693 | 28611332 | 29274797 | 29824364 |
| 19618675 | 20937545 | 22202510 | 22899139 | 23724954 | 25460093 | 27435736 | 28611599 | 29274800 | 29824512 |
| 19618911 | 20937670 | 22202650 | 22899165 | 23725045 | 25460311 | 27436063 | 28611914 | 29274805 | 29824560 |
| 19618927 | 20937875 | 22202870 | 22899207 | 23725451 | 25460601 | 27436833 | 28612491 | 29274835 | 29824611 |
| 19619442 | 20938653 | 22203264 | 22899272 | 23725599 | 25460801 | 27437659 | 28612696 | 29274836 | 29824735 |
| 19619736 | 20939086 | 22203391 | 22899555 | 23727004 | 25462408 | 27438212 | 28612932 | 29274839 | 29825026 |
| 19619741 | 20939150 | 22203415 | 22899914 | 23727044 | 25462696 | 27438233 | 28613105 | 29274846 | 29825263 |
| 19619869 | 20939483 | 22204246 | 22900103 | 23727400 | 25462726 | 27438294 | 28613341 | 29274854 | 29825575 |
| 19621223 | 20939898 | 22204306 | 22900284 | 23727526 | 25464036 | 27438392 | 28613432 | 29274863 | 29825731 |
| 19621430 | 20939977 | 22204419 | 22900731 | 23727682 | 25465391 | 27438625 | 28613693 | 29274870 | 29828564 |
| 19621764 | 20941038 | 22204612 | 22900858 | 23727815 | 25466593 | 27438648 | 28614638 | 29274871 | 29828603 |
| 19622288 | 20941298 | 22205508 | 22901709 | 23729189 | 25466928 | 27439284 | 28614699 | 29274874 | 29829129 |
| 19623479 | 20941382 | 22205651 | 22901847 | 23729289 | 25467001 | 27440167 | 28615050 | 29274875 | 29829407 |
| 19623557 | 20941705 | 22206217 | 22901930 | 23729367 | 25467511 | 27440463 | 28615370 | 29274883 | 29829501 |
| 19624357 | 20941860 | 22206559 | 22902049 | 23730287 | 25467942 | 27441110 | 28615763 | 29274887 | 29830037 |
| 19624608 | 20942182 | 22206827 | 22902199 | 23731010 | 25468282 | 27441243 | 28616251 | 29274892 | 29830188 |
| 19624897 | 20942785 | 22206854 | 22902230 | 23731317 | 25468690 | 27442748 | 28616568 | 29274894 | 29830652 |
| 19624975 | 20943167 | 22207012 | 22902346 | 23731445 | 25469906 | 27444936 | 28616991 | 29274900 | 29830855 |
| 19625117 | 20943549 | 22207782 | 22902595 | 23731902 | 25470078 | 27445475 | 28617023 | 29274906 | 29831183 |
| 19626152 | 20943573 | 22207803 | 22902607 | 23733372 | 25470135 | 27445840 | 28617172 | 29274930 | 29831824 |
| 19626184 | 20943750 | 22207906 | 22902756 | 23733468 | 25471628 | 27445844 | 28617228 | 29274941 | 29832415 |
| 19626332 | 20944522 | 22207996 | 22903297 | 23734237 | 25471960 | 27446543 | 28617293 | 29274953 | 29832829 |
| 19626711 | 20944756 | 22208045 | 22903418 | 23734524 | 25472189 | 27446750 | 28617440 | 29274958 | 29833777 |
| 19627164 | 20944841 | 22208409 | 22903509 | 23734778 | 25474289 | 27446895 | 28618072 | 29274961 | 29834311 |
| 19627252 | 20944973 | 22208762 | 22903535 | 23734805 | 25475467 | 27447119 | 28618521 | 29274965 | 29834977 |
| 19627962 | 20945011 | 22208852 | 22903565 | 23735058 | 25475742 | 27447220 | 28618925 | 29274983 | 29835071 |
| 19628655 | 20945867 | 22208853 | 22903655 | 23735902 | 25476133 | 27448288 | 28618974 | 29274992 | 29835880 |
| 19628734 | 20946274 | 22209137 | 22903798 | 23735914 | 25476506 | 27448425 | 28619632 | 29274996 | 29836111 |
| 19629005 | 20946372 | 22209285 | 22904199 | 23736239 | 25476561 | 27448426 | 28620220 | 29274997 | 29836897 |
| 19630887 | 20946579 | 22209317 | 22904708 | 23736496 | 25476990 | 27448887 | 28620420 | 29275002 | 29837318 |
| 19630933 | 20947844 | 22209687 | 22904791 | 23736853 | 25477092 | 27449008 | 28621010 | 29275003 | 29837330 |
| 19631677 | 20948294 | 22209995 | 22905261 | 23737660 | 25479103 | 27449357 | 28621489 | 29275005 | 29838003 |
| 19632472 | 20948327 | 22210015 | 22905412 | 23737928 | 25479412 | 27449703 | 28622115 | 29275010 | 29838241 |
| 19632618 | 20948353 | 22210509 | 22905495 | 23737984 | 25479934 | 27450025 | 28622223 | 29275013 | 29839101 |
| 19632656 | 20948936 | 22210661 | 22905500 | 23738133 | 25479935 | 27450027 | 28622985 | 29275017 | 29839316 |
| 19634909 | 20949273 | 22210802 | 22905833 | 23738991 | 25480988 | 27451007 | 28623239 | 29275026 | 29839674 |
| 19634997 | 20949710 | 22211055 | 22906462 | 23739561 | 25481233 | 27451059 | 28623368 | 29275027 | 29839947 |
| 19635218 | 20951359 | 22211451 | 22906539 | 23739577 | 25481855 | 27451274 | 28623557 | 29275039 | 29840838 |
| 19635272 | 20951822 | 22211610 | 22906789 | 23739729 | 25482019 | 27451366 | 28624569 | 29275042 | 29841796 |
| 19635537 | 20951963 | 22212075 | 22907354 | 23740066 | 25483406 | 27452279 | 28624610 | 29275044 | 29842384 |
| 19635801 | 20952372 | 22212103 | 22907412 | 23740176 | 25483491 | 27452598 | 28624859 | 29275066 | 29843102 |
| 19636032 | 20954317 | 22212328 | 22908019 | 23740427 | 25483517 | 27452806 | 28624956 | 29275081 | 29843169 |
| 19636338 | 20955338 | 22212374 | 22908299 | 23740571 | 25486067 | 27452908 | 28625029 | 29275087 | 29843315 |
| 19636702 | 20955599 | 22212421 | 22909317 | 23740675 | 25486072 | 27453226 | 28625329 | 29275115 | 29843535 |
| 19637084 | 20955627 | 22213074 | 22909415 | 23741591 | 25486570 | 27453511 | 28625370 | 29275144 | 29844045 |
| 19637157 | 20956527 | 22213270 | 22910150 | 23741993 | 25486857 | 27453771 | 28625568 | 29275152 | 29844946 |
| 19637405 | 20957534 | 22213296 | 22910181 | 23742448 | 25487541 | 27454135 | 28625886 | 29275158 | 29845007 |
| 19638712 | 20958004 | 22213728 | 22910271 | 23742535 | 25488694 | 27454691 | 28626630 | 29275167 | 29846256 |
| 19638956 | 20958220 | 22214089 | 22910581 | 23743115 | 25489368 | 27455082 | 28626839 | 29275175 | 29847365 |
| 19639246 | 20958621 | 22214354 | 22910663 | 23743410 | 25490058 | 27455841 | 28626922 | 29275176 | 29847455 |
| 19639336 | 20958938 | 22214363 | 22910861 | 23743522 | 25490064 | 27455941 | 28627265 | 29275179 | 29847601 |
| 19639381 | 20959287 | 22214469 | 22911003 | 23744039 | 25490667 | 27455960 | 28627321 | 29275183 | 29847710 |
| 19639514 | 20959717 | 22214697 | 22911071 | 23744079 | 25492290 | 27455982 | 28627547 | 29275190 | 29847799 |
| 19639930 | 20959783 | 22214883 | 22911145 | 23744186 | 25492509 | 27456075 | 28628176 | 29275198 | 29848266 |
| 19641214 | 20960221 | 22214983 | 22911209 | 23744702 | 25494282 | 27456193 | 28628318 | 29275213 | 29848757 |
| 19642020 | 20960673 | 22215001 | 22911510 | 23744801 | 25495034 | 27456565 | 28628525 | 29275215 | 29848974 |
| 19642214 | 20961228 | 22215218 | 22911540 | 23745344 | 25495053 | 27457671 | 28628648 | 29275216 | 29849683 |
| 19642303 | 20961335 | 22215683 | 22912582 | 23746864 | 25495504 | 27458079 | 28629021 | 29275225 | 29850068 |
| 19642453 | 20961657 | 22215803 | 22913340 | 23747039 | 25498603 | 27458279 | 28629186 | 29275234 | 29850482 |
| 19642973 | 20963062 | 22215901 | 22913443 | 23747916 | 25498876 | 27458285 | 28629441 | 29275235 | 29850685 |
| 19643093 | 20963352 | 22216062 | 22913535 | 23748571 | 25500004 | 27459250 | 28630102 | 29275244 | 29851589 |
| 19643217 | 20963468 | 22216660 | 22913618 | 23748826 | 25500101 | 27460096 | 28630148 | 29275256 | 29851595 |
| 19644709 | 20963704 | 22216664 | 22913720 | 23749196 | 25501586 | 27460794 | 28630242 | 29275259 | 29852004 |
| 19646369 | 20964625 | 22216794 | 22914478 | 23749341 | 25502014 | 27461287 | 28630859 | 29275261 | 29852040 |
| 19647210 | 20964875 | 22217099 | 22914851 | 23749520 | 25502079 | 27462111 | 28631116 | 29275264 | 29852851 |
| 19647246 | 20965087 | 22218466 | 22914995 | 23750640 | 25502195 | 27462309 | 28631546 | 29275274 | 29852938 |
| 19647618 | 20965369 | 22219411 | 22915126 | 23750822 | 25502588 | 27462311 | 28632286 | 29275275 | 29853202 |
| 19648065 | 20965953 | 22219593 | 22915156 | 23751004 | 25502784 | 27462896 | 28632329 | 29275276 | 29853292 |
| 19648797 | 20966170 | 22219871 | 22915508 | 23751055 | 25502944 | 27463037 | 28632658 | 29275279 | 29853352 |
| 19649646 | 20966276 | 22220861 | 22916295 | 23751309 | 25503528 | 27463333 | 28632718 | 29275291 | 29853388 |
| 19651304 | 20966992 | 22221408 | 22916804 | 23751722 | 25503601 | 27463995 | 28632853 | 29275303 | 29853408 |
| 19651551 | 20968475 | 22221493 | 22916951 | 23752087 | 25503764 | 27464971 | 28633073 | 29275310 | 29853496 |
| 19651831 | 20968575 | 22221631 | 22917020 | 23752108 | 25504124 | 27465492 | 28633213 | 29275320 | 29853523 |
| 19651892 | 20968723 | 22221934 | 22917057 | 23752370 | 25504582 | 27465819 | 28633249 | 29275330 | 29854156 |
| 19652053 | 20969216 | 22222428 | 22917150 | 23753058 | 25504739 | 27465952 | 28633638 | 29275331 | 29854535 |
| 19652382 | 20971187 | 22222868 | 22917597 | 23753934 | 25504844 | 27466483 | 28633898 | 29275332 | 29855040 |
| 19652616 | 20971358 | 22223044 | 22917612 | 23754442 | 25505820 | 27466654 | 28634758 | 29275337 | 29855584 |
| 19653268 | 20971378 | 22223085 | 22918021 | 23754789 | 25505831 | 27466708 | 28635032 | 29275357 | 29855735 |
| 19654432 | 20971912 | 22223455 | 22918577 | 23754913 | 25505846 | 27467306 | 28635881 | 29275365 | 29856177 |
| 19654594 | 20973229 | 22223673 | 22919006 | 23755046 | 25506711 | 27468448 | 28636287 | 29275375 | 29856904 |
| 19655031 | 20973255 | 22223977 | 22919011 | 23755394 | 25507254 | 27468652 | 28636480 | 29275392 | 29856920 |
| 19655609 | 20974141 | 22224447 | 22919090 | 23756027 | 25508478 | 27470045 | 28636569 | 29275400 | 29857399 |
| 19655612 | 20974151 | 22225220 | 22919524 | 23756268 | 25508563 | 27470057 | 28636817 | 29275406 | 29859442 |
| 19655763 | 20974472 | 22225421 | 22919529 | 23756342 | 25508805 | 27470788 | 28637582 | 29275410 | 29860011 |
| 19656200 | 20975239 | 22225569 | 22919701 | 23756843 | 25508910 | 27471028 | 28637779 | 29275429 | 29860013 |
| 19657054 | 20975308 | 22226307 | 22919973 | 23756861 | 25509323 | 27471092 | 28637849 | 29275442 | 29860579 |
| 19657353 | 20976078 | 22226379 | 22920242 | 23757092 | 25511145 | 27471715 | 28638406 | 29275481 | 29861244 |
| 19657908 | 20976244 | 22226534 | 22920791 | 23757317 | 25511872 | 27472526 | 28638568 | 29275483 | 29861407 |
| 19657925 | 20976294 | 22227530 | 22921036 | 23757331 | 25513363 | 27473040 | 28638725 | 29275500 | 29861958 |
| 19657942 | 20977035 | 22228134 | 22921162 | 23757457 | 25514225 | 27473078 | 28638861 | 29275507 | 29862121 |
| 19658169 | 20977036 | 22228203 | 22921404 | 23757935 | 25514618 | 27473230 | 28640693 | 29275518 | 29863020 |
| 19658516 | 20977659 | 22228486 | 22921661 | 23758025 | 25515266 | 27474682 | 28640785 | 29275522 | 29863243 |
| 19658607 | 20977762 | 22228544 | 22921689 | 23758170 | 25515424 | 27475772 | 28641997 | 29275528 | 29863798 |
| 19659235 | 20978844 | 22228783 | 22921702 | 23758372 | 25516660 | 27475794 | 28642223 | 29275547 | 29864076 |
| 19659288 | 20979881 | 22229055 | 22921719 | 23759025 | 25516764 | 27476296 | 28642844 | 29275572 | 29864142 |
| 19659318 | 20980062 | 22229100 | 22921814 | 23759045 | 25516964 | 27477234 | 28643719 | 29275575 | 29865588 |
| 19659687 | 20980602 | 22229340 | 22921873 | 23759131 | 25517183 | 27477452 | 28644399 | 29275585 | 29867385 |
| 19660103 | 20981363 | 22229584 | 22922272 | 23759570 | 25518310 | 27477731 | 28644819 | 29275587 | 29867645 |
| 19660225 | 20982046 | 22229972 | 22922277 | 23759703 | 25521448 | 27477874 | 28645324 | 29275599 | 29867665 |
| 19660528 | 20982533 | 22230113 | 22922611 | 23759892 | 25521549 | 27478122 | 28645444 | 29275605 | 29868412 |
| 19660806 | 20983301 | 22230763 | 22922649 | 23760259 | 25522077 | 27478638 | 28646146 | 29275609 | 29868778 |
| 19660878 | 20983999 | 22230861 | 22922971 | 23760739 | 25523696 | 27479628 | 28646354 | 29275638 | 29869718 |
| 19661020 | 20984034 | 22230867 | 22923234 | 23761044 | 25523831 | 27480480 | 28646439 | 29275641 | 29869741 |
| 19661597 | 20984556 | 22230932 | 22923472 | 23761095 | 25524633 | 27480782 | 28646525 | 29275643 | 29869854 |
| 19661768 | 20985616 | 22231625 | 22923666 | 23761982 | 25525080 | 27480888 | 28647340 | 29275661 | 29869935 |
| 19661980 | 20985951 | 22231889 | 22923814 | 23762021 | 25525252 | 27481007 | 28647446 | 29275668 | 29870357 |
| 19662414 | 20987968 | 22232919 | 22923952 | 23762429 | 25531747 | 27481365 | 28648602 | 29275712 | 29870831 |
| 19662820 | 20988817 | 22233158 | 22923954 | 23762983 | 25531830 | 27481547 | 28648733 | 29275715 | 29871338 |
| 19662829 | 20989057 | 22233208 | 22923973 | 23765445 | 25532215 | 27482059 | 28649007 | 29275723 | 29872361 |
| 19662887 | 20989534 | 22233561 | 22924218 | 23765455 | 25532545 | 27482329 | 28649943 | 29275736 | 29872441 |
| 19662925 | 20989759 | 22233595 | 22924503 | 23765537 | 25533577 | 27482628 | 28650139 | 29275739 | 29872876 |
| 19663029 | 20990078 | 22233657 | 22924546 | 23765586 | 25533676 | 27483917 | 28650219 | 29275748 | 29872971 |
| 19663337 | 20990859 | 22233804 | 22924938 | 23766140 | 25534051 | 27483954 | 28650723 | 29275750 | 29873778 |
| 19663741 | 20991803 | 22234020 | 22925288 | 23766711 | 25534756 | 27485368 | 28651209 | 29275764 | 29873898 |
| 19663914 | 20995176 | 22234145 | 22925539 | 23767365 | 25535382 | 27485420 | 28651550 | 29275775 | 29874202 |
| 19664003 | 20995244 | 22234254 | 22925972 | 23768565 | 25536277 | 27485439 | 28651616 | 29275785 | 29874399 |
| 19665263 | 20995415 | 22234304 | 22926546 | 23769387 | 25537150 | 27485569 | 28651737 | 29275788 | 29875211 |
| 19665360 | 20995897 | 22235043 | 22926608 | 23769401 | 25537930 | 27486734 | 28652672 | 29275790 | 29875698 |
| 19665396 | 20995956 | 22235141 | 22926835 | 23769707 | 25538419 | 27487714 | 28652786 | 29275793 | 29875921 |
| 19665934 | 20996369 | 22235212 | 22926921 | 23769829 | 25538657 | 27487842 | 28653409 | 29275806 | 29876554 |
| 19665943 | 20997305 | 22235634 | 22926987 | 23770172 | 25538719 | 27488196 | 28653802 | 29275808 | 29877198 |
| 19665997 | 20999075 | 22235746 | 22927003 | 23770776 | 25540091 | 27488542 | 28653848 | 29275811 | 29877309 |
| 19666545 | 20999214 | 22235906 | 22927107 | 23771049 | 25540238 | 27488768 | 28653921 | 29275812 | 29877401 |
| 19666620 | 21001476 | 22236056 | 22927214 | 23772157 | 25540634 | 27489473 | 28654553 | 29275836 | 29877415 |
| 19666914 | 21001477 | 22236627 | 22927250 | 23772161 | 25541952 | 27489622 | 28655484 | 29275842 | 29877453 |
| 19667022 | 21002376 | 22236661 | 22927735 | 23773051 | 25544487 | 27489685 | 28655790 | 29275858 | 29878746 |
| 19667537 | 21003182 | 22236694 | 22928193 | 23773380 | 25544878 | 27489691 | 28655794 | 29275868 | 29879213 |
| 19667670 | 21003428 | 22236755 | 22928221 | 23773505 | 25545886 | 27489985 | 28656247 | 29275873 | 29879391 |
| 19667734 | 21003960 | 22236762 | 22928384 | 23773850 | 25547340 | 27490219 | 28656253 | 29275883 | 29879596 |
| 19667932 | 21004602 | 22236929 | 22928410 | 23774018 | 25548730 | 27490999 | 28656873 | 29275906 | 29879835 |
| 19667949 | 21005710 | 22237069 | 22928719 | 23774042 | 25549927 | 27491632 | 28656902 | 29275907 | 29880119 |
| 19668071 | 21007206 | 22237455 | 22929145 | 23774571 | 25551316 | 27491843 | 28658396 | 29275908 | 29880482 |
| 19668280 | 21007955 | 22237537 | 22929259 | 23774688 | 25551365 | 27492067 | 28658719 | 29275913 | 29880626 |
| 19668345 | 21008094 | 22237802 | 22929571 | 23775144 | 25552936 | 27492923 | 28659428 | 29275931 | 29881585 |
| 19668434 | 21008766 | 22237810 | 22929699 | 23775778 | 25553299 | 27493234 | 28660103 | 29275945 | 29881841 |
| 19668779 | 21008779 | 22237898 | 22930300 | 23775912 | 25553709 | 27494442 | 28660584 | 29275947 | 29882314 |
| 19669129 | 21010386 | 22238023 | 22930595 | 23776197 | 25554881 | 27495570 | 28660849 | 29275959 | 29882391 |
| 19669788 | 21010984 | 22238347 | 22931355 | 23776627 | 25555490 | 27495594 | 28661601 | 29275962 | 29882406 |
| 19669913 | 21011660 | 22238440 | 22931373 | 23776754 | 25555883 | 27496231 | 28661870 | 29275968 | 29883541 |
| 19670077 | 21011731 | 22238476 | 22931685 | 23776766 | 25557025 | 27496689 | 28662688 | 29275977 | 29883642 |
| 19670219 | 21011879 | 22238593 | 22931712 | 23777048 | 25557397 | 27497090 | 28663149 | 29275985 | 29883884 |
| 19670261 | 21011980 | 22238722 | 22932031 | 23778224 | 25558144 | 27497785 | 28663572 | 29275988 | 29884127 |
| 19670930 | 21012293 | 22238906 | 22932291 | 23778681 | 25559088 | 27498009 | 28663814 | 29275999 | 29884497 |
| 19671084 | 21012512 | 22239074 | 22932641 | 23779067 | 25559571 | 27498312 | 28663953 | 29276003 | 29885121 |
| 19671766 | 21013158 | 22239266 | 22932729 | 23779208 | 25560593 | 27498349 | 28665168 | 29276010 | 29886072 |
| 19672098 | 21013419 | 22239521 | 22932856 | 23779405 | 25560763 | 27498833 | 28665845 | 29276012 | 29886444 |
| 19672271 | 21013594 | 22239625 | 22933019 | 23779711 | 25561328 | 27498872 | 28666831 | 29276026 | 29886663 |
| 19672646 | 21013618 | 22239728 | 22933405 | 23780442 | 25561550 | 27499119 | 28667031 | 29276044 | 29887244 |
| 19672839 | 21013883 | 22239749 | 22933671 | 23781002 | 25562047 | 27499754 | 28667516 | 29276060 | 29888180 |
| 19672981 | 21014240 | 22239799 | 22934154 | 23781182 | 25562651 | 27500680 | 28667734 | 29276064 | 29888347 |
| 19673091 | 21014387 | 22240440 | 22934207 | 23781229 | 25565255 | 27501037 | 28667783 | 29276065 | 29888393 |
| 19673540 | 21014674 | 22240534 | 22934548 | 23781693 | 25565659 | 27502210 | 28668444 | 29276066 | 29889802 |
| 19673890 | 21015127 | 22240731 | 22934690 | 23781834 | 25566936 | 27502286 | 28669190 | 29276069 | 29890215 |
| 19674180 | 21016073 | 22242149 | 22935159 | 23781991 | 25567358 | 27503035 | 28669926 | 29276081 | 29891940 |
| 19674399 | 21016341 | 22242336 | 22935499 | 23782287 | 25567391 | 27503578 | 28669950 | 29276096 | 29892740 |
| 19674542 | 21016470 | 22243006 | 22935699 | 23782472 | 25567794 | 27504732 | 28670560 | 29276104 | 29892806 |
| 19675303 | 21018391 | 22243178 | 22935734 | 23782826 | 25569475 | 27504850 | 28671726 | 29276109 | 29892929 |
| 19675321 | 21018691 | 22243750 | 22935766 | 23782833 | 25571764 | 27506108 | 28672060 | 29276115 | 29893427 |
| 19675933 | 21020252 | 22243861 | 22935804 | 23784400 | 25574515 | 27506540 | 28672264 | 29276124 | 29893524 |
| 19676812 | 21020351 | 22244299 | 22936289 | 23784491 | 25575165 | 27507817 | 28672290 | 29276132 | 29893755 |
| 19676847 | 21020586 | 22244846 | 22936299 | 23784936 | 25576557 | 27508285 | 28673552 | 29276134 | 29893764 |
| 19677198 | 21021335 | 22245377 | 22936470 | 23785041 | 25576780 | 27508935 | 28673599 | 29276140 | 29893940 |
| 19677399 | 21021946 | 22245593 | 22936729 | 23785175 | 25577750 | 27509088 | 28674225 | 29276145 | 29894064 |
| 19677627 | 21021951 | 22245965 | 22937485 | 23785970 | 25578377 | 27509841 | 28674258 | 29276170 | 29896008 |
| 19678004 | 21022589 | 22246165 | 22937560 | 23786143 | 25578678 | 27510430 | 28674743 | 29276174 | 29896655 |
| 19678330 | 21023334 | 22246331 | 22937900 | 23786566 | 25579173 | 27510516 | 28674912 | 29276175 | 29897320 |
| 19678874 | 21023427 | 22246546 | 22937944 | 23786708 | 25579195 | 27510829 | 28674940 | 29276180 | 29897729 |
| 19679307 | 21023434 | 22246691 | 22938000 | 23787946 | 25579698 | 27510965 | 28675110 | 29276190 | 29898754 |
| 19680616 | 21023704 | 22246972 | 22938189 | 23788004 | 25580603 | 27511139 | 28675484 | 29276195 | 29899029 |
| 19680713 | 21023897 | 22247362 | 22938230 | 23788362 | 25581412 | 27511496 | 28675526 | 29276205 | 29899383 |
| 19680921 | 21023919 | 22247468 | 22938399 | 23788375 | 25582712 | 27511641 | 28676510 | 29276210 | 29899508 |
| 19681488 | 21024682 | 22247498 | 22938669 | 23788746 | 25583992 | 27511805 | 28677094 | 29276223 | 29899837 |
| 19681892 | 21025042 | 22248040 | 22938992 | 23789594 | 25584363 | 27512378 | 28677643 | 29276235 | 29900100 |
| 19682138 | 21025241 | 22248638 | 22939238 | 23789752 | 25585937 | 27513372 | 28678658 | 29276248 | 29901939 |
| 19682169 | 21026069 | 22248702 | 22939361 | 23789770 | 25585938 | 27513449 | 28678724 | 29276259 | 29901974 |
| 19682984 | 21026297 | 22248712 | 22939426 | 23789865 | 25586339 | 27513572 | 28679218 | 29276260 | 29902218 |
| 19683382 | 21026415 | 22248828 | 22939928 | 23790081 | 25586455 | 27514490 | 28679503 | 29276263 | 29902589 |
| 19683694 | 21026853 | 22248923 | 22940325 | 23790225 | 25586773 | 27515484 | 28679982 | 29276294 | 29902928 |
| 19684435 | 21027145 | 22249022 | 22940380 | 23790406 | 25587419 | 27515590 | 28680052 | 29276296 | 29903141 |
| 19684585 | 21027989 | 22249060 | 22940665 | 23790676 | 25587434 | 27515769 | 28680737 | 29276320 | 29903436 |
| 19685506 | 21028724 | 22249437 | 22940781 | 23790914 | 25589459 | 27515806 | 28681380 | 29276323 | 29903771 |
| 19685887 | 21029115 | 22249637 | 22941197 | 23791332 | 25589748 | 27516901 | 28682171 | 29276327 | 29903841 |
| 19686087 | 21029705 | 22249698 | 22941271 | 23791557 | 25590197 | 27517786 | 28682554 | 29276333 | 29904866 |
| 19686881 | 21029835 | 22249789 | 22941603 | 23791694 | 25590365 | 27518161 | 28683652 | 29276341 | 29905670 |
| 19687338 | 21031014 | 22250046 | 22942322 | 23793621 | 25591703 | 27518381 | 28683735 | 29276348 | 29905848 |
| 19687396 | 21031342 | 22251480 | 22942454 | 23794010 | 25593065 | 27518636 | 28683855 | 29276359 | 29906471 |
| 19687620 | 21031469 | 22251527 | 22942618 | 23794178 | 25593212 | 27519022 | 28684320 | 29276363 | 29906547 |
| 19687736 | 21031752 | 22251686 | 22942683 | 23794477 | 25593340 | 27519447 | 28684346 | 29276369 | 29906806 |
| 19687899 | 21032390 | 22251772 | 22942723 | 23795305 | 25593790 | 27519456 | 28684419 | 29276385 | 29906861 |
| 19688609 | 21033261 | 22252451 | 22942976 | 23795572 | 25594676 | 27519861 | 28684829 | 29276386 | 29907003 |
| 19688617 | 21033615 | 22252778 | 22943628 | 23795869 | 25594846 | 27519939 | 28685189 | 29276388 | 29907536 |
| 19688921 | 21035914 | 22253430 | 22943793 | 23796839 | 25595674 | 27520777 | 28685375 | 29276390 | 29907750 |
| 19688982 | 21036302 | 22253598 | 22943867 | 23796918 | 25597200 | 27521300 | 28685610 | 29276396 | 29907782 |
| 19689467 | 21036651 | 22253848 | 22943887 | 23797143 | 25597519 | 27521527 | 28685723 | 29276400 | 29908229 |
| 19689645 | 21036774 | 22253850 | 22944091 | 23798369 | 25597617 | 27521732 | 28686518 | 29276402 | 29909066 |
| 19689719 | 21039743 | 22254221 | 22944130 | 23798664 | 25598113 | 27521735 | 28686732 | 29276414 | 29909498 |
| 19689834 | 21039782 | 22254567 | 22944402 | 23798685 | 25599319 | 27522104 | 28686885 | 29276417 | 29909674 |
| 19690036 | 21040089 | 22254592 | 22944760 | 23799508 | 25599554 | 27522137 | 28687317 | 29276426 | 29909925 |
| 19690414 | 21041523 | 22254636 | 22944897 | 23799687 | 25599741 | 27524293 | 28687372 | 29276430 | 29911038 |
| 19690487 | 21042364 | 22254842 | 22944983 | 23799701 | 25605892 | 27524326 | 28687438 | 29276435 | 29911211 |
| 19690575 | 21045444 | 22255232 | 22945295 | 23801093 | 25606753 | 27524542 | 28687546 | 29276437 | 29911394 |
| 19690681 | 21045975 | 22255333 | 22945566 | 23801267 | 25608069 | 27524673 | 28687883 | 29276439 | 29911972 |
| 19690712 | 21047111 | 22255678 | 22945706 | 23801694 | 25608104 | 27526040 | 28688504 | 29276440 | 29913126 |
| 19691197 | 21047242 | 22255758 | 22946471 | 23802658 | 25608550 | 27526726 | 28688985 | 29276445 | 29913301 |
| 19691508 | 21047329 | 22255892 | 22946487 | 23802833 | 25609036 | 27527134 | 28689298 | 29276477 | 29913788 |
| 19691702 | 21048249 | 22256096 | 22946580 | 23803188 | 25610063 | 27528177 | 28689344 | 29276488 | 29913938 |
| 19692396 | 21048340 | 22256254 | 22946939 | 23803442 | 25610287 | 27528724 | 28689634 | 29276489 | 29914366 |
| 19692495 | 21048446 | 22256321 | 22947400 | 23803473 | 25610583 | 27529681 | 28690039 | 29276491 | 29915279 |
| 19692517 | 21049511 | 22257726 | 22947563 | 23804367 | 25611637 | 27529719 | 28690867 | 29276494 | 29915421 |
| 19693262 | 21050308 | 22257802 | 22947584 | 23805048 | 25614732 | 27530321 | 28692051 | 29276503 | 29915517 |
| 19693378 | 21050818 | 22257934 | 22947878 | 23805309 | 25616106 | 27530869 | 28692209 | 29276505 | 29916134 |
| 19693470 | 21051189 | 22257943 | 22948275 | 23805540 | 25616488 | 27531633 | 28693140 | 29276531 | 29916421 |
| 19695008 | 21051379 | 22258077 | 22948285 | 23805674 | 25618350 | 27531919 | 28693162 | 29276537 | 29916794 |
| 19695215 | 21051481 | 22258105 | 22948356 | 23805900 | 25618758 | 27532053 | 28694221 | 29276538 | 29916929 |
| 19695377 | 21051559 | 22258913 | 22948596 | 23806420 | 25619243 | 27532300 | 28694281 | 29276552 | 29917254 |
| 19695935 | 21051897 | 22259180 | 22948765 | 23806801 | 25621136 | 27532419 | 28694385 | 29276555 | 29917372 |
| 19696606 | 21052646 | 22259263 | 22948791 | 23807211 | 25621589 | 27532421 | 28694731 | 29276560 | 29917390 |
| 19696670 | 21053084 | 22259477 | 22949171 | 23807259 | 25622562 | 27532468 | 28695125 | 29276562 | 29917391 |
| 19697360 | 21053567 | 22259503 | 22949324 | 23807359 | 25622822 | 27533680 | 28695663 | 29276565 | 29917648 |
| 19698042 | 21053815 | 22259759 | 22949442 | 23807431 | 25622967 | 27534120 | 28695671 | 29276567 | 29918081 |
| 19698052 | 21053969 | 22259985 | 22949529 | 23808218 | 25624279 | 27534309 | 28696698 | 29276568 | 29918599 |
| 19698540 | 21054535 | 22260422 | 22949664 | 23809025 | 25624354 | 27534983 | 28696826 | 29276576 | 29919603 |
| 19698580 | 21055032 | 22260647 | 22949686 | 23809326 | 25625610 | 27536065 | 28697051 | 29276583 | 29919829 |
| 19699530 | 21055322 | 22261804 | 22949723 | 23809413 | 25626440 | 27536076 | 28697385 | 29276588 | 29920109 |
| 19699549 | 21055524 | 22261889 | 22949780 | 23809441 | 25626580 | 27536441 | 28698885 | 29276589 | 29920264 |
| 19700174 | 21056901 | 22261947 | 22950467 | 23811203 | 25628812 | 27536485 | 28698982 | 29276632 | 29922045 |
| 19700572 | 21056973 | 22262154 | 22950524 | 23811847 | 25630681 | 27537174 | 28699074 | 29276638 | 29922097 |
| 19700804 | 21057214 | 22262438 | 22951457 | 23811930 | 25631045 | 27537459 | 28699823 | 29276659 | 29922521 |
| 19701370 | 21058178 | 22262707 | 22951565 | 23812018 | 25633052 | 27537601 | 28699934 | 29276662 | 29923267 |
| 19701991 | 21058362 | 22262895 | 22951630 | 23812308 | 25634974 | 27538015 | 28700364 | 29276670 | 29926934 |
| 19702731 | 21058367 | 22263090 | 22951681 | 23813426 | 25635220 | 27538466 | 28700667 | 29276673 | 29928229 |
| 19702958 | 21060392 | 22263208 | 22951824 | 23813530 | 25635232 | 27539614 | 28700707 | 29276694 | 29928796 |
| 19703510 | 21061313 | 22263298 | 22952252 | 23813664 | 25636290 | 27539730 | 28701102 | 29276700 | 29929487 |
| 19703603 | 21061871 | 22263353 | 22952307 | 23814969 | 25636679 | 27539776 | 28701395 | 29276706 | 29930335 |
| 19703616 | 21062533 | 22263433 | 22952335 | 23815130 | 25636782 | 27539939 | 28701542 | 29276712 | 29930371 |
| 19704166 | 21062632 | 22263483 | 22952746 | 23815350 | 25637009 | 27540133 | 28701919 | 29276718 | 29930733 |
| 19704563 | 21063487 | 22263716 | 22952983 | 23816060 | 25639433 | 27540541 | 28702305 | 29276720 | 29931243 |
| 19704680 | 21063879 | 22264052 | 22953699 | 23816550 | 25639697 | 27540737 | 28702322 | 29276722 | 29931750 |
| 19705438 | 21063985 | 22264671 | 22953805 | 23816702 | 25640460 | 27541636 | 28702641 | 29276731 | 29931770 |
| 19706123 | 21064010 | 22264734 | 22954196 | 23817489 | 25640986 | 27541782 | 28702645 | 29276736 | 29934217 |
| 19706439 | 21064234 | 22265042 | 22954533 | 23817732 | 25641089 | 27542082 | 28703118 | 29276737 | 29934246 |
| 19706794 | 21065072 | 22265062 | 22954684 | 23818018 | 25644215 | 27542609 | 28703334 | 29276752 | 29934621 |
| 19706842 | 21065118 | 22265235 | 22955025 | 23818447 | 25644320 | 27543142 | 28703340 | 29276754 | 29934946 |
| 19706973 | 21065898 | 22266179 | 22955125 | 23819228 | 25644350 | 27543223 | 28703587 | 29276760 | 29935002 |
| 19707173 | 21066256 | 22266204 | 22955645 | 23819392 | 25644414 | 27543264 | 28704048 | 29276764 | 29936464 |
| 19707769 | 21067009 | 22266243 | 22955709 | 23819502 | 25644823 | 27543310 | 28704137 | 29276782 | 29936529 |
| 19708203 | 21067554 | 22266245 | 22955720 | 23819503 | 25645120 | 27543328 | 28706091 | 29276785 | 29937387 |
| 19708738 | 21068236 | 22266327 | 22956081 | 23820147 | 25646259 | 27543519 | 28706134 | 29276791 | 29938036 |
| 19709193 | 21068744 | 22266354 | 22956359 | 23820325 | 25646710 | 27543710 | 28706998 | 29276794 | 29938481 |
| 19709306 | 21070536 | 22266372 | 22956601 | 23820651 | 25646751 | 27544104 | 28707062 | 29276811 | 29938537 |
| 19709400 | 21071351 | 22266450 | 22957532 | 23820775 | 25647143 | 27544182 | 28708392 | 29276816 | 29938567 |
| 19709406 | 21072171 | 22266454 | 22958096 | 23822441 | 25647153 | 27544227 | 28708784 | 29276829 | 29939868 |
| 19710848 | 21072838 | 22266758 | 22958123 | 23822782 | 25647611 | 27544271 | 28708922 | 29276843 | 29939873 |
| 19710863 | 21073433 | 22266817 | 22958262 | 23822851 | 25647830 | 27545938 | 28708995 | 29276860 | 29940078 |
| 19711070 | 21073740 | 22267014 | 22958441 | 23824638 | 25648123 | 27546363 | 28709300 | 29276872 | 29940193 |
| 19711791 | 21074014 | 22267265 | 22959082 | 23825305 | 25648395 | 27547160 | 28710028 | 29276878 | 29940515 |
| 19712191 | 21074204 | 22267649 | 22959157 | 23825530 | 25649102 | 27547876 | 28710208 | 29276880 | 29940887 |
| 19712278 | 21074397 | 22267910 | 22959166 | 23826043 | 25649978 | 27548289 | 28710806 | 29276900 | 29941384 |
| 19712589 | 21074442 | 22267980 | 22959509 | 23826327 | 25650709 | 27548630 | 28711046 | 29276909 | 29942321 |
| 19712799 | 21075199 | 22268205 | 22959560 | 23826354 | 25650962 | 27549163 | 28711048 | 29276922 | 29942500 |
| 19713468 | 21075856 | 22268362 | 22959719 | 23826742 | 25651604 | 27549733 | 28711178 | 29276927 | 29942515 |
| 19713480 | 21075944 | 22268841 | 22959863 | 23828243 | 25651882 | 27549880 | 28711326 | 29276952 | 29943019 |
| 19713907 | 21076135 | 22269000 | 22960052 | 23828272 | 25652793 | 27550576 | 28711387 | 29276963 | 29943146 |
| 19713910 | 21076386 | 22269161 | 22960084 | 23828319 | 25653814 | 27551582 | 28711522 | 29276965 | 29943165 |
| 19714414 | 21077458 | 22269246 | 22960172 | 23829317 | 25654055 | 27551782 | 28711583 | 29276968 | 29943258 |
| 19714539 | 21077652 | 22269278 | 22960512 | 23829457 | 25654398 | 27552640 | 28712648 | 29276974 | 29943376 |
| 19715738 | 21077982 | 22270141 | 22960800 | 23829640 | 25656323 | 27552960 | 28712971 | 29276984 | 29943452 |
| 19715879 | 21078148 | 22270227 | 22960928 | 23829712 | 25657581 | 27553367 | 28713405 | 29276988 | 29943520 |
| 19716053 | 21078926 | 22270371 | 22961125 | 23829713 | 25657958 | 27553477 | 28713482 | 29276995 | 29943844 |
| 19716382 | 21079446 | 22270388 | 22961140 | 23830441 | 25657984 | 27554059 | 28713860 | 29277006 | 29944238 |
| 19716642 | 21079896 | 22270543 | 22961143 | 23830654 | 25658016 | 27554647 | 28714008 | 29277012 | 29945039 |
| 19717017 | 21080284 | 22270555 | 22961306 | 23830680 | 25660011 | 27555092 | 28714454 | 29277015 | 29945337 |
| 19717502 | 21080816 | 22270647 | 22961472 | 23830691 | 25660714 | 27555991 | 28714579 | 29277036 | 29945378 |
| 19718431 | 21080829 | 22270742 | 22961508 | 23830707 | 25661485 | 27557255 | 28716163 | 29277037 | 29945534 |
| 19718501 | 21080936 | 22271064 | 22961533 | 23830922 | 25662819 | 27557683 | 28716839 | 29277043 | 29946384 |
| 19718633 | 21081072 | 22271107 | 22961568 | 23832350 | 25664070 | 27557771 | 28717344 | 29277053 | 29946963 |
| 19718885 | 21082684 | 22271418 | 22962749 | 23832907 | 25664160 | 27558091 | 28718001 | 29277061 | 29947138 |
| 19718919 | 21083294 | 22271609 | 22963101 | 23833159 | 25664353 | 27558305 | 28718059 | 29277069 | 29947489 |
| 19719033 | 21083611 | 22271753 | 22963125 | 23833239 | 25665258 | 27558546 | 28718685 | 29277077 | 29948665 |
| 19719381 | 21084037 | 22271913 | 22963216 | 23833834 | 25665294 | 27558815 | 28718736 | 29277083 | 29948727 |
| 19720758 | 21084106 | 22272581 | 22963255 | 23834467 | 25667766 | 27559763 | 28719311 | 29277101 | 29948788 |
| 19720815 | 21084878 | 22272876 | 22963309 | 23834718 | 25668339 | 27560819 | 28719386 | 29277117 | 29948955 |
| 19721191 | 21086155 | 22272981 | 22963559 | 23834744 | 25669038 | 27561102 | 28719412 | 29277122 | 29949214 |
| 19721749 | 21086185 | 22273193 | 22963864 | 23835046 | 25669235 | 27561250 | 28719414 | 29277127 | 29949338 |
| 19722757 | 21086291 | 22274243 | 22963958 | 23836517 | 25670274 | 27561711 | 28720656 | 29277135 | 29949645 |
| 19722866 | 21087222 | 22274365 | 22964052 | 23836594 | 25674605 | 27561783 | 28721238 | 29277136 | 29949803 |
| 19722928 | 21087350 | 22274643 | 22964066 | 23837085 | 25676291 | 27562155 | 28721939 | 29277142 | 29951705 |
| 19723511 | 21089187 | 22274719 | 22964149 | 23837204 | 25679757 | 27562191 | 28722088 | 29277177 | 29951746 |
| 19723716 | 21089470 | 22275781 | 22964238 | 23837970 | 25680119 | 27562317 | 28723560 | 29277187 | 29951896 |
| 19723997 | 21089744 | 22276016 | 22964506 | 23838290 | 25681392 | 27562388 | 28723614 | 29277190 | 29952021 |
| 19724494 | 21090641 | 22276511 | 22964567 | 23838502 | 25683898 | 27562835 | 28723939 | 29277196 | 29952030 |
| 19724527 | 21090931 | 22276555 | 22964767 | 23838782 | 25684260 | 27563390 | 28724089 | 29277212 | 29952211 |
| 19725862 | 21091556 | 22276640 | 22964879 | 23839418 | 25684424 | 27563949 | 28724114 | 29277213 | 29952998 |
| 19726209 | 21092507 | 22276715 | 22964975 | 23840132 | 25686634 | 27564063 | 28724495 | 29277214 | 29953000 |
| 19726331 | 21092808 | 22276788 | 22965019 | 23841325 | 25687068 | 27564326 | 28724546 | 29277219 | 29953834 |
| 19726389 | 21093631 | 22276811 | 22965458 | 23841332 | 25687346 | 27565884 | 28725124 | 29277227 | 29954237 |
| 19726623 | 21095016 | 22276922 | 22965711 | 23841649 | 25687357 | 27566236 | 28725845 | 29277233 | 29954445 |
| 19726721 | 21095415 | 22277138 | 22966139 | 23842341 | 25687617 | 27566291 | 28726126 | 29277238 | 29954450 |
| 19726755 | 21095784 | 22277260 | 22966638 | 23842916 | 25687751 | 27566772 | 28726590 | 29277273 | 29954996 |
| 19726959 | 21096131 | 22277317 | 22966714 | 23843188 | 25687916 | 27567698 | 28726835 | 29277276 | 29955170 |
| 19727253 | 21096445 | 22277462 | 22966872 | 23843284 | 25688117 | 27568646 | 28727904 | 29277292 | 29956223 |
| 19727858 | 21096983 | 22277707 | 22966923 | 23843498 | 25688172 | 27568669 | 28728004 | 29277295 | 29956264 |
| 19728577 | 21096991 | 22277708 | 22967145 | 23843652 | 25689082 | 27568843 | 28728100 | 29277296 | 29956490 |
| 19728868 | 21097366 | 22278108 | 22967239 | 23843790 | 25689535 | 27568953 | 28728143 | 29277299 | 29956532 |
| 19728883 | 21098158 | 22278671 | 22967358 | 23844530 | 25690083 | 27569189 | 28728285 | 29277335 | 29957652 |
| 19729033 | 21099157 | 22278914 | 22968077 | 23845606 | 25690408 | 27569875 | 28729792 | 29277340 | 29959760 |
| 19729361 | 21099354 | 22279002 | 22968078 | 23846099 | 25690904 | 27570025 | 28729967 | 29277343 | 29960278 |
| 19730102 | 21099959 | 22279211 | 22968174 | 23846125 | 25695577 | 27570233 | 28731091 | 29277349 | 29960306 |
| 19730223 | 21100123 | 22279499 | 22968342 | 23846238 | 25696589 | 27570953 | 28731328 | 29277355 | 29960453 |
| 19730488 | 21100285 | 22279559 | 22968488 | 23846481 | 25696592 | 27571402 | 28731364 | 29277358 | 29961657 |
| 19730740 | 21100349 | 22279728 | 22968593 | 23846911 | 25697044 | 27571492 | 28731514 | 29277363 | 29962596 |
| 19731206 | 21100784 | 22280241 | 22968642 | 23846994 | 25697153 | 27573380 | 28731785 | 29277372 | 29963522 |
| 19732221 | 21100866 | 22280316 | 22968943 | 23847205 | 25697689 | 27573524 | 28732344 | 29277385 | 29963720 |
| 19732301 | 21102082 | 22280770 | 22969603 | 23847524 | 25698384 | 27573721 | 28732608 | 29277389 | 29964178 |
| 19733167 | 21102499 | 22280973 | 22970071 | 23847553 | 25698759 | 27574301 | 28733444 | 29277408 | 29964491 |
| 19733207 | 21102664 | 22281031 | 22970328 | 23847822 | 25698887 | 27575109 | 28733785 | 29277409 | 29965545 |
| 19733287 | 21105329 | 22281360 | 22970360 | 23848047 | 25701489 | 27576547 | 28734084 | 29277414 | 29965552 |
| 19733312 | 21105453 | 22281986 | 22970396 | 23848259 | 25702925 | 27576836 | 28734374 | 29277415 | 29965561 |
| 19733653 | 21105802 | 22282280 | 22970648 | 23848309 | 25705276 | 27578258 | 28734648 | 29277420 | 29965661 |
| 19733776 | 21106259 | 22282364 | 22970917 | 23848425 | 25706051 | 27579370 | 28735022 | 29277426 | 29966479 |
| 19734440 | 21106732 | 22282622 | 22971145 | 23848839 | 25707234 | 27579989 | 28735717 | 29277442 | 29967062 |
| 19734464 | 21107183 | 22282856 | 22971192 | 23849186 | 25709826 | 27580951 | 28735911 | 29277446 | 29967067 |
| 19734558 | 21107516 | 22282998 | 22971651 | 23849711 | 25710049 | 27580970 | 28736272 | 29277452 | 29967410 |
| 19734859 | 21107699 | 22283024 | 22971702 | 23849819 | 25710497 | 27581195 | 28736586 | 29277460 | 29969076 |
| 19735147 | 21107865 | 22283755 | 22971742 | 23849868 | 25710927 | 27581765 | 28737051 | 29277465 | 29969735 |
| 19735409 | 21107918 | 22283977 | 22972133 | 23850692 | 25712005 | 27581851 | 28737126 | 29277494 | 29969858 |
| 19735572 | 21108221 | 22283981 | 22972332 | 23850958 | 25714050 | 27582284 | 28737978 | 29277495 | 29970015 |
| 19735717 | 21109166 | 22284302 | 22972422 | 23851206 | 25714064 | 27582294 | 28738577 | 29277497 | 29970221 |
| 19735785 | 21111330 | 22284730 | 22972470 | 23852283 | 25715277 | 27582658 | 28738781 | 29277510 | 29970614 |
| 19735925 | 21114367 | 22284863 | 22972865 | 23852957 | 25715855 | 27583117 | 28739246 | 29277513 | 29970875 |
| 19736050 | 21114407 | 22284884 | 22972963 | 23853121 | 25716409 | 27583364 | 28739730 | 29277515 | 29971078 |
| 19736585 | 21115027 | 22284991 | 22973193 | 23853457 | 25716789 | 27583487 | 28740614 | 29277521 | 29971240 |
| 19737075 | 21115079 | 22285175 | 22973501 | 23853498 | 25717611 | 27583567 | 28740644 | 29277525 | 29971425 |
| 19737149 | 21115767 | 22285204 | 22973770 | 23853878 | 25718020 | 27583907 | 28740684 | 29277527 | 29971908 |
| 19737178 | 21115922 | 22285347 | 22973775 | 23854064 | 25718096 | 27585309 | 28741024 | 29277539 | 29972817 |
| 19737519 | 21116143 | 22285792 | 22973793 | 23854163 | 25718909 | 27585655 | 28741563 | 29277541 | 29973112 |
| 19737581 | 21116162 | 22285975 | 22974119 | 23854901 | 25719542 | 27588409 | 28741784 | 29277544 | 29973727 |
| 19737689 | 21117130 | 22285984 | 22974139 | 23855168 | 25719899 | 27588515 | 28742308 | 29277548 | 29973948 |
| 19738098 | 21117312 | 22286776 | 22974332 | 23855350 | 25721014 | 27588676 | 28742837 | 29277568 | 29974018 |
| 19738237 | 21117399 | 22286800 | 22974388 | 23855368 | 25721561 | 27589493 | 28743669 | 29277584 | 29974826 |
| 19738876 | 21117462 | 22287462 | 22974436 | 23855409 | 25722014 | 27590176 | 28744018 | 29277592 | 29974967 |
| 19739248 | 21117830 | 22287868 | 22974646 | 23856816 | 25722798 | 27590874 | 28744189 | 29277593 | 29975054 |
| 19739505 | 21118013 | 22288086 | 22974713 | 23857046 | 25724148 | 27590981 | 28745335 | 29277597 | 29975643 |
| 19739588 | 21118492 | 22288170 | 22975051 | 23857057 | 25725086 | 27590992 | 28745432 | 29277598 | 29975653 |
| 19739924 | 21118513 | 22288622 | 22975370 | 23857523 | 25725270 | 27591114 | 28745554 | 29277618 | 29976557 |
| 19740166 | 21118671 | 22288695 | 22975525 | 23857579 | 25725763 | 27591337 | 28745617 | 29277622 | 29976644 |
| 19740273 | 21119129 | 22288763 | 22975688 | 23857654 | 25725765 | 27591528 | 28745881 | 29277643 | 29976912 |
| 19741210 | 21119547 | 22288989 | 22975827 | 23857795 | 25726833 | 27591684 | 28745999 | 29277662 | 29977223 |
| 19741583 | 21119856 | 22289173 | 22975945 | 23857881 | 25727950 | 27592026 | 28746003 | 29277679 | 29977775 |
| 19741694 | 21121253 | 22289236 | 22976058 | 23857970 | 25728006 | 27592281 | 28746147 | 29277680 | 29979003 |
| 19741928 | 21122015 | 22289265 | 22976477 | 23858759 | 25729867 | 27592745 | 28747017 | 29277681 | 29979010 |
| 19741934 | 21122046 | 22289279 | 22976770 | 23859211 | 25730274 | 27592783 | 28747954 | 29277694 | 29980314 |
| 19741950 | 21122958 | 22289475 | 22976821 | 23859796 | 25730481 | 27592884 | 28748088 | 29277698 | 29980721 |
| 19742003 | 21123245 | 22289785 | 22976869 | 23859966 | 25731603 | 27592912 | 28748376 | 29277708 | 29981084 |
| 19742206 | 21123486 | 22289828 | 22976937 | 23861030 | 25733642 | 27593956 | 28748626 | 29277722 | 29981187 |
| 19742209 | 21123591 | 22290430 | 22977193 | 23861359 | 25734090 | 27594008 | 28748643 | 29277724 | 29981490 |
| 19742308 | 21123928 | 22290711 | 22977223 | 23861498 | 25734206 | 27594102 | 28748885 | 29277732 | 29981777 |
| 19743178 | 21124214 | 22290749 | 22977342 | 23861760 | 25735616 | 27594718 | 28748907 | 29277744 | 29982269 |
| 19743281 | 21126151 | 22290987 | 22977610 | 23862111 | 25737378 | 27595070 | 28749056 | 29277750 | 29982845 |
| 19744262 | 21126360 | 22291509 | 22977790 | 23862357 | 25737622 | 27596329 | 28749069 | 29277761 | 29982914 |
| 19744739 | 21126418 | 22291733 | 22977968 | 23862993 | 25737853 | 27597108 | 28750305 | 29277769 | 29983822 |
| 19744941 | 21127621 | 22291860 | 22978028 | 23863082 | 25738714 | 27597134 | 28750461 | 29277773 | 29985242 |
| 19745132 | 21127877 | 22291902 | 22978671 | 23863670 | 25739878 | 27597729 | 28750949 | 29277779 | 29985393 |
| 19745183 | 21128619 | 22292000 | 22978728 | 23864259 | 25740908 | 27598436 | 28751484 | 29277791 | 29985448 |
| 19745288 | 21128631 | 22292006 | 22979035 | 23864299 | 25741101 | 27598850 | 28751941 | 29277797 | 29986267 |
| 19745368 | 21130063 | 22292208 | 22979339 | 23864601 | 25741400 | 27598864 | 28752083 | 29277819 | 29986478 |
| 19745494 | 21130350 | 22292629 | 22980151 | 23864959 | 25743483 | 27599146 | 28752622 | 29277820 | 29986923 |
| 19746231 | 21130400 | 22293186 | 22980161 | 23866093 | 25744206 | 27599593 | 28752937 | 29277835 | 29987399 |
| 19746347 | 21130482 | 22293298 | 22980251 | 23866994 | 25744352 | 27600004 | 28753086 | 29277840 | 29988240 |
| 19746542 | 21131046 | 22293302 | 22980318 | 23867169 | 25744484 | 27600235 | 28753320 | 29277852 | 29988999 |
| 19746648 | 21131297 | 22293518 | 22980594 | 23867740 | 25744745 | 27600501 | 28753380 | 29277860 | 29989557 |
| 19746714 | 21133670 | 22293654 | 22980755 | 23867817 | 25746682 | 27600922 | 28753393 | 29277877 | 29989831 |
| 19746864 | 21133671 | 22293798 | 22980872 | 23868270 | 25750131 | 27601214 | 28753793 | 29277884 | 29990388 |
| 19747016 | 21133873 | 22294262 | 22981006 | 23868680 | 25750533 | 27601692 | 28754985 | 29277890 | 29991340 |
| 19747202 | 21133886 | 22294581 | 22981105 | 23869117 | 25752180 | 27601725 | 28755118 | 29277903 | 29991543 |
| 19747315 | 21135375 | 22294718 | 22981272 | 23869825 | 25752950 | 27602033 | 28756191 | 29277905 | 29991645 |
| 19747385 | 21135734 | 22294843 | 22981481 | 23870318 | 25753236 | 27603033 | 28756650 | 29277913 | 29991766 |
| 19747922 | 21135770 | 22295982 | 22981565 | 23871579 | 25754250 | 27603155 | 28756821 | 29277923 | 29992300 |
| 19748585 | 21136130 | 22296233 | 22981677 | 23871936 | 25754947 | 27603658 | 28757075 | 29277956 | 29992457 |
| 19748735 | 21136690 | 22296922 | 22982241 | 23872018 | 25755648 | 27603734 | 28757284 | 29277957 | 29992627 |
| 19748886 | 21136870 | 22296973 | 22982423 | 23872159 | 25756636 | 27603994 | 28757480 | 29277959 | 29993830 |
| 19749038 | 21138156 | 22296992 | 22982458 | 23872460 | 25757599 | 27604046 | 28757791 | 29277960 | 29995764 |
| 19750104 | 21138440 | 22297989 | 22982473 | 23872830 | 25758199 | 27605057 | 28757819 | 29277979 | 29995856 |
| 19750168 | 21138694 | 22298370 | 22982576 | 23872935 | 25758222 | 27605610 | 28758382 | 29277980 | 29996065 |
| 19750272 | 21138836 | 22299247 | 22982608 | 23874525 | 25758709 | 27605612 | 28758601 | 29277981 | 29997137 |
| 19751577 | 21139078 | 22299743 | 22983104 | 23875626 | 25758876 | 27605659 | 28758608 | 29277986 | 29997718 |
| 19751856 | 21140535 | 22299980 | 22983154 | 23876148 | 25759541 | 27606377 | 28758825 | 29277987 | 29998153 |
| 19751987 | 21140619 | 22300003 | 22983368 | 23876172 | 25760291 | 27606762 | 28759111 | 29278009 | 29998320 |
| 19752075 | 21141581 | 22300006 | 22983443 | 23876351 | 25760924 | 27606899 | 28759252 | 29278011 | 29999015 |
| 19752405 | 21141757 | 22300754 | 22983716 | 23876413 | 25761149 | 27607693 | 28760317 | 29278029 | 29999155 |
| 19752479 | 21142016 | 22301002 | 22983886 | 23876573 | 25762044 | 27608887 | 28760354 | 29278030 | 30000230 |
| 19752788 | 21142079 | 22301705 | 22984187 | 23876614 | 25765502 | 27608933 | 28760414 | 29278032 | 30000411 |
| 19753219 | 21142537 | 22301992 | 22985130 | 23877230 | 25768812 | 27610265 | 28760578 | 29278042 | 30000460 |
| 19754159 | 21142582 | 22302519 | 22985714 | 23877598 | 25769409 | 27610415 | 28761241 | 29278052 | 30000670 |
| 19754296 | 21142981 | 22302535 | 22985786 | 23877792 | 25769483 | 27610912 | 28761266 | 29278058 | 30000773 |
| 19754298 | 21143106 | 22302623 | 22985948 | 23877992 | 25769950 | 27610948 | 28762952 | 29278066 | 30000894 |
| 19755396 | 21143580 | 22302852 | 22986152 | 23878134 | 25772178 | 27611079 | 28763045 | 29278077 | 30001054 |
| 19755577 | 21143729 | 22303003 | 22986385 | 23878613 | 25775160 | 27611833 | 28763058 | 29278081 | 30002120 |
| 19755726 | 21143875 | 22304030 | 22986457 | 23878665 | 25775556 | 27611874 | 28763818 | 29278085 | 30002454 |
| 19756035 | 21144472 | 22304038 | 22986821 | 23879199 | 25775789 | 27612046 | 28764721 | 29278093 | 30003092 |
| 19756054 | 21144778 | 22304899 | 22986839 | 23882228 | 25776353 | 27612477 | 28764811 | 29278095 | 30003508 |
| 19756066 | 21144984 | 22305309 | 22987095 | 23882805 | 25776751 | 27613180 | 28764827 | 29278109 | 30003799 |
| 19756613 | 21144998 | 22305539 | 22987191 | 23882841 | 25777098 | 27613528 | 28765163 | 29278128 | 30004650 |
| 19756648 | 21145726 | 22305566 | 22987244 | 23883982 | 25777830 | 27613760 | 28765228 | 29278134 | 30004752 |
| 19757073 | 21146805 | 22305775 | 22987400 | 23884116 | 25781303 | 27614784 | 28765513 | 29278149 | 30005566 |
| 19757160 | 21147161 | 22306019 | 22987544 | 23884155 | 25782497 | 27615458 | 28765682 | 29278159 | 30006527 |
| 19757281 | 21147338 | 22306186 | 22987556 | 23884207 | 25782917 | 27615499 | 28765880 | 29278161 | 30006639 |
| 19757449 | 21147580 | 22306226 | 22988133 | 23885390 | 25784440 | 27615703 | 28766471 | 29278167 | 30007223 |
| 19757930 | 21147705 | 22306291 | 22988258 | 23885634 | 25785259 | 27616088 | 28766661 | 29278171 | 30007776 |
| 19758088 | 21148367 | 22306365 | 22988337 | 23885726 | 25785818 | 27617051 | 28767828 | 29278176 | 30008370 |
| 19758374 | 21148413 | 22306603 | 22988365 | 23885962 | 25787185 | 27617163 | 28767987 | 29278182 | 30008711 |
| 19758405 | 21149628 | 22306673 | 22988434 | 23886261 | 25787290 | 27617617 | 28768201 | 29278191 | 30009265 |
| 19759699 | 21150024 | 22306675 | 22988853 | 23886403 | 25787383 | 27617635 | 28768345 | 29278199 | 30009386 |
| 19759932 | 21150238 | 22306747 | 22989028 | 23886590 | 25788099 | 27617750 | 28768828 | 29278214 | 30009452 |
| 19760047 | 21151350 | 22306772 | 22989080 | 23886904 | 25789334 | 27618267 | 28769368 | 29278215 | 30009837 |
| 19761208 | 21151508 | 22307107 | 22989676 | 23887815 | 25789752 | 27618525 | 28769496 | 29278219 | 30009842 |
| 19761249 | 21151798 | 22307167 | 22989772 | 23887828 | 25789757 | 27618968 | 28770210 | 29278234 | 30010237 |
| 19761372 | 21151820 | 22307191 | 22989887 | 23888463 | 25789830 | 27619604 | 28770403 | 29278245 | 30011036 |
| 19762443 | 21151899 | 22307440 | 22989943 | 23888774 | 25790684 | 27619999 | 28770631 | 29278246 | 30011626 |
| 19762494 | 21152115 | 22307888 | 22990026 | 23889291 | 25791256 | 27620210 | 28770903 | 29278248 | 30011993 |
| 19762530 | 21152433 | 22308037 | 22990072 | 23890031 | 25791851 | 27620393 | 28771139 | 29278254 | 30012265 |
| 19763149 | 21152854 | 22308219 | 22990142 | 23890141 | 25792978 | 27620864 | 28771360 | 29278258 | 30012644 |
| 19763537 | 21152916 | 22308364 | 22990275 | 23890233 | 25793352 | 27621480 | 28771529 | 29278275 | 30012824 |
| 19764951 | 21153300 | 22308582 | 22990293 | 23890682 | 25794876 | 27621513 | 28771926 | 29278280 | 30013753 |
| 19765562 | 21153998 | 22308658 | 22990308 | 23891037 | 25794981 | 27621758 | 28772003 | 29278284 | 30013770 |
| 19765715 | 21156460 | 22308983 | 22990346 | 23891325 | 25796663 | 27621956 | 28772269 | 29278290 | 30014071 |
| 19766402 | 21158626 | 22309957 | 22991100 | 23893477 | 25798036 | 27623214 | 28773693 | 29278299 | 30014465 |
| 19766476 | 21159090 | 22310162 | 22991186 | 23893522 | 25798232 | 27623219 | 28773883 | 29278304 | 30014607 |
| 19766500 | 21159943 | 22310406 | 22991552 | 23894398 | 25798467 | 27623516 | 28773921 | 29278305 | 30015055 |
| 19767499 | 21160292 | 22310478 | 22991943 | 23894449 | 25799995 | 27624020 | 28773969 | 29278343 | 30016523 |
| 19767519 | 21161500 | 22311114 | 22992029 | 23894671 | 25801045 | 27625503 | 28774467 | 29278345 | 30017246 |
| 19767694 | 21161802 | 22311308 | 22992071 | 23895325 | 25801101 | 27625767 | 28774502 | 29278353 | 30017452 |
| 19767699 | 21161893 | 22311370 | 22992305 | 23895362 | 25801327 | 27626272 | 28774887 | 29278354 | 30017471 |
| 19767767 | 21162025 | 22311541 | 22992398 | 23895385 | 25802640 | 27626329 | 28774965 | 29278364 | 30017589 |
| 19767812 | 21162342 | 22311696 | 22992473 | 23896187 | 25802773 | 27626813 | 28774969 | 29278368 | 30017818 |
| 19768141 | 21162385 | 22311750 | 22992611 | 23896190 | 25803775 | 27627047 | 28774974 | 29278370 | 30018151 |
| 19768848 | 21166243 | 22311775 | 22992654 | 23897429 | 25804314 | 27627357 | 28775986 | 29278371 | 30018414 |
| 19768987 | 21167129 | 22311800 | 22992663 | 23897909 | 25804338 | 27627588 | 28776237 | 29278376 | 30018437 |
| 19769167 | 21167772 | 22311879 | 22992672 | 23898191 | 25804889 | 27627824 | 28776508 | 29278382 | 30018804 |
| 19769289 | 21167863 | 22311922 | 22992686 | 23898289 | 25804951 | 27627886 | 28776834 | 29278386 | 30019613 |
| 19769311 | 21168025 | 22312225 | 22992863 | 23898908 | 25806433 | 27628018 | 28776916 | 29278399 | 30020025 |
| 19769441 | 21168111 | 22312352 | 22992996 | 23899246 | 25810420 | 27628019 | 28777336 | 29278408 | 30020682 |
| 19769478 | 21168133 | 22312370 | 22993044 | 23899376 | 25813275 | 27628953 | 28777609 | 29278420 | 30021559 |
| 19769523 | 21168438 | 22312446 | 22993098 | 23899741 | 25815071 | 27629018 | 28777614 | 29278423 | 30022036 |
| 19769693 | 21168575 | 22312508 | 22993254 | 23900051 | 25817995 | 27629272 | 28778394 | 29278426 | 30023241 |
| 19769962 | 21168995 | 22312723 | 22993351 | 23900441 | 25818007 | 27629453 | 28778400 | 29278451 | 30024129 |
| 19770412 | 21169802 | 22312778 | 22993374 | 23900477 | 25818660 | 27629687 | 28778675 | 29278457 | 30024767 |
| 19770433 | 21170518 | 22313402 | 22993885 | 23901330 | 25819805 | 27629849 | 28779095 | 29278460 | 30024957 |
| 19770636 | 21170652 | 22313450 | 22994011 | 23901599 | 25821375 | 27630199 | 28779516 | 29278471 | 30025027 |
| 19771003 | 21171760 | 22314190 | 22994277 | 23901845 | 25822580 | 27631525 | 28779598 | 29278477 | 30025723 |
| 19771011 | 21174449 | 22314237 | 22994372 | 23901975 | 25823157 | 27631536 | 28779735 | 29278495 | 30026968 |
| 19771394 | 21175717 | 22314449 | 22994944 | 23902276 | 25823326 | 27632367 | 28779868 | 29278502 | 30027308 |
| 19771798 | 21176055 | 22314857 | 22995316 | 23903765 | 25824192 | 27632881 | 28779922 | 29278508 | 30028192 |
| 19772735 | 21176640 | 22315188 | 22995379 | 23904077 | 25825532 | 27633261 | 28779933 | 29278509 | 30028636 |
| 19772967 | 21176981 | 22315233 | 22995712 | 23904140 | 25827125 | 27633272 | 28780155 | 29278511 | 30028945 |
| 19773005 | 21177110 | 22316587 | 22996082 | 23904420 | 25827849 | 27633462 | 28780179 | 29278513 | 30029251 |
| 19773660 | 21177458 | 22317018 | 22996242 | 23905226 | 25828628 | 27634135 | 28780402 | 29278516 | 30029252 |
| 19773705 | 21177604 | 22317567 | 22996257 | 23905281 | 25829661 | 27635935 | 28780609 | 29278517 | 30029830 |
| 19774047 | 21177805 | 22318242 | 22996302 | 23905655 | 25831971 | 27636234 | 28780925 | 29278521 | 30030275 |
| 19774057 | 21178292 | 22318324 | 22996398 | 23905790 | 25833127 | 27636781 | 28780934 | 29278529 | 30030362 |
| 19774248 | 21178303 | 22318394 | 22996467 | 23905793 | 25835193 | 27637003 | 28781615 | 29278544 | 30030943 |
| 19775278 | 21179105 | 22318456 | 22997223 | 23906008 | 25835492 | 27637158 | 28781750 | 29278569 | 30031761 |
| 19775518 | 21180261 | 22318556 | 22997327 | 23906323 | 25836750 | 27637383 | 28781759 | 29278570 | 30031890 |
| 19775626 | 21180615 | 22319071 | 22997636 | 23906326 | 25837672 | 27637741 | 28782551 | 29278578 | 30032044 |
| 19776741 | 21180616 | 22319088 | 22997753 | 23906383 | 25839061 | 27637766 | 28782868 | 29278586 | 30032639 |
| 19776900 | 21181087 | 22319167 | 22998177 | 23907211 | 25842863 | 27637921 | 28784218 | 29278591 | 30035515 |
| 19777101 | 21181212 | 22319368 | 22998325 | 23907433 | 25842877 | 27638002 | 28784273 | 29278592 | 30036085 |
| 19777374 | 21181859 | 22319776 | 22998328 | 23907531 | 25843498 | 27638444 | 28784461 | 29278598 | 30036706 |
| 19777619 | 21182393 | 22319837 | 22998456 | 23908416 | 25844297 | 27638959 | 28784583 | 29278629 | 30036792 |
| 19777865 | 21184813 | 22320106 | 22998504 | 23908613 | 25844867 | 27639132 | 28784664 | 29278648 | 30037101 |
| 19778250 | 21184950 | 22320458 | 22998938 | 23908779 | 25845409 | 27639546 | 28785137 | 29278654 | 30037128 |
| 19778595 | 21185095 | 22320966 | 22999051 | 23908863 | 25846390 | 27640324 | 28785991 | 29278660 | 30037612 |
| 19778717 | 21186218 | 22321061 | 22999655 | 23909133 | 25846852 | 27640598 | 28786223 | 29278687 | 30037652 |
| 19778870 | 21186718 | 22321136 | 22999845 | 23909258 | 25847064 | 27640987 | 28786539 | 29278690 | 30038113 |
| 19779678 | 21187087 | 22321210 | 22999852 | 23909719 | 25848668 | 27641189 | 28786974 | 29278691 | 30039149 |
| 19779689 | 21187269 | 22321211 | 23000051 | 23910385 | 25849110 | 27641241 | 28787189 | 29278702 | 30039371 |
| 19780940 | 21187646 | 22321726 | 23000088 | 23910540 | 25855263 | 27641676 | 28787353 | 29278709 | 30039404 |
| 19781043 | 21188351 | 22321751 | 23000137 | 23910602 | 25855480 | 27641951 | 28787461 | 29278747 | 30039542 |
| 19781673 | 21188478 | 22321757 | 23000325 | 23911615 | 25855951 | 27642943 | 28787963 | 29278752 | 30040063 |
| 19782071 | 21188892 | 22321884 | 23000328 | 23911775 | 25856410 | 27643129 | 28788133 | 29278758 | 30040204 |
| 19782197 | 21191008 | 22321930 | 23001175 | 23911908 | 25856543 | 27643210 | 28788675 | 29278772 | 30040584 |
| 19783239 | 21192279 | 22322394 | 23001184 | 23912174 | 25856638 | 27643823 | 28789036 | 29278773 | 30041586 |
| 19783328 | 21192674 | 22322531 | 23001400 | 23912455 | 25856663 | 27644018 | 28789337 | 29278778 | 30042027 |
| 19783851 | 21193603 | 22322596 | 23001436 | 23912584 | 25858449 | 27644380 | 28789514 | 29278781 | 30043053 |
| 19783853 | 21193886 | 22322747 | 23001449 | 23912846 | 25860438 | 27646831 | 28789979 | 29278790 | 30043130 |
| 19784458 | 21193895 | 22322930 | 23001530 | 23913797 | 25862682 | 27647461 | 28790393 | 29278794 | 30043744 |
| 19784492 | 21194666 | 22323437 | 23001743 | 23913832 | 25863486 | 27647697 | 28790931 | 29278802 | 30043764 |
| 19784513 | 21194876 | 22323918 | 23001831 | 23913922 | 25863489 | 27648863 | 28790972 | 29278805 | 30043970 |
| 19784755 | 21194934 | 22324456 | 23001937 | 23913957 | 25863980 | 27648895 | 28792069 | 29278816 | 30044773 |
| 19784931 | 21194993 | 22324614 | 23001970 | 23914275 | 25864844 | 27648976 | 28792701 | 29278820 | 30045820 |
| 19785223 | 21195662 | 22324620 | 23001979 | 23914304 | 25864865 | 27649230 | 28792777 | 29278843 | 30047367 |
| 19785741 | 21195910 | 22324685 | 23002810 | 23914672 | 25864913 | 27650073 | 28793228 | 29278846 | 30047770 |
| 19785789 | 21196261 | 22324782 | 23003052 | 23914754 | 25866044 | 27650210 | 28795684 | 29278857 | 30048103 |
| 19786087 | 21196393 | 22324856 | 23003264 | 23914781 | 25866234 | 27650220 | 28795770 | 29278858 | 30049348 |
| 19786261 | 21196465 | 22324927 | 23003461 | 23915075 | 25866361 | 27650573 | 28796448 | 29278863 | 30049488 |
| 19786586 | 21196520 | 22325022 | 23003805 | 23915220 | 25866362 | 27651074 | 28796736 | 29278864 | 30049580 |
| 19787019 | 21197373 | 22325277 | 23004234 | 23915529 | 25868407 | 27651095 | 28796970 | 29278881 | 30049773 |
| 19787228 | 21197805 | 22326021 | 23004393 | 23915564 | 25868766 | 27651167 | 28797737 | 29278885 | 30049984 |
| 19787436 | 21198909 | 22326315 | 23004459 | 23915851 | 25869375 | 27651258 | 28798977 | 29278896 | 30050560 |
| 19787550 | 21199612 | 22326349 | 23004722 | 23916219 | 25869431 | 27651313 | 28799074 | 29278899 | 30052233 |
| 19787596 | 21200178 | 22326351 | 23005079 | 23916401 | 25870278 | 27651415 | 28799295 | 29278901 | 30052651 |
| 19787950 | 21200905 | 22327059 | 23005197 | 23916611 | 25871760 | 27651486 | 28799539 | 29278904 | 30053715 |
| 19788278 | 21201375 | 22327139 | 23005286 | 23917365 | 25873059 | 27651647 | 28799613 | 29278916 | 30054129 |
| 19788312 | 21202073 | 22327537 | 23005712 | 23917616 | 25875510 | 27651756 | 28800209 | 29278918 | 30054659 |
| 19788376 | 21202423 | 22327737 | 23006645 | 23917850 | 25875838 | 27651966 | 28800287 | 29278927 | 30055103 |
| 19788637 | 21202882 | 22328099 | 23007099 | 23918237 | 25876019 | 27652078 | 28800402 | 29278945 | 30056080 |
| 19789020 | 21204357 | 22328267 | 23007376 | 23918597 | 25876242 | 27652148 | 28801038 | 29278960 | 30056564 |
| 19789084 | 21205529 | 22328302 | 23007429 | 23918632 | 25876639 | 27652150 | 28801327 | 29278965 | 30056582 |
| 19789509 | 21206236 | 22328543 | 23007701 | 23918707 | 25876820 | 27652530 | 28802538 | 29278969 | 30057612 |
| 19789941 | 21206699 | 22328613 | 23008301 | 23919022 | 25878985 | 27653358 | 28802543 | 29278970 | 30057618 |
| 19790104 | 21206811 | 22328682 | 23008381 | 23919038 | 25880961 | 27653850 | 28802618 | 29278974 | 30059466 |
| 19790560 | 21206954 | 22328869 | 23008705 | 23919209 | 25881517 | 27653933 | 28803592 | 29278987 | 30059771 |
| 19790616 | 21207477 | 22329575 | 23008752 | 23919281 | 25881748 | 27655305 | 28803614 | 29278991 | 30060050 |
| 19790921 | 21209492 | 22329707 | 23009475 | 23919788 | 25882658 | 27655421 | 28803727 | 29278992 | 30060135 |
| 19791732 | 21210199 | 22329857 | 23009694 | 23920010 | 25882666 | 27655469 | 28803929 | 29278999 | 30061344 |
| 19792367 | 21210330 | 22329940 | 23009738 | 23920437 | 25883331 | 27656700 | 28804064 | 29279010 | 30061783 |
| 19793154 | 21211644 | 22329980 | 23009754 | 23920502 | 25884806 | 27656784 | 28804455 | 29279012 | 30062356 |
| 19793767 | 21212142 | 22330399 | 23010049 | 23921010 | 25888187 | 27657302 | 28804664 | 29279014 | 30062774 |
| 19793849 | 21212346 | 22330433 | 23010280 | 23921363 | 25888623 | 27657316 | 28805325 | 29279017 | 30063861 |
| 19794167 | 21212498 | 22330909 | 23010397 | 23922544 | 25889948 | 27657789 | 28805545 | 29279022 | 30064025 |
| 19794456 | 21212794 | 22331767 | 23010536 | 23922787 | 25892311 | 27658157 | 28806753 | 29279038 | 30064620 |
| 19794572 | 21212796 | 22331813 | 23010561 | 23923252 | 25893506 | 27658344 | 28807015 | 29279039 | 30065034 |
| 19795288 | 21213163 | 22331932 | 23010694 | 23923592 | 25894048 | 27658883 | 28807412 | 29279043 | 30065340 |
| 19795642 | 21214195 | 22332652 | 23010812 | 23923733 | 25894392 | 27660044 | 28807807 | 29279044 | 30066169 |
| 19796155 | 21214367 | 22333358 | 23011104 | 23924085 | 25895124 | 27660253 | 28808062 | 29279047 | 30066678 |
| 19796404 | 21214427 | 22333512 | 23011276 | 23925259 | 25898434 | 27660664 | 28808150 | 29279050 | 30067121 |
| 19796466 | 21214495 | 22333614 | 23011519 | 23925295 | 25900220 | 27660666 | 28808273 | 29279054 | 30067249 |
| 19796628 | 21214610 | 22333626 | 23011568 | 23925506 | 25900439 | 27661152 | 28808351 | 29279058 | 30067285 |
| 19796821 | 21214637 | 22333790 | 23011618 | 23925535 | 25901960 | 27661285 | 28808938 | 29279062 | 30067347 |
| 19798913 | 21215817 | 22334020 | 23011655 | 23925818 | 25902180 | 27662147 | 28809603 | 29279067 | 30067423 |
| 19800727 | 21216488 | 22334355 | 23012241 | 23926833 | 25903721 | 27662149 | 28809626 | 29279069 | 30067818 |
| 19801606 | 21216981 | 22335746 | 23012551 | 23926929 | 25904599 | 27662375 | 28810319 | 29279086 | 30067870 |
| 19802211 | 21217229 | 22335881 | 23012599 | 23927734 | 25906863 | 27662623 | 28810674 | 29279099 | 30068012 |
| 19802299 | 21217244 | 22335948 | 23012644 | 23927888 | 25907085 | 27663307 | 28810681 | 29279106 | 30068620 |
| 19802841 | 21218174 | 22335955 | 23012883 | 23928391 | 25907542 | 27663762 | 28810829 | 29279108 | 30068729 |
| 19803213 | 21218688 | 22336084 | 23012901 | 23928447 | 25907604 | 27663881 | 28811395 | 29279113 | 30069046 |
| 19803514 | 21218900 | 22336519 | 23013094 | 23928746 | 25908303 | 27664245 | 28812776 | 29279116 | 30069690 |
| 19803717 | 21219441 | 22336575 | 23013816 | 23928954 | 25908689 | 27664738 | 28813127 | 29279129 | 30069870 |
| 19803752 | 21219496 | 22336719 | 23013893 | 23929120 | 25908893 | 27666735 | 28813303 | 29279141 | 30070852 |
| 19805112 | 21219973 | 22336912 | 23014357 | 23930582 | 25908920 | 27666738 | 28813698 | 29279164 | 30071613 |
| 19805404 | 21219979 | 22337027 | 23014407 | 23930760 | 25909098 | 27666902 | 28813847 | 29279185 | 30071674 |
| 19805919 | 21220575 | 22337039 | 23014545 | 23930814 | 25909172 | 27667128 | 28814052 | 29279196 | 30071871 |
| 19805932 | 21220860 | 22337284 | 23015133 | 23931215 | 25909591 | 27667165 | 28814346 | 29279211 | 30072252 |
| 19806618 | 21221377 | 22337537 | 23015343 | 23931393 | 25910328 | 27667443 | 28815357 | 29279213 | 30072384 |
| 19807226 | 21221752 | 22338052 | 23015388 | 23932258 | 25912678 | 27667761 | 28815457 | 29279215 | 30072906 |
| 19807276 | 21222215 | 22338063 | 23015652 | 23933638 | 25913868 | 27667829 | 28815522 | 29279219 | 30073626 |
| 19807439 | 21222391 | 22338610 | 23015947 | 23934294 | 25913964 | 27668461 | 28815593 | 29279230 | 30073692 |
| 19807977 | 21222482 | 22338842 | 23016446 | 23934500 | 25916362 | 27669105 | 28816295 | 29279231 | 30074403 |
| 19808448 | 21222492 | 22338884 | 23016801 | 23934675 | 25917657 | 27669469 | 28816921 | 29279232 | 30074608 |
| 19809545 | 21223334 | 22339104 | 23016835 | 23934919 | 25918494 | 27669867 | 28817839 | 29279234 | 30075422 |
| 19809579 | 21223499 | 22339249 | 23017583 | 23935195 | 25918674 | 27670044 | 28818829 | 29279235 | 30075735 |
| 19809581 | 21224098 | 22339346 | 23017600 | 23935316 | 25920197 | 27670310 | 28820206 | 29279236 | 30076059 |
| 19810774 | 21224524 | 22339563 | 23017678 | 23935436 | 25920223 | 27670930 | 28820582 | 29279241 | 30076090 |
| 19810780 | 21224806 | 22339797 | 23018200 | 23935514 | 25921987 | 27671867 | 28820630 | 29279245 | 30077234 |
| 19812232 | 21225113 | 22340673 | 23018429 | 23935757 | 25923781 | 27672157 | 28821361 | 29279258 | 30077666 |
| 19812410 | 21225774 | 22340932 | 23018895 | 23936773 | 25923952 | 27673715 | 28821476 | 29279274 | 30078015 |
| 19812496 | 21225844 | 22341057 | 23018913 | 23937317 | 25925699 | 27673950 | 28821656 | 29279301 | 30079337 |
| 19813102 | 21226153 | 22341363 | 23019038 | 23937511 | 25925913 | 27675255 | 28821796 | 29279306 | 30079345 |
| 19813801 | 21226632 | 22341529 | 23019172 | 23937591 | 25927171 | 27675490 | 28821854 | 29279308 | 30081281 |
| 19813902 | 21226894 | 22341618 | 23019381 | 23937700 | 25927588 | 27676653 | 28821883 | 29279312 | 30082211 |
| 19814049 | 21227164 | 22342024 | 23019603 | 23937952 | 25927983 | 27676851 | 28822123 | 29279314 | 30082486 |
| 19814472 | 21227216 | 22342252 | 23019947 | 23938625 | 25928156 | 27677382 | 28822515 | 29279320 | 30083007 |
| 19815451 | 21227772 | 22342261 | 23020297 | 23939880 | 25928219 | 27677605 | 28823415 | 29279323 | 30083538 |
| 19815952 | 21227799 | 22343006 | 23020360 | 23939895 | 25928987 | 27677815 | 28823907 | 29279331 | 30083554 |
| 19816422 | 21227937 | 22343486 | 23020639 | 23940603 | 25930198 | 27678763 | 28824048 | 29279350 | 30083571 |
| 19816682 | 21229263 | 22343973 | 23021179 | 23941443 | 25930774 | 27678821 | 28824277 | 29279360 | 30083587 |
| 19816927 | 21229992 | 22344533 | 23021273 | 23941612 | 25930919 | 27678932 | 28824956 | 29279363 | 30083590 |
| 19816988 | 21230088 | 22344627 | 23021627 | 23941764 | 25932169 | 27679080 | 28825041 | 29279370 | 30084868 |
| 19817331 | 21230987 | 22345174 | 23022554 | 23942167 | 25932252 | 27679341 | 28825511 | 29279378 | 30085662 |
| 19817483 | 21231925 | 22345251 | 23022653 | 23942686 | 25933300 | 27679355 | 28825658 | 29279387 | 30087120 |
| 19817740 | 21232152 | 22345457 | 23022678 | 23942776 | 25933712 | 27679799 | 28826050 | 29279393 | 30087151 |
| 19818171 | 21232435 | 22345888 | 23022697 | 23943047 | 25934053 | 27679822 | 28827111 | 29279394 | 30088176 |
| 19818812 | 21233348 | 22345917 | 23022739 | 23943083 | 25934445 | 27680280 | 28827310 | 29279397 | 30088993 |
| 19819547 | 21234274 | 22346258 | 23022942 | 23944270 | 25934869 | 27680436 | 28827536 | 29279401 | 30090443 |
| 19819565 | 21234328 | 22346929 | 23023137 | 23944737 | 25936169 | 27682044 | 28828256 | 29279410 | 30090520 |
| 19819865 | 21234588 | 22346943 | 23023329 | 23944747 | 25938513 | 27684184 | 28828418 | 29279417 | 30091582 |
| 19821163 | 21236289 | 22347467 | 23024314 | 23944770 | 25938705 | 27684323 | 28828420 | 29279424 | 30092155 |
| 19821945 | 21236728 | 22347623 | 23024354 | 23945570 | 25939201 | 27684371 | 28828612 | 29279444 | 30092672 |
| 19822048 | 21236943 | 22347631 | 23024398 | 23945744 | 25939782 | 27684433 | 28828769 | 29279466 | 30093897 |
| 19822298 | 21236991 | 22347742 | 23025255 | 23945935 | 25940433 | 27684820 | 28828838 | 29279468 | 30094160 |
| 19823624 | 21237102 | 22348346 | 23026581 | 23946280 | 25941017 | 27685533 | 28829109 | 29279485 | 30094198 |
| 19823941 | 21237625 | 22348886 | 23026968 | 23946354 | 25941265 | 27685564 | 28829142 | 29279497 | 30094526 |
| 19824257 | 21238772 | 22349816 | 23027090 | 23946696 | 25941906 | 27685910 | 28829208 | 29279505 | 30095069 |
| 19824333 | 21238815 | 22349965 | 23027684 | 23947214 | 25942102 | 27686012 | 28829415 | 29279519 | 30096466 |
| 19824433 | 21238967 | 22349973 | 23028570 | 23947643 | 25943914 | 27686256 | 28829474 | 29279548 | 30096851 |
| 19824444 | 21239052 | 22350122 | 23028663 | 23948049 | 25944265 | 27686746 | 28829514 | 29279549 | 30096974 |
| 19825716 | 21239204 | 22350178 | 23028773 | 23948056 | 25945564 | 27687219 | 28829519 | 29279550 | 30096987 |
| 19825753 | 21239450 | 22350397 | 23028802 | 23948361 | 25946061 | 27687423 | 28829566 | 29279554 | 30097152 |
| 19826136 | 21239587 | 22350552 | 23028956 | 23948595 | 25946803 | 27687514 | 28830029 | 29279567 | 30098065 |
| 19826235 | 21239682 | 22350694 | 23029175 | 23948861 | 25946952 | 27689202 | 28830455 | 29279568 | 30098549 |
| 19827408 | 21239773 | 22350981 | 23029287 | 23948917 | 25947128 | 27689206 | 28830855 | 29279569 | 30098806 |
| 19827417 | 21240305 | 22351158 | 23029709 | 23949333 | 25947226 | 27689626 | 28832468 | 29279574 | 30098934 |
| 19827795 | 21241972 | 22351586 | 23029830 | 23949473 | 25947268 | 27689735 | 28832891 | 29279580 | 30100491 |
| 19827980 | 21242119 | 22351652 | 23029884 | 23950219 | 25947360 | 27692329 | 28832969 | 29279581 | 30101407 |
| 19830405 | 21242297 | 22351681 | 23030014 | 23950618 | 25948384 | 27692458 | 28833007 | 29279592 | 30101413 |
| 19830456 | 21243311 | 22351731 | 23030141 | 23950944 | 25951944 | 27693299 | 28833159 | 29279599 | 30101452 |
| 19830567 | 21243778 | 22352000 | 23030260 | 23951272 | 25952148 | 27693468 | 28833693 | 29279604 | 30101722 |
| 19831363 | 21243784 | 22352047 | 23030465 | 23951593 | 25952368 | 27693770 | 28833778 | 29279607 | 30101729 |
| 19831428 | 21244230 | 22352073 | 23030674 | 23951815 | 25952469 | 27694547 | 28833883 | 29279626 | 30102349 |
| 19831499 | 21244354 | 22352185 | 23030688 | 23952022 | 25952936 | 27695229 | 28834162 | 29279632 | 30102589 |
| 19831691 | 21244625 | 22352863 | 23030693 | 23952679 | 25953228 | 27695230 | 28834235 | 29279634 | 30102715 |
| 19831693 | 21245490 | 22353037 | 23030705 | 23952892 | 25953625 | 27695542 | 28834332 | 29279641 | 30103159 |
| 19831728 | 21245855 | 22353577 | 23030752 | 23952979 | 25954463 | 27695900 | 28834969 | 29279642 | 30104019 |
| 19831734 | 21246126 | 22353950 | 23030760 | 23953400 | 25954673 | 27696546 | 28835289 | 29279644 | 30104097 |
| 19832055 | 21246127 | 22353980 | 23031007 | 23953552 | 25955951 | 27696933 | 28835841 | 29279661 | 30104287 |
| 19833599 | 21246801 | 22353991 | 23031116 | 23953808 | 25956803 | 27696971 | 28835878 | 29279677 | 30104572 |
| 19833966 | 21247025 | 22354118 | 23031181 | 23954572 | 25956953 | 27697539 | 28835906 | 29279682 | 30105172 |
| 19834293 | 21247372 | 22354136 | 23031316 | 23954588 | 25957716 | 27698231 | 28835960 | 29279685 | 30105662 |
| 19834473 | 21249035 | 22354217 | 23031348 | 23955736 | 25958838 | 27698608 | 28836458 | 29279686 | 30106007 |
| 19834876 | 21249289 | 22354443 | 23031366 | 23955851 | 25959350 | 27699012 | 28836863 | 29279695 | 30106042 |
| 19834967 | 21249363 | 22354449 | 23031682 | 23956246 | 25961240 | 27699106 | 28837044 | 29279700 | 30107739 |
| 19835132 | 21249781 | 22354838 | 23031782 | 23956353 | 25963269 | 27699432 | 28837532 | 29279707 | 30107995 |
| 19835338 | 21251551 | 22355071 | 23032017 | 23957109 | 25963519 | 27700130 | 28837664 | 29279715 | 30108065 |
| 19835510 | 21251571 | 22355194 | 23032116 | 23957314 | 25963926 | 27700191 | 28837823 | 29279717 | 30108067 |
| 19835859 | 21251894 | 22355322 | 23032271 | 23957508 | 25964207 | 27701051 | 28837851 | 29279720 | 30108436 |
| 19836457 | 21252115 | 22355443 | 23032296 | 23957742 | 25965093 | 27701162 | 28837875 | 29279722 | 30108929 |
| 19836969 | 21253164 | 22355452 | 23032778 | 23958247 | 25967254 | 27701740 | 28838178 | 29279726 | 30109406 |
| 19837779 | 21253696 | 22355585 | 23032801 | 23959296 | 25968303 | 27701944 | 28839027 | 29279744 | 30109484 |
| 19838404 | 21254748 | 22357134 | 23032821 | 23959790 | 25968330 | 27702439 | 28839104 | 29279746 | 30109536 |
| 19839074 | 21254971 | 22357362 | 23032881 | 23959853 | 25968470 | 27702859 | 28839241 | 29279747 | 30109892 |
| 19839222 | 21255354 | 22357819 | 23033200 | 23960041 | 25968518 | 27702995 | 28839276 | 29279759 | 30110295 |
| 19839497 | 21255641 | 22357944 | 23033227 | 23960280 | 25968705 | 27703981 | 28839286 | 29279761 | 30110401 |
| 19839728 | 21256097 | 22358037 | 23033310 | 23960294 | 25969039 | 27704555 | 28839328 | 29279766 | 30111367 |
| 19839775 | 21256298 | 22358047 | 23033420 | 23961297 | 25970578 | 27704571 | 28839641 | 29279774 | 30111766 |
| 19839791 | 21256322 | 22358389 | 23033422 | 23961365 | 25970851 | 27704628 | 28839747 | 29279778 | 30111918 |
| 19840891 | 21257146 | 22358417 | 23033558 | 23962401 | 25971068 | 27705878 | 28840108 | 29279779 | 30112008 |
| 19841175 | 21257286 | 22358576 | 23033580 | 23962503 | 25971363 | 27706042 | 28840349 | 29279809 | 30112131 |
| 19841711 | 21258061 | 22359207 | 23033787 | 23962886 | 25971415 | 27706404 | 28840833 | 29279812 | 30113375 |
| 19842204 | 21258207 | 22359380 | 23033935 | 23963202 | 25971563 | 27706677 | 28841066 | 29279825 | 30113898 |
| 19842587 | 21258431 | 22359504 | 23034046 | 23963374 | 25972748 | 27706819 | 28841326 | 29279827 | 30114339 |
| 19842689 | 21258546 | 22359837 | 23034118 | 23963734 | 25972768 | 27707469 | 28841703 | 29279837 | 30115023 |
| 19843060 | 21258591 | 22360086 | 23034948 | 23963782 | 25973223 | 27707525 | 28841797 | 29279855 | 30116235 |
| 19843419 | 21258602 | 22360408 | 23035082 | 23963797 | 25974973 | 27707828 | 28841836 | 29279856 | 30116350 |
| 19843997 | 21259046 | 22360534 | 23035244 | 23964093 | 25975175 | 27708726 | 28842146 | 29279877 | 30116447 |
| 19844102 | 21260623 | 22360893 | 23035489 | 23964110 | 25975940 | 27708960 | 28842253 | 29279882 | 30116632 |
| 19844189 | 21261449 | 22361490 | 23035573 | 23964139 | 25976179 | 27709139 | 28842543 | 29279893 | 30116777 |
| 19844450 | 21261517 | 22361508 | 23035705 | 23964530 | 25976657 | 27710103 | 28842743 | 29279903 | 30116969 |
| 19844520 | 21261760 | 22361693 | 23036343 | 23965038 | 25978211 | 27710460 | 28842800 | 29279905 | 30117260 |
| 19844595 | 21261818 | 22361714 | 23036640 | 23965170 | 25978817 | 27710577 | 28843049 | 29279915 | 30117701 |
| 19844923 | 21262033 | 22361794 | 23037253 | 23965187 | 25978830 | 27711686 | 28843198 | 29279929 | 30118243 |
| 19844998 | 21262312 | 22361993 | 23037469 | 23965188 | 25978896 | 27711715 | 28843250 | 29279931 | 30118319 |
| 19845131 | 21263308 | 22362105 | 23037902 | 23965640 | 25979882 | 27712273 | 28843683 | 29279955 | 30118780 |
| 19845423 | 21263987 | 22362139 | 23038162 | 23966391 | 25982605 | 27712290 | 28843777 | 29279958 | 30118917 |
| 19845516 | 21264015 | 22362569 | 23038424 | 23967617 | 25983417 | 27712392 | 28844078 | 29279965 | 30119199 |
| 19845800 | 21264416 | 22362614 | 23038530 | 23968009 | 25984451 | 27712462 | 28844214 | 29279982 | 30119388 |
| 19846198 | 21264628 | 22362640 | 23038610 | 23969111 | 25987998 | 27712543 | 28845003 | 29279995 | 30119611 |
| 19846764 | 21264908 | 22362727 | 23038643 | 23969377 | 25989252 | 27712663 | 28845497 | 29279996 | 30119745 |
| 19846883 | 21265275 | 22363491 | 23038810 | 23969388 | 25989608 | 27713681 | 28845867 | 29280003 | 30119970 |
| 19847478 | 21265509 | 22363746 | 23038946 | 23969409 | 25992768 | 27713777 | 28845872 | 29280026 | 30120475 |
| 19847501 | 21265788 | 22363919 | 23039088 | 23969531 | 25993274 | 27714177 | 28846029 | 29280027 | 30121900 |
| 19847751 | 21266622 | 22364010 | 23039125 | 23969540 | 25993480 | 27714537 | 28846798 | 29280031 | 30123341 |
| 19847998 | 21267055 | 22364551 | 23039710 | 23969603 | 25993499 | 27714763 | 28846963 | 29280035 | 30124082 |
| 19849672 | 21267088 | 22364709 | 23039750 | 23969686 | 25993572 | 27715046 | 28847467 | 29280037 | 30124259 |
| 19851008 | 21267447 | 22364813 | 23039859 | 23969777 | 25993816 | 27716254 | 28847526 | 29280046 | 30125122 |
| 19851141 | 21268640 | 22365202 | 23039927 | 23969877 | 25994823 | 27716298 | 28847535 | 29280060 | 30125716 |
| 19851840 | 21270320 | 22365691 | 23039977 | 23970273 | 25996175 | 27716338 | 28847648 | 29280066 | 30126276 |
| 19852433 | 21270607 | 22365712 | 23040383 | 23970336 | 25996334 | 27716584 | 28848961 | 29280071 | 30126617 |
| 19852475 | 21270917 | 22365976 | 23040845 | 23970384 | 25996575 | 27717074 | 28850610 | 29280083 | 30127030 |
| 19852549 | 21271917 | 22366115 | 23041082 | 23970714 | 25998311 | 27717134 | 28852062 | 29280091 | 30127045 |
| 19852716 | 21272738 | 22366232 | 23041115 | 23970769 | 25999428 | 27717391 | 28852075 | 29280095 | 30127630 |
| 19852777 | 21272948 | 22366244 | 23041202 | 23971450 | 25999649 | 27717508 | 28852162 | 29280102 | 30127685 |
| 19852957 | 21273304 | 22366335 | 23041440 | 23971528 | 26001064 | 27717766 | 28852515 | 29280103 | 30128292 |
| 19853085 | 21273343 | 22366621 | 23041553 | 23972007 | 26001065 | 27718932 | 28853576 | 29280106 | 30128738 |
| 19853124 | 21273647 | 22366642 | 23041577 | 23972483 | 26001603 | 27719730 | 28853762 | 29280113 | 30129696 |
| 19854242 | 21274132 | 22366904 | 23042607 | 23972580 | 26003403 | 27719819 | 28855363 | 29280116 | 30130216 |
| 19854662 | 21274806 | 22367542 | 23042684 | 23973020 | 26006103 | 27719956 | 28856186 | 29280120 | 30130396 |
| 19854676 | 21275897 | 22367705 | 23042781 | 23973048 | 26008293 | 27720390 | 28856418 | 29280126 | 30131499 |
| 19854706 | 21275939 | 22367845 | 23043136 | 23973239 | 26008953 | 27721360 | 28856535 | 29280133 | 30131683 |
| 19854942 | 21278277 | 22367887 | 23043535 | 23973431 | 26009014 | 27721609 | 28856649 | 29280147 | 30132627 |
| 19855616 | 21278346 | 22368029 | 23043593 | 23973464 | 26009659 | 27721916 | 28856781 | 29280150 | 30132945 |
| 19856070 | 21278541 | 22368205 | 23044095 | 23974251 | 26011515 | 27722181 | 28857747 | 29280152 | 30133095 |
| 19856191 | 21280576 | 22368599 | 23044370 | 23974683 | 26014960 | 27722315 | 28857940 | 29280161 | 30134183 |
| 19856421 | 21281098 | 22368633 | 23044532 | 23974962 | 26015286 | 27722385 | 28857967 | 29280166 | 30134547 |
| 19856482 | 21281346 | 22368711 | 23044777 | 23975067 | 26015491 | 27722681 | 28857989 | 29280169 | 30134932 |
| 19856869 | 21281454 | 22368836 | 23044860 | 23975381 | 26015768 | 27722763 | 28858002 | 29280171 | 30136413 |
| 19856954 | 21281504 | 22369006 | 23045029 | 23975902 | 26015842 | 27723824 | 28858374 | 29280178 | 30136707 |
| 19857097 | 21284447 | 22369012 | 23045299 | 23976735 | 26015970 | 27723829 | 28858506 | 29280188 | 30136792 |
| 19857529 | 21284696 | 22369165 | 23045342 | 23977189 | 26017088 | 27724169 | 28859015 | 29280195 | 30137123 |
| 19857563 | 21284812 | 22369277 | 23045563 | 23977226 | 26017275 | 27724536 | 28859554 | 29280197 | 30137821 |
| 19857700 | 21285085 | 22369574 | 23045591 | 23977490 | 26017332 | 27724682 | 28859991 | 29280222 | 30137965 |
| 19858269 | 21287779 | 22369694 | 23045846 | 23977634 | 26018010 | 27724718 | 28860291 | 29280224 | 30138262 |
| 19858313 | 21287906 | 22370039 | 23046083 | 23977907 | 26018326 | 27724851 | 28860729 | 29280230 | 30138309 |
| 19859025 | 21288014 | 22370501 | 23046172 | 23978798 | 26018819 | 27724914 | 28861029 | 29280232 | 30138731 |
| 19859535 | 21288021 | 22370512 | 23046368 | 23979491 | 26020711 | 27725164 | 28861138 | 29280245 | 30138987 |
| 19859839 | 21289566 | 22370721 | 23046475 | 23979991 | 26021351 | 27725671 | 28861635 | 29280249 | 30139257 |
| 19859935 | 21289576 | 22370780 | 23046504 | 23980016 | 26021899 | 27725693 | 28861695 | 29280272 | 30139384 |
| 19860200 | 21290514 | 22370914 | 23046633 | 23980027 | 26022094 | 27726104 | 28861827 | 29280282 | 30140128 |
| 19860360 | 21290976 | 22371019 | 23046664 | 23980288 | 26023339 | 27726800 | 28861974 | 29280292 | 30140435 |
| 19860906 | 21291634 | 22371206 | 23047371 | 23980295 | 26023577 | 27727724 | 28862024 | 29280297 | 30141152 |
| 19861016 | 21294050 | 22371364 | 23047421 | 23980599 | 26023988 | 27728391 | 28863343 | 29280298 | 30141326 |
| 19861090 | 21294069 | 22371430 | 23047423 | 23981035 | 26024244 | 27728498 | 28864312 | 29280309 | 30141730 |
| 19861408 | 21294541 | 22371525 | 23047666 | 23981074 | 26024546 | 27728799 | 28864466 | 29280318 | 30141832 |
| 19861459 | 21294677 | 22371701 | 23047680 | 23981259 | 26025557 | 27729106 | 28864888 | 29280324 | 30142715 |
| 19861857 | 21295200 | 22371764 | 23047862 | 23981359 | 26026220 | 27729126 | 28865157 | 29280331 | 30143496 |
| 19862159 | 21295411 | 22371927 | 23048245 | 23981492 | 26026549 | 27729655 | 28865205 | 29280335 | 30143546 |
| 19862165 | 21295565 | 22371994 | 23048561 | 23981861 | 26026708 | 27730098 | 28865674 | 29280343 | 30144945 |
| 19862354 | 21295781 | 22372589 | 23048610 | 23982006 | 26027570 | 27730661 | 28865988 | 29280344 | 30145707 |
| 19863205 | 21297048 | 22372638 | 23048612 | 23982922 | 26027697 | 27730678 | 28866426 | 29280384 | 30145984 |
| 19863864 | 21299972 | 22373261 | 23048707 | 23983872 | 26027905 | 27731423 | 28866942 | 29280387 | 30146835 |
| 19863932 | 21300508 | 22373327 | 23048904 | 23983945 | 26031090 | 27732185 | 28867258 | 29280400 | 30147325 |
| 19864209 | 21301328 | 22373379 | 23048909 | 23984049 | 26031238 | 27732462 | 28867332 | 29280404 | 30147450 |
| 19864218 | 21301551 | 22373647 | 23049435 | 23984548 | 26031889 | 27733260 | 28867530 | 29280413 | 30147992 |
| 19864873 | 21302001 | 22373655 | 23050015 | 23984619 | 26033276 | 27733302 | 28867621 | 29280426 | 30148729 |
| 19864913 | 21302406 | 22373667 | 23050285 | 23985676 | 26033396 | 27733626 | 28868120 | 29280432 | 30151139 |
| 19865351 | 21302579 | 22373984 | 23050336 | 23986138 | 26034171 | 27734292 | 28868161 | 29280435 | 30151633 |
| 19865423 | 21303343 | 22374292 | 23050417 | 23986547 | 26034522 | 27734607 | 28868230 | 29280438 | 30151639 |
| 19865724 | 21303685 | 22374301 | 23050451 | 23986856 | 26035649 | 27734940 | 28868481 | 29280442 | 30152429 |
| 19866201 | 21304230 | 22374838 | 23050807 | 23986884 | 26037383 | 27735548 | 28869486 | 29280445 | 30152644 |
| 19866592 | 21304519 | 22375050 | 23051443 | 23986886 | 26037746 | 27735599 | 28869557 | 29280450 | 30153133 |
| 19866828 | 21305717 | 22375132 | 23051562 | 23986958 | 26038908 | 27735723 | 28869716 | 29280474 | 30153655 |
| 19867360 | 21306405 | 22375497 | 23051771 | 23986969 | 26039431 | 27735866 | 28869863 | 29280522 | 30153656 |
| 19867619 | 21306874 | 22375615 | 23051775 | 23987117 | 26039483 | 27735987 | 28869993 | 29280530 | 30154449 |
| 19867900 | 21306962 | 22376516 | 23051815 | 23987754 | 26039699 | 27736336 | 28871071 | 29280549 | 30155280 |
| 19869519 | 21307262 | 22376552 | 23051995 | 23988667 | 26040682 | 27737703 | 28872545 | 29280559 | 30155721 |
| 19869979 | 21307682 | 22376676 | 23052228 | 23989021 | 26041256 | 27738860 | 28873869 | 29280574 | 30156196 |
| 19870308 | 21307915 | 22377138 | 23052415 | 23989114 | 26041452 | 27739254 | 28873940 | 29280577 | 30157111 |
| 19870662 | 21309141 | 22377299 | 23052423 | 23990133 | 26042136 | 27739309 | 28875326 | 29280587 | 30157700 |
| 19871031 | 21309287 | 22377713 | 23052551 | 23990220 | 26042701 | 27739362 | 28875507 | 29280588 | 30158178 |
| 19871142 | 21311531 | 22377730 | 23052965 | 23990275 | 26042805 | 27739407 | 28876966 | 29280590 | 30158882 |
| 19871963 | 21312689 | 22377953 | 23053001 | 23990404 | 26044764 | 27740263 | 28877530 | 29280594 | 30159126 |
| 19872469 | 21313617 | 22377969 | 23053725 | 23990642 | 26047497 | 27740502 | 28877643 | 29280612 | 30159203 |
| 19873075 | 21313691 | 22377974 | 23053809 | 23990673 | 26048794 | 27740606 | 28877980 | 29280613 | 30159881 |
| 19873538 | 21314457 | 22378482 | 23054012 | 23990677 | 26049293 | 27741658 | 28878361 | 29280615 | 30159955 |
| 19873598 | 21314651 | 22378831 | 23054269 | 23991924 | 26049625 | 27742405 | 28878672 | 29280619 | 30159991 |
| 19874013 | 21314746 | 22378844 | 23054396 | 23992536 | 26049921 | 27742633 | 28878962 | 29280621 | 30160017 |
| 19874456 | 21314763 | 22379430 | 23054615 | 23993362 | 26051504 | 27743325 | 28879483 | 29280629 | 30160041 |
| 19874738 | 21315653 | 22379734 | 23055046 | 23993674 | 26051619 | 27743466 | 28880016 | 29280646 | 30160148 |
| 19874855 | 21315698 | 22380088 | 23055738 | 23996950 | 26052354 | 27743508 | 28880713 | 29280647 | 30160204 |
| 19875249 | 21317907 | 22380127 | 23055972 | 23997595 | 26052543 | 27744408 | 28880744 | 29280653 | 30160287 |
| 19875449 | 21318220 | 22380396 | 23056200 | 23997967 | 26052557 | 27744605 | 28881083 | 29280661 | 30160470 |
| 19875455 | 21318579 | 22380561 | 23056378 | 23998441 | 26055237 | 27745403 | 28881896 | 29280662 | 30160551 |
| 19875927 | 21319459 | 22380657 | 23056704 | 23998534 | 26055594 | 27745426 | 28882073 | 29280667 | 30160856 |
| 19877415 | 21321240 | 22380674 | 23057305 | 23999551 | 26056492 | 27745554 | 28882527 | 29280668 | 30161951 |
| 19877547 | 21321417 | 22380704 | 23057313 | 24000030 | 26057690 | 27746355 | 28883338 | 29280669 | 30161990 |
| 19878003 | 21321836 | 22380734 | 23057365 | 24000609 | 26057747 | 27746745 | 28883506 | 29280670 | 30162075 |
| 19878335 | 21322294 | 22380970 | 23057578 | 24001298 | 26058008 | 27746887 | 28883768 | 29280684 | 30162080 |
| 19878751 | 21322705 | 22381234 | 23058173 | 24001764 | 26058715 | 27746957 | 28884309 | 29280691 | 30162191 |
| 19879364 | 21324344 | 22381308 | 23058437 | 24001876 | 26060946 | 27747402 | 28884953 | 29280704 | 30163568 |
| 19879439 | 21325514 | 22381372 | 23058519 | 24002321 | 26061347 | 27747690 | 28885171 | 29280708 | 30163773 |
| 19879691 | 21325724 | 22381548 | 23058779 | 24002520 | 26061504 | 27748355 | 28885349 | 29280709 | 30164007 |
| 19879822 | 21326590 | 22381997 | 23059068 | 24002632 | 26062030 | 27748369 | 28885686 | 29280716 | 30164815 |
| 19880256 | 21327003 | 22382116 | 23059289 | 24002947 | 26062595 | 27748670 | 28886354 | 29280717 | 30164977 |
| 19880567 | 21327138 | 22382380 | 23059825 | 24003031 | 26063242 | 27749273 | 28886416 | 29280719 | 30165162 |
| 19880648 | 21328203 | 22382989 | 23060006 | 24003153 | 26063726 | 27749792 | 28886775 | 29280723 | 30166803 |
| 19880885 | 21329273 | 22382996 | 23060046 | 24003216 | 26064229 | 27750566 | 28886776 | 29280734 | 30166859 |
| 19881421 | 21329612 | 22383095 | 23060524 | 24003298 | 26065005 | 27750769 | 28887052 | 29280735 | 30167289 |
| 19881453 | 21329807 | 22383299 | 23060546 | 24003360 | 26066376 | 27751073 | 28887724 | 29280736 | 30167325 |
| 19881471 | 21330877 | 22383319 | 23061150 | 24003522 | 26066541 | 27751277 | 28888012 | 29280737 | 30167510 |
| 19881495 | 21331025 | 22383322 | 23061256 | 24003639 | 26071009 | 27751388 | 28888320 | 29280758 | 30167925 |
| 19881637 | 21331358 | 22383579 | 23061283 | 24003655 | 26071738 | 27751957 | 28889886 | 29280762 | 30168394 |
| 19882581 | 21332154 | 22383770 | 23061520 | 24003818 | 26072534 | 27752323 | 28889903 | 29280766 | 30168740 |
| 19882741 | 21333824 | 22384159 | 23061612 | 24004088 | 26073591 | 27752796 | 28890509 | 29280776 | 30168846 |
| 19883028 | 21335992 | 22384201 | 23061722 | 24005036 | 26073652 | 27752824 | 28890537 | 29280780 | 30168906 |
| 19883307 | 21336614 | 22384531 | 23061911 | 24005881 | 26073950 | 27753223 | 28890821 | 29280782 | 30169335 |
| 19884024 | 21337612 | 22384566 | 23062271 | 24006418 | 26074243 | 27753580 | 28891266 | 29280798 | 30169385 |
| 19884080 | 21338803 | 22384854 | 23062294 | 24006631 | 26074355 | 27754033 | 28891387 | 29280802 | 30169485 |
| 19884459 | 21338883 | 22384950 | 23062453 | 24007057 | 26074452 | 27754424 | 28892021 | 29280811 | 30169679 |
| 19884469 | 21339268 | 22385110 | 23062581 | 24007401 | 26075657 | 27754617 | 28892311 | 29280822 | 30169703 |
| 19884494 | 21339612 | 22385153 | 23062617 | 24007420 | 26077824 | 27754662 | 28892395 | 29280825 | 30169715 |
| 19884995 | 21341380 | 22385570 | 23062751 | 24007894 | 26078076 | 27754803 | 28893624 | 29280834 | 30169797 |
| 19885629 | 21341546 | 22385753 | 23062860 | 24008132 | 26078223 | 27754869 | 28893858 | 29280836 | 30170840 |
| 19886288 | 21341648 | 22386345 | 23063189 | 24008193 | 26079988 | 27755360 | 28894599 | 29280843 | 30172654 |
| 19887022 | 21342498 | 22387033 | 23063477 | 24008286 | 26080049 | 27755397 | 28894693 | 29280852 | 30172878 |
| 19887078 | 21343355 | 22387211 | 23063603 | 24008755 | 26081434 | 27755797 | 28895077 | 29280856 | 30172976 |
| 19887128 | 21344150 | 22387429 | 23063645 | 24009681 | 26081763 | 27756407 | 28895339 | 29280863 | 30173051 |
| 19887467 | 21344235 | 22387476 | 23063821 | 24009744 | 26085619 | 27756657 | 28895341 | 29280866 | 30173071 |
| 19887888 | 21344256 | 22387490 | 23063825 | 24009900 | 26086814 | 27756693 | 28895599 | 29280874 | 30174207 |
| 19888146 | 21344428 | 22387916 | 23063977 | 24010239 | 26087290 | 27756779 | 28895652 | 29280899 | 30175746 |
| 19888984 | 21344922 | 22387974 | 23064043 | 24011124 | 26087409 | 27757112 | 28895993 | 29280900 | 30175791 |
| 19889567 | 21345391 | 22388177 | 23064201 | 24012527 | 26087708 | 27757293 | 28896332 | 29280904 | 30175953 |
| 19889635 | 21345815 | 22388234 | 23064330 | 24012899 | 26088813 | 27757573 | 28896494 | 29280919 | 30175992 |
| 19890764 | 21346064 | 22388762 | 23064386 | 24013038 | 26089003 | 27757580 | 28896997 | 29280920 | 30176399 |
| 19891171 | 21346153 | 22388999 | 23064867 | 24013252 | 26089373 | 27757815 | 28898052 | 29280929 | 30178372 |
| 19892101 | 21346354 | 22389337 | 23064949 | 24013384 | 26089882 | 27757844 | 28899272 | 29280950 | 30178453 |
| 19892231 | 21346632 | 22389686 | 23065113 | 24013473 | 26090130 | 27758355 | 28899991 | 29280959 | 30178481 |
| 19892418 | 21347383 | 22389899 | 23065396 | 24013693 | 26090133 | 27758864 | 28900196 | 29280974 | 30178985 |
| 19892700 | 21348523 | 22390004 | 23065613 | 24013876 | 26090910 | 27759521 | 28900461 | 29280990 | 30179352 |
| 19893320 | 21348543 | 22390057 | 23065742 | 24014170 | 26091895 | 27760382 | 28900603 | 29280995 | 30179496 |
| 19893693 | 21348853 | 22390291 | 23066726 | 24014404 | 26092500 | 27760784 | 28901134 | 29281021 | 30179550 |
| 19894659 | 21349195 | 22390335 | 23066759 | 24015072 | 26092526 | 27760826 | 28901345 | 29281027 | 30179769 |
| 19895059 | 21349560 | 22390367 | 23066938 | 24015124 | 26094464 | 27761346 | 28901352 | 29281037 | 30179924 |
| 19895531 | 21350065 | 22390404 | 23066985 | 24015177 | 26096534 | 27761631 | 28901413 | 29281040 | 30180395 |
| 19895858 | 21350082 | 22390851 | 23067250 | 24015677 | 26097209 | 27761960 | 28902258 | 29281052 | 30180576 |
| 19895895 | 21351939 | 22390867 | 23067363 | 24015801 | 26097347 | 27762755 | 28902545 | 29281057 | 30180675 |
| 19896007 | 21353125 | 22390922 | 23067500 | 24016289 | 26099052 | 27762766 | 28902558 | 29281070 | 30181665 |
| 19896322 | 21353293 | 22391053 | 23067608 | 24016432 | 26099737 | 27763572 | 28902909 | 29281092 | 30181725 |
| 19896330 | 21354550 | 22391576 | 23067687 | 24016641 | 26100347 | 27763842 | 28903053 | 29281119 | 30182335 |
| 19897562 | 21355761 | 22391765 | 23068158 | 24017038 | 26100781 | 27763853 | 28903368 | 29281120 | 30182511 |
| 19898088 | 21356069 | 22392290 | 23068409 | 24017189 | 26102159 | 27764522 | 28903567 | 29281126 | 30182981 |
| 19898499 | 21356278 | 22392628 | 23068938 | 24017520 | 26104147 | 27764637 | 28903900 | 29281128 | 30183236 |
| 19898871 | 21357066 | 22392831 | 23068994 | 24017752 | 26105089 | 27764821 | 28904513 | 29281134 | 30183307 |
| 19899173 | 21357829 | 22392890 | 23069000 | 24018290 | 26105333 | 27765970 | 28904706 | 29281138 | 30183586 |
| 19899227 | 21359264 | 22392924 | 23069464 | 24019301 | 26107662 | 27766395 | 28905007 | 29281150 | 30183935 |
| 19899415 | 21359585 | 22393452 | 23069945 | 24019841 | 26108774 | 27766775 | 28905241 | 29281153 | 30184235 |
| 19899889 | 21361058 | 22393582 | 23070367 | 24020287 | 26109319 | 27766837 | 28905564 | 29281159 | 30184626 |
| 19900100 | 21361363 | 22393597 | 23070380 | 24020293 | 26109949 | 27767053 | 28905768 | 29281165 | 30186183 |
| 19900234 | 21361365 | 22393631 | 23070388 | 24020688 | 26110582 | 27767273 | 28906395 | 29281182 | 30186711 |
| 19900466 | 21361573 | 22393820 | 23070558 | 24020723 | 26111458 | 27767779 | 28906986 | 29281187 | 30186944 |
| 19900599 | 21361646 | 22394667 | 23070559 | 24020875 | 26111742 | 27767801 | 28907153 | 29281198 | 30187622 |
| 19900904 | 21362392 | 22394706 | 23070642 | 24021231 | 26111859 | 27768379 | 28907500 | 29281200 | 30188829 |
| 19901040 | 21363206 | 22394850 | 23071069 | 24021490 | 26112360 | 27768954 | 28907567 | 29281209 | 30188954 |
| 19901380 | 21363235 | 22395075 | 23071162 | 24021556 | 26112762 | 27770347 | 28907680 | 29281210 | 30189001 |
| 19901595 | 21363331 | 22395218 | 23071261 | 24021964 | 26116386 | 27770595 | 28908209 | 29281221 | 30189390 |
| 19901629 | 21363908 | 22395767 | 23071660 | 24022122 | 26117140 | 27770750 | 28908481 | 29281229 | 30189724 |
| 19901716 | 21363925 | 22396337 | 23071751 | 24022160 | 26117734 | 27771045 | 28909776 | 29281231 | 30190030 |
| 19903032 | 21364129 | 22396376 | 23071800 | 24022446 | 26117944 | 27771187 | 28909967 | 29281238 | 30190149 |
| 19903106 | 21365572 | 22396569 | 23071924 | 24022463 | 26118198 | 27771968 | 28910352 | 29281242 | 30190250 |
| 19903360 | 21365613 | 22396850 | 23072329 | 24022850 | 26118444 | 27772066 | 28911869 | 29281262 | 30190296 |
| 19903555 | 21365900 | 22396963 | 23072365 | 24023173 | 26119137 | 27772357 | 28912266 | 29281265 | 30190839 |
| 19903608 | 21366061 | 22397247 | 23072645 | 24023504 | 26122276 | 27772677 | 28912856 | 29281278 | 30190866 |
| 19904196 | 21366999 | 22397605 | 23072817 | 24023983 | 26123354 | 27772856 | 28913300 | 29281282 | 30190982 |
| 19904556 | 21367218 | 22398160 | 23073003 | 24024725 | 26124713 | 27773398 | 28913594 | 29281283 | 30191611 |
| 19905049 | 21368126 | 22398298 | 23073186 | 24024806 | 26124991 | 27773917 | 28914487 | 29281288 | 30191801 |
| 19905758 | 21368273 | 22398438 | 23073302 | 24025623 | 26125242 | 27774244 | 28914562 | 29281298 | 30191968 |
| 19906042 | 21368803 | 22398698 | 23073623 | 24025858 | 26127276 | 27774394 | 28914793 | 29281300 | 30192238 |
| 19906309 | 21372011 | 22398755 | 23074374 | 24025893 | 26127369 | 27774990 | 28915172 | 29281302 | 30192782 |
| 19906622 | 21372934 | 22398868 | 23074509 | 24025907 | 26128419 | 27775068 | 28915847 | 29281307 | 30194877 |
| 19906698 | 21375216 | 22398909 | 23074984 | 24026022 | 26128965 | 27775171 | 28917271 | 29281318 | 30194998 |
| 19907264 | 21376349 | 22398947 | 23075143 | 24026319 | 26129410 | 27775364 | 28917358 | 29281405 | 30195512 |
| 19907349 | 21376594 | 22399171 | 23075582 | 24026450 | 26130012 | 27776229 | 28917506 | 29281417 | 30197099 |
| 19908091 | 21376862 | 22399374 | 23075614 | 24026803 | 26130778 | 27776250 | 28917684 | 29281429 | 30197703 |
| 19908122 | 21377839 | 22400348 | 23075662 | 24028040 | 26132539 | 27776591 | 28918287 | 29281431 | 30197820 |
| 19908214 | 21378121 | 22400403 | 23075977 | 24028757 | 26132805 | 27777077 | 28919667 | 29281436 | 30199930 |
| 19908220 | 21378136 | 22400737 | 23076272 | 24028864 | 26133821 | 27777566 | 28919691 | 29281442 | 30200364 |
| 19908313 | 21378354 | 22400937 | 23076823 | 24029444 | 26134117 | 27778731 | 28919699 | 29281445 | 30201194 |
| 19909117 | 21378857 | 22401704 | 23076843 | 24029964 | 26134283 | 27778882 | 28919731 | 29281453 | 30201307 |
| 19909362 | 21379477 | 22401828 | 23077515 | 24030314 | 26134947 | 27779556 | 28920272 | 29281472 | 30202058 |
| 19909707 | 21381957 | 22402266 | 23077530 | 24031930 | 26136495 | 27779704 | 28920431 | 29281474 | 30202334 |
| 19911033 | 21382005 | 22402415 | 23077840 | 24031971 | 26137561 | 27780840 | 28920714 | 29281481 | 30202615 |
| 19911125 | 21382256 | 22402822 | 23077916 | 24032179 | 26137984 | 27781038 | 28920867 | 29281511 | 30202746 |
| 19911498 | 21382298 | 22403034 | 23078143 | 24032195 | 26138194 | 27781277 | 28921196 | 29281521 | 30203368 |
| 19911690 | 21382379 | 22403369 | 23078518 | 24032605 | 26138549 | 27781869 | 28921575 | 29281535 | 30203469 |
| 19912408 | 21382539 | 22403693 | 23078614 | 24032903 | 26141820 | 27782473 | 28921608 | 29281555 | 30203993 |
| 19913470 | 21383502 | 22403792 | 23078618 | 24033434 | 26142291 | 27782543 | 28922606 | 29281557 | 30204198 |
| 19913656 | 21384458 | 22404020 | 23078769 | 24033959 | 26142716 | 27782617 | 28922791 | 29281559 | 30204271 |
| 19913692 | 21384731 | 22404491 | 23079112 | 24034801 | 26143156 | 27784343 | 28924281 | 29281566 | 30204886 |
| 19913761 | 21384733 | 22404598 | 23079343 | 24034810 | 26143715 | 27784565 | 28924960 | 29281568 | 30204974 |
| 19914469 | 21384916 | 22404639 | 23079633 | 24035853 | 26144389 | 27784639 | 28925267 | 29281584 | 30205196 |
| 19915007 | 21385302 | 22404727 | 23079802 | 24035946 | 26145166 | 27784836 | 28926207 | 29281585 | 30205222 |
| 19915177 | 21386085 | 22404900 | 23079998 | 24036256 | 26146212 | 27784888 | 28926330 | 29281595 | 30205337 |
| 19915715 | 21386965 | 22404958 | 23080067 | 24036630 | 26146223 | 27786149 | 28926830 | 29281600 | 30205369 |
| 19916165 | 21387082 | 22405178 | 23080267 | 24036953 | 26146835 | 27787210 | 28927672 | 29281603 | 30205865 |
| 19916294 | 21387800 | 22405183 | 23080346 | 24037359 | 26147063 | 27788007 | 28927856 | 29281607 | 30206193 |
| 19916849 | 21388583 | 22405342 | 23080497 | 24037515 | 26148384 | 27788663 | 28927915 | 29281609 | 30206876 |
| 19917642 | 21388686 | 22405482 | 23080529 | 24037902 | 26149429 | 27789122 | 28927990 | 29281618 | 30207339 |
| 19918036 | 21388875 | 22405731 | 23080568 | 24038671 | 26150954 | 27789125 | 28928231 | 29281625 | 30207972 |
| 19918230 | 21388878 | 22406153 | 23080706 | 24039490 | 26152463 | 27789194 | 28928334 | 29281642 | 30208490 |
| 19918280 | 21388957 | 22406184 | 23080795 | 24040119 | 26152968 | 27789503 | 28928453 | 29281657 | 30208593 |
| 19918781 | 21390437 | 22406655 | 23080833 | 24040648 | 26153968 | 27789864 | 28928760 | 29281666 | 30209114 |
| 19919427 | 21391048 | 22406865 | 23081031 | 24041171 | 26154266 | 27789972 | 28928816 | 29281668 | 30209270 |
| 19919919 | 21391349 | 22407420 | 23081221 | 24041693 | 26154586 | 27790254 | 28928920 | 29281670 | 30209403 |
| 19920418 | 21391471 | 22407866 | 23081376 | 24042334 | 26155902 | 27790479 | 28929180 | 29281671 | 30209522 |
| 19920500 | 21391679 | 22408111 | 23081711 | 24042396 | 26157345 | 27790543 | 28929889 | 29281687 | 30211064 |
| 19921334 | 21391896 | 22408115 | 23081863 | 24043038 | 26157568 | 27791798 | 28930141 | 29281688 | 30211080 |
| 19921485 | 21392200 | 22408119 | 23081996 | 24043057 | 26158587 | 27792518 | 28931408 | 29281694 | 30211555 |
| 19921924 | 21392441 | 22408548 | 23082094 | 24043415 | 26160080 | 27792695 | 28931768 | 29281695 | 30212372 |
| 19921957 | 21392911 | 22408722 | 23082163 | 24043506 | 26162899 | 27793062 | 28931955 | 29281696 | 30212496 |
| 19922578 | 21393116 | 22408932 | 23082248 | 24043509 | 26163017 | 27793380 | 28931979 | 29281698 | 30213016 |
| 19922725 | 21393575 | 22409094 | 23082311 | 24043605 | 26163263 | 27793627 | 28932038 | 29281702 | 30213117 |
| 19922762 | 21393986 | 22409659 | 23082745 | 24043950 | 26163984 | 27794041 | 28932781 | 29281710 | 30213640 |
| 19923112 | 21395632 | 22409700 | 23083155 | 24044158 | 26165195 | 27794132 | 28932899 | 29281712 | 30213997 |
| 19923338 | 21396341 | 22409839 | 23084106 | 24044377 | 26165910 | 27794539 | 28933386 | 29281716 | 30214894 |
| 19923708 | 21397260 | 22409984 | 23084309 | 24044574 | 26168028 | 27794928 | 28933805 | 29281723 | 30215367 |
| 19923739 | 21397500 | 22410059 | 23084525 | 24045057 | 26169348 | 27797240 | 28934181 | 29281730 | 30215388 |
| 19923755 | 21398078 | 22411350 | 23084860 | 24045092 | 26169624 | 27797578 | 28934926 | 29281731 | 30215489 |
| 19924183 | 21398139 | 22411529 | 23085175 | 24045488 | 26169881 | 27797657 | 28935228 | 29281737 | 30215910 |
| 19925185 | 21398819 | 22411720 | 23085227 | 24045551 | 26171197 | 27797739 | 28935849 | 29281749 | 30216155 |
| 19925546 | 21399000 | 22412430 | 23085633 | 24046625 | 26171244 | 27797746 | 28936154 | 29281751 | 30216354 |
| 19925726 | 21399390 | 22412699 | 23085675 | 24046779 | 26172150 | 27798849 | 28936470 | 29281757 | 30216537 |
| 19925800 | 21399531 | 22412755 | 23085700 | 24048091 | 26172519 | 27799004 | 28937110 | 29281769 | 30216766 |
| 19926277 | 21399662 | 22412919 | 23085839 | 24048234 | 26172751 | 27799387 | 28938125 | 29281783 | 30216936 |
| 19926654 | 21400230 | 22413039 | 23085927 | 24048673 | 26174059 | 27799549 | 28938244 | 29281786 | 30216946 |
| 19926812 | 21401855 | 22413698 | 23086118 | 24048758 | 26174535 | 27799828 | 28938290 | 29281806 | 30219318 |
| 19927489 | 21402357 | 22413832 | 23086316 | 24048800 | 26174914 | 27799962 | 28938602 | 29281811 | 30219343 |
| 19927613 | 21403421 | 22414045 | 23086399 | 24049175 | 26175146 | 27800182 | 28938893 | 29281818 | 30219461 |
| 19928174 | 21403472 | 22414358 | 23086575 | 24049662 | 26181110 | 27800382 | 28942350 | 29281821 | 30219554 |
| 19928179 | 21403694 | 22414428 | 23087262 | 24049786 | 26182264 | 27800815 | 28942427 | 29281822 | 30219604 |
| 19928849 | 21406496 | 22414445 | 23087415 | 24049971 | 26182470 | 27800995 | 28942457 | 29281829 | 30220965 |
| 19929279 | 21407103 | 22414528 | 23087578 | 24050239 | 26186453 | 27801156 | 28942683 | 29281834 | 30221802 |
| 19929439 | 21407270 | 22414762 | 23087656 | 24050940 | 26187264 | 27801174 | 28942891 | 29281838 | 30221922 |
| 19929553 | 21407770 | 22414862 | 23087692 | 24051566 | 26188082 | 27801224 | 28942934 | 29281844 | 30222000 |
| 19929877 | 21407879 | 22415537 | 23087863 | 24051593 | 26188437 | 27801687 | 28942968 | 29281856 | 30222687 |
| 19930083 | 21408024 | 22415564 | 23087871 | 24051794 | 26188724 | 27801847 | 28943877 | 29281862 | 30223324 |
| 19930401 | 21408417 | 22415689 | 23088220 | 24052210 | 26188999 | 27802553 | 28944255 | 29281868 | 30223385 |
| 19930531 | 21409273 | 22415803 | 23088560 | 24053256 | 26190065 | 27802680 | 28944519 | 29281872 | 30223390 |
| 19930781 | 21409463 | 22415867 | 23088799 | 24053516 | 26190144 | 27803282 | 28944586 | 29281904 | 30223443 |
| 19931537 | 21409885 | 22416203 | 23089120 | 24053764 | 26190300 | 27803409 | 28944701 | 29281916 | 30223609 |
| 19931583 | 21410037 | 22416319 | 23089357 | 24054449 | 26190420 | 27803627 | 28944738 | 29281920 | 30224649 |
| 19931836 | 21410131 | 22416483 | 23089402 | 24054820 | 26190875 | 27803663 | 28944802 | 29281926 | 30224693 |
| 19932044 | 21410250 | 22416508 | 23089594 | 24055236 | 26191139 | 27803999 | 28945096 | 29281932 | 30224805 |
| 19932132 | 21410404 | 22416648 | 23089860 | 24055951 | 26191418 | 27804526 | 28945699 | 29281936 | 30224944 |
| 19932481 | 21411034 | 22416785 | 23089889 | 24055984 | 26192537 | 27804869 | 28946294 | 29281937 | 30225343 |
| 19932552 | 21411465 | 22416990 | 23089989 | 24056142 | 26193090 | 27805523 | 28946439 | 29281940 | 30225529 |
| 19932735 | 21413668 | 22417005 | 23090254 | 24056448 | 26195049 | 27805879 | 28946748 | 29281942 | 30225694 |
| 19932977 | 21413990 | 22417012 | 23090347 | 24056861 | 26195578 | 27806792 | 28946903 | 29281949 | 30226874 |
| 19933025 | 21414563 | 22417139 | 23090834 | 24056908 | 26197956 | 27806930 | 28947102 | 29281971 | 30227895 |
| 19933153 | 21414850 | 22417638 | 23091132 | 24057335 | 26198944 | 27808176 | 28947270 | 29281988 | 30229191 |
| 19933283 | 21415671 | 22417836 | 23091171 | 24057939 | 26199954 | 27808208 | 28947638 | 29281989 | 30229370 |
| 19933681 | 21416152 | 22417869 | 23091331 | 24058140 | 26200516 | 27808986 | 28947645 | 29281998 | 30229469 |
| 19933956 | 21416273 | 22418005 | 23091728 | 24058343 | 26200705 | 27809103 | 28948060 | 29282000 | 30229813 |
| 19933995 | 21418867 | 22418172 | 23091795 | 24058773 | 26201644 | 27809270 | 28949058 | 29282006 | 30231035 |
| 19934380 | 21420825 | 22418638 | 23092265 | 24059007 | 26202072 | 27809461 | 28949279 | 29282010 | 30231119 |
| 19935062 | 21420867 | 22418813 | 23092420 | 24059137 | 26202552 | 27810733 | 28949962 | 29282012 | 30231883 |
| 19935192 | 21421353 | 22419031 | 23092594 | 24059222 | 26203007 | 27810736 | 28950888 | 29282013 | 30231945 |
| 19935618 | 21421439 | 22419226 | 23092760 | 24059651 | 26203324 | 27811495 | 28950898 | 29282018 | 30231950 |
| 19935649 | 21421870 | 22419332 | 23092842 | 24059882 | 26203395 | 27811789 | 28950919 | 29282022 | 30232134 |
| 19936582 | 21422061 | 22419384 | 23093291 | 24060134 | 26203785 | 27812062 | 28951318 | 29282031 | 30233354 |
| 19936837 | 21422131 | 22419550 | 23094933 | 24060274 | 26204116 | 27812184 | 28952004 | 29282057 | 30233379 |
| 19937249 | 21422221 | 22420102 | 23095144 | 24060313 | 26205644 | 27812273 | 28952143 | 29282060 | 30233717 |
| 19937272 | 21422312 | 22420122 | 23095447 | 24060885 | 26205653 | 27812422 | 28952172 | 29282068 | 30234328 |
| 19937278 | 21422752 | 22420170 | 23095784 | 24061246 | 26206508 | 27812945 | 28952375 | 29282079 | 30234805 |
| 19937334 | 21422957 | 22420195 | 23095862 | 24061415 | 26206685 | 27813153 | 28952711 | 29282093 | 30235073 |
| 19937600 | 21423354 | 22420219 | 23095990 | 24062016 | 26209466 | 27813158 | 28952730 | 29282108 | 30235129 |
| 19938040 | 21423376 | 22420374 | 23096083 | 24062183 | 26209679 | 27813523 | 28953185 | 29282113 | 30235295 |
| 19939098 | 21424270 | 22420656 | 23096121 | 24062349 | 26210347 | 27814638 | 28953241 | 29282120 | 30235492 |
| 19939099 | 21424607 | 22420689 | 23096141 | 24062659 | 26211700 | 27814773 | 28953919 | 29282140 | 30235515 |
| 19939182 | 21424942 | 22420802 | 23096999 | 24062736 | 26212533 | 27814978 | 28954494 | 29282143 | 30235548 |
| 19939418 | 21425089 | 22422038 | 23097007 | 24063318 | 26214355 | 27815532 | 28954757 | 29282150 | 30235959 |
| 19939618 | 21425592 | 22422361 | 23097138 | 24063542 | 26215643 | 27815771 | 28954962 | 29282161 | 30236015 |
| 19940251 | 21426780 | 22422716 | 23097367 | 24063780 | 26216750 | 27816079 | 28955102 | 29282176 | 30236058 |
| 19940334 | 21427137 | 22423025 | 23098035 | 24063928 | 26217009 | 27816132 | 28955337 | 29282178 | 30236106 |
| 19940783 | 21427902 | 22423065 | 23098301 | 24064904 | 26220406 | 27818379 | 28955495 | 29282179 | 30236320 |
| 19941353 | 21428341 | 22423157 | 23098503 | 24064975 | 26220509 | 27819452 | 28955888 | 29282191 | 30236429 |
| 19941585 | 21430240 | 22423205 | 23098551 | 24065093 | 26221556 | 27820691 | 28956131 | 29282197 | 30237057 |
| 19941852 | 21430285 | 22423339 | 23098659 | 24065899 | 26223722 | 27820787 | 28956792 | 29282204 | 30237226 |
| 19942141 | 21431066 | 22423484 | 23098818 | 24065911 | 26225332 | 27820811 | 28956968 | 29282206 | 30237865 |
| 19942162 | 21431754 | 22423507 | 23098891 | 24066259 | 26225666 | 27820986 | 28957134 | 29282219 | 30238092 |
| 19942376 | 21432158 | 22423532 | 23098898 | 24067920 | 26227822 | 27822669 | 28957536 | 29282225 | 30238098 |
| 19942398 | 21432680 | 22423692 | 23099004 | 24068815 | 26227995 | 27822859 | 28957583 | 29282241 | 30238479 |
| 19942554 | 21432960 | 22423939 | 23099088 | 24069820 | 26228964 | 27823033 | 28958011 | 29282244 | 30238616 |
| 19942741 | 21433385 | 22424069 | 23099166 | 24069880 | 26229249 | 27824231 | 28958467 | 29282246 | 30238623 |
| 19943370 | 21433751 | 22424172 | 23099505 | 24070375 | 26229929 | 27824726 | 28958902 | 29282248 | 30238711 |
| 19943819 | 21433898 | 22424747 | 23099571 | 24070737 | 26232427 | 27824892 | 28959089 | 29282265 | 30239157 |
| 19944047 | 21435503 | 22424773 | 23100199 | 24071051 | 26232654 | 27825365 | 28959403 | 29282270 | 30239424 |
| 19944523 | 21435998 | 22424811 | 23100269 | 24071149 | 26233213 | 27825375 | 28959547 | 29282273 | 30239671 |
| 19944667 | 21436182 | 22425004 | 23100615 | 24071289 | 26235911 | 27826173 | 28959590 | 29282275 | 30240295 |
| 19945299 | 21437174 | 22425157 | 23100874 | 24071298 | 26236154 | 27826675 | 28960161 | 29282305 | 30240465 |
| 19945673 | 21437342 | 22425338 | 23101020 | 24071708 | 26237535 | 27826862 | 28960199 | 29282307 | 30241190 |
| 19947144 | 21438341 | 22425630 | 23101290 | 24071740 | 26238408 | 27827028 | 28961720 | 29282308 | 30241675 |
| 19947482 | 21439510 | 22426737 | 23101341 | 24071952 | 26239322 | 27828831 | 28961901 | 29282321 | 30243107 |
| 19947828 | 21440608 | 22426861 | 23102085 | 24072225 | 26239446 | 27829250 | 28961925 | 29282328 | 30243186 |
| 19947988 | 21441068 | 22427330 | 23102095 | 24072826 | 26240120 | 27829890 | 28962278 | 29282330 | 30245898 |
| 19948173 | 21441176 | 22427492 | 23102214 | 24073072 | 26240267 | 27830120 | 28962447 | 29282336 | 30247117 |
| 19948417 | 21441554 | 22427736 | 23102438 | 24073677 | 26240328 | 27830359 | 28962577 | 29282344 | 30247148 |
| 19948483 | 21441750 | 22428059 | 23102536 | 24073774 | 26243683 | 27830814 | 28962844 | 29282354 | 30247493 |
| 19948803 | 21441819 | 22428106 | 23102602 | 24073894 | 26245545 | 27831024 | 28962948 | 29282355 | 30247779 |
| 19948821 | 21442129 | 22428468 | 23102772 | 24074316 | 26245838 | 27832108 | 28963043 | 29282362 | 30247953 |
| 19949261 | 21442235 | 22428478 | 23102834 | 24074511 | 26246159 | 27832151 | 28963455 | 29282379 | 30249118 |
| 19949703 | 21442376 | 22429079 | 23102886 | 24074628 | 26246790 | 27832962 | 28963485 | 29282393 | 30249229 |
| 19950103 | 21443223 | 22429257 | 23102930 | 24074702 | 26246884 | 27833349 | 28963645 | 29282394 | 30249475 |
| 19950151 | 21444096 | 22429410 | 23103007 | 24075595 | 26249274 | 27834250 | 28963686 | 29282399 | 30250053 |
| 19950270 | 21445397 | 22429436 | 23103635 | 24075637 | 26249922 | 27834356 | 28964374 | 29282401 | 30250093 |
| 19950297 | 21446548 | 22429438 | 23103685 | 24075657 | 26251399 | 27834430 | 28964791 | 29282406 | 30250108 |
| 19950515 | 21446640 | 22429462 | 23103969 | 24076354 | 26251808 | 27834768 | 28964878 | 29282409 | 30250265 |
| 19950707 | 21447032 | 22429476 | 23104116 | 24077062 | 26252862 | 27834851 | 28964976 | 29282417 | 30251048 |
| 19950726 | 21447532 | 22429865 | 23104210 | 24077418 | 26252987 | 27835545 | 28965688 | 29282418 | 30251193 |
| 19952209 | 21447581 | 22430094 | 23104246 | 24078055 | 26253067 | 27835629 | 28965708 | 29282447 | 30251195 |
| 19952646 | 21449627 | 22430931 | 23104559 | 24078154 | 26253115 | 27835943 | 28965755 | 29282456 | 30251390 |
| 19952902 | 21452209 | 22430989 | 23104600 | 24078285 | 26253594 | 27835986 | 28965859 | 29282465 | 30252737 |
| 19953277 | 21452829 | 22431104 | 23104744 | 24078300 | 26253797 | 27836086 | 28965975 | 29282469 | 30253631 |
| 19953358 | 21453155 | 22431409 | 23104861 | 24078526 | 26255391 | 27836103 | 28966566 | 29282482 | 30253694 |
| 19953684 | 21453771 | 22431445 | 23104903 | 24079556 | 26255472 | 27836503 | 28967399 | 29282491 | 30254686 |
| 19954178 | 21453899 | 22431776 | 23105192 | 24079576 | 26255520 | 27836785 | 28967871 | 29282492 | 30255107 |
| 19954956 | 21453924 | 22433142 | 23105261 | 24079996 | 26260091 | 27837772 | 28968058 | 29282495 | 30255152 |
| 19955153 | 21454596 | 22433446 | 23105628 | 24080051 | 26260509 | 27837921 | 28968261 | 29282505 | 30255931 |
| 19955514 | 21456169 | 22433747 | 23105659 | 24080210 | 26261678 | 27838016 | 28968683 | 29282515 | 30256126 |
| 19955889 | 21456532 | 22433986 | 23105737 | 24080306 | 26262360 | 27839117 | 28968815 | 29282518 | 30256570 |
| 19955931 | 21457118 | 22434379 | 23105997 | 24080457 | 26263442 | 27839583 | 28969008 | 29282529 | 30257575 |
| 19956443 | 21457574 | 22434771 | 23106594 | 24080796 | 26263674 | 27839957 | 28969446 | 29282531 | 30257826 |
| 19957099 | 21458848 | 22435181 | 23106926 | 24081692 | 26265326 | 27840783 | 28969738 | 29282549 | 30258248 |
| 19957393 | 21459743 | 22436501 | 23106928 | 24083321 | 26266447 | 27841014 | 28970539 | 29282566 | 30258299 |
| 19957582 | 21459860 | 22436579 | 23107120 | 24083525 | 26266555 | 27841446 | 28970880 | 29282572 | 30258669 |
| 19958469 | 21460046 | 22436948 | 23107159 | 24083543 | 26266594 | 27841590 | 28971002 | 29282573 | 30258993 |
| 19958637 | 21460956 | 22436953 | 23107505 | 24083893 | 26267245 | 27842595 | 28971605 | 29282574 | 30259619 |
| 19958680 | 21462249 | 22437501 | 23107602 | 24084871 | 26268231 | 27842805 | 28971649 | 29282585 | 30259750 |
| 19958785 | 21463406 | 22437530 | 23108217 | 24085512 | 26269007 | 27842924 | 28973327 | 29282609 | 30259920 |
| 19958852 | 21463497 | 22437831 | 23108473 | 24085653 | 26269619 | 27843168 | 28973376 | 29282622 | 30260257 |
| 19959083 | 21464826 | 22438229 | 23108568 | 24085891 | 26271624 | 27843452 | 28973450 | 29282627 | 30260441 |
| 19959323 | 21465096 | 22438354 | 23109316 | 24086289 | 26271833 | 27843822 | 28973821 | 29282637 | 30260560 |
| 19959571 | 21466476 | 22438837 | 23109429 | 24086929 | 26272140 | 27844016 | 28973956 | 29282638 | 30260663 |
| 19959821 | 21467245 | 22438862 | 23110110 | 24087054 | 26273145 | 27844885 | 28974226 | 29282644 | 30262297 |
| 19960378 | 21467307 | 22439077 | 23110237 | 24088084 | 26273393 | 27844979 | 28974448 | 29282646 | 30262954 |
| 19960515 | 21467345 | 22439078 | 23110243 | 24088164 | 26274185 | 27844987 | 28974452 | 29282662 | 30263507 |
| 19961392 | 21468046 | 22439378 | 23110269 | 24088591 | 26274599 | 27845929 | 28975060 | 29282666 | 30263814 |
| 19961401 | 21468635 | 22439648 | 23110284 | 24088658 | 26275070 | 27846089 | 28975202 | 29282679 | 30264322 |
| 19961820 | 21468881 | 22440146 | 23110466 | 24088666 | 26276450 | 27847345 | 28975764 | 29282691 | 30265530 |
| 19962235 | 21469282 | 22440279 | 23110955 | 24088950 | 26276924 | 27847711 | 28976025 | 29282693 | 30266038 |
| 19962282 | 21469348 | 22440295 | 23111268 | 24088989 | 26276972 | 27848999 | 28976785 | 29282707 | 30266075 |
| 19962447 | 21469942 | 22440730 | 23111356 | 24090216 | 26277620 | 27849027 | 28976956 | 29282714 | 30266096 |
| 19962579 | 21470202 | 22440846 | 23111456 | 24090661 | 26278230 | 27849106 | 28977075 | 29282721 | 30266370 |
| 19962590 | 21470203 | 22441088 | 23111932 | 24091050 | 26280316 | 27849459 | 28977316 | 29282726 | 30266783 |
| 19963021 | 21470345 | 22441221 | 23112045 | 24091588 | 26280473 | 27849676 | 28977345 | 29282729 | 30267603 |
| 19963032 | 21470573 | 22441554 | 23112273 | 24091686 | 26282784 | 27850012 | 28977440 | 29282730 | 30268489 |
| 19963130 | 21471354 | 22441603 | 23112449 | 24091755 | 26283053 | 27850701 | 28977837 | 29282744 | 30268751 |
| 19965529 | 21471762 | 22442251 | 23112624 | 24091940 | 26283237 | 27850766 | 28977916 | 29282749 | 30268949 |
| 19965810 | 21472407 | 22442313 | 23112769 | 24092046 | 26283560 | 27851176 | 28977966 | 29282757 | 30269001 |
| 19965838 | 21472584 | 22442757 | 23112839 | 24092775 | 26287153 | 27852141 | 28978182 | 29282780 | 30269127 |
| 19966606 | 21473213 | 22442815 | 23112851 | 24092939 | 26289151 | 27852712 | 28978642 | 29282784 | 30271303 |
| 19966686 | 21473351 | 22443607 | 23113223 | 24092975 | 26290359 | 27852722 | 28978853 | 29282793 | 30271389 |
| 19966923 | 21473622 | 22444471 | 23113299 | 24092989 | 26291002 | 27853095 | 28979181 | 29282804 | 30272188 |
| 19967168 | 21473935 | 22444481 | 23113381 | 24093178 | 26291476 | 27853367 | 28979262 | 29282809 | 30272593 |
| 19967295 | 21476833 | 22444876 | 23113395 | 24093932 | 26291507 | 27853683 | 28979302 | 29282810 | 30272963 |
| 19967423 | 21478273 | 22445077 | 23113457 | 24094156 | 26291698 | 27853996 | 28979474 | 29282820 | 30273180 |
| 19967887 | 21478497 | 22445139 | 23113480 | 24094474 | 26291956 | 27854047 | 28979605 | 29282826 | 30273384 |
| 19967889 | 21479080 | 22445175 | 23113537 | 24094977 | 26292076 | 27854322 | 28979644 | 29282841 | 30273767 |
| 19967997 | 21479778 | 22445230 | 23113722 | 24095210 | 26292227 | 27854844 | 28979718 | 29282847 | 30273907 |
| 19968293 | 21480072 | 22445783 | 23113773 | 24096024 | 26292295 | 27855152 | 28980056 | 29282848 | 30274039 |
| 19968607 | 21480241 | 22446039 | 23113977 | 24097140 | 26292582 | 27855154 | 28981116 | 29282852 | 30274238 |
| 19968695 | 21480342 | 22446074 | 23114099 | 24097272 | 26292864 | 27855501 | 28981732 | 29282867 | 30274419 |
| 19968953 | 21483511 | 22446467 | 23114230 | 24097809 | 26293182 | 27855574 | 28982336 | 29282874 | 30274511 |
| 19968987 | 21483677 | 22446486 | 23114290 | 24098076 | 26294582 | 27855814 | 28982540 | 29282884 | 30274883 |
| 19969035 | 21483797 | 22446533 | 23114312 | 24098682 | 26295182 | 27856659 | 28982908 | 29282894 | 30275704 |
| 19970232 | 21485144 | 22446709 | 23114355 | 24098734 | 26296255 | 27857783 | 28982946 | 29282902 | 30276019 |
| 19970475 | 21486056 | 22446886 | 23114391 | 24099463 | 26296358 | 27857891 | 28983037 | 29282915 | 30276760 |
| 19970507 | 21486610 | 22446976 | 23114459 | 24099915 | 26299583 | 27858679 | 28983980 | 29282919 | 30276914 |
| 19970555 | 21487044 | 22447126 | 23114661 | 24099961 | 26299970 | 27858914 | 28984014 | 29282920 | 30277355 |
| 19970650 | 21487290 | 22447301 | 23114795 | 24100022 | 26300976 | 27859199 | 28984224 | 29282926 | 30277858 |
| 19970978 | 21487356 | 22447577 | 23115067 | 24100034 | 26302317 | 27859972 | 28984474 | 29282928 | 30278188 |
| 19971043 | 21488014 | 22447984 | 23115134 | 24100474 | 26302817 | 27860583 | 28984932 | 29282930 | 30278409 |
| 19971289 | 21488762 | 22448420 | 23115175 | 24100864 | 26305132 | 27860655 | 28985305 | 29282942 | 30278531 |
| 19971309 | 21489399 | 22449516 | 23115336 | 24100998 | 26305164 | 27860697 | 28986176 | 29282950 | 30278761 |
| 19972258 | 21490137 | 22449779 | 23115619 | 24101351 | 26307351 | 27861305 | 28986213 | 29282959 | 30279056 |
| 19972290 | 21490384 | 22450015 | 23116178 | 24101359 | 26308614 | 27861362 | 28986318 | 29282963 | 30279413 |
| 19972948 | 21491397 | 22450237 | 23116189 | 24101381 | 26308770 | 27861635 | 28986662 | 29282964 | 30280084 |
| 19973260 | 21492516 | 22450238 | 23116207 | 24101636 | 26308812 | 27862007 | 28987285 | 29282989 | 30280389 |
| 19974195 | 21493459 | 22450473 | 23116329 | 24102224 | 26308950 | 27862117 | 28987443 | 29282995 | 30280444 |
| 19975116 | 21493666 | 22450612 | 23116405 | 24103204 | 26308994 | 27862370 | 28987768 | 29282996 | 30280692 |
| 19976134 | 21494103 | 22450641 | 23116546 | 24103216 | 26309972 | 27862551 | 28988025 | 29283000 | 30281032 |
| 19976490 | 21495964 | 22451083 | 23116669 | 24103785 | 26310991 | 27864247 | 28988270 | 29283005 | 30281041 |
| 19976669 | 21496628 | 22451196 | 23117948 | 24103925 | 26312237 | 27865130 | 28988394 | 29283011 | 30281151 |
| 19977660 | 21496753 | 22451231 | 23118102 | 24104235 | 26312289 | 27865379 | 28988458 | 29283021 | 30281365 |
| 19977698 | 21496916 | 22451292 | 23118448 | 24104735 | 26312650 | 27866071 | 28988691 | 29283022 | 30281402 |
| 19977930 | 21497153 | 22451594 | 23118512 | 24104761 | 26314103 | 27866653 | 28988838 | 29283028 | 30281514 |
| 19978027 | 21497314 | 22451782 | 23119227 | 24104784 | 26314556 | 27867213 | 28989016 | 29283037 | 30282022 |
| 19978156 | 21497378 | 22451865 | 23119271 | 24104917 | 26314584 | 27867405 | 28989037 | 29283038 | 30282396 |
| 19978804 | 21498885 | 22451937 | 23119418 | 24105147 | 26315377 | 27867485 | 28989142 | 29283074 | 30282873 |
| 19978807 | 21500233 | 22451948 | 23119477 | 24105153 | 26315545 | 27868295 | 28989364 | 29283090 | 30283202 |
| 19978931 | 21500425 | 22452310 | 23119740 | 24105519 | 26315816 | 27868524 | 28989494 | 29283092 | 30283250 |
| 19979673 | 21500475 | 22452348 | 23119808 | 24105966 | 26317102 | 27868833 | 28989674 | 29283100 | 30284461 |
| 19980191 | 21501186 | 22452544 | 23119837 | 24106970 | 26317544 | 27869025 | 28990187 | 29283105 | 30284515 |
| 19980215 | 21501880 | 22452672 | 23120085 | 24107191 | 26318366 | 27869538 | 28990464 | 29283115 | 30284888 |
| 19980247 | 21502196 | 22453474 | 23120125 | 24107719 | 26318727 | 27869712 | 28990820 | 29283116 | 30284994 |
| 19980395 | 21502927 | 22453514 | 23120614 | 24107856 | 26319359 | 27870219 | 28991119 | 29283117 | 30285255 |
| 19980512 | 21503122 | 22453709 | 23120766 | 24108088 | 26320475 | 27870789 | 28992090 | 29283126 | 30285795 |
| 19980526 | 21504873 | 22453949 | 23120887 | 24108127 | 26320943 | 27871204 | 28992185 | 29283156 | 30286004 |
| 19981180 | 21505305 | 22454069 | 23120928 | 24108286 | 26322612 | 27872102 | 28992242 | 29283167 | 30286641 |
| 19981774 | 21505340 | 22454146 | 23121007 | 24108588 | 26322715 | 27872775 | 28992297 | 29283173 | 30287168 |
| 19982724 | 21505714 | 22454319 | 23121157 | 24109108 | 26323740 | 27874344 | 28992349 | 29283179 | 30287957 |
| 19983636 | 21506637 | 22454641 | 23121776 | 24109722 | 26324884 | 27874651 | 28992719 | 29283187 | 30288059 |
| 19983640 | 21507642 | 22454962 | 23121872 | 24110369 | 26325375 | 27874965 | 28993638 | 29283210 | 30289165 |
| 19983660 | 21509358 | 22455568 | 23121968 | 24110580 | 26327079 | 27875396 | 28993756 | 29283212 | 30290285 |
| 19984035 | 21509365 | 22456741 | 23122188 | 24111511 | 26327547 | 27876554 | 28994051 | 29283216 | 30291483 |
| 19985331 | 21509534 | 22456753 | 23122264 | 24112640 | 26327910 | 27876885 | 28994181 | 29283221 | 30291716 |
| 19985753 | 21509759 | 22457683 | 23122320 | 24113201 | 26329181 | 27877475 | 28995280 | 29283223 | 30292327 |
| 19985885 | 21509964 | 22457852 | 23122493 | 24113388 | 26329852 | 27878077 | 28995419 | 29283229 | 30292731 |
| 19986052 | 21510386 | 22457941 | 23122514 | 24114040 | 26330032 | 27879063 | 28995460 | 29283232 | 30293125 |
| 19986339 | 21510444 | 22458084 | 23122617 | 24114989 | 26330390 | 27879421 | 28995563 | 29283238 | 30293646 |
| 19986551 | 21510675 | 22458404 | 23122629 | 24115191 | 26331552 | 27879845 | 28996351 | 29283242 | 30294100 |
| 19986600 | 21511612 | 22458584 | 23122858 | 24115229 | 26333397 | 27879997 | 28996522 | 29283249 | 30294632 |
| 19986781 | 21511816 | 22458818 | 23123076 | 24115264 | 26333933 | 27880139 | 28996610 | 29283258 | 30294703 |
| 19986964 | 21512941 | 22459000 | 23123472 | 24115309 | 26336407 | 27881026 | 28996973 | 29283267 | 30295372 |
| 19987077 | 21513069 | 22459484 | 23123575 | 24115359 | 26336480 | 27881716 | 28997081 | 29283273 | 30295957 |
| 19987137 | 21513499 | 22459833 | 23124459 | 24116268 | 26338182 | 27881913 | 28998824 | 29283282 | 30296554 |
| 19987209 | 21514168 | 22460126 | 23124493 | 24116454 | 26339527 | 27881983 | 28998839 | 29283284 | 30297211 |
| 19987399 | 21514619 | 22460336 | 23124620 | 24117142 | 26341771 | 27882481 | 28999044 | 29283288 | 30300220 |
| 19987691 | 21515689 | 22460439 | 23124680 | 24117187 | 26341893 | 27882784 | 28999540 | 29283291 | 30300552 |
| 19987957 | 21515755 | 22460872 | 23124991 | 24118062 | 26342444 | 27882786 | 28999814 | 29283296 | 30300870 |
| 19988288 | 21516562 | 22461196 | 23125460 | 24118207 | 26343305 | 27882913 | 29000678 | 29283299 | 30301247 |
| 19988742 | 21516727 | 22461521 | 23126026 | 24118287 | 26343996 | 27883220 | 29001006 | 29283302 | 30301491 |
| 19988839 | 21516944 | 22461537 | 23126415 | 24118399 | 26344745 | 27884084 | 29001018 | 29283305 | 30301736 |
| 19988998 | 21517283 | 22461698 | 23126681 | 24119310 | 26345177 | 27884507 | 29001103 | 29283306 | 30302357 |
| 19989293 | 21517345 | 22461916 | 23126881 | 24119435 | 26345668 | 27884764 | 29001409 | 29283331 | 30302603 |
| 19989431 | 21517523 | 22461969 | 23127559 | 24119509 | 26345765 | 27884909 | 29001853 | 29283340 | 30302622 |
| 19989456 | 21517762 | 22461995 | 23127775 | 24120333 | 26345869 | 27885209 | 29002191 | 29283343 | 30302885 |
| 19989515 | 21518329 | 22462011 | 23128254 | 24120569 | 26349146 | 27885588 | 29002892 | 29283345 | 30303465 |
| 19989772 | 21518346 | 22462031 | 23128414 | 24120856 | 26349453 | 27885991 | 29002909 | 29283361 | 30303674 |
| 19990100 | 21518464 | 22462048 | 23128460 | 24121572 | 26352448 | 27886184 | 29002920 | 29283364 | 30304311 |
| 19990747 | 21518803 | 22462103 | 23128623 | 24121602 | 26353294 | 27886225 | 29002986 | 29283399 | 30304507 |
| 19990964 | 21519389 | 22462128 | 23128704 | 24122172 | 26353428 | 27886653 | 29003011 | 29283416 | 30305365 |
| 19991149 | 21519992 | 22462565 | 23128784 | 24122890 | 26354113 | 27887878 | 29003781 | 29283418 | 30305408 |
| 19991283 | 21520205 | 22462929 | 23128799 | 24123489 | 26354677 | 27888052 | 29004015 | 29283420 | 30305736 |
| 19991477 | 21520338 | 22463306 | 23128880 | 24123792 | 26355094 | 27888213 | 29004044 | 29283431 | 30305899 |
| 19991951 | 21523267 | 22463429 | 23129053 | 24124344 | 26355588 | 27888934 | 29004247 | 29283434 | 30306124 |
| 19992128 | 21524455 | 22464054 | 23129270 | 24124597 | 26355870 | 27889316 | 29004913 | 29283443 | 30306665 |
| 19992136 | 21525223 | 22464309 | 23129322 | 24126322 | 26355888 | 27889769 | 29005146 | 29283444 | 30307041 |
| 19992261 | 21525929 | 22464649 | 23129512 | 24126630 | 26356623 | 27889998 | 29005449 | 29283446 | 30307545 |
| 19993071 | 21525968 | 22464697 | 23129756 | 24127139 | 26357352 | 27890095 | 29005462 | 29283455 | 30309092 |
| 19993282 | 21526713 | 22465537 | 23130234 | 24127904 | 26357428 | 27890103 | 29005825 | 29283473 | 30309491 |
| 19993667 | 21527006 | 22466203 | 23130441 | 24128065 | 26357621 | 27890124 | 29006468 | 29283482 | 30309605 |
| 19994216 | 21527156 | 22466376 | 23130482 | 24128503 | 26358005 | 27890582 | 29006717 | 29283497 | 30309664 |
| 19994300 | 21527890 | 22466524 | 23130637 | 24128565 | 26358079 | 27890758 | 29007405 | 29283505 | 30310371 |
| 19994336 | 21528293 | 22466635 | 23131192 | 24128661 | 26358404 | 27891095 | 29008064 | 29283506 | 30310990 |
| 19996016 | 21529206 | 22466834 | 23131261 | 24129274 | 26361733 | 27891241 | 29008197 | 29283509 | 30311025 |
| 19996090 | 21530227 | 22466914 | 23131388 | 24129394 | 26362719 | 27891304 | 29008641 | 29283517 | 30311422 |
| 19996451 | 21531446 | 22466976 | 23131758 | 24129622 | 26364236 | 27891484 | 29008827 | 29283520 | 30312321 |
| 19996676 | 21532212 | 22467087 | 23131873 | 24129897 | 26364495 | 27892115 | 29010452 | 29283524 | 30312504 |
| 19996908 | 21533806 | 22467701 | 23131948 | 24129994 | 26364946 | 27892495 | 29010863 | 29283539 | 30312562 |
| 19997390 | 21534536 | 22467766 | 23131950 | 24130175 | 26365583 | 27892587 | 29010905 | 29283540 | 30313274 |
| 19997868 | 21535124 | 22467847 | 23131957 | 24131530 | 26366292 | 27892789 | 29010943 | 29283541 | 30313318 |
| 19998008 | 21535206 | 22467855 | 23132015 | 24131729 | 26366557 | 27893308 | 29010994 | 29283543 | 30313497 |
| 19998107 | 21536028 | 22468082 | 23132026 | 24132042 | 26366881 | 27893527 | 29011522 | 29283555 | 30313999 |
| 19998582 | 21536615 | 22468140 | 23132310 | 24132198 | 26368071 | 27894211 | 29011983 | 29283561 | 30315012 |
| 19998837 | 21536802 | 22468259 | 23132394 | 24133177 | 26368512 | 27894747 | 29012320 | 29283568 | 30315413 |
| 20000266 | 21536875 | 22468381 | 23132472 | 24133689 | 26369211 | 27896218 | 29012601 | 29283570 | 30315696 |
| 20000280 | 21538233 | 22468618 | 23132520 | 24133803 | 26369568 | 27896419 | 29012626 | 29283575 | 30317772 |
| 20000793 | 21538350 | 22468686 | 23132686 | 24134702 | 26369734 | 27896713 | 29013402 | 29283576 | 30318211 |
| 20002153 | 21538633 | 22468720 | 23132863 | 24135016 | 26370159 | 27897367 | 29013738 | 29283578 | 30318363 |
| 20002427 | 21539114 | 22468767 | 23133199 | 24135124 | 26372996 | 27897628 | 29013860 | 29283580 | 30318475 |
| 20002640 | 21539152 | 22468813 | 23133271 | 24135141 | 26373832 | 27898020 | 29014883 | 29283584 | 30318550 |
| 20002699 | 21539186 | 22469218 | 23133918 | 24136014 | 26375152 | 27898094 | 29015400 | 29283585 | 30319114 |
| 20003176 | 21539980 | 22469277 | 23134278 | 24137991 | 26375204 | 27898188 | 29015511 | 29283588 | 30319653 |
| 20003578 | 21541830 | 22469324 | 23134752 | 24138494 | 26376497 | 27898606 | 29016516 | 29283593 | 30319837 |
| 20004037 | 21541838 | 22469386 | 23135125 | 24138502 | 26376540 | 27898986 | 29016875 | 29283599 | 30320075 |
| 20004578 | 21542152 | 22469550 | 23135215 | 24138702 | 26376617 | 27899429 | 29017336 | 29283601 | 30320143 |
| 20005365 | 21544298 | 22470075 | 23135495 | 24139551 | 26379010 | 27899749 | 29018035 | 29283611 | 30320657 |
| 20005538 | 21545191 | 22470869 | 23135519 | 24139558 | 26379553 | 27899996 | 29018036 | 29283617 | 30322057 |
| 20006127 | 21545438 | 22471099 | 23135579 | 24139936 | 26380428 | 27900160 | 29018068 | 29283627 | 30322077 |
| 20007698 | 21547199 | 22471298 | 23135591 | 24140888 | 26380664 | 27901365 | 29018528 | 29283637 | 30322620 |
| 20007871 | 21547266 | 22471547 | 23135751 | 24140969 | 26382476 | 27903101 | 29018534 | 29283639 | 30323092 |
| 20008460 | 21547458 | 22471674 | 23136075 | 24141111 | 26382976 | 27903345 | 29018877 | 29283651 | 30323234 |
| 20009225 | 21548225 | 22471705 | 23136101 | 24141164 | 26383332 | 27903437 | 29018954 | 29283666 | 30323504 |
| 20009371 | 21548751 | 22471738 | 23136302 | 24141448 | 26383396 | 27903534 | 29018990 | 29283680 | 30323515 |
| 20009409 | 21549219 | 22472628 | 23136549 | 24141459 | 26383578 | 27903842 | 29019396 | 29283682 | 30323648 |
| 20009774 | 21549730 | 22473176 | 23136748 | 24141751 | 26384548 | 27904335 | 29020120 | 29283687 | 30323853 |
| 20010100 | 21550117 | 22473178 | 23136851 | 24142235 | 26384632 | 27904699 | 29020476 | 29283693 | 30323903 |
| 20011233 | 21550510 | 22473627 | 23136858 | 24143572 | 26391315 | 27905815 | 29020508 | 29283710 | 30324812 |
| 20011396 | 21551349 | 22473642 | 23136882 | 24144917 | 26391335 | 27907025 | 29020782 | 29283714 | 30324875 |
| 20011715 | 21552385 | 22473702 | 23137033 | 24145239 | 26392603 | 27907036 | 29021372 | 29283729 | 30324912 |
| 20012372 | 21552530 | 22473760 | 23137215 | 24145781 | 26393320 | 27907719 | 29022925 | 29283732 | 30325736 |
| 20012667 | 21552856 | 22473911 | 23137279 | 24145854 | 26393379 | 27908661 | 29023053 | 29283740 | 30326182 |
| 20013027 | 21554762 | 22474542 | 23137314 | 24146651 | 26393967 | 27908728 | 29023351 | 29283746 | 30326369 |
| 20013178 | 21555668 | 22474635 | 23137623 | 24147139 | 26394807 | 27909022 | 29024592 | 29283756 | 30327125 |
| 20014031 | 21555969 | 22475207 | 23137656 | 24147160 | 26395166 | 27909376 | 29024802 | 29283763 | 30327533 |
| 20014654 | 21556910 | 22475246 | 23137761 | 24147661 | 26395397 | 27909480 | 29024898 | 29283775 | 30328300 |
| 20014655 | 21557098 | 22475387 | 23137773 | 24147758 | 26398681 | 27909749 | 29025071 | 29283776 | 30329371 |
| 20014712 | 21557544 | 22475615 | 23137843 | 24147783 | 26399026 | 27910770 | 29026263 | 29283790 | 30330195 |
| 20015041 | 21557552 | 22475630 | 23137986 | 24148319 | 26403525 | 27910998 | 29026654 | 29283794 | 30330262 |
| 20015167 | 21557592 | 22476036 | 23138100 | 24148556 | 26403734 | 27911191 | 29026829 | 29283795 | 30330464 |
| 20015647 | 21557607 | 22476444 | 23138106 | 24148676 | 26404133 | 27912472 | 29026991 | 29283796 | 30330862 |
| 20015726 | 21557617 | 22476663 | 23138108 | 24149214 | 26405437 | 27912852 | 29026995 | 29283803 | 30332691 |
| 20015810 | 21558450 | 22476676 | 23138819 | 24149258 | 26405690 | 27914654 | 29027399 | 29283804 | 30332693 |
| 20016142 | 21558452 | 22477556 | 23138925 | 24149269 | 26407142 | 27914711 | 29027757 | 29283827 | 30332908 |
| 20016757 | 21558881 | 22477701 | 23139201 | 24149489 | 26408212 | 27915196 | 29028099 | 29283832 | 30333542 |
| 20016761 | 21558973 | 22477722 | 23139464 | 24149623 | 26408299 | 27915256 | 29028656 | 29283833 | 30333786 |
| 20017201 | 21561012 | 22477905 | 23139561 | 24149635 | 26408616 | 27915479 | 29028766 | 29283837 | 30335059 |
| 20018052 | 21561079 | 22478060 | 23139712 | 24150374 | 26410379 | 27915493 | 29029650 | 29283850 | 30335299 |
| 20018249 | 21562128 | 22478269 | 23139789 | 24150466 | 26411542 | 27915665 | 29029909 | 29283876 | 30335844 |
| 20018273 | 21562194 | 22478795 | 23139880 | 24151382 | 26412606 | 27916181 | 29029957 | 29283887 | 30338126 |
| 20018402 | 21564512 | 22478938 | 23140180 | 24151448 | 26415768 | 27916196 | 29030080 | 29283891 | 30338602 |
| 20018571 | 21565448 | 22479276 | 23140344 | 24151885 | 26417087 | 27916255 | 29030563 | 29283894 | 30338881 |
| 20018867 | 21565833 | 22479311 | 23140376 | 24152202 | 26418164 | 27916520 | 29031560 | 29283897 | 30340173 |
| 20018879 | 21566105 | 22479560 | 23140404 | 24152371 | 26418280 | 27916880 | 29031751 | 29283923 | 30340799 |
| 20018942 | 21566225 | 22479871 | 23141351 | 24152619 | 26419458 | 27917044 | 29031845 | 29283924 | 30341278 |
| 20019288 | 21566230 | 22480073 | 23141511 | 24152923 | 26419728 | 27917502 | 29032161 | 29283926 | 30341640 |
| 20020067 | 21566659 | 22480277 | 23141635 | 24153131 | 26420088 | 27917751 | 29032506 | 29283935 | 30341693 |
| 20020855 | 21566877 | 22480353 | 23141701 | 24153250 | 26420586 | 27917814 | 29032862 | 29283943 | 30342313 |
| 20021781 | 21567016 | 22480520 | 23141775 | 24153314 | 26424014 | 27918296 | 29032925 | 29283944 | 30342547 |
| 20022142 | 21568046 | 22480523 | 23141955 | 24153422 | 26424392 | 27918989 | 29033374 | 29283945 | 30343204 |
| 20022221 | 21569478 | 22480792 | 23142068 | 24153501 | 26424427 | 27919247 | 29033398 | 29283948 | 30343790 |
| 20022532 | 21569599 | 22480843 | 23142450 | 24154332 | 26424908 | 27919753 | 29033491 | 29283960 | 30344747 |
| 20022795 | 21570861 | 22480845 | 23143152 | 24154676 | 26425715 | 27919892 | 29033548 | 29283963 | 30345342 |
| 20023002 | 21571057 | 22480902 | 23143215 | 24154979 | 26426072 | 27920765 | 29033763 | 29283969 | 30345473 |
| 20023006 | 21571843 | 22481040 | 23143350 | 24155102 | 26427086 | 27920799 | 29034021 | 29283971 | 30345721 |
| 20024102 | 21572910 | 22481188 | 23143389 | 24155115 | 26428539 | 27921384 | 29034841 | 29283983 | 30345928 |
| 20024376 | 21572995 | 22481220 | 23143661 | 24155769 | 26428552 | 27921490 | 29035328 | 29283995 | 30345991 |
| 20024537 | 21573865 | 22481297 | 23143852 | 24156443 | 26428921 | 27922106 | 29035553 | 29283997 | 30346170 |
| 20024694 | 21573916 | 22481328 | 23143874 | 24156567 | 26430246 | 27922202 | 29035968 | 29284002 | 30346320 |
| 20025821 | 21575439 | 22481731 | 23144382 | 24157375 | 26430328 | 27922548 | 29036300 | 29284009 | 30346411 |
| 20025893 | 21575993 | 22482683 | 23144988 | 24157673 | 26430847 | 27922798 | 29036330 | 29284014 | 30346691 |
| 20025923 | 21576226 | 22483465 | 23145515 | 24158144 | 26431681 | 27922873 | 29036432 | 29284035 | 30347265 |
| 20026315 | 21576379 | 22483886 | 23146312 | 24158684 | 26431692 | 27923538 | 29036793 | 29284040 | 30347368 |
| 20026387 | 21576788 | 22484282 | 23146801 | 24159172 | 26434725 | 27924295 | 29037168 | 29284062 | 30347889 |
| 20026697 | 21576893 | 22484399 | 23146810 | 24160021 | 26435600 | 27924358 | 29037239 | 29284069 | 30348185 |
| 20027071 | 21577707 | 22484647 | 23147170 | 24160075 | 26436537 | 27924913 | 29037415 | 29284071 | 30348191 |
| 20027480 | 21577985 | 22484668 | 23147231 | 24160402 | 26436575 | 27926843 | 29040058 | 29284087 | 30348274 |
| 20028041 | 21577991 | 22485136 | 23147719 | 24160606 | 26440347 | 27927753 | 29040339 | 29284101 | 30349008 |
| 20028164 | 21578918 | 22485220 | 23148002 | 24160869 | 26441126 | 27928000 | 29040659 | 29284104 | 30349066 |
| 20028188 | 21579004 | 22485416 | 23148429 | 24161015 | 26441167 | 27928159 | 29043277 | 29284115 | 30349111 |
| 20028222 | 21579015 | 22485483 | 23148463 | 24161269 | 26441344 | 27928516 | 29043490 | 29284132 | 30349299 |
| 20028393 | 21579580 | 22485827 | 23148529 | 24161711 | 26441391 | 27928546 | 29043498 | 29284134 | 30350461 |
| 20028539 | 21579742 | 22485898 | 23148573 | 24161996 | 26441706 | 27928549 | 29043515 | 29284136 | 30350597 |
| 20029183 | 21579895 | 22486474 | 23148597 | 24162009 | 26442172 | 27928668 | 29043654 | 29284138 | 30350964 |
| 20029405 | 21579990 | 22486530 | 23148931 | 24162286 | 26443472 | 27931544 | 29043872 | 29284139 | 30351485 |
| 20029725 | 21581585 | 22486586 | 23149336 | 24162445 | 26443613 | 27931776 | 29044093 | 29284156 | 30351954 |
| 20029951 | 21581883 | 22487332 | 23149418 | 24164158 | 26444617 | 27931830 | 29044148 | 29284160 | 30352116 |
| 20030334 | 21582514 | 22487343 | 23149622 | 24164541 | 26446023 | 27932126 | 29044178 | 29284164 | 30353021 |
| 20030840 | 21582745 | 22487590 | 23149639 | 24164761 | 26446206 | 27934237 | 29044217 | 29284165 | 30353152 |
| 20030862 | 21582808 | 22487790 | 23149674 | 24164951 | 26446294 | 27934281 | 29044737 | 29284168 | 30353305 |
| 20031267 | 21583194 | 22488084 | 23149916 | 24165470 | 26448339 | 27934643 | 29044747 | 29284170 | 30353396 |
| 20032082 | 21583359 | 22488088 | 23149980 | 24166076 | 26450294 | 27934726 | 29044995 | 29284172 | 30353727 |
| 20032122 | 21583498 | 22488115 | 23150371 | 24166480 | 26452443 | 27935057 | 29045015 | 29284179 | 30354590 |
| 20032353 | 21583858 | 22488903 | 23150546 | 24166522 | 26453937 | 27935354 | 29045029 | 29284191 | 30355344 |
| 20032590 | 21583901 | 22489124 | 23150639 | 24167518 | 26454689 | 27936070 | 29045425 | 29284193 | 30355572 |
| 20032799 | 21584257 | 22489196 | 23150893 | 24168227 | 26456349 | 27936220 | 29045634 | 29284210 | 30355661 |
| 20033328 | 21585187 | 22489406 | 23150910 | 24168262 | 26456356 | 27936412 | 29046440 | 29284214 | 30355963 |
| 20033515 | 21585246 | 22489489 | 23151160 | 24168997 | 26456468 | 27936767 | 29046915 | 29284216 | 30356058 |
| 20033573 | 21585446 | 22489668 | 23151670 | 24169419 | 26456656 | 27936965 | 29047244 | 29284223 | 30356231 |
| 20033878 | 21586261 | 22489728 | 23151849 | 24169872 | 26457473 | 27937264 | 29047764 | 29284239 | 30356310 |
| 20034010 | 21587578 | 22489923 | 23152481 | 24169875 | 26457624 | 27937484 | 29048260 | 29284242 | 30357419 |
| 20034147 | 21587851 | 22490560 | 23152486 | 24170802 | 26458262 | 27937575 | 29048356 | 29284245 | 30357629 |
| 20034148 | 21588685 | 22491126 | 23152998 | 24171016 | 26458949 | 27937815 | 29048552 | 29284246 | 30357687 |
| 20034660 | 21589031 | 22491143 | 23153129 | 24171057 | 26459060 | 27938564 | 29049284 | 29284250 | 30358863 |
| 20034754 | 21589305 | 22491160 | 23153338 | 24171745 | 26459800 | 27939889 | 29049558 | 29284258 | 30358877 |
| 20035151 | 21590547 | 22491204 | 23153558 | 24172055 | 26460870 | 27940000 | 29049596 | 29284262 | 30360309 |
| 20035239 | 21592951 | 22491405 | 23153897 | 24173214 | 26460892 | 27940524 | 29049760 | 29284264 | 30360666 |
| 20035925 | 21593557 | 22491420 | 23154059 | 24173281 | 26462062 | 27941109 | 29050346 | 29284268 | 30361297 |
| 20038048 | 21595073 | 22491541 | 23154151 | 24173364 | 26462702 | 27941318 | 29050439 | 29284278 | 30362125 |
| 20038627 | 21595235 | 22491572 | 23154399 | 24173414 | 26465714 | 27941396 | 29050621 | 29284279 | 30362565 |
| 20038667 | 21595671 | 22491659 | 23154408 | 24174049 | 26466504 | 27941542 | 29051081 | 29284280 | 30362761 |
| 20038978 | 21596616 | 22492017 | 23154456 | 24174728 | 26466835 | 27941596 | 29051201 | 29284287 | 30363692 |
| 20039611 | 21596907 | 22492304 | 23155625 | 24175351 | 26466969 | 27941748 | 29051900 | 29284294 | 30364231 |
| 20040127 | 21597087 | 22492976 | 23155790 | 24176077 | 26468336 | 27941759 | 29052020 | 29284299 | 30364933 |
| 20040342 | 21597483 | 22493139 | 23155902 | 24176302 | 26468709 | 27942897 | 29052266 | 29284308 | 30365166 |
| 20040399 | 21597734 | 22493165 | 23156569 | 24176552 | 26468796 | 27943537 | 29052749 | 29284322 | 30365794 |
| 20040856 | 21598101 | 22493394 | 23156748 | 24176632 | 26469676 | 27944369 | 29053701 | 29284335 | 30366053 |
| 20041034 | 21598798 | 22493520 | 23156853 | 24176685 | 26470359 | 27945183 | 29053942 | 29284341 | 30366155 |
| 20041372 | 21598909 | 22493585 | 23156926 | 24177135 | 26471041 | 27945650 | 29054004 | 29284346 | 30366599 |
| 20041448 | 21599503 | 22494642 | 23157092 | 24177559 | 26471413 | 27945866 | 29054222 | 29284351 | 30367131 |
| 20041528 | 21599515 | 22494736 | 23157269 | 24177744 | 26474426 | 27946179 | 29054352 | 29284360 | 30367793 |
| 20041553 | 21600860 | 22494838 | 23157274 | 24178460 | 26475483 | 27946822 | 29054487 | 29284365 | 30367824 |
| 20042182 | 21602563 | 22494851 | 23157496 | 24179402 | 26476787 | 27947801 | 29054706 | 29284373 | 30368233 |
| 20042221 | 21602656 | 22494997 | 23157646 | 24179491 | 26477985 | 27947856 | 29054888 | 29284376 | 30368759 |
| 20042323 | 21604004 | 22495617 | 23157842 | 24179807 | 26478465 | 27948286 | 29055736 | 29284380 | 30369206 |
| 20042375 | 21605120 | 22495618 | 23158095 | 24179869 | 26478857 | 27948372 | 29056709 | 29284381 | 30369811 |
| 20042571 | 21606319 | 22495759 | 23158272 | 24180203 | 26479360 | 27948747 | 29056972 | 29284388 | 30369822 |
| 20042795 | 21606403 | 22495785 | 23158727 | 24180652 | 26481974 | 27948900 | 29057019 | 29284391 | 30370486 |
| 20042815 | 21608215 | 22496901 | 23159622 | 24181364 | 26483468 | 27948947 | 29057030 | 29284399 | 30370498 |
| 20043171 | 21608909 | 22497582 | 23159658 | 24181861 | 26484758 | 27949433 | 29057848 | 29284403 | 30371293 |
| 20043295 | 21609033 | 22497758 | 23159757 | 24182093 | 26484911 | 27949865 | 29057881 | 29284417 | 30372678 |
| 20043313 | 21609575 | 22497807 | 23160052 | 24182194 | 26485556 | 27950196 | 29058474 | 29284431 | 30372944 |
| 20043455 | 21609901 | 22498134 | 23160212 | 24182751 | 26485600 | 27951104 | 29058737 | 29284432 | 30373005 |
| 20044512 | 21611047 | 22498193 | 23160492 | 24183134 | 26486887 | 27952102 | 29059688 | 29284434 | 30373549 |
| 20044887 | 21611339 | 22498211 | 23160626 | 24184140 | 26486912 | 27952125 | 29059734 | 29284439 | 30373760 |
| 20045811 | 21613762 | 22498563 | 23160702 | 24184534 | 26487122 | 27952153 | 29059847 | 29284448 | 30373908 |
| 20046075 | 21614310 | 22498637 | 23160988 | 24185064 | 26487943 | 27952192 | 29060350 | 29284450 | 30374802 |
| 20046097 | 21614907 | 22498949 | 23161375 | 24185114 | 26488534 | 27952610 | 29060603 | 29284483 | 30375454 |
| 20046162 | 21615356 | 22498977 | 23161387 | 24185588 | 26488655 | 27952646 | 29061075 | 29284495 | 30375607 |
| 20046374 | 21616175 | 22499283 | 23162178 | 24185945 | 26488780 | 27953297 | 29061188 | 29284513 | 30375844 |
| 20046608 | 21618486 | 22499407 | 23162201 | 24186356 | 26495612 | 27953519 | 29061846 | 29284532 | 30376305 |
| 20047380 | 21618575 | 22499632 | 23162501 | 24187100 | 26497404 | 27954403 | 29063641 | 29284533 | 30376718 |
| 20047518 | 21619215 | 22499866 | 23162606 | 24187329 | 26498519 | 27954433 | 29064355 | 29284538 | 30377963 |
| 20047578 | 21619451 | 22499874 | 23162707 | 24187542 | 26500093 | 27954790 | 29064624 | 29284571 | 30378053 |
| 20048115 | 21619551 | 22500232 | 23162824 | 24187774 | 26501430 | 27955229 | 29064852 | 29284572 | 30379209 |
| 20048307 | 21619764 | 22500293 | 23162842 | 24188226 | 26501737 | 27955462 | 29065558 | 29284574 | 30379271 |
| 20048725 | 21620343 | 22500342 | 23162888 | 24188527 | 26501770 | 27956126 | 29066098 | 29284576 | 30379617 |
| 20048810 | 21620624 | 22500418 | 23162990 | 24188534 | 26502167 | 27956736 | 29066255 | 29284592 | 30380437 |
| 20048919 | 21620854 | 22500667 | 23163011 | 24189208 | 26502705 | 27957568 | 29067689 | 29284596 | 30380480 |
| 20049584 | 21622801 | 22501169 | 23163438 | 24189397 | 26503293 | 27957584 | 29067858 | 29284598 | 30380643 |
| 20049806 | 21624231 | 22501640 | 23163464 | 24189541 | 26503604 | 27957718 | 29068206 | 29284608 | 30380857 |
| 20050013 | 21624915 | 22502176 | 23163994 | 24190207 | 26503956 | 27957984 | 29068414 | 29284610 | 30380861 |
| 20050517 | 21626423 | 22502192 | 23164298 | 24190522 | 26504656 | 27959034 | 29068796 | 29284627 | 30381342 |
| 20051055 | 21626433 | 22502374 | 23164934 | 24191177 | 26505063 | 27959665 | 29069042 | 29284631 | 30381617 |
| 20051788 | 21626768 | 22502952 | 23165462 | 24191309 | 26505499 | 27960539 | 29069260 | 29284636 | 30381690 |
| 20052135 | 21626869 | 22503042 | 23165673 | 24191395 | 26506477 | 27960717 | 29069567 | 29284648 | 30381964 |
| 20052540 | 21628894 | 22503140 | 23165756 | 24191489 | 26507566 | 27960791 | 29069725 | 29284652 | 30382617 |
| 20052811 | 21629223 | 22503314 | 23166037 | 24192247 | 26507870 | 27961538 | 29070561 | 29284655 | 30382788 |
| 20052937 | 21629800 | 22503397 | 23166341 | 24193020 | 26508440 | 27962000 | 29070564 | 29284664 | 30382822 |
| 20053026 | 21630313 | 22503737 | 23166459 | 24193105 | 26510958 | 27962701 | 29071900 | 29284675 | 30382858 |
| 20053102 | 21630366 | 22503786 | 23166925 | 24193285 | 26510979 | 27962904 | 29072020 | 29284676 | 30383002 |
| 20053133 | 21630659 | 22504826 | 23166953 | 24193637 | 26511073 | 27963059 | 29072194 | 29284678 | 30383094 |
| 20053154 | 21632031 | 22504949 | 23166995 | 24193885 | 26511972 | 27963131 | 29072619 | 29284685 | 30383393 |
| 20053411 | 21632217 | 22505206 | 23167248 | 24194072 | 26512140 | 27963720 | 29072930 | 29284711 | 30383550 |
| 20054111 | 21632224 | 22505865 | 23167357 | 24194163 | 26512623 | 27963891 | 29072939 | 29284716 | 30384150 |
| 20054294 | 21632905 | 22505900 | 23167409 | 24194429 | 26515541 | 27964353 | 29073289 | 29284728 | 30385094 |
| 20054642 | 21632978 | 22505953 | 23167477 | 24194658 | 26517894 | 27964444 | 29073779 | 29284729 | 30385302 |
| 20054762 | 21634496 | 22505971 | 23167773 | 24195533 | 26518001 | 27964707 | 29074387 | 29284730 | 30385931 |
| 20055010 | 21634924 | 22505980 | 23167793 | 24195920 | 26520324 | 27965206 | 29074492 | 29284736 | 30386028 |
| 20055099 | 21634998 | 22506835 | 23167909 | 24196141 | 26520419 | 27965826 | 29075601 | 29284745 | 30386645 |
| 20055804 | 21635306 | 22507084 | 23167996 | 24196298 | 26520425 | 27966168 | 29076495 | 29284746 | 30386722 |
| 20056097 | 21635741 | 22507400 | 23168070 | 24196492 | 26520659 | 27967904 | 29076843 | 29284757 | 30386831 |
| 20056252 | 21636463 | 22508060 | 23168200 | 24196607 | 26521649 | 27967990 | 29076943 | 29284759 | 30387458 |
| 20056910 | 21637191 | 22508066 | 23168316 | 24196832 | 26523210 | 27968423 | 29076952 | 29284786 | 30387612 |
| 20057874 | 21638208 | 22508130 | 23168368 | 24197505 | 26523482 | 27969506 | 29076986 | 29284789 | 30387618 |
| 20059264 | 21638673 | 22508184 | 23168547 | 24197835 | 26524429 | 27970669 | 29077141 | 29284795 | 30387695 |
| 20059502 | 21639886 | 22508254 | 23168680 | 24197873 | 26524602 | 27970731 | 29078138 | 29284823 | 30388159 |
| 20059735 | 21640810 | 22508286 | 23168844 | 24199241 | 26527654 | 27970918 | 29078684 | 29284842 | 30388275 |
| 20060044 | 21641495 | 22508345 | 23169167 | 24199375 | 26528043 | 27971209 | 29078734 | 29284845 | 30388400 |
| 20060081 | 21642265 | 22508784 | 23169506 | 24199798 | 26531920 | 27971446 | 29078760 | 29284852 | 30389505 |
| 20060548 | 21642327 | 22509457 | 23169519 | 24199841 | 26532155 | 27971658 | 29079036 | 29284855 | 30390153 |
| 20060851 | 21642665 | 22509547 | 23169633 | 24200892 | 26534293 | 27972483 | 29080072 | 29284858 | 30390260 |
| 20061249 | 21643213 | 22509583 | 23169901 | 24201118 | 26535996 | 27973128 | 29080124 | 29284861 | 30390499 |
| 20061332 | 21643353 | 22509956 | 23170069 | 24201658 | 26536986 | 27973584 | 29080625 | 29284867 | 30391758 |
| 20061406 | 21645032 | 22509984 | 23170412 | 24201942 | 26536992 | 27973757 | 29081470 | 29284872 | 30391860 |
| 20061716 | 21645410 | 22510289 | 23170527 | 24202027 | 26537339 | 27973806 | 29081787 | 29284893 | 30392832 |
| 20062644 | 21647069 | 22510294 | 23170861 | 24202142 | 26538964 | 27974728 | 29081890 | 29284914 | 30392912 |
| 20062807 | 21647079 | 22511097 | 23171192 | 24202164 | 26538982 | 27975041 | 29082139 | 29284925 | 30393020 |
| 20063765 | 21647781 | 22511378 | 23171327 | 24202217 | 26539011 | 27975135 | 29082177 | 29284929 | 30393809 |
| 20063959 | 21648024 | 22511414 | 23171388 | 24202743 | 26539283 | 27975696 | 29082535 | 29284930 | 30394024 |
| 20064454 | 21648327 | 22511454 | 23171445 | 24203097 | 26539480 | 27976131 | 29082832 | 29284931 | 30394347 |
| 20064744 | 21648791 | 22511547 | 23171633 | 24203864 | 26540222 | 27977134 | 29083445 | 29284932 | 30394434 |
| 20065024 | 21649375 | 22511882 | 23171727 | 24204533 | 26541668 | 27977406 | 29084143 | 29284933 | 30394565 |
| 20065597 | 21649424 | 22512090 | 23171940 | 24204616 | 26543446 | 27977821 | 29084465 | 29284938 | 30394647 |
| 20065802 | 21649561 | 22512422 | 23172623 | 24204854 | 26545232 | 27978028 | 29084866 | 29284957 | 30395242 |
| 20065819 | 21649670 | 22512448 | 23172879 | 24204944 | 26545516 | 27978331 | 29085082 | 29284958 | 30395704 |
| 20066461 | 21649782 | 22512486 | 23172931 | 24205016 | 26546524 | 27978356 | 29085456 | 29284964 | 30395784 |
| 20066539 | 21650044 | 22513166 | 23173250 | 24205022 | 26549706 | 27978626 | 29085514 | 29284965 | 30396442 |
| 20067374 | 21650070 | 22513504 | 23173270 | 24205153 | 26549823 | 27979335 | 29086056 | 29284969 | 30397574 |
| 20067513 | 21650183 | 22513729 | 23173760 | 24205160 | 26549963 | 27979505 | 29086332 | 29284970 | 30397679 |
| 20067582 | 21650713 | 22513792 | 23173868 | 24205220 | 26550384 | 27979607 | 29086413 | 29284990 | 30397906 |
| 20067976 | 21650986 | 22513988 | 23174383 | 24205420 | 26550752 | 27980408 | 29086556 | 29285006 | 30397982 |
| 20068331 | 21652207 | 22514230 | 23174448 | 24205782 | 26551061 | 27980536 | 29087041 | 29285053 | 30398284 |
| 20068681 | 21652491 | 22514540 | 23174792 | 24206136 | 26551133 | 27980744 | 29087182 | 29285054 | 30398387 |
| 20068705 | 21652519 | 22515311 | 23174979 | 24206323 | 26551443 | 27981179 | 29087441 | 29285056 | 30398684 |
| 20069208 | 21653072 | 22515547 | 23175051 | 24207028 | 26551834 | 27981707 | 29087830 | 29285075 | 30398764 |
| 20069519 | 21653107 | 22515837 | 23175427 | 24207103 | 26554393 | 27981766 | 29088320 | 29285087 | 30398783 |
| 20069627 | 21654485 | 22515903 | 23175525 | 24207747 | 26555759 | 27982771 | 29088378 | 29285103 | 30398862 |
| 20070643 | 21654533 | 22516304 | 23175829 | 24207835 | 26556497 | 27983169 | 29088658 | 29285104 | 30398918 |
| 20071185 | 21655007 | 22516520 | 23176022 | 24207892 | 26557415 | 27983279 | 29088835 | 29285115 | 30399444 |
| 20071298 | 21656008 | 22516735 | 23176452 | 24208130 | 26558397 | 27984000 | 29090148 | 29285121 | 30399709 |
| 20071490 | 21656174 | 22516953 | 23176519 | 24208200 | 26558529 | 27984026 | 29090425 | 29285136 | 30399956 |
| 20071776 | 21657079 | 22517368 | 23176795 | 24208964 | 26558534 | 27984282 | 29090521 | 29285147 | 30400391 |
| 20072600 | 21658429 | 22517485 | 23176894 | 24209416 | 26559097 | 27985044 | 29091333 | 29285153 | 30401355 |
| 20072763 | 21658725 | 22517515 | 23176970 | 24210611 | 26559198 | 27985787 | 29091965 | 29285154 | 30402160 |
| 20072884 | 21658804 | 22518319 | 23176988 | 24210774 | 26559209 | 27986116 | 29092353 | 29285175 | 30402468 |
| 20073501 | 21659393 | 22518591 | 23177297 | 24211024 | 26560366 | 27986216 | 29092385 | 29285178 | 30402700 |
| 20074642 | 21659440 | 22518828 | 23177417 | 24212205 | 26561019 | 27986393 | 29092785 | 29285182 | 30403140 |
| 20074675 | 21660794 | 22518912 | 23177447 | 24212901 | 26561581 | 27986478 | 29092883 | 29285183 | 30403265 |
| 20074826 | 21660956 | 22518973 | 23177501 | 24213149 | 26561663 | 27987278 | 29092887 | 29285185 | 30403387 |
| 20074961 | 21660970 | 22519125 | 23177509 | 24213539 | 26561980 | 27989116 | 29092929 | 29285194 | 30404057 |
| 20075567 | 21661181 | 22519494 | 23177514 | 24213826 | 26564231 | 27989483 | 29093357 | 29285198 | 30404157 |
| 20076160 | 21661578 | 22519512 | 23177677 | 24214195 | 26565320 | 27989785 | 29093613 | 29285203 | 30404423 |
| 20076638 | 21661579 | 22519527 | 23178081 | 24214337 | 26570129 | 27990258 | 29094824 | 29285215 | 30406150 |
| 20077503 | 21662150 | 22519635 | 23178772 | 24214971 | 26570945 | 27991364 | 29095492 | 29285223 | 30406368 |
| 20077663 | 21662893 | 22519757 | 23178779 | 24215406 | 26571630 | 27991400 | 29095959 | 29285245 | 30406533 |
| 20077994 | 21664244 | 22519782 | 23179317 | 24216157 | 26574224 | 27992045 | 29096034 | 29285246 | 30406682 |
| 20078163 | 21665038 | 22520210 | 23179431 | 24216399 | 26574408 | 27992301 | 29096526 | 29285274 | 30406862 |
| 20078493 | 21665206 | 22520462 | 23179456 | 24216451 | 26574908 | 27993950 | 29096603 | 29285301 | 30407028 |
| 20079044 | 21665891 | 22520537 | 23179593 | 24216557 | 26575141 | 27993974 | 29096865 | 29285303 | 30408459 |
| 20079167 | 21666258 | 22520592 | 23179662 | 24216567 | 26575607 | 27994126 | 29097023 | 29285317 | 30408580 |
| 20079515 | 21666759 | 22520644 | 23179821 | 24216713 | 26575711 | 27994461 | 29097861 | 29285325 | 30408633 |
| 20079687 | 21667701 | 22520934 | 23180484 | 24216863 | 26575911 | 27994526 | 29098061 | 29285337 | 30408782 |
| 20080846 | 21667884 | 22521018 | 23181003 | 24217225 | 26576158 | 27994731 | 29098491 | 29285340 | 30409020 |
| 20080882 | 21668252 | 22521032 | 23181420 | 24218257 | 26576483 | 27994970 | 29098696 | 29285342 | 30409112 |
| 20080901 | 21668368 | 22522288 | 23181477 | 24218394 | 26576631 | 27995330 | 29098893 | 29285348 | 30410228 |
| 20081395 | 21669110 | 22522341 | 23181506 | 24218472 | 26577034 | 27996055 | 29099008 | 29285353 | 30411045 |
| 20081796 | 21669414 | 22522549 | 23181546 | 24218983 | 26577477 | 27996220 | 29099089 | 29285367 | 30411205 |
| 20082061 | 21669495 | 22522873 | 23181724 | 24219270 | 26577886 | 27996390 | 29099714 | 29285370 | 30412723 |
| 20083067 | 21670163 | 22522994 | 23181737 | 24219335 | 26578484 | 27996849 | 29099753 | 29285373 | 30412906 |
| 20083085 | 21671056 | 22523014 | 23181845 | 24220425 | 26580025 | 27997262 | 29100455 | 29285383 | 30413599 |
| 20083275 | 21671123 | 22523024 | 23182117 | 24220825 | 26581399 | 27997703 | 29100976 | 29285400 | 30413733 |
| 20085041 | 21671539 | 22523036 | 23182247 | 24221072 | 26582420 | 27998329 | 29101235 | 29285403 | 30414043 |
| 20085365 | 21672039 | 22523073 | 23182606 | 24221732 | 26582475 | 27999175 | 29101860 | 29285429 | 30414063 |
| 20086441 | 21672170 | 22523120 | 23182742 | 24222110 | 26582933 | 27999920 | 29102156 | 29285439 | 30414382 |
| 20087137 | 21672740 | 22523149 | 23182774 | 24222387 | 26584208 | 28000165 | 29102441 | 29285443 | 30414608 |
| 20088363 | 21673185 | 22523205 | 23182786 | 24222396 | 26585384 | 28000772 | 29102687 | 29285453 | 30414929 |
| 20088993 | 21673397 | 22523261 | 23182970 | 24223087 | 26589838 | 28001086 | 29103225 | 29285455 | 30415436 |
| 20089057 | 21673979 | 22523449 | 23183076 | 24223708 | 26591533 | 28001293 | 29103280 | 29285487 | 30415963 |
| 20089236 | 21674353 | 22523509 | 23183207 | 24223859 | 26591633 | 28001639 | 29104710 | 29285490 | 30416385 |
| 20090024 | 21674661 | 22523918 | 23183224 | 24224054 | 26591884 | 28001760 | 29106591 | 29285498 | 30416705 |
| 20090080 | 21675006 | 22524838 | 23183716 | 24224063 | 26591969 | 28001807 | 29106800 | 29285504 | 30416947 |
| 20090876 | 21675184 | 22525131 | 23183802 | 24224095 | 26592254 | 28002013 | 29106878 | 29285517 | 30417430 |
| 20090916 | 21676176 | 22525509 | 23183987 | 24224399 | 26592733 | 28002033 | 29107233 | 29285522 | 30420033 |
| 20091077 | 21676986 | 22525575 | 23184200 | 24225076 | 26593259 | 28002347 | 29107433 | 29285535 | 30420113 |
| 20091214 | 21678674 | 22525624 | 23184739 | 24225418 | 26593619 | 28002638 | 29107524 | 29285536 | 30420224 |
| 20091246 | 21678713 | 22525688 | 23184821 | 24226032 | 26594165 | 28004259 | 29107579 | 29285537 | 30421898 |
| 20091334 | 21678829 | 22526082 | 23185074 | 24226289 | 26594176 | 28004377 | 29108136 | 29285538 | 30421989 |
| 20091449 | 21679928 | 22526206 | 23185161 | 24226469 | 26594686 | 28004555 | 29108232 | 29285565 | 30422382 |
| 20091678 | 21679989 | 22527057 | 23185371 | 24226494 | 26595134 | 28004814 | 29108738 | 29285577 | 30422983 |
| 20091680 | 21679990 | 22528906 | 23185558 | 24226615 | 26596260 | 28005554 | 29110136 | 29285581 | 30423565 |
| 20091697 | 21680538 | 22529220 | 23185574 | 24226653 | 26596853 | 28005846 | 29111160 | 29285587 | 30424539 |
| 20092038 | 21681372 | 22529240 | 23185714 | 24227057 | 26597700 | 28006166 | 29111752 | 29285595 | 30424933 |
| 20092144 | 21682056 | 22529259 | 23185728 | 24227131 | 26598637 | 28006268 | 29111791 | 29285602 | 30425230 |
| 20092213 | 21682829 | 22529281 | 23185897 | 24227218 | 26599960 | 28007255 | 29111842 | 29285611 | 30425234 |
| 20092316 | 21683139 | 22529354 | 23186782 | 24227567 | 26600188 | 28007606 | 29111846 | 29285614 | 30425931 |
| 20092479 | 21683630 | 22529501 | 23186966 | 24227924 | 26600610 | 28007674 | 29112134 | 29285620 | 30426029 |
| 20092798 | 21683799 | 22529676 | 23187335 | 24228621 | 26600654 | 28008531 | 29112139 | 29285625 | 30426051 |
| 20092903 | 21684038 | 22529817 | 23187694 | 24229737 | 26600914 | 28008882 | 29113975 | 29285638 | 30426498 |
| 20093533 | 21685191 | 22530019 | 23187848 | 24230244 | 26601023 | 28009469 | 29114571 | 29285643 | 30426783 |
| 20093594 | 21685952 | 22530397 | 23188297 | 24230255 | 26601653 | 28009980 | 29114922 | 29285654 | 30426806 |
| 20094153 | 21687159 | 22530725 | 23188916 | 24230996 | 26601924 | 28010479 | 29116388 | 29285671 | 30426868 |
| 20094605 | 21687517 | 22530881 | 23189013 | 24232226 | 26602798 | 28010781 | 29116821 | 29285677 | 30426964 |
| 20094845 | 21687807 | 22530952 | 23189280 | 24232829 | 26604404 | 28011777 | 29116872 | 29285689 | 30427312 |
| 20094985 | 21688065 | 22531419 | 23189294 | 24233048 | 26606653 | 28011897 | 29117763 | 29285691 | 30429717 |
| 20095658 | 21688706 | 22531748 | 23189823 | 24233094 | 26607054 | 28011968 | 29117941 | 29285702 | 30429757 |
| 20095911 | 21689319 | 22532196 | 23189909 | 24233244 | 26607273 | 28012592 | 29118228 | 29285703 | 30429997 |
| 20096052 | 21689461 | 22532705 | 23189968 | 24233359 | 26607831 | 28012644 | 29118697 | 29285704 | 30430571 |
| 20096099 | 21689558 | 22532736 | 23190482 | 24234503 | 26609161 | 28013177 | 29118758 | 29285712 | 30431740 |
| 20096382 | 21689565 | 22532817 | 23190920 | 24234570 | 26610500 | 28013274 | 29118842 | 29285719 | 30431841 |
| 20096693 | 21689985 | 22533254 | 23190968 | 24234926 | 26611352 | 28013819 | 29118977 | 29285723 | 30432557 |
| 20096768 | 21690141 | 22533304 | 23191149 | 24235335 | 26612550 | 28014322 | 29119144 | 29285725 | 30433550 |
| 20097254 | 21690408 | 22533424 | 23191441 | 24236270 | 26612898 | 28014876 | 29119485 | 29285735 | 30434316 |
| 20097537 | 21690448 | 22533462 | 23191526 | 24236810 | 26612935 | 28015116 | 29119829 | 29285737 | 30435201 |
| 20097636 | 21690741 | 22533659 | 23191626 | 24238156 | 26613930 | 28015187 | 29119972 | 29285739 | 30435371 |
| 20097688 | 21691217 | 22533824 | 23191888 | 24238435 | 26614627 | 28015926 | 29120105 | 29285742 | 30436745 |
| 20098713 | 21692322 | 22533873 | 23191902 | 24238775 | 26614814 | 28015945 | 29120239 | 29285755 | 30437477 |
| 20098788 | 21693253 | 22534206 | 23191928 | 24239395 | 26614960 | 28016303 | 29120664 | 29285766 | 30437733 |
| 20098913 | 21694141 | 22534283 | 23192684 | 24239587 | 26617075 | 28016955 | 29121571 | 29285773 | 30438013 |
| 20099054 | 21694302 | 22534349 | 23192708 | 24239600 | 26617994 | 28017309 | 29121947 | 29285777 | 30438589 |
| 20099161 | 21695823 | 22534463 | 23192878 | 24239716 | 26618411 | 28017604 | 29122475 | 29285783 | 30439052 |
| 20099276 | 21697258 | 22534623 | 23193016 | 24239912 | 26618530 | 28017659 | 29123469 | 29285790 | 30439057 |
| 20099446 | 21698759 | 22534769 | 23193356 | 24240012 | 26619215 | 28017963 | 29123480 | 29285798 | 30439073 |
| 20100318 | 21700121 | 22534812 | 23193507 | 24240125 | 26619506 | 28018646 | 29123633 | 29285802 | 30439201 |
| 20100410 | 21700206 | 22535115 | 23193519 | 24240667 | 26619606 | 28018669 | 29123698 | 29285814 | 30439514 |
| 20100555 | 21700264 | 22535285 | 23193553 | 24241178 | 26622258 | 28019303 | 29124267 | 29285815 | 30440635 |
| 20100805 | 21700366 | 22535572 | 23193810 | 24241598 | 26622614 | 28019400 | 29124284 | 29285822 | 30441023 |
| 20101065 | 21700764 | 22535676 | 23194311 | 24242667 | 26622912 | 28019635 | 29124836 | 29285833 | 30444063 |
| 20101712 | 21701054 | 22536166 | 23194587 | 24243345 | 26624480 | 28019765 | 29124912 | 29285841 | 30444285 |
| 20102821 | 21705995 | 22536395 | 23194994 | 24243432 | 26627249 | 28020178 | 29125326 | 29285849 | 30444646 |
| 20102884 | 21707314 | 22536692 | 23195215 | 24243693 | 26628318 | 28020285 | 29126446 | 29285858 | 30445099 |
| 20103366 | 21707466 | 22536814 | 23195508 | 24243913 | 26629821 | 28020691 | 29126934 | 29285864 | 30445678 |
| 20104180 | 21707804 | 22537186 | 23195644 | 24244153 | 26630176 | 28020756 | 29127284 | 29285874 | 30445776 |
| 20104382 | 21707903 | 22537213 | 23195675 | 24244443 | 26630200 | 28021322 | 29127490 | 29285889 | 30446917 |
| 20105765 | 21708092 | 22537406 | 23196313 | 24244777 | 26630605 | 28021533 | 29127660 | 29285902 | 30447194 |
| 20106129 | 21708571 | 22537422 | 23196469 | 24244828 | 26632314 | 28021702 | 29127883 | 29285909 | 30447260 |
| 20107090 | 21709500 | 22537535 | 23196661 | 24244857 | 26633291 | 28022503 | 29128831 | 29285914 | 30447268 |
| 20108044 | 21710541 | 22537580 | 23196991 | 24244966 | 26633442 | 28022724 | 29128996 | 29285916 | 30447328 |
| 20108705 | 21710790 | 22537913 | 23197128 | 24245272 | 26633753 | 28023180 | 29129070 | 29285924 | 30447403 |
| 20108711 | 21711542 | 22538089 | 23197278 | 24245768 | 26634132 | 28023305 | 29129818 | 29285944 | 30447501 |
| 20109377 | 21711768 | 22538237 | 23197359 | 24246164 | 26634697 | 28023608 | 29130691 | 29285948 | 30448446 |
| 20109423 | 21711926 | 22538244 | 23197667 | 24246395 | 26634965 | 28024607 | 29130843 | 29285958 | 30448695 |
| 20110854 | 21712641 | 22538294 | 23197863 | 24247376 | 26635475 | 28025044 | 29130923 | 29285963 | 30448719 |
| 20111182 | 21713538 | 22538469 | 23198224 | 24247560 | 26635494 | 28025720 | 29130963 | 29285976 | 30449082 |
| 20112094 | 21713691 | 22538506 | 23198478 | 24247693 | 26636635 | 28025858 | 29131007 | 29285982 | 30449191 |
| 20112354 | 21713721 | 22538563 | 23198494 | 24247919 | 26636912 | 28026001 | 29131268 | 29285998 | 30450363 |
| 20112524 | 21714141 | 22539112 | 23198554 | 24248568 | 26637428 | 28026933 | 29131801 | 29286004 | 30450646 |
| 20112615 | 21715311 | 22539152 | 23198597 | 24248693 | 26637743 | 28027608 | 29132873 | 29286021 | 30451180 |
| 20112668 | 21715887 | 22539229 | 23198604 | 24248861 | 26638633 | 28028278 | 29133502 | 29286024 | 30451479 |
| 20114153 | 21715976 | 22539537 | 23199077 | 24248954 | 26639768 | 28028958 | 29133973 | 29286063 | 30451724 |
| 20114377 | 21716199 | 22539767 | 23199806 | 24249014 | 26639950 | 28029187 | 29134210 | 29286070 | 30452132 |
| 20114535 | 21716223 | 22539852 | 23199840 | 24249163 | 26640577 | 28029336 | 29134712 | 29286074 | 30452161 |
| 20114914 | 21716238 | 22539955 | 23199970 | 24249220 | 26640788 | 28029352 | 29135223 | 29286082 | 30452262 |
| 20114944 | 21716573 | 22540043 | 23199978 | 24249374 | 26640968 | 28029410 | 29135238 | 29286097 | 30452737 |
| 20115083 | 21716656 | 22540094 | 23200261 | 24249471 | 26643215 | 28029578 | 29135660 | 29286103 | 30453249 |
| 20115120 | 21716693 | 22540309 | 23200899 | 24249636 | 26644405 | 28029641 | 29136651 | 29286141 | 30453381 |
| 20115633 | 21716894 | 22540520 | 23200916 | 24249786 | 26646335 | 28029747 | 29137629 | 29286161 | 30455525 |
| 20116202 | 21717545 | 22540884 | 23201435 | 24249953 | 26646360 | 28029793 | 29137681 | 29286166 | 30455890 |
| 20116228 | 21718055 | 22541009 | 23201448 | 24250562 | 26646842 | 28030348 | 29137811 | 29286171 | 30456292 |
| 20116336 | 21719381 | 22541127 | 23201451 | 24250567 | 26647392 | 28030679 | 29138139 | 29286179 | 30458023 |
| 20116340 | 21719533 | 22541210 | 23201675 | 24251171 | 26648202 | 28031375 | 29138274 | 29286184 | 30458886 |
| 20116565 | 21720194 | 22541768 | 23201710 | 24251535 | 26648264 | 28032664 | 29138547 | 29286194 | 30458895 |
| 20116684 | 21720534 | 22541797 | 23201803 | 24252152 | 26648749 | 28032878 | 29139486 | 29286220 | 30459897 |
| 20117134 | 21720975 | 22541888 | 23201910 | 24252632 | 26649613 | 28033049 | 29140765 | 29286221 | 30460396 |
| 20117238 | 21720980 | 22542163 | 23202430 | 24252684 | 26650984 | 28033453 | 29141012 | 29286228 | 30461200 |
| 20117506 | 21721298 | 22542219 | 23202572 | 24252691 | 26651186 | 28033738 | 29141709 | 29286229 | 30461270 |
| 20118120 | 21721329 | 22542319 | 23202626 | 24253943 | 26651398 | 28033942 | 29141729 | 29286233 | 30461696 |
| 20118633 | 21723034 | 22543039 | 23202627 | 24253989 | 26652762 | 28034059 | 29142586 | 29286237 | 30461927 |
| 20118716 | 21723067 | 22543071 | 23202707 | 24254280 | 26653715 | 28034214 | 29143190 | 29286239 | 30462056 |
| 20118780 | 21723356 | 22543196 | 23202954 | 24254484 | 26653771 | 28035597 | 29143248 | 29286241 | 30462559 |
| 20118976 | 21723930 | 22543239 | 23203090 | 24254522 | 26654228 | 28035650 | 29143863 | 29286255 | 30462666 |
| 20119345 | 21723956 | 22543326 | 23203227 | 24254768 | 26654650 | 28035794 | 29144155 | 29286268 | 30463310 |
| 20119790 | 21724070 | 22543353 | 23203397 | 24254824 | 26655885 | 28036133 | 29145159 | 29286308 | 30463531 |
| 20120516 | 21724580 | 22543564 | 23203745 | 24254837 | 26656263 | 28036903 | 29145177 | 29286310 | 30463798 |
| 20120797 | 21725079 | 22543639 | 23203853 | 24254952 | 26656987 | 28037174 | 29146248 | 29286311 | 30464039 |
| 20121295 | 21725524 | 22543851 | 23203906 | 24254959 | 26657016 | 28037308 | 29146344 | 29286316 | 30464143 |
| 20121415 | 21726083 | 22544434 | 23203950 | 24255960 | 26657457 | 28037646 | 29146395 | 29286319 | 30464178 |
| 20121938 | 21727616 | 22545135 | 23204225 | 24256465 | 26657477 | 28037685 | 29146524 | 29286354 | 30464207 |
| 20122272 | 21727774 | 22545154 | 23204300 | 24256787 | 26657988 | 28038232 | 29146770 | 29286358 | 30464410 |
| 20122446 | 21729338 | 22545160 | 23204346 | 24256788 | 26658008 | 28038319 | 29147166 | 29286360 | 30464855 |
| 20123063 | 21730053 | 22545643 | 23204390 | 24257200 | 26658501 | 28038385 | 29147863 | 29286365 | 30465795 |
| 20124405 | 21730112 | 22546000 | 23204559 | 24258139 | 26658591 | 28038954 | 29147976 | 29286370 | 30466093 |
| 20125065 | 21730684 | 22546110 | 23204763 | 24259362 | 26659105 | 28039373 | 29147986 | 29286371 | 30466513 |
| 20125433 | 21730941 | 22546134 | 23204994 | 24259659 | 26659189 | 28039404 | 29148152 | 29286372 | 30466701 |
| 20125953 | 21731046 | 22546140 | 23205412 | 24259768 | 26659315 | 28039858 | 29148287 | 29286380 | 30466823 |
| 20126019 | 21731143 | 22546859 | 23205568 | 24260485 | 26660085 | 28039920 | 29148561 | 29286390 | 30466846 |
| 20126258 | 21731559 | 22546951 | 23205772 | 24260799 | 26661371 | 28040367 | 29149543 | 29286401 | 30467118 |
| 20126834 | 21731763 | 22546988 | 23205843 | 24260872 | 26661643 | 28040397 | 29149820 | 29286407 | 30467301 |
| 20127199 | 21732864 | 22547281 | 23205871 | 24261170 | 26661827 | 28041408 | 29150035 | 29286432 | 30467560 |
| 20127785 | 21733333 | 22547542 | 23206297 | 24261203 | 26661877 | 28041670 | 29150414 | 29286441 | 30467694 |
| 20127786 | 21733468 | 22548145 | 23206706 | 24261325 | 26662809 | 28041864 | 29150629 | 29286452 | 30467706 |
| 20128138 | 21733582 | 22548494 | 23206767 | 24261736 | 26663158 | 28042168 | 29151380 | 29286457 | 30468389 |
| 20128684 | 21734193 | 22548649 | 23208022 | 24262058 | 26663639 | 28042550 | 29151881 | 29286462 | 30468839 |
| 20129256 | 21734959 | 22549725 | 23208567 | 24262300 | 26664692 | 28043058 | 29152272 | 29286482 | 30468928 |
| 20129629 | 21735970 | 22549815 | 23208795 | 24262588 | 26665325 | 28043648 | 29152402 | 29286486 | 30469033 |
| 20129699 | 21737828 | 22550033 | 23208869 | 24263607 | 26666511 | 28043673 | 29152675 | 29286500 | 30469037 |
| 20130259 | 21738101 | 22550257 | 23209043 | 24264312 | 26667127 | 28043721 | 29152986 | 29286513 | 30469079 |
| 20130974 | 21738137 | 22550360 | 23209320 | 24264616 | 26667460 | 28043730 | 29153016 | 29286518 | 30469135 |
| 20131777 | 21738901 | 22550371 | 23209546 | 24265726 | 26668909 | 28043878 | 29153103 | 29286529 | 30469482 |
| 20131867 | 21739016 | 22550596 | 23209843 | 24265841 | 26671597 | 28044063 | 29153223 | 29286536 | 30469558 |
| 20133342 | 21739422 | 22550743 | 23210083 | 24266039 | 26671854 | 28044327 | 29154091 | 29286537 | 30469604 |
| 20133379 | 21739440 | 22550842 | 23210301 | 24266090 | 26673466 | 28044507 | 29154138 | 29286563 | 30470040 |
| 20133634 | 21739562 | 22551061 | 23210465 | 24266365 | 26673785 | 28045578 | 29154181 | 29286565 | 30470222 |
| 20134458 | 21739784 | 22551252 | 23210650 | 24266440 | 26673952 | 28046087 | 29154255 | 29286581 | 30470494 |
| 20134729 | 21739875 | 22551346 | 23211979 | 24266475 | 26675050 | 28046972 | 29155813 | 29286584 | 30470931 |
| 20135794 | 21739957 | 22551446 | 23212186 | 24266482 | 26676221 | 28047840 | 29155997 | 29286588 | 30471658 |
| 20135971 | 21740250 | 22551809 | 23213101 | 24266748 | 26679135 | 28047911 | 29156732 | 29286591 | 30472996 |
| 20136037 | 21741072 | 22552039 | 23213528 | 24267322 | 26679168 | 28048360 | 29157346 | 29286600 | 30473614 |
| 20136166 | 21743675 | 22552552 | 23213546 | 24267665 | 26680427 | 28048466 | 29158080 | 29286610 | 30473703 |
| 20136208 | 21743678 | 22553426 | 23213610 | 24268100 | 26680957 | 28048809 | 29158156 | 29286622 | 30473892 |
| 20136222 | 21744395 | 22553433 | 23213612 | 24268420 | 26683545 | 28049227 | 29158472 | 29286623 | 30474210 |
| 20136731 | 21744795 | 22554808 | 23213684 | 24268530 | 26683904 | 28049773 | 29159516 | 29286627 | 30474524 |
| 20136837 | 21745120 | 22554840 | 23213830 | 24268669 | 26684282 | 28049783 | 29160177 | 29286641 | 30474981 |
| 20137085 | 21745124 | 22554844 | 23213982 | 24268675 | 26686776 | 28050236 | 29161023 | 29286643 | 30475643 |
| 20137456 | 21746034 | 22554878 | 23214060 | 24268712 | 26688127 | 28051687 | 29161320 | 29286647 | 30475895 |
| 20138220 | 21746783 | 22554931 | 23214306 | 24268859 | 26688249 | 28052117 | 29161809 | 29286659 | 30476025 |
| 20138522 | 21747195 | 22555869 | 23215250 | 24269248 | 26688963 | 28052251 | 29161972 | 29286664 | 30476909 |
| 20138540 | 21748120 | 22555969 | 23216018 | 24269265 | 26691555 | 28052569 | 29162373 | 29286665 | 30477243 |
| 20138845 | 21748121 | 22556444 | 23216155 | 24269868 | 26691582 | 28053793 | 29162567 | 29286670 | 30477264 |
| 20139046 | 21749246 | 22556909 | 23216201 | 24270204 | 26691859 | 28053856 | 29162848 | 29286677 | 30477283 |
| 20139821 | 21750523 | 22557005 | 23216895 | 24270655 | 26691865 | 28054029 | 29162991 | 29286706 | 30477330 |
| 20140005 | 21750641 | 22557052 | 23216971 | 24271256 | 26691876 | 28054253 | 29162998 | 29286731 | 30477476 |
| 20140018 | 21750770 | 22557302 | 23217293 | 24271501 | 26692303 | 28054891 | 29163019 | 29286739 | 30477755 |
| 20140118 | 21750862 | 22557712 | 23217526 | 24271657 | 26693581 | 28055715 | 29163228 | 29286740 | 30477866 |
| 20140135 | 21750964 | 22558090 | 23217886 | 24272184 | 26694209 | 28056022 | 29163702 | 29286746 | 30477929 |
| 20140329 | 21751214 | 22558105 | 23218097 | 24273073 | 26695109 | 28056210 | 29163922 | 29286758 | 30478505 |
| 20140680 | 21751235 | 22558830 | 23218208 | 24274181 | 26695206 | 28057161 | 29164098 | 29286765 | 30478733 |
| 20140736 | 21751242 | 22558855 | 23218235 | 24274892 | 26696209 | 28057610 | 29165170 | 29286780 | 30478873 |
| 20141224 | 21752732 | 22559154 | 23218262 | 24275042 | 26696986 | 28058394 | 29165187 | 29286781 | 30479988 |
| 20141628 | 21753168 | 22559201 | 23218268 | 24275223 | 26697164 | 28058507 | 29166175 | 29286797 | 30480113 |
| 20142665 | 21753503 | 22559249 | 23218433 | 24275242 | 26700214 | 28059020 | 29167056 | 29286806 | 30480128 |
| 20142776 | 21754593 | 22559420 | 23218671 | 24277250 | 26700908 | 28059487 | 29167451 | 29286816 | 30480956 |
| 20142977 | 21755584 | 22560066 | 23218902 | 24277862 | 26701601 | 28061924 | 29167539 | 29286818 | 30481471 |
| 20143625 | 21756566 | 22560188 | 23219241 | 24277967 | 26701965 | 28062114 | 29167810 | 29286820 | 30481533 |
| 20143698 | 21757266 | 22560361 | 23219686 | 24278552 | 26702258 | 28062231 | 29168217 | 29286826 | 30481984 |
| 20143962 | 21758705 | 22560812 | 23220069 | 24278583 | 26702653 | 28062297 | 29168234 | 29286828 | 30482006 |
| 20144232 | 21758738 | 22561122 | 23220155 | 24279070 | 26702804 | 28062415 | 29168351 | 29286840 | 30482915 |
| 20145128 | 21759512 | 22561470 | 23220543 | 24279211 | 26706449 | 28062779 | 29169737 | 29286842 | 30483241 |
| 20145746 | 21761043 | 22561665 | 23220547 | 24279443 | 26706709 | 28063045 | 29170328 | 29286844 | 30483521 |
| 20147789 | 21761790 | 22562582 | 23220632 | 24279820 | 26707164 | 28063058 | 29170415 | 29286864 | 30483680 |
| 20147919 | 21762083 | 22563890 | 23220956 | 24279849 | 26708993 | 28063103 | 29170550 | 29286868 | 30483833 |
| 20148008 | 21762168 | 22564596 | 23221001 | 24279925 | 26709414 | 28063168 | 29171468 | 29286875 | 30483906 |
| 20148055 | 21762284 | 22564634 | 23221016 | 24280965 | 26710536 | 28063178 | 29171586 | 29286877 | 30484012 |
| 20148345 | 21763381 | 22565196 | 23221202 | 24281130 | 26711958 | 28064100 | 29172288 | 29286881 | 30484200 |
| 20149114 | 21764434 | 22565711 | 23221573 | 24281380 | 26712072 | 28064116 | 29172941 | 29286886 | 30484643 |
| 20149159 | 21765556 | 22566811 | 23221579 | 24282179 | 26713498 | 28064134 | 29173481 | 29286892 | 30484772 |
| 20149203 | 21765655 | 22566938 | 23221644 | 24282225 | 26714237 | 28064211 | 29173616 | 29286895 | 30485089 |
| 20149319 | 21766724 | 22567448 | 23221838 | 24282230 | 26715672 | 28065184 | 29173717 | 29286910 | 30485486 |
| 20149441 | 21767453 | 22567790 | 23221956 | 24282921 | 26717116 | 28065437 | 29175390 | 29286920 | 30485603 |
| 20149481 | 21767455 | 22567855 | 23222113 | 24283568 | 26718562 | 28065799 | 29175478 | 29286965 | 30485610 |
| 20149523 | 21767507 | 22568083 | 23222171 | 24283587 | 26718915 | 28066945 | 29176193 | 29286976 | 30486138 |
| 20149845 | 21769425 | 22568713 | 23222280 | 24284728 | 26719862 | 28067596 | 29176628 | 29286978 | 30486239 |
| 20149958 | 21770878 | 22569054 | 23222360 | 24284861 | 26720476 | 28067790 | 29176707 | 29286991 | 30487634 |
| 20150475 | 21771504 | 22569294 | 23222914 | 24285049 | 26721072 | 28068359 | 29177068 | 29286997 | 30487723 |
| 20150908 | 21771813 | 22569362 | 23222919 | 24285360 | 26721273 | 28068438 | 29177230 | 29287006 | 30487977 |
| 20151067 | 21772816 | 22569395 | 23223059 | 24285399 | 26723439 | 28069046 | 29177303 | 29287016 | 30488214 |
| 20151275 | 21772890 | 22570621 | 23224002 | 24285493 | 26724130 | 28069728 | 29177660 | 29287019 | 30488905 |
| 20151346 | 21773575 | 22570676 | 23224258 | 24286237 | 26724529 | 28069956 | 29178163 | 29287031 | 30489524 |
| 20151805 | 21773807 | 22570765 | 23224731 | 24286595 | 26726629 | 28070065 | 29178283 | 29287032 | 30489770 |
| 20152168 | 21774274 | 22571391 | 23224796 | 24286714 | 26727148 | 28070622 | 29178556 | 29287041 | 30490275 |
| 20152419 | 21774304 | 22571447 | 23224830 | 24287007 | 26727384 | 28070627 | 29178687 | 29287058 | 30490657 |
| 20153044 | 21774841 | 22571466 | 23225211 | 24287025 | 26728490 | 28071431 | 29178764 | 29287059 | 30491486 |
| 20153337 | 21774953 | 22571539 | 23225728 | 24287436 | 26728534 | 28071654 | 29179064 | 29287065 | 30491818 |
| 20153387 | 21775309 | 22571617 | 23225866 | 24287452 | 26728725 | 28071689 | 29179494 | 29287070 | 30492506 |
| 20153578 | 21775767 | 22571728 | 23226010 | 24287994 | 26730044 | 28071962 | 29179518 | 29287090 | 30493160 |
| 20153819 | 21776546 | 22572288 | 23226364 | 24288848 | 26731143 | 28073146 | 29179525 | 29287096 | 30493266 |
| 20153981 | 21776724 | 22572316 | 23226464 | 24289888 | 26731606 | 28073154 | 29179793 | 29287099 | 30493373 |
| 20154238 | 21776999 | 22572347 | 23227023 | 24289923 | 26733127 | 28073313 | 29181167 | 29287101 | 30493903 |
| 20155556 | 21778147 | 22572806 | 23227110 | 24290086 | 26733744 | 28073786 | 29181384 | 29287115 | 30494203 |
| 20155869 | 21778587 | 22573212 | 23227192 | 24290232 | 26736707 | 28074467 | 29181499 | 29287120 | 30494917 |
| 20155947 | 21778615 | 22573330 | 23227277 | 24290661 | 26736911 | 28074851 | 29183314 | 29287123 | 30495423 |
| 20156872 | 21778633 | 22573634 | 23227320 | 24290736 | 26737013 | 28074907 | 29183590 | 29287125 | 30495513 |
| 20156999 | 21779144 | 22573660 | 23227803 | 24290823 | 26737171 | 28075530 | 29184266 | 29287126 | 30495589 |
| 20158175 | 21780279 | 22573690 | 23228101 | 24291161 | 26737504 | 28076643 | 29184297 | 29287133 | 30495859 |
| 20158434 | 21780606 | 22573843 | 23228118 | 24292037 | 26738425 | 28076740 | 29185359 | 29287144 | 30496233 |
| 20158865 | 21780955 | 22574018 | 23228148 | 24292151 | 26739376 | 28077258 | 29185425 | 29287146 | 30496834 |
| 20159432 | 21781727 | 22574066 | 23228202 | 24292730 | 26740002 | 28077788 | 29185511 | 29287155 | 30497011 |
| 20159442 | 21781767 | 22574601 | 23228483 | 24293144 | 26740073 | 28078472 | 29186166 | 29287157 | 30497171 |
| 20159447 | 21782366 | 22574674 | 23229710 | 24293459 | 26740311 | 28078897 | 29186255 | 29287164 | 30498599 |
| 20159625 | 21782394 | 22574956 | 23229720 | 24293971 | 26742063 | 28079174 | 29186313 | 29287167 | 30498622 |
| 20159708 | 21782454 | 22574996 | 23229795 | 24294246 | 26742663 | 28079379 | 29186604 | 29287178 | 30498961 |
| 20159877 | 21783352 | 22575025 | 23229911 | 24294503 | 26742962 | 28079386 | 29186741 | 29287183 | 30499487 |
| 20160100 | 21783841 | 22576228 | 23230313 | 24294677 | 26743542 | 28079529 | 29187070 | 29287184 | 30499653 |
| 20161007 | 21784390 | 22576719 | 23230710 | 24295507 | 26744954 | 28079808 | 29187423 | 29287186 | 30499814 |
| 20161864 | 21784731 | 22576869 | 23231352 | 24295673 | 26745799 | 28080221 | 29188195 | 29287191 | 30500458 |
| 20162678 | 21784977 | 22578411 | 23232613 | 24296383 | 26746428 | 28080299 | 29188564 | 29287196 | 30500486 |
| 20163492 | 21785028 | 22578990 | 23232677 | 24296912 | 26747630 | 28080530 | 29188882 | 29287203 | 30501187 |
| 20163782 | 21785391 | 22579061 | 23232933 | 24297529 | 26747726 | 28080689 | 29189104 | 29287223 | 30501202 |
| 20163865 | 21786701 | 22579075 | 23232947 | 24297809 | 26747769 | 28082568 | 29189945 | 29287225 | 30501262 |
| 20163949 | 21786743 | 22579681 | 23233791 | 24297962 | 26747797 | 28082668 | 29189957 | 29287231 | 30501305 |
| 20164819 | 21787515 | 22580028 | 23233856 | 24297973 | 26748649 | 28082869 | 29190117 | 29287232 | 30502094 |
| 20164959 | 21787941 | 22580094 | 23233988 | 24298116 | 26749616 | 28083371 | 29191208 | 29287234 | 30502161 |
| 20165834 | 21788317 | 22580230 | 23234300 | 24298137 | 26749956 | 28083573 | 29192726 | 29287249 | 30502389 |
| 20166088 | 21789136 | 22580346 | 23234328 | 24298171 | 26752072 | 28083899 | 29193187 | 29287251 | 30502676 |
| 20166590 | 21789370 | 22581029 | 23234520 | 24298365 | 26753264 | 28084524 | 29193741 | 29287258 | 30502940 |
| 20166617 | 21789910 | 22581176 | 23234689 | 24300710 | 26754102 | 28084769 | 29194043 | 29287264 | 30503091 |
| 20166893 | 21790731 | 22581401 | 23234769 | 24301546 | 26754882 | 28084930 | 29194267 | 29287268 | 30503208 |
| 20167575 | 21790774 | 22581965 | 23235245 | 24301740 | 26757223 | 28085022 | 29194630 | 29287277 | 30503553 |
| 20168293 | 21791020 | 22581983 | 23235552 | 24302661 | 26758570 | 28085681 | 29194815 | 29287279 | 30504003 |
| 20168453 | 21791038 | 22582006 | 23235557 | 24302713 | 26759272 | 28085957 | 29194848 | 29287283 | 30504069 |
| 20168766 | 21791591 | 22582181 | 23235571 | 24303225 | 26760275 | 28086185 | 29194866 | 29287288 | 30505401 |
| 20169208 | 21792949 | 22582547 | 23235587 | 24303929 | 26762355 | 28086414 | 29194946 | 29287302 | 30505783 |
| 20170132 | 21793279 | 22582693 | 23235757 | 24304512 | 26763203 | 28087244 | 29196314 | 29287331 | 30505832 |
| 20170246 | 21793289 | 22582704 | 23235810 | 24305304 | 26763519 | 28087548 | 29196572 | 29287340 | 30506394 |
| 20170274 | 21794134 | 22582746 | 23236103 | 24305912 | 26763715 | 28087867 | 29196820 | 29287354 | 30507294 |
| 20170523 | 21794415 | 22582839 | 23236161 | 24306334 | 26764034 | 28088020 | 29197234 | 29287356 | 30507833 |
| 20171060 | 21796160 | 22583114 | 23236603 | 24306663 | 26765475 | 28088642 | 29197923 | 29287370 | 30507977 |
| 20171414 | 21796291 | 22583517 | 23236735 | 24307115 | 26767005 | 28088731 | 29197949 | 29287378 | 30508217 |
| 20171652 | 21797311 | 22584497 | 23236975 | 24307267 | 26767068 | 28089100 | 29198606 | 29287406 | 30509550 |
| 20171859 | 21797471 | 22584884 | 23236976 | 24307447 | 26767979 | 28089105 | 29198683 | 29287408 | 30509824 |
| 20172316 | 21797862 | 22585299 | 23237317 | 24307573 | 26769971 | 28089273 | 29198983 | 29287412 | 30510380 |
| 20172322 | 21798563 | 22585517 | 23237334 | 24307703 | 26772654 | 28089682 | 29199092 | 29287428 | 30510395 |
| 20172678 | 21800404 | 22585633 | 23238034 | 24308876 | 26773323 | 28089730 | 29199186 | 29287429 | 30510402 |
| 20173455 | 21801402 | 22585685 | 23238229 | 24309216 | 26773696 | 28089777 | 29199287 | 29287431 | 30510606 |
| 20173692 | 21801498 | 22585733 | 23238337 | 24309333 | 26774841 | 28090372 | 29199314 | 29287433 | 30511126 |
| 20173892 | 21802487 | 22585929 | 23238546 | 24309426 | 26774891 | 28090457 | 29199392 | 29287438 | 30511198 |
| 20174791 | 21803511 | 22586048 | 23238671 | 24309447 | 26775789 | 28091146 | 29199531 | 29287449 | 30511449 |
| 20175173 | 21803745 | 22586391 | 23238794 | 24310186 | 26776151 | 28091266 | 29199557 | 29287450 | 30511954 |
| 20175411 | 21804559 | 22586902 | 23238900 | 24310503 | 26782907 | 28092540 | 29200498 | 29287467 | 30512693 |
| 20175609 | 21807093 | 22587072 | 23239005 | 24311425 | 26783603 | 28092641 | 29201152 | 29287478 | 30514080 |
| 20175720 | 21807165 | 22587093 | 23239186 | 24311921 | 26784741 | 28092692 | 29201542 | 29287488 | 30516787 |
| 20175731 | 21807349 | 22587551 | 23239216 | 24312164 | 26785065 | 28093197 | 29201833 | 29287492 | 30516865 |
| 20175981 | 21807593 | 22587621 | 23239243 | 24313322 | 26786265 | 28093718 | 29202311 | 29287495 | 30517129 |
| 20176555 | 21807943 | 22587809 | 23239265 | 24313497 | 26788157 | 28093909 | 29202464 | 29287517 | 30518679 |
| 20176595 | 21807947 | 22587861 | 23239432 | 24313905 | 26788876 | 28094925 | 29202554 | 29287523 | 30519540 |
| 20176900 | 21808501 | 22588252 | 23239587 | 24314243 | 26789117 | 28094964 | 29202714 | 29287525 | 30519652 |
| 20177368 | 21808985 | 22588302 | 23239808 | 24314736 | 26789375 | 28095057 | 29203609 | 29287534 | 30520023 |
| 20177511 | 21809405 | 22588539 | 23240202 | 24314751 | 26789715 | 28095705 | 29203632 | 29287541 | 30520146 |
| 20177547 | 21809782 | 22588925 | 23240454 | 24315310 | 26790490 | 28096194 | 29203906 | 29287554 | 30520370 |
| 20177551 | 21810203 | 22589026 | 23240464 | 24315701 | 26792209 | 28096433 | 29204146 | 29287559 | 30520438 |
| 20177622 | 21810906 | 22589356 | 23240805 | 24316097 | 26794637 | 28096602 | 29204249 | 29287567 | 30520538 |
| 20177908 | 21811292 | 22589417 | 23241486 | 24317574 | 26796405 | 28096819 | 29204458 | 29287579 | 30520810 |
| 20177944 | 21811549 | 22589466 | 23241716 | 24318433 | 26799338 | 28097907 | 29204486 | 29287583 | 30520894 |
| 20177965 | 21811949 | 22589561 | 23242094 | 24318589 | 26800272 | 28098143 | 29205967 | 29287587 | 30520978 |
| 20178329 | 21811988 | 22589565 | 23242326 | 24318632 | 26800489 | 28098782 | 29206211 | 29287605 | 30523028 |
| 20178540 | 21812191 | 22589881 | 23242371 | 24319529 | 26800783 | 28099488 | 29206893 | 29287610 | 30523361 |
| 20178826 | 21812689 | 22589990 | 23242512 | 24319566 | 26801162 | 28099905 | 29206944 | 29287618 | 30523577 |
| 20179036 | 21812708 | 22590016 | 23242860 | 24319658 | 26801205 | 28099938 | 29207303 | 29287622 | 30525381 |
| 20179042 | 21812964 | 22590262 | 23243132 | 24319791 | 26801731 | 28100518 | 29207512 | 29287629 | 30526480 |
| 20179144 | 21814041 | 22591123 | 23243401 | 24321815 | 26802431 | 28100773 | 29207648 | 29287637 | 30527706 |
| 20179545 | 21815328 | 22591556 | 23243410 | 24321893 | 26804622 | 28101249 | 29207652 | 29287638 | 30527719 |
| 20179885 | 21815953 | 22591786 | 23243888 | 24322002 | 26805768 | 28101954 | 29208062 | 29287640 | 30527991 |
| 20180149 | 21816712 | 22592282 | 23243948 | 24322729 | 26805966 | 28101970 | 29208917 | 29287644 | 30528248 |
| 20180464 | 21817570 | 22592753 | 23244292 | 24323060 | 26806613 | 28102757 | 29209180 | 29287650 | 30528996 |
| 20180793 | 21817899 | 22592964 | 23244521 | 24323534 | 26806806 | 28102819 | 29209320 | 29287657 | 30529059 |
| 20180989 | 21818119 | 22593469 | 23244808 | 24323717 | 26807669 | 28103017 | 29209429 | 29287665 | 30529402 |
| 20181018 | 21818211 | 22593562 | 23244904 | 24324160 | 26807987 | 28103071 | 29209850 | 29287666 | 30529568 |
| 20181026 | 21819577 | 22594317 | 23245016 | 24324188 | 26808343 | 28103749 | 29210106 | 29287672 | 30529660 |
| 20181479 | 21820474 | 22594330 | 23245136 | 24324979 | 26809423 | 28104026 | 29210179 | 29287674 | 30529751 |
| 20181596 | 21820951 | 22594496 | 23245274 | 24325113 | 26809467 | 28104358 | 29210216 | 29287679 | 30529761 |
| 20182113 | 21823372 | 22594506 | 23245745 | 24325186 | 26809496 | 28104847 | 29210255 | 29287687 | 30530061 |
| 20182586 | 21823527 | 22594889 | 23245895 | 24326084 | 26809944 | 28105242 | 29210444 | 29287694 | 30530170 |
| 20182722 | 21823622 | 22595315 | 23246312 | 24326588 | 26811075 | 28105711 | 29210487 | 29287701 | 30531083 |
| 20182849 | 21825003 | 22595495 | 23246469 | 24326685 | 26811193 | 28105880 | 29210564 | 29287722 | 30531662 |
| 20183080 | 21825136 | 22595773 | 23246587 | 24326874 | 26811279 | 28106105 | 29210630 | 29287723 | 30532110 |
| 20183153 | 21826123 | 22596072 | 23246847 | 24326941 | 26811958 | 28106491 | 29210653 | 29287727 | 30532425 |
| 20183248 | 21826449 | 22596645 | 23246896 | 24327008 | 26812976 | 28106995 | 29210910 | 29287743 | 30532480 |
| 20183857 | 21826715 | 22596943 | 23246990 | 24327289 | 26813538 | 28107165 | 29211030 | 29287744 | 30532909 |
| 20184184 | 21827595 | 22597018 | 23247439 | 24327546 | 26813988 | 28107862 | 29211039 | 29287749 | 30533426 |
| 20184510 | 21828639 | 22597374 | 23247864 | 24327604 | 26815599 | 28108090 | 29211268 | 29287751 | 30533576 |
| 20184613 | 21829025 | 22598139 | 23247893 | 24327641 | 26815853 | 28108394 | 29212023 | 29287753 | 30533583 |
| 20184939 | 21829267 | 22598509 | 23247921 | 24327813 | 26816203 | 28108585 | 29212356 | 29287760 | 30533649 |
| 20184968 | 21829830 | 22598889 | 23248014 | 24328750 | 26816286 | 28108910 | 29212392 | 29287782 | 30534908 |
| 20185641 | 21830083 | 22599016 | 23248044 | 24328923 | 26817587 | 28109219 | 29212396 | 29287786 | 30534955 |
| 20185993 | 21830647 | 22600290 | 23248047 | 24329158 | 26818053 | 28109524 | 29212606 | 29287790 | 30535461 |
| 20186984 | 21830893 | 22600466 | 23248279 | 24329285 | 26818141 | 28110373 | 29213247 | 29287802 | 30535679 |
| 20187080 | 21831023 | 22600697 | 23248287 | 24330268 | 26818271 | 28111291 | 29213635 | 29287803 | 30535765 |
| 20187663 | 21831520 | 22600957 | 23248636 | 24330461 | 26818331 | 28113310 | 29214031 | 29287811 | 30535959 |
| 20187793 | 21831792 | 22600997 | 23248986 | 24330778 | 26818825 | 28113897 | 29214301 | 29287818 | 30536763 |
| 20188068 | 21832197 | 22601588 | 23249262 | 24331139 | 26819909 | 28113983 | 29214504 | 29287821 | 30536873 |
| 20188211 | 21832282 | 22602114 | 23249419 | 24331207 | 26820635 | 28114127 | 29214882 | 29287840 | 30537019 |
| 20189131 | 21832869 | 22602332 | 23249742 | 24331221 | 26820671 | 28114331 | 29215182 | 29287869 | 30537826 |
| 20189154 | 21832886 | 22602536 | 23249976 | 24331375 | 26820809 | 28114475 | 29215495 | 29287890 | 30538192 |
| 20189308 | 21833477 | 22602956 | 23250109 | 24331985 | 26821293 | 28115021 | 29215764 | 29287912 | 30538808 |
| 20189518 | 21834992 | 22603311 | 23250498 | 24332088 | 26823896 | 28115165 | 29215913 | 29287923 | 30539051 |
| 20189609 | 21835270 | 22603689 | 23250566 | 24333378 | 26825398 | 28115554 | 29216035 | 29287925 | 30539356 |
| 20190107 | 21835869 | 22603793 | 23250804 | 24333755 | 26826097 | 28115643 | 29216380 | 29287934 | 30539521 |
| 20190177 | 21836170 | 22604043 | 23250805 | 24334058 | 26826182 | 28116412 | 29216642 | 29287940 | 30541300 |
| 20192296 | 21836201 | 22604119 | 23250950 | 24335474 | 26827841 | 28116459 | 29217314 | 29287966 | 30541544 |
| 20192809 | 21836368 | 22604378 | 23251103 | 24336143 | 26828679 | 28116601 | 29217532 | 29287969 | 30542427 |
| 20193153 | 21836641 | 22604575 | 23251442 | 24336753 | 26828835 | 28117284 | 29218707 | 29287979 | 30542898 |
| 20193736 | 21836916 | 22604647 | 23251773 | 24336813 | 26829898 | 28117328 | 29219401 | 29287984 | 30544903 |
| 20194043 | 21837343 | 22605042 | 23251774 | 24337188 | 26832208 | 28117497 | 29219863 | 29287996 | 30545171 |
| 20194211 | 21837593 | 22605271 | 23252037 | 24338943 | 26832693 | 28117853 | 29220734 | 29287997 | 30545172 |
| 20194493 | 21837762 | 22605315 | 23252374 | 24339502 | 26832921 | 28118034 | 29221227 | 29288024 | 30545286 |
| 20194788 | 21837963 | 22605929 | 23252439 | 24339974 | 26834184 | 28118524 | 29221601 | 29288029 | 30545584 |
| 20195465 | 21838702 | 22606051 | 23252565 | 24340191 | 26834266 | 28119866 | 29221620 | 29288035 | 30546074 |
| 20196529 | 21838896 | 22606113 | 23252571 | 24341182 | 26834542 | 28119934 | 29222219 | 29288059 | 30546094 |
| 20196910 | 21838957 | 22606334 | 23252799 | 24341212 | 26835068 | 28120439 | 29222887 | 29288071 | 30546713 |
| 20197781 | 21839937 | 22606536 | 23252930 | 24341274 | 26835697 | 28121397 | 29222982 | 29288074 | 30547242 |
| 20197798 | 21840337 | 22606679 | 23252943 | 24341811 | 26835953 | 28121607 | 29223202 | 29288077 | 30547665 |
| 20199116 | 21840750 | 22606890 | 23253012 | 24341973 | 26836166 | 28122285 | 29223900 | 29288084 | 30547908 |
| 20199344 | 21841197 | 22607321 | 23253063 | 24342194 | 26836286 | 28122288 | 29225051 | 29288085 | 30548172 |
| 20199573 | 21841231 | 22607503 | 23253126 | 24342440 | 26836841 | 28122400 | 29225776 | 29288098 | 30548750 |
| 20199887 | 21841814 | 22608297 | 23253698 | 24343270 | 26838864 | 28122537 | 29226070 | 29288102 | 30549037 |
| 20200883 | 21842666 | 22608307 | 23253850 | 24343684 | 26839015 | 28122805 | 29226183 | 29288116 | 30549366 |
| 20200924 | 21842924 | 22608323 | 23254210 | 24343784 | 26839271 | 28122935 | 29226243 | 29288122 | 30549449 |
| 20201797 | 21842931 | 22608567 | 23254213 | 24344367 | 26840074 | 28124743 | 29226299 | 29288124 | 30549638 |
| 20201821 | 21843156 | 22609066 | 23254283 | 24344956 | 26843682 | 28126175 | 29226817 | 29288135 | 30549878 |
| 20202715 | 21843602 | 22609527 | 23254536 | 24344959 | 26843988 | 28126632 | 29227011 | 29288140 | 30550150 |
| 20203408 | 21843684 | 22609982 | 23254546 | 24345177 | 26844192 | 28126771 | 29227135 | 29288146 | 30551485 |
| 20203970 | 21844308 | 22610298 | 23254744 | 24345692 | 26845195 | 28127551 | 29227876 | 29288148 | 30552066 |
| 20204080 | 21844731 | 22610422 | 23254999 | 24346116 | 26845762 | 28127736 | 29227921 | 29288159 | 30552264 |
| 20204204 | 21844768 | 22610455 | 23255034 | 24346233 | 26846274 | 28127825 | 29228246 | 29288162 | 30553159 |
| 20205128 | 21845297 | 22610767 | 23255059 | 24346930 | 26846398 | 28127879 | 29228280 | 29288175 | 30553476 |
| 20205310 | 21846107 | 22610779 | 23255430 | 24347096 | 26847343 | 28128687 | 29228889 | 29288180 | 30553558 |
| 20205817 | 21847299 | 22610980 | 23255487 | 24347492 | 26848313 | 28129744 | 29229546 | 29288197 | 30554363 |
| 20206684 | 21848681 | 22611110 | 23255503 | 24348347 | 26848889 | 28129777 | 29229615 | 29288234 | 30556322 |
| 20207309 | 21848967 | 22611241 | 23255611 | 24348528 | 26850786 | 28130008 | 29229799 | 29288239 | 30556981 |
| 20207312 | 21849308 | 22611419 | 23256222 | 24348740 | 26852218 | 28130126 | 29230025 | 29288267 | 30557180 |
| 20207700 | 21849679 | 22611570 | 23256320 | 24349003 | 26852970 | 28130347 | 29230305 | 29288272 | 30557573 |
| 20207784 | 21849706 | 22612327 | 23257075 | 24349749 | 26853583 | 28130382 | 29230308 | 29288275 | 30557939 |
| 20208036 | 21850279 | 22612439 | 23257241 | 24349938 | 26854302 | 28130431 | 29231444 | 29288285 | 30558706 |
| 20208304 | 21850417 | 22612655 | 23257415 | 24350326 | 26854418 | 28131086 | 29231590 | 29288287 | 30558968 |
| 20209517 | 21851456 | 22612710 | 23257448 | 24351529 | 26854622 | 28131505 | 29231686 | 29288311 | 30559018 |
| 20209690 | 21852252 | 22613131 | 23257671 | 24351674 | 26855617 | 28131711 | 29232265 | 29288316 | 30559339 |
| 20209953 | 21852768 | 22613358 | 23257699 | 24351994 | 26856085 | 28131716 | 29232629 | 29288328 | 30559620 |
| 20210227 | 21853280 | 22614754 | 23257835 | 24352093 | 26857833 | 28131886 | 29232992 | 29288334 | 30559690 |
| 20210606 | 21854542 | 22614777 | 23257932 | 24352172 | 26860017 | 28132150 | 29233855 | 29288374 | 30560395 |
| 20210628 | 21855230 | 22614855 | 23257978 | 24353007 | 26860620 | 28132251 | 29233943 | 29288378 | 30560513 |
| 20210720 | 21855243 | 22615288 | 23258147 | 24353298 | 26861268 | 28132355 | 29233944 | 29288381 | 30560631 |
| 20210742 | 21855424 | 22615359 | 23258219 | 24353691 | 26861885 | 28132947 | 29234127 | 29288390 | 30562050 |
| 20211134 | 21855739 | 22615489 | 23258311 | 24354137 | 26862052 | 28133421 | 29234561 | 29288393 | 30562130 |
| 20211666 | 21856045 | 22615711 | 23259189 | 24354253 | 26863455 | 28133591 | 29234712 | 29288399 | 30562172 |
| 20211881 | 21856634 | 22615748 | 23259354 | 24354319 | 26863732 | 28133615 | 29234910 | 29288403 | 30562334 |
| 20212917 | 21857019 | 22615843 | 23259454 | 24354609 | 26864587 | 28135069 | 29235368 | 29288406 | 30563018 |
| 20213040 | 21857408 | 22615924 | 23259542 | 24354888 | 26864684 | 28135107 | 29235602 | 29288412 | 30563056 |
| 20213266 | 21857605 | 22616053 | 23259666 | 24355379 | 26864713 | 28135436 | 29235643 | 29288421 | 30563279 |
| 20213270 | 21858175 | 22616057 | 23259677 | 24355540 | 26866630 | 28135537 | 29235719 | 29288448 | 30563520 |
| 20213624 | 21858613 | 22616248 | 23259687 | 24355842 | 26867334 | 28136434 | 29237386 | 29288468 | 30563536 |
| 20214391 | 21858762 | 22616269 | 23260044 | 24355915 | 26868431 | 28136878 | 29237633 | 29288469 | 30563537 |
| 20214409 | 21859983 | 22616361 | 23260170 | 24356017 | 26869457 | 28137222 | 29237729 | 29288479 | 30563575 |
| 20214943 | 21861158 | 22616506 | 23260446 | 24356599 | 26869705 | 28137470 | 29237927 | 29288492 | 30563624 |
| 20215528 | 21861557 | 22616514 | 23260726 | 24356794 | 26871479 | 28138476 | 29239033 | 29288493 | 30564696 |
| 20215729 | 21862141 | 22616518 | 23260737 | 24357083 | 26872095 | 28139219 | 29241339 | 29288501 | 30564827 |
| 20215782 | 21862670 | 22616890 | 23260945 | 24357164 | 26872906 | 28139341 | 29241671 | 29288507 | 30564958 |
| 20215806 | 21862765 | 22617014 | 23261061 | 24357237 | 26876123 | 28139751 | 29241866 | 29288515 | 30565663 |
| 20215855 | 21863210 | 22617191 | 23261107 | 24357382 | 26878375 | 28140001 | 29242164 | 29288518 | 30566944 |
| 20216218 | 21864929 | 22617328 | 23261149 | 24357588 | 26878693 | 28140471 | 29242582 | 29288523 | 30567195 |
| 20216439 | 21865757 | 22617473 | 23261298 | 24358007 | 26880327 | 28140577 | 29242952 | 29288527 | 30567377 |
| 20216441 | 21866473 | 22617727 | 23261774 | 24358265 | 26880709 | 28140641 | 29243530 | 29288536 | 30567905 |
| 20216533 | 21867301 | 22617889 | 23261984 | 24359096 | 26880898 | 28140714 | 29243808 | 29288537 | 30568896 |
| 20217352 | 21867595 | 22618128 | 23262226 | 24359624 | 26882317 | 28140840 | 29244260 | 29288548 | 30569690 |
| 20217554 | 21867756 | 22618339 | 23262303 | 24360157 | 26882668 | 28141358 | 29244596 | 29288557 | 30570003 |
| 20217714 | 21867877 | 22618668 | 23262400 | 24360180 | 26884349 | 28141540 | 29244740 | 29288561 | 30570476 |
| 20217855 | 21868759 | 22618755 | 23262780 | 24361272 | 26886804 | 28141620 | 29245123 | 29288562 | 30570959 |
| 20218200 | 21868924 | 22618792 | 23262954 | 24361317 | 26887176 | 28142176 | 29245214 | 29288563 | 30571285 |
| 20218747 | 21869278 | 22618918 | 23263012 | 24361437 | 26887513 | 28143550 | 29246180 | 29288581 | 30571475 |
| 20218836 | 21870188 | 22619103 | 23263413 | 24361711 | 26887873 | 28143661 | 29246235 | 29288597 | 30572110 |
| 20218969 | 21870221 | 22619134 | 23263510 | 24361855 | 26888111 | 28143695 | 29246274 | 29288600 | 30573100 |
| 20219426 | 21870783 | 22619418 | 23263658 | 24361921 | 26889214 | 28143819 | 29246591 | 29288611 | 30573635 |
| 20219666 | 21870923 | 22619437 | 23263692 | 24362001 | 26889678 | 28143866 | 29247101 | 29288624 | 30573900 |
| 20219768 | 21870930 | 22620058 | 23263719 | 24362086 | 26889700 | 28144119 | 29247228 | 29288649 | 30573939 |
| 20220136 | 21872136 | 22620284 | 23263737 | 24362427 | 26890553 | 28144621 | 29247339 | 29288652 | 30574506 |
| 20220336 | 21872180 | 22620556 | 23264068 | 24362815 | 26892793 | 28144760 | 29247444 | 29288662 | 30574634 |
| 20220388 | 21872691 | 22620731 | 23264098 | 24362961 | 26893501 | 28145049 | 29247478 | 29288678 | 30574750 |
| 20221183 | 21873132 | 22621298 | 23264180 | 24363438 | 26893923 | 28145212 | 29248313 | 29288689 | 30574925 |
| 20221781 | 21873774 | 22621541 | 23264415 | 24364180 | 26894318 | 28145378 | 29248526 | 29288692 | 30575282 |
| 20221793 | 21873949 | 22621596 | 23264839 | 24364249 | 26895689 | 28145704 | 29248654 | 29288694 | 30576368 |
| 20222429 | 21874284 | 22621656 | 23265767 | 24364335 | 26895867 | 28148398 | 29248864 | 29288713 | 30576678 |
| 20222595 | 21875033 | 22621837 | 23265824 | 24365388 | 26896441 | 28148643 | 29248871 | 29288716 | 30576733 |
| 20222627 | 21875114 | 22622035 | 23266059 | 24366074 | 26897960 | 28149449 | 29249068 | 29288720 | 30577157 |
| 20223343 | 21875354 | 22623232 | 23266242 | 24366291 | 26900532 | 28149535 | 29249636 | 29288721 | 30577294 |
| 20224021 | 21875485 | 22623250 | 23266486 | 24366348 | 26901102 | 28149935 | 29249801 | 29288722 | 30577418 |
| 20224036 | 21875978 | 22623402 | 23266500 | 24367114 | 26901158 | 28150757 | 29250918 | 29288738 | 30577693 |
| 20224154 | 21876243 | 22623775 | 23266575 | 24367533 | 26901514 | 28150938 | 29250936 | 29288747 | 30578100 |
| 20224325 | 21876542 | 22623780 | 23266815 | 24367846 | 26903628 | 28151236 | 29251830 | 29288751 | 30578275 |
| 20224860 | 21876594 | 22624155 | 23267257 | 24367877 | 26904195 | 28151395 | 29251872 | 29288763 | 30578715 |
| 20225004 | 21876651 | 22624533 | 23267312 | 24368102 | 26904500 | 28151555 | 29251903 | 29288764 | 30578725 |
| 20225103 | 21877063 | 22624695 | 23267931 | 24368501 | 26905016 | 28151895 | 29252522 | 29288772 | 30578896 |
| 20225297 | 21877964 | 22624768 | 23268173 | 24368887 | 26905536 | 28151924 | 29252643 | 29288775 | 30579544 |
| 20225503 | 21878028 | 22624813 | 23268245 | 24369051 | 26905681 | 28151929 | 29252917 | 29288786 | 30580100 |
| 20227246 | 21878416 | 22624882 | 23268289 | 24369314 | 26907031 | 28152265 | 29253315 | 29288799 | 30580489 |
| 20227651 | 21879035 | 22624888 | 23268486 | 24369334 | 26907625 | 28153117 | 29253800 | 29288821 | 30580581 |
| 20227800 | 21880692 | 22624915 | 23268831 | 24369413 | 26908250 | 28153472 | 29254038 | 29288829 | 30582359 |
| 20228207 | 21881035 | 22625197 | 23268874 | 24370400 | 26909987 | 28153497 | 29254273 | 29288855 | 30582776 |
| 20229022 | 21881117 | 22625287 | 23269074 | 24370573 | 26910290 | 28154062 | 29254445 | 29288873 | 30583850 |
| 20229070 | 21881212 | 22625305 | 23269179 | 24370797 | 26910776 | 28154176 | 29256083 | 29288874 | 30584088 |
| 20229285 | 21881502 | 22625708 | 23270406 | 24371088 | 26911595 | 28154925 | 29256188 | 29288881 | 30584117 |
| 20229611 | 21883453 | 22626123 | 23270503 | 24371224 | 26912948 | 28155049 | 29256410 | 29288884 | 30584371 |
| 20229754 | 21883605 | 22626670 | 23270647 | 24371317 | 26913732 | 28155840 | 29256672 | 29288887 | 30584449 |
| 20229925 | 21884319 | 22626697 | 23270688 | 24372052 | 26913751 | 28155931 | 29256746 | 29288891 | 30585617 |
| 20230073 | 21884768 | 22626840 | 23270771 | 24372720 | 26915020 | 28155971 | 29257046 | 29288892 | 30585676 |
| 20230525 | 21884863 | 22626847 | 23271040 | 24373149 | 26915048 | 28156403 | 29257093 | 29288894 | 30586306 |
| 20230907 | 21885096 | 22626878 | 23271063 | 24373473 | 26915787 | 28156413 | 29257498 | 29288909 | 30586360 |
| 20230943 | 21885153 | 22627039 | 23271503 | 24373821 | 26916586 | 28157304 | 29258619 | 29288912 | 30586761 |
| 20230973 | 21885179 | 22627147 | 23271854 | 24374481 | 26916722 | 28158819 | 29258642 | 29288916 | 30586762 |
| 20231088 | 21886377 | 22628351 | 23271875 | 24375923 | 26916816 | 28158827 | 29258776 | 29288919 | 30587008 |
| 20231604 | 21886726 | 22628476 | 23272593 | 24375939 | 26916988 | 28159250 | 29259208 | 29288920 | 30588033 |
| 20232063 | 21887200 | 22628840 | 23273126 | 24376555 | 26917674 | 28159573 | 29259505 | 29288923 | 30589789 |
| 20232862 | 21887945 | 22628886 | 23273612 | 24376969 | 26918753 | 28160200 | 29260482 | 29288933 | 30590039 |
| 20233748 | 21889107 | 22628899 | 23274008 | 24376987 | 26919130 | 28160246 | 29260487 | 29288936 | 30590513 |
| 20233970 | 21889302 | 22629058 | 23274046 | 24377269 | 26919239 | 28160340 | 29260841 | 29288942 | 30590792 |
| 20234148 | 21890389 | 22629098 | 23274187 | 24377305 | 26919548 | 28160360 | 29261804 | 29288945 | 30591391 |
| 20234449 | 21890418 | 22629565 | 23274538 | 24377603 | 26921148 | 28160399 | 29262378 | 29288977 | 30592185 |
| 20235069 | 21891417 | 22629582 | 23274863 | 24377886 | 26921404 | 28161755 | 29262608 | 29288989 | 30592702 |
| 20235603 | 21892091 | 22629735 | 23275253 | 24378374 | 26921901 | 28162843 | 29263107 | 29288995 | 30593078 |
| 20235872 | 21892467 | 22630688 | 23275355 | 24378682 | 26922210 | 28163300 | 29263965 | 29288997 | 30593590 |
| 20236273 | 21892504 | 22631293 | 23275609 | 24378975 | 26923243 | 28163308 | 29264264 | 29289005 | 30594084 |
| 20236894 | 21892780 | 22631477 | 23275754 | 24379310 | 26923305 | 28163482 | 29264507 | 29289024 | 30594396 |
| 20237125 | 21893644 | 22631579 | 23275931 | 24379335 | 26924987 | 28163553 | 29264652 | 29289030 | 30594654 |
