# Supplementary material for: Publication speed in pharmacy practice journals: A comparative analysis
Source: PLoS One. 2021 Jun 29;16(6):e0253713. doi: 10.1371/journal.pone.0253713 (PMC8241115; doi:10.1371/journal.pone.0253713)
Supplement: S2 Appendix — (DOCX) [file pone.0253713.s002.docx]

**Publication speed in pharmacy practice journals: a comparative analysis**

**Supporting information 2. Articles published in the pharmacy practice journals between 2009 and 2018**

Antonio M. MENDES, Fernanda S. TONIN, Roberto PONTAROLO, Fernando FERNANDEZ-LLIMOS.

| **Journal** | **Publication year** | | | | | | | | | | |
| --- | --- | --- | --- | --- | --- | --- | --- | --- | --- | --- | --- |
|  | **2009** | **2010** | **2011** | **2012** | **2013** | **2014** | **2015** | **2016** | **2017** | **2018** | **TOTAL** |
| Am J Health Syst Pharm | 373 | 380 | 360 | 346 | 320 | 344 | 375 | 382 | 371 | 343 | 3594 |
| Am J Pharm Educ | 167 | 210 | 211 | 200 | 232 | 195 | 171 | 204 | 193 | 165 | 1948 |
| Ann Pharm Fr | 58 | 40 | 46 | 46 | 49 | 48 | 51 | 49 | 50 | 56 | 493 |
| Can J Clin Pharmacol | 40 | 12 | 0 | 0 | 0 | 0 | 0 | 0 | 0 | 0 | 52 |
| Can J Hosp Pharm | 87 | 76 | 81 | 77 | 83 | 89 | 97 | 99 | 92 | 77 | 858 |
| Can Pharm J (Ott) | 0 | 0 | 0 | 114 | 106 | 64 | 65 | 61 | 71 | 71 | 552 |
| Consult Pharm | 77 | 79 | 67 | 70 | 66 | 87 | 93 | 89 | 114 | 87 | 829 |
| Curr Pharm Teach Learn | 0 | 0 | 0 | 0 | 0 | 0 | 4 | 22 | 164 | 222 | 412 |
| Hosp Pharm | 0 | 1 | 0 | 0 | 157 | 165 | 163 | 114 | 138 | 74 | 812 |
| Int J Clin Pharm | 0 | 0 | 119 | 113 | 151 | 166 | 161 | 186 | 152 | 189 | 1237 |
| Int J Pharm Compd | 77 | 77 | 22 | 75 | 75 | 79 | 73 | 75 | 69 | 63 | 685 |
| Int J Pharm Pract | 52 | 57 | 62 | 62 | 59 | 66 | 75 | 63 | 64 | 106 | 666 |
| J Am Pharm Assoc (2003) | 124 | 125 | 111 | 132 | 106 | 114 | 114 | 121 | 196 | 158 | 1301 |
| J Basic Clin Pharm | 9 | 38 | 27 | 26 | 19 | 28 | 22 | 26 | 0 | 0 | 195 |
| J Manag Care Spec Pharm | 0 | 0 | 0 | 0 | 0 | 101 | 114 | 152 | 151 | 146 | 664 |
| J Pain Palliat Care Pharmacother | 51 | 58 | 51 | 65 | 63 | 95 | 70 | 71 | 42 | 20 | 586 |
| J Pharm Bioallied Sci | 0 | 71 | 89 | 189 | 109 | 97 | 261 | 107 | 106 | 34 | 1063 |
| J Pharm Policy Pract | 0 | 0 | 0 | 0 | 11 | 19 | 31 | 39 | 39 | 30 | 169 |
| J Pharm Pract | 0 | 72 | 67 | 76 | 80 | 76 | 81 | 78 | 146 | 201 | 877 |
| J Pharmacol Pharmacother | 0 | 31 | 91 | 97 | 95 | 70 | 70 | 47 | 40 | 0 | 541 |
| J Res Pharm Pract | 0 | 0 | 0 | 17 | 32 | 31 | 38 | 54 | 45 | 35 | 252 |
| J Young Pharm | 0 | 84 | 56 | 48 | 44 | 0 | 0 | 0 | 0 | 0 | 232 |
| P T | 90 | 82 | 113 | 107 | 105 | 106 | 119 | 139 | 139 | 132 | 1132 |
| Pharm Hist | 11 | 11 | 10 | 7 | 13 | 7 | 2 | 7 | 0 | 0 | 68 |
| Pharm Hist (Lond) | 8 | 14 | 9 | 17 | 17 | 20 | 16 | 16 | 0 | 0 | 117 |
| Pharm Pat Anal | 0 | 0 | 0 | 66 | 72 | 58 | 37 | 47 | 38 | 31 | 349 |
| Pharm Pract (Granada) | 35 | 35 | 36 | 33 | 34 | 37 | 37 | 43 | 51 | 50 | 391 |
| Pharmacy (Basel) | 0 | 0 | 0 | 0 | 0 | 0 | 30 | 36 | 67 | 136 | 269 |
| Regul Toxicol Pharmacol | 123 | 150 | 145 | 174 | 140 | 220 | 260 | 281 | 249 | 274 | 2016 |
| Res Social Adm Pharm | 40 | 37 | 42 | 58 | 94 | 85 | 87 | 106 | 144 | 283 | 976 |
| Saudi Pharm J | 16 | 31 | 35 | 48 | 53 | 81 | 97 | 91 | 178 | 162 | 792 |
| Yakugaku Zasshi | 190 | 233 | 240 | 199 | 175 | 181 | 191 | 223 | 203 | 215 | 2050 |
| Yakushigaku Zasshi | 9 | 14 | 13 | 0 | 0 | 14 | 19 | 9 | 0 | 0 | 78 |
| **TOTAL** | 1637 | 2018 | 2103 | 2462 | 2560 | 2743 | 3024 | 3037 | 3312 | 3360 | 26256 |
| Journal names are abbreviated using the National Library of Medicine Title Abbreviation (https://www.ncbi.nlm.nih.gov/nlmcatalog/journals/) | | | | | | | | | | | |
