# Supplementary material for: Publication speed in pharmacy practice journals: A comparative analysis
Source: PLoS One. 2021 Jun 29;16(6):e0253713. doi: 10.1371/journal.pone.0253713 (PMC8241115; doi:10.1371/journal.pone.0253713)
Supplement: S3 Appendix — (DOCX) [file pone.0253713.s003.docx]

**Publication speed in pharmacy practice journals: a comparative analysis**

**Supporting information 3. Acceptance, lead, total, and indexing lag times for pharmacy practice journals (2009-2018)**

**Table S3. 1. Availability of completion dates of the publication process in the pharmacy practice journals between 2009 and 2018**

| Country of publication | N | Dates provided in metadata | | |
| --- | --- | --- | --- | --- |
|  |  | Submission | acceptance | online publication |
| Canada | 1,462 | 0 (0%) | 0 (0%) | 495 (33.9%) |
| France | 493 | 460 (99.3%) | 465 (94.3%) | 465 (94.3%) |
| India | 2,283 | 766 (33.6%) | 758 (33.2%) | 126 (5.5%) |
| Japan | 2,128 | 0 (0%) | 0 (0%) | 52 (2.4%) |
| Netherlands | 3,253 | 3,145 (96.7%) | 3,146 (96.7%) | 3,144 (96.6%) |
| Saudi Arabia | 792 | 711 (100.0%) | 710 (89.6%) | 771 (97.3%) |
| Spain | 391 | 383 (98.0%) | 380 (97.2%) | 304 (77.7%) |
| Switzerland | 269 | 268 (99.6%) | 268 (99.6%) | 269 (100%) |
| United Kingdom | 1,887 | 530 (28.1%) | 531 (28.1%) | 1,021 (54.1%) |
| United States | 13,298 | 2,637 (19.8%) | 2,706 (20.3%) | 2,822 (21.2%) |
| TOTAL | 26,256 | 8,900 (33.9%) | 8,964 (34.1%) | 9,469 (36.1%) |
|  |  |  |  |  |
| Comparison group | 23,888 | 11,676 (48.9%) | 12,127 (50.8%) | 15,589 (65.3%) |

**Table S3. 2. Acceptance lag times for pharmacy practice journals (2009-2018)**

| **Journal** | | **Acceptance lag** | | **Trend** | |
| --- | --- | --- | --- | --- | --- |
|  |  | **n** | **Median (IQR)** | **Spearman’s rho** | **p-value** |
| 01 | Am J Health Syst Pharm | - | - | - | - |
| 02 | Am J Pharm Educ | 900 | 75 (59 – 105) | -0.003 | 0.935 |
| 03 | Ann Pharm Fr | 460 | 76 (51 – 126) | 0.041 | 0.384 |
| 04 | Can J Clin Pharmacol | - | - | - | - |
| 05 | Can J Hosp Pharm | - | - | - | - |
| 06 | Can Pharm J (Ott) | - | - | - | - |
| 07 | Consult Pharm | - | - | - | - |
| 08 | Curr Pharm Teach Learn | 407 | 290 (230 – 349) | -0.085 | 0.087 |
| 09 | Hosp Pharm | - | - | - | - |
| 10 | Int J Clin Pharm | 1,161 | 171 (128 – 234) | **0.097** | 0.001 |
| 11 | Int J Pharm Compd | - | - | - | - |
| 12 | Int J Pharm Pract | 365 | 238 (182 – 304) | -0.116 | 0.027 |
| 13 | J Am Pharm Assoc (2003) | 355 | 147 (103 – 206) | 0.069 | 0.193 |
| 14 | J Basic Clin Pharm | 84 | 46 (27 – 80) | -0.032 | 0.770 |
| 15 | J Manag Care Spec Pharm | - | - | - | - |
| 16 | J Pain Palliat Care Pharmacother | - | - | - | - |
| 17 | J Pharm Bioallied Sci | 552 | 56 (9 – 91) | **-0.221** | <0.001 |
| 18 | J Pharm Policy Pract | 165 | 111 (69 – 160) | -0.105 | 0.178 |
| 18 | J Pharm Pract | - | - | - | - |
| 20 | J Pharmacol Pharmacother | 83 | 127 (87 – 174) | **0.265** | 0.015 |
| 21 | J Res Pharm Pract | - | - | - | - |
| 22 | J Young Pharm | 39 | 60 (45 – 98) | - | - |
| 23 | P T | - | - | - | - |
| 24 | Pharm Hist | - | - | - | - |
| 25 | Pharm Hist (Lond) | - | - | - | - |
| 26 | Pharm Pat Anal | - | - | - | - |
| 27 | Pharm Pract (Granada) | 378 | 135 (103 – 171) | 0.026 | 0.621 |
| 28 | Pharmacy (Basel) | 268 | 48 (32 – 71) | **-0.436** | <0.001 |
| 29 | Regul Toxicol Pharmacol | 1,984 | 98 (59 – 147) | **0.061** | 0.006 |
| 30 | Res Social Adm Pharm | 973 | 95 (1 – 135) | **-0.086** | 0.007 |
| 31 | Saudi Pharm J | 710 | 65 (40 – 104) | **0.196** | <0.001 |
| 32 | Yakugaku Zasshi | - | - | - | - |
| 33 | Yakushigaku Zasshi | - | - | - | - |
|  | TOTAL | 8,884 | 105 (57 – 173) | **0.163** | <0.001 |
|  | Comparison group | 11,166 | 97 (56 – 155) | **0.045** | <0.001 |

**Table S3. 3. Lead lag for pharmacy practice journals (2009-2018)**

| **Journal** | | **Lead lag** | | **Trend** | |
| --- | --- | --- | --- | --- | --- |
|  |  | **n** | **Median (IQR)** | **Spearman’s rho** | **p-value** |
| 01 | Am J Health Syst Pharm | - | - | - | - |
| 02 | Am J Pharm Educ | - | - | - | - |
| 03 | Ann Pharm Fr | 461 | 41 (33 – 54) | **-0.282** | <0.001 |
| 04 | Can J Clin Pharmacol | - | - | - | - |
| 05 | Can J Hosp Pharm | - | - | - | - |
| 06 | Can Pharm J (Ott) | - | - | - | - |
| 07 | Consult Pharm | - | - | - | - |
| 08 | Curr Pharm Teach Learn | 383 | 35 (21 – 66) | **-0.658** | <0.001 |
| 09 | Hosp Pharm | - | - | - | - |
| 10 | Int J Clin Pharm | 1,142 | 16 (11 – 24) | **-0.208** | <0.001 |
| 11 | Int J Pharm Compd | - | - | - | - |
| 12 | Int J Pharm Pract | 360 | 50 (39 – 66) | **-0.225** | <0.001 |
| 13 | J Am Pharm Assoc (2003) | 290 | 48 (39 – 63) | **-0.494** | <0.001 |
| 14 | J Basic Clin Pharm | 83 | 30 (15 – 66) | -0.042 | 0.706 |
| 15 | J Manag Care Spec Pharm | - | - | - | - |
| 16 | J Pain Palliat Care Pharmacother | - | - | - | - |
| 17 | J Pharm Bioallied Sci | - | - | - | - |
| 18 | J Pharm Policy Pract | 165 | 22 (13 – 36) | 0.024 | 0.764 |
| 18 | J Pharm Pract | - | - | - | - |
| 20 | J Pharmacol Pharmacother | - | - | - | - |
| 21 | J Res Pharm Pract | - | - | - | - |
| 22 | J Young Pharm | 39 | 30 (25 – 47) | - | - |
| 23 | P T | - | - | - | - |
| 24 | Pharm Hist | - | - | - | - |
| 25 | Pharm Hist (Lond) | - | - | - | - |
| 26 | Pharm Pat Anal | - | - | - | - |
| 27 | Pharm Pract (Granada) | 297 | 33 (16 – 57) | **-0.294** | <0.001 |
| 28 | Pharmacy (Basel) | 268 | 5 (3 – 7) | **-0.353** | <0.001 |
| 29 | Regul Toxicol Pharmacol | 1,980 | 6 (3-10) | **-0.519** | <0.001 |
| 30 | Res Social Adm Pharm | 927 | 12 (4 – 43) | **-0.818** | <0.001 |
| 31 | Saudi Pharm J | 705 | 8 (4 – 13) | **-0.681** | <0.001 |
| 32 | Yakugaku Zasshi | - | - | - | - |
| 33 | Yakushigaku Zasshi | - | - | - | - |
|  | TOTAL | 7,100 | 13 (6 – 35) | **-0.230** | <0.001 |
|  | Comparison group | 10,574 | 23 (9 – 45) | **-0.127** | <0.001 |

**Table S3. 4. Total lag times for pharmacy practice journals (2009-2018)**

| **Journal** | | **Total lag** | | **Trend** | |
| --- | --- | --- | --- | --- | --- |
|  |  | **n** | **Median (IQR)** | **Spearman’s rho** | **p-value** |
| 01 | Am J Health Syst Pharm | - | - | - | - |
| 02 | Am J Pharm Educ | - | - | - | - |
| 03 | Ann Pharm Fr | 456 | 120 (92 – 176) | -0.042 | 0.367 |
| 04 | Can J Clin Pharmacol | - | - | - | - |
| 05 | Can J Hosp Pharm | - | - | - | - |
| 06 | Can Pharm J (Ott) | - | - | - | - |
| 07 | Consult Pharm | - | - | - | - |
| 08 | Curr Pharm Teach Learn | 383 | 332 (268 – 399) | **-0.309** | <0.001 |
| 09 | Hosp Pharm | - | - | - | - |
| 10 | Int J Clin Pharm | 1,142 | 192 (147 – 261) | **0.062** | 0.036 |
| 11 | Int J Pharm Compd | - | - | - | - |
| 12 | Int J Pharm Pract | 359 | 295 (243 – 366) | **-0.170** | 0.001 |
| 13 | J Am Pharm Assoc (2003) | 284 | 199 (153 – 255) | **-0.132** | 0.026 |
| 14 | J Basic Clin Pharm | 83 | 88 (51 – 154) | -0.056 | 0.613 |
| 15 | J Manag Care Spec Pharm | - | - | - | - |
| 16 | J Pain Palliat Care Pharmacother | - | - | - | - |
| 17 | J Pharm Bioallied Sci | - | - | - | - |
| 18 | J Pharm Policy Pract | 165 | 138 (97 – 188) | -0.081 | 0.302 |
| 18 | J Pharm Pract | - | - | - | - |
| 20 | J Pharmacol Pharmacother | - | - | - | - |
| 21 | J Res Pharm Pract | - | - | - | - |
| 22 | J Young Pharm | 39 | 94 (82 – 141) | - | - |
| 23 | P T | - | - | - | - |
| 24 | Pharm Hist | - | - | - | - |
| 25 | Pharm Hist (Lond) | - | - | - | - |
| 26 | Pharm Pat Anal | - | - | - | - |
| 27 | Pharm Pract (Granada) | 296 | 179 (139 – 218) | **-0.184** | 0.002 |
| 28 | Pharmacy (Basel) | 269 | 54 (36 – 78) | **-0.465** | <0.001 |
| 29 | Regul Toxicol Pharmacol | 1,979 | 106 (67 – 155) | 0.028 | 0.221 |
| 30 | Res Social Adm Pharm | 927 | 119 (29 – 179) | **-0.388** | <0.001 |
| 31 | Saudi Pharm J | 705 | 76 (51 – 18) | 0.055 | 0.145 |
| 32 | Yakugaku Zasshi | - | - | - | - |
| 33 | Yakushigaku Zasshi | - | - | - | - |
|  | TOTAL | 7,086 | 138 (79 – 217) | **0.041** | 0.001 |
|  | Comparison group | 9,914 | 131 (82 – 197) | -0.002 | 0.831 |

**Table S3. 5. Indexing lag for pharmacy practice journals (2009-2018)**

| **Journal** | | **Indexing lag** | | **Trend** | |
| --- | --- | --- | --- | --- | --- |
|  |  | **n** | **Median (IQR)** | **Spearman’s rho** | **p-value** |
| 01 | Am J Health Syst Pharm | 200 | 2 (2 – 2) | 0.035 | 0.626 |
| 02 | Am J Pharm Educ | - | - | - | - |
| 03 | Ann Pharm Fr | 463 | 26 (5 – 55) | **-0.547** | <0.001 |
| 04 | Can J Clin Pharmacol | 52 | 3 (2 – 4) | 0.187 | 0.184 |
| 05 | Can J Hosp Pharm | 249 | 13 (8 – 13) | **-0.579** | <0.001 |
| 06 | Can Pharm J (Ott) | 194 | 51(37 – 81) | -0.002 | 0.973 |
| 07 | Consult Pharm | - | - | - | - |
| 08 | Curr Pharm Teach Learn | 385 | 144 (108 – 196) | **-0.484** | <0.001 |
| 09 | Hosp Pharm | 246 | 135 (102 – 184) | **-0.255** | <0.001 |
| 10 | Int J Clin Pharm | 1,143 | 2 (1 – 2) | **0.188** | <0.001 |
| 11 | Int J Pharm Compd | - | - | - | - |
| 12 | Int J Pharm Pract | 456 | 2 (1 – 85) | **-0.708** | <0.001 |
| 13 | J Am Pharm Assoc (2003) | 304 | 5 (4 – 5) | **-0.184** | 0.001 |
| 14 | J Basic Clin Pharm | - | - | - | - |
| 15 | J Manag Care Spec Pharm | 55 | 27 (1 – 81) | **-0.574** | <0.001 |
| 16 | J Pain Palliat Care Pharmacother | 279 | 1 (1 – 2) | **-0.352** | <0.001 |
| 17 | J Pharm Bioallied Sci | - | - | - | - |
| 18 | J Pharm Policy Pract | 148 | 7 (4 – 12) | **-0.176** | 0.034 |
| 18 | J Pharm Pract | 702 | 3 (2 – 59) | **-0.264** | <0.001 |
| 20 | J Pharmacol Pharmacother | - | - | - | - |
| 21 | J Res Pharm Pract | - | - | - | - |
| 22 | J Young Pharm | 42 | 100 (66 – 167) | - | - |
| 23 | P T | - | - | - | - |
| 24 | Pharm Hist | - | - | - | - |
| 25 | Pharm Hist (Lond) | - | - | - | - |
| 26 | Pharm Pat Anal | 118 | 1 (1 – 1) | -0.046 | 0.624 |
| 27 | Pharm Pract (Granada) | 217 | 30 (19 – 55) | -0.111 | 0.104 |
| 28 | Pharmacy (Basel) | 269 | 3 (1 – 28) | 0.115 | 0.059 |
| 29 | Regul Toxicol Pharmacol | 1,941 | 5 (4 – 6) | **-0.171** | <0.001 |
| 30 | Res Social Adm Pharm | 921 | 17 (7 – 33) | **-0.103** | 0.002 |
| 31 | Saudi Pharm J | 755 | 253 (126 – 372) | **-0.247** | <0.001 |
| 32 | Yakugaku Zasshi | 52 | 2 (1 – 4) | -0.083 | 0.559 |
| 33 | Yakushigaku Zasshi | - | - | - | - |
|  | TOTAL | 9,189 | 5 (2 – 46) | 0.008 | 0.446 |
|  | Comparison group | 14,297 | 4 (2 – 12) | **-0.049** | <0.001 |
