# Supplementary material for: Publication speed in pharmacy practice journals: A comparative analysis
Source: PLoS One. 2021 Jun 29;16(6):e0253713. doi: 10.1371/journal.pone.0253713 (PMC8241115; doi:10.1371/journal.pone.0253713)
Supplement: S4 Appendix — (DOCX) [file pone.0253713.s004.docx]

**Publication speed in pharmacy practice journals: a comparative analysis**

**Supporting information 4. Acceptance, lead, total, and indexing lag times trends (2009-2018)**

**Table S4. 1. Acceptance lag time trend between 2009 and 2018**

| **Year** | **Comparison group** | | **Pharmacy practice** | |
| --- | --- | --- | --- | --- |
|  | **n** | **Median (IQR)** | **n** | **Median (IQR)** |
| 2009 | 386 | 90 (43 – 145) | 377 | 88 (61 – 137) |
| 2010 | 578 | 92 (53 – 153) | 521 | 82 (55 – 126) |
| 2011 | 640 | 91 (49 – 144) | 647 | 93 (61 – 146) |
| 2012 | 1,381 | 88 (52 – 147) | 749 | 85 (56 – 139) |
| 2013 | 1,160 | 98 (58 – 155) | 840 | 98 (58 – 175) |
| 2014 | 663 | 100 (58 – 162) | 968 | 91 (52 – 163) |
| 2015 | 672 | 98 (60 – 154) | 1,012 | 98 (50 – 157) |
| 2016 | 1,103 | 100 (61 – 151) | 928 | 105 (39 – 169) |
| 2017 | 1,958 | 101 (57 – 161) | 1,252 | 134 (78 – 219) |
| 2018 | 2,605 | 100 (57 – 161) | 1,590 | 134 (69 – 215) |

**Table S4. 2. Lead lag time trend between 2009 and 2018**

| **Year** | **Comparison group** | | **Pharmacy practice** | |
| --- | --- | --- | --- | --- |
|  | **n** | **Median (IQR)** | **n** | **Median (IQR)** |
| 2009 | 338 | 21 (8 – 43) | 238 | 22 (8 – 68) |
| 2010 | 510 | 23 (9 – 42) | 285 | 15 (6 – 49) |
| 2011 | 575 | 27 (9 – 55) | 426 | 20 (10 – 51) |
| 2012 | 1,351 | 29 (12 – 50) | 469 | 14 (9 – 37) |
| 2013 | 1,149 | 30 (15 – 49) | 579 | 23 (11 – 47) |
| 2014 | 645 | 26 (12 – 50) | 663 | 15 (9 – 32) |
| 2015 | 670 | 26 (11 – 53) | 783 | 11 (7 – 23) |
| 2016 | 1,046 | 22 (9 – 42) | 868 | 10 (5 – 23) |
| 2017 | 1,836 | 22 (9 – 43) | 1,216 | 14 (4 – 43) |
| 2018 | 2,439 | 17 (6 – 39) | 1,573 | 9 (2 – 28) |

**Table S4. 3. Total lag time trend between 2009 and 2018**

| **Year** | **Comparison group** | | **Pharmacy practice** | |
| --- | --- | --- | --- | --- |
|  | **n** | **Median (IQR)** | **n** | **Median (IQR)** |
| 2009 | 316 | 117 (62 – 189) | 233 | 141 (87 – 203) |
| 2010 | 491 | 131 (80 – 200) | 285 | 127 (88 – 185) |
| 2011 | 528 | 133 (84 – 190) | 426 | 142 (93 – 210) |
| 2012 | 1,232 | 128 (80 – 189) | 469 | 135 (83 – 198) |
| 2013 | 1,046 | 135 (87 – 201) | 578 | 151 (95 – 229) |
| 2014 | 598 | 139 (87 – 211) | 663 | 130 (71 – 203) |
| 2015 | 618 | 134 (86 – 206) | 782 | 120 (70 – 185) |
| 2016 | 993 | 130 (86 – 192) | 862 | 113 (47 – 189) |
| 2017 | 1,747 | 132 (82 – 198) | 1216 | 161 (92 – 257) |
| 2018 | 2,330 | 127 (79 – 196) | 1572 | 149 (78 – 241) |

**Table S4. 4. Indexing lag time trend between 2009 and 2018**

| **Year** | **Comparison group** | | **Pharmacy practice** | |
| --- | --- | --- | --- | --- |
|  | **n** | **Median (IQR)** | **n** | **Median (IQR)** |
| 2009 | 566 | 4 (2 – 18) | 247 | 13 (4 – 44) |
| 2010 | 778 | 4 (2 – 7) | 300 | 16 (4 – 180) |
| 2011 | 977 | 3 (2 – 12) | 450 | 13 (3 – 72) |
| 2012 | 2081 | 4 (2 – 10) | 545 | 7 (2 – 37) |
| 2013 | 1683 | 4 (2 – 7) | 700 | 5 (2 – 29) |
| 2014 | 866 | 4 (2 – 14) | 779 | 4 (2 – 19) |
| 2015 | 862 | 4 (1 – 20) | 979 | 5 (2 – 38) |
| 2016 | 1,314 | 4 (2 – 23) | 1,263 | 5 (2 – 29) |
| 2017 | 2,335 | 3 (1 – 17) | 1,769 | 8 (4 – 100) |
| 2018 | 2,804 | 3 (2 – 8) | 2,157 | 6 (2 – 54) |
