# Supplementary material for: Publication speed in pharmacy practice journals: A comparative analysis
Source: PLoS One. 2021 Jun 29;16(6):e0253713. doi: 10.1371/journal.pone.0253713 (PMC8241115; doi:10.1371/journal.pone.0253713)
Supplement: S5 Appendix — (DOCX) [file pone.0253713.s005.docx]

**Publication speed in pharmacy practice journals: a comparative analysis**

**Supporting information 4. Pharmacy practice journals’ data (violin plots with lines representing percentile25, median, and percentil75)**

| **Am J Health Syst Pharm** | **2009** | **2010** | **2011** | **2012** | **2013** | **2014** | **2015** | **2016** | **2017** | **2018** | **TOTAL** |
| --- | --- | --- | --- | --- | --- | --- | --- | --- | --- | --- | --- |
| Articles in PubMed | 373 | 380 | 360 | 346 | 320 | 344 | 375 | 382 | 371 | 343 | 3594 |
| Submission date; n(%) | 0 | 0 | 0 | 0 | 0 | 0 | 0 | 0 | 0 | 0 | 0 |
| Acceptance date; n(%) | 0 | 0 | 0 | 0 | 0 | 0 | 0 | 0 | 0 | 0 | 0 |
| Online publication date; n(%) | 0 | 0 | 0 | 0 | 0 | 0 | 3 (0.8) | 39 (10.2) | 69 (18.6) | 89 (25.9) | 200 (5.6) |
|  |  |  |  |  |  |  |  |  |  |  |  |
| Acceptance lag; median(IQR) | NR | NR | NR | NR | NR | NR | NR | NR | NR | NR | NR |
| Lead lag; median(IQR) | NR | NR | NR | NR | NR | NR | NR | NR | NR | NR | NR |
| Total lag; median(IQR) | NR | NR | NR | NR | NR | NR | NR | NR | NR | NR | NR |
| Indexing lag; median(IQR) | NR | NR | NR | NR | NR | NR | 2 (2-2) | 2 (2-2) | 2 (2-2) | 2 (2-2) | 2 (2-2) |

| 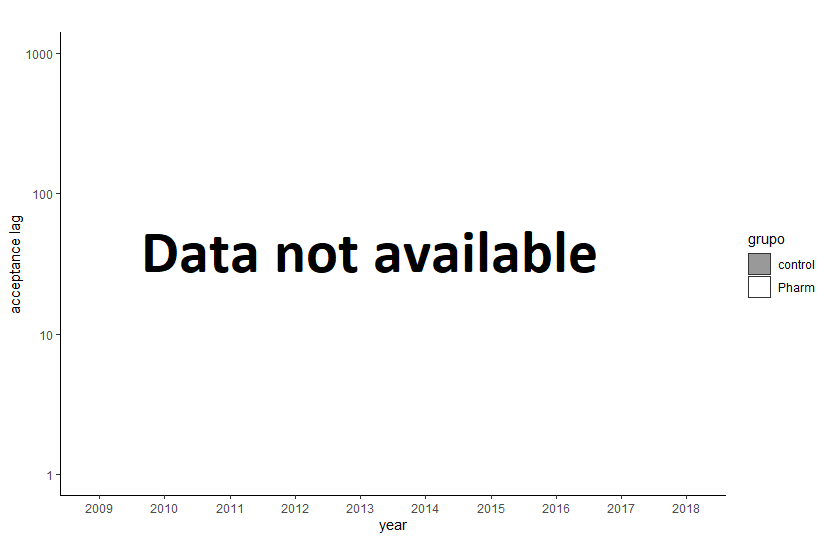 | 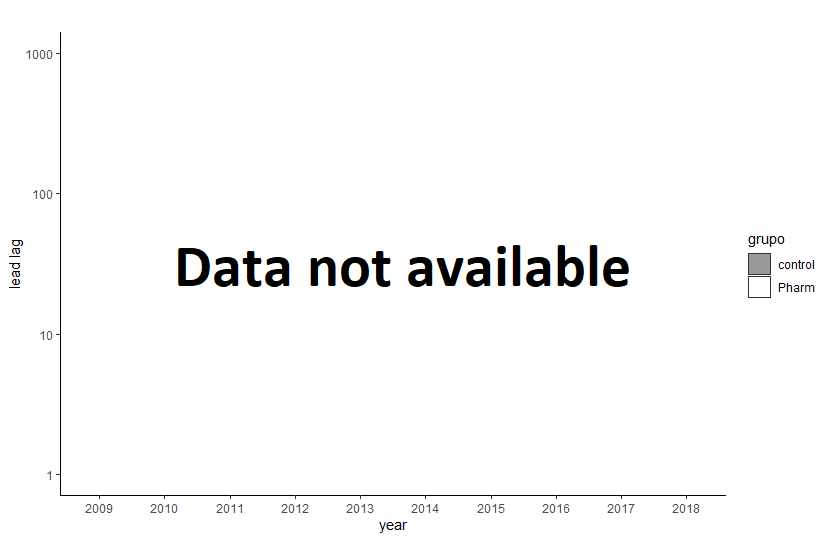 |
| --- | --- |
| 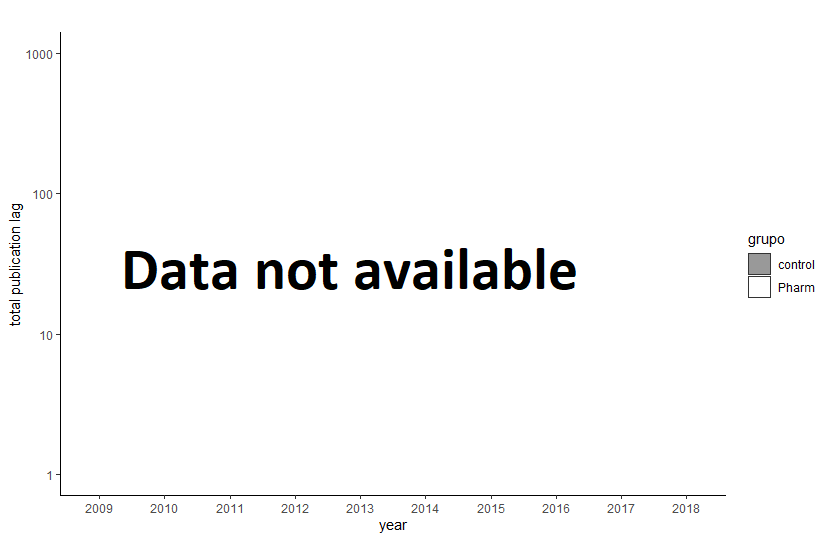 | 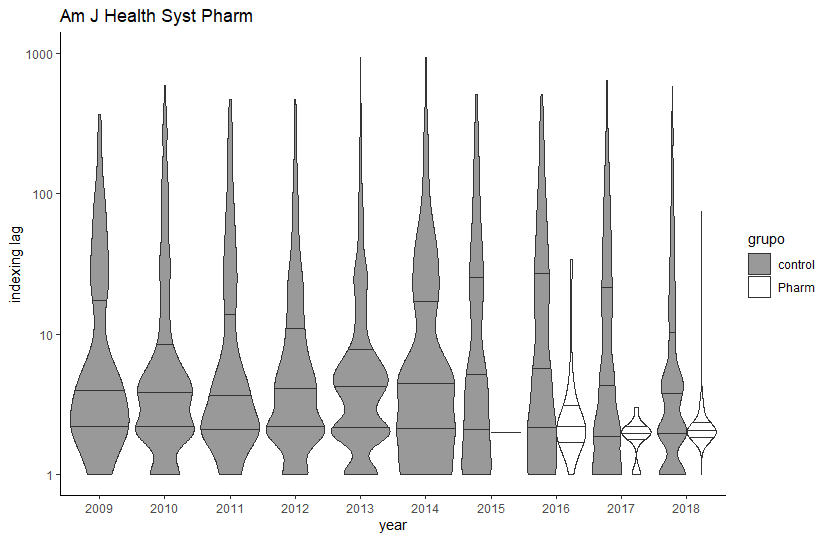 |

| **Am J Pharm Educ** | **2009** | **2010** | **2011** | **2012** | **2013** | **2014** | **2015** | **2016** | **2017** | **2018** | **TOTAL** |
| --- | --- | --- | --- | --- | --- | --- | --- | --- | --- | --- | --- |
| Articles in PubMed | 167 | 210 | 211 | 200 | 232 | 195 | 171 | 204 | 193 | 165 | 1948 |
| Submission date; n(%) | 109 (65.3) | 140 (66.7) | 146 (69.2) | 148 (74.0) | 153 (65.9) | 145 (74.4) | 62 (36.3) | 0 | 0 | 0 | 903 (46.4) |
| Acceptance date; n(%) | 109 (65.3) | 140 (66.7) | 146 (69.2) | 148 (74.0) | 151 (65.1) | 148 (75.9) | 62 (36.3) | 0 | 0 | 0 | 0 |
| Online publication date; n(%) | 0 | 0 | 0 | 0 | 0 | 0 | 0 | 0 | 0 | 0 | 0 |
|  |  |  |  |  |  |  |  |  |  |  |  |
| Acceptance lag; median(IQR) | 83 (64-132) | 77 (57-105) | 75 (56-103) | 73 (52-93) | 69 (59-103) | 72 (57-93) | 105 (74-142) | NR | NR | NR | 75 (58-105) |
| Lead lag; median(IQR) | NR | NR | NR | NR | NR | NR | NR | NR | NR | NR | NR |
| Total lag; median(IQR) | NR | NR | NR | NR | NR | NR | NR | NR | NR | NR | NR |
| Indexing lag; median(IQR) | NR | NR | NR | NR | NR | NR | NR | NR | NR | NR | NR |

| 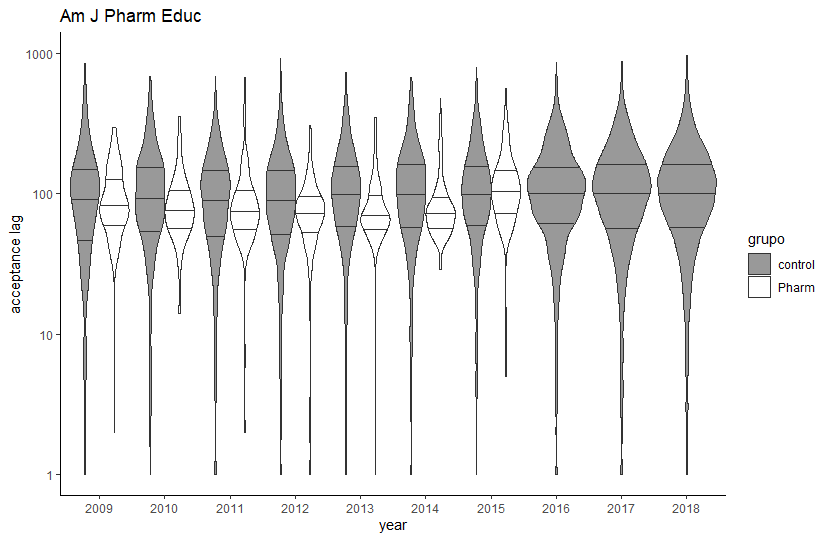 | 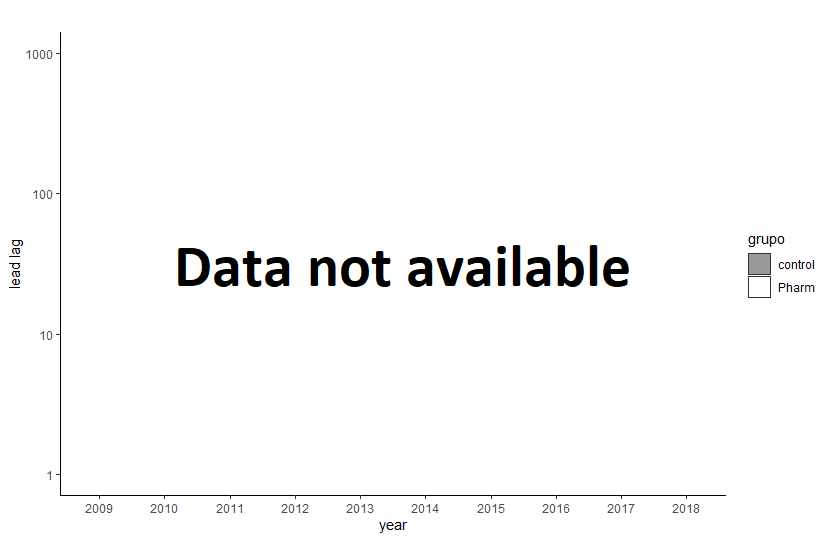 |
| --- | --- |
| 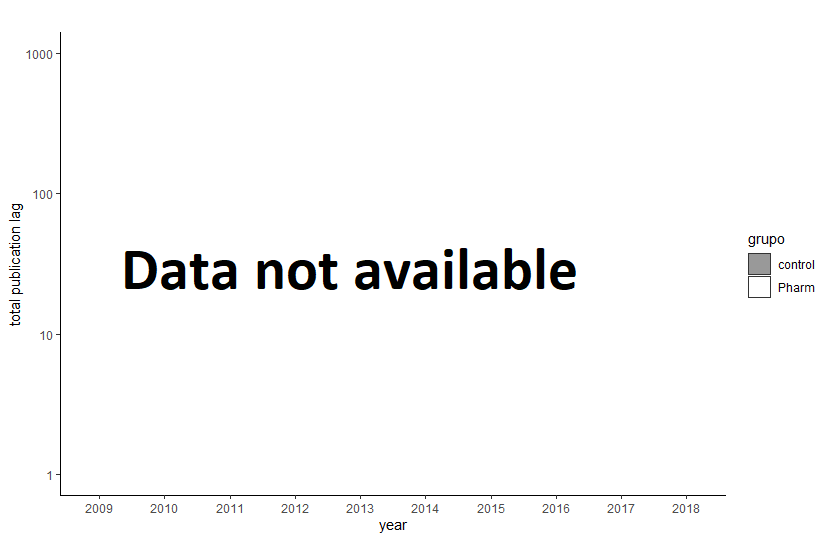 | 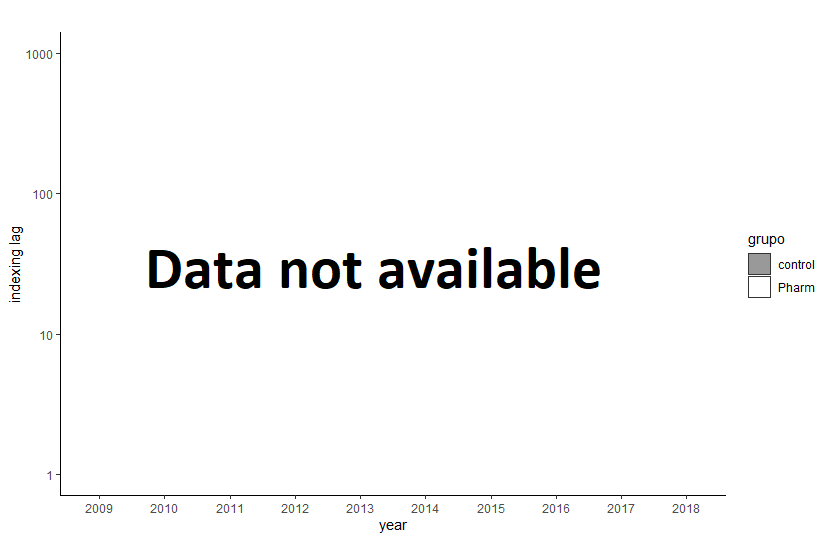 |

| **Ann Pharm Fr** | **2009** | **2010** | **2011** | **2012** | **2013** | **2014** | **2015** | **2016** | **2017** | **2018** | **TOTAL** |
| --- | --- | --- | --- | --- | --- | --- | --- | --- | --- | --- | --- |
| Articles in PubMed | 58 | 40 | 46 | 46 | 49 | 48 | 51 | 49 | 50 | 56 | 493 |
| Submission date; n(%) | 53 (91.4) | 37 (92.5) | 42 (91.3) | 40 (87.0) | 41 (91.8) | 46 (95.8) | 48 (94.1) | 48 (98.0) | 49 (98.0) | 52 (92.9) | 460 (93.3) |
| Acceptance date; n(%) | 58 (100) | 37 (92.5) | 42 (91.3) | 40 (87.0) | 45 (91.8) | 46 (95.8) | 48 (94.1) | 48 (98.0) | 49 (98.0) | 52 (92.9) | 465 (94.3) |
| Online publication date; n(%) | 58 (100) | 35 (87.5) | 42 (91.3) | 40 (87.0) | 46 (93.9) | 46 (95.8) | 49 (96.1) | 48 (98.0) | 49 (98.0) | 52 (92.9) | 465 (94.3) |
|  |  |  |  |  |  |  |  |  |  |  |  |
| Acceptance lag | 85 (53-136) | 82 (58-123) | 78 (53-116) | 94 (70-129) | 66 (34-92) | 65 (45-93) | 73 (53-103) | 78 (47-138) | 90 (50-141) | 77 (58-154) | 76 (51-126) |
| Lead lag | 51 (42-64 | 50 (41-72) | 50 (39-66) | 40 (33-50) | 36 (34-46) | 36 (31-49) | 34 (30-42) | 37 (31-49) | 41 (33-49) | 40 (30-55) | 41 (33-54) |
| Total lag | 147 (105-187 | 152 (97-184) | 131 (94-181) | 135 (108-168) | 109 (72-137) | 100 (85-147) | 108 (86-141) | 120 (87-187) | 122 (93-195) | 118 (99-203) | 120 (92-176) |
| Indexing lag | 37 (22-52) | 40 (31-76) | 35 (25-47) | 48 (28-67) | 30 (26-68) | 100 (69-136) | 5 (5-107) | 6 (5-12) | 5 (4-5) | 5 (4-5) | 26 (5-55) |

| 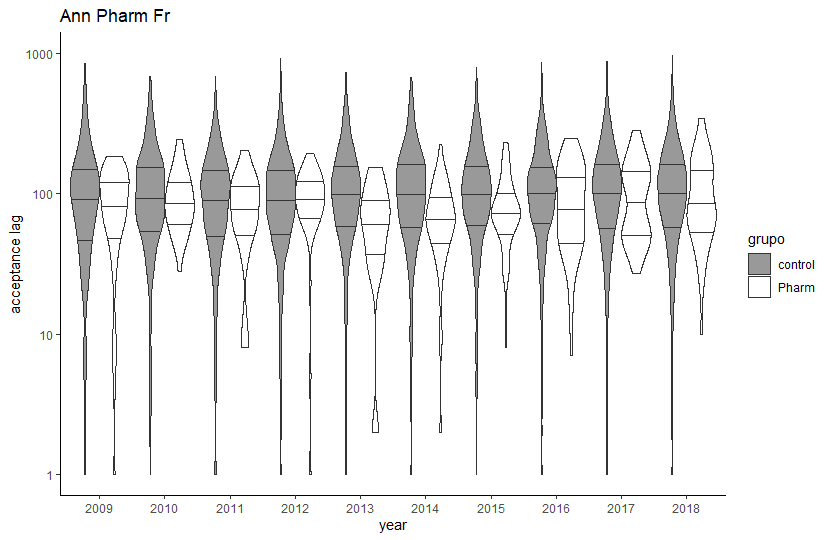 | 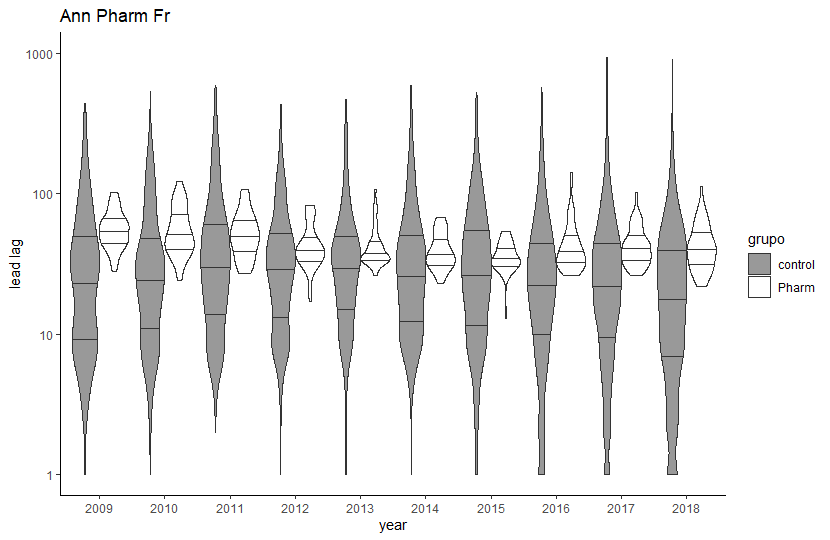 |
| --- | --- |
| 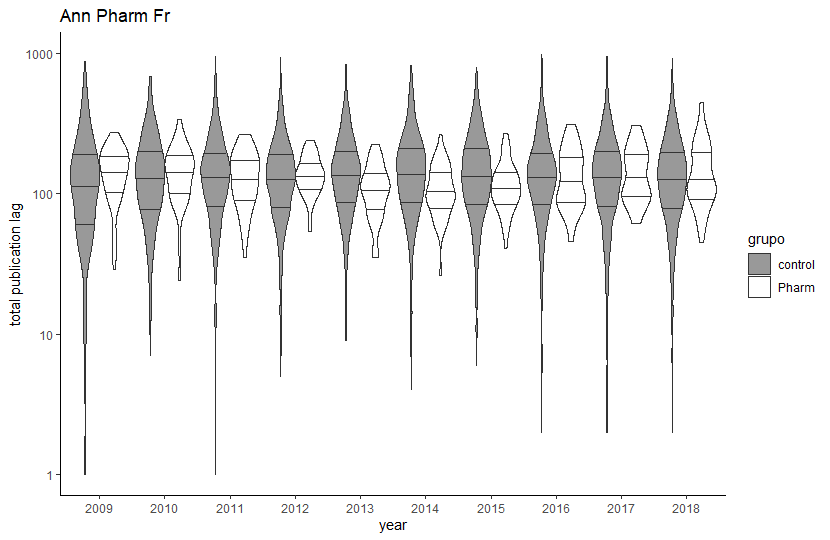 | 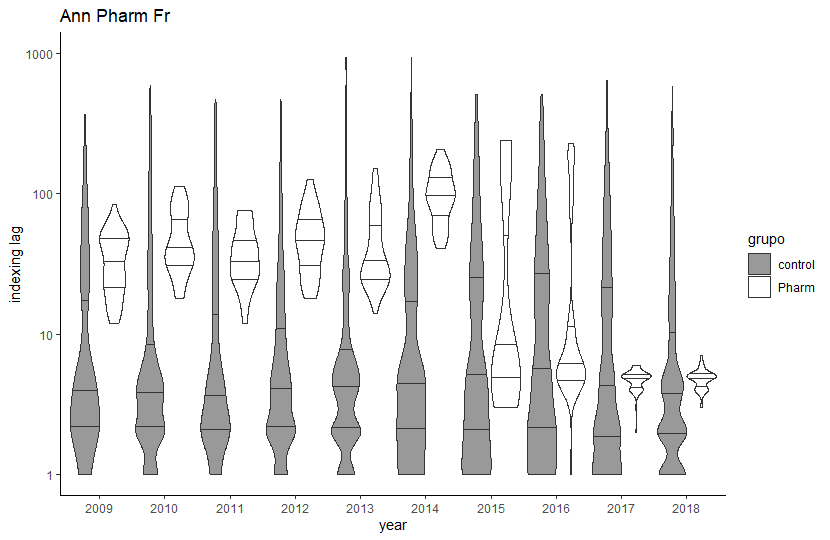 |

| **Can J Clin Pharmacol** | **2009** | **2010** | **2011** | **2012** | **2013** | **2014** | **2015** | **2016** | **2017** | **2018** | **TOTAL** |
| --- | --- | --- | --- | --- | --- | --- | --- | --- | --- | --- | --- |
| Articles in PubMed | 40 | 12 | 0 | 0 | 0 | 0 | 0 | 0 | 0 | 0 | 52 |
| Submission date; n(%) | 0 | 0 | - | - | - | - | - | - | - | - | 0 |
| Acceptance date; n(%) | 0 | 0 | - | - | - | - | - | - | - | - | 0 |
| Online publication date; n(%) | 0 | 0 | - | - | - | - | - | - | - | - | 0 |
|  |  |  |  |  |  |  |  |  |  |  |  |
| Acceptance lag | NR | NR | - | - | - | - | - | - | - | - | NR |
| Lead lag | NR | NR | - | - | - | - | - | - | - | - | NR |
| Total lag | NR | NR | - | - | - | - | - | - | - | - | NR |
| Indexing lag | 2 (2-4) | 3 (3-8) | - | - | - | - | - | - | - | - | 3 (2-4) |

| 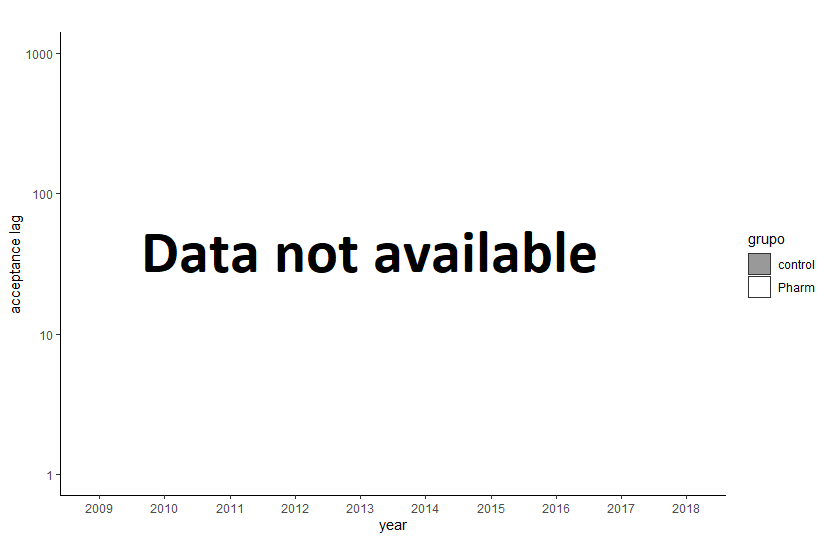 | 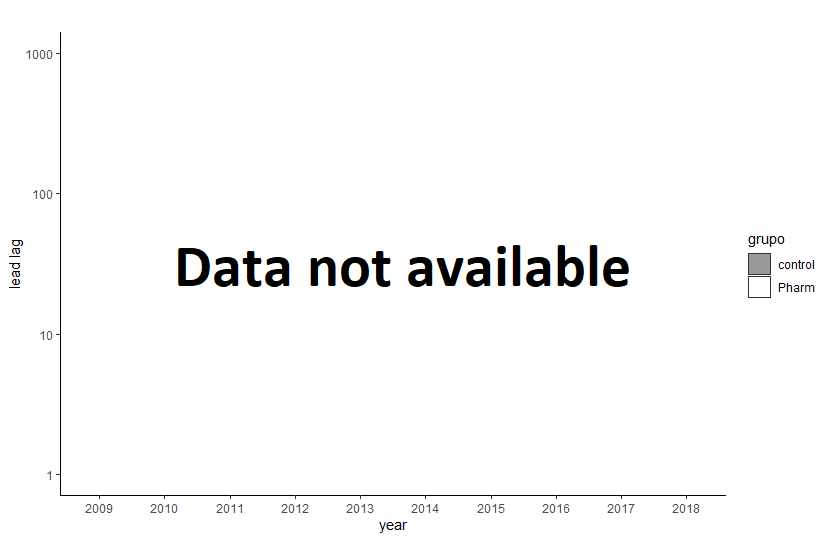 |
| --- | --- |
| 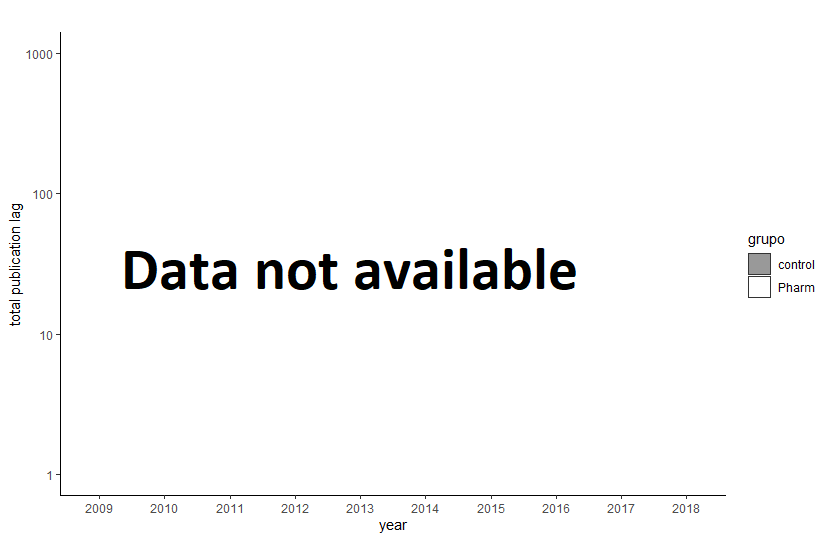 | 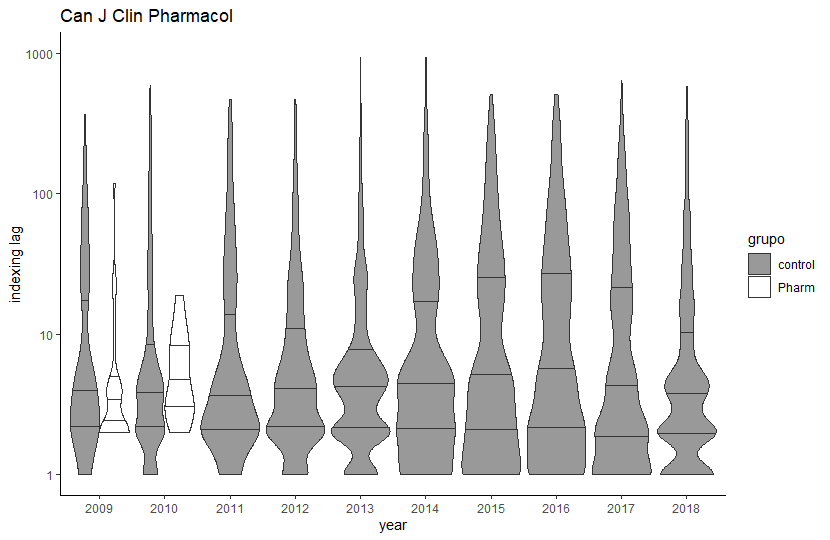 |

| **Can J Hosp Pharm** | **2009** | **2010** | **2011** | **2012** | **2013** | **2014** | **2015** | **2016** | **2017** | **2018** | **TOTAL** |
| --- | --- | --- | --- | --- | --- | --- | --- | --- | --- | --- | --- |
| Articles in PubMed | 87 | 76 | 81 | 77 | 83 | 89 | 97 | 99 | 92 | 77 | 858 |
| Submission date; n(%) | 0 | 0 | 0 | 0 | 0 | 0 | 0 | 0 | 0 | 0 | 0 |
| Acceptance date; n(%) | 0 | 0 | 0 | 0 | 0 | 0 | 0 | 0 | 0 | 0 | 0 |
| Online publication date; n(%) | 0 | 0 | 0 | 0 | 0 | 0 | 0 | 83 (83.8) | 90 (97.8) | 76 (98.7) | 249 (29.0) |
|  |  |  |  |  |  |  |  |  |  |  |  |
| Acceptance lag | NR | NR | NR | NR | NR | NR | NR | NR | NR | NR | NR |
| Lead lag | NR | NR | NR | NR | NR | NR | NR | NR | NR | NR | NR |
| Total lag | NR | NR | NR | NR | NR | NR | NR | NR | NR | NR | NR |
| Indexing lag | NR | NR | NR | NR | NR | NR | NR | 13 (13-14) | 13 (8-15) | 8 (7-10) | 13 (8-13) |

| 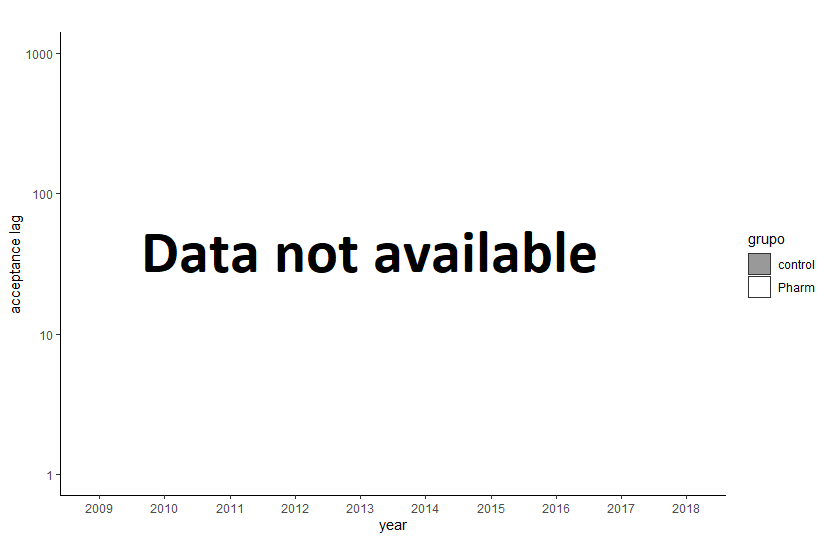 | 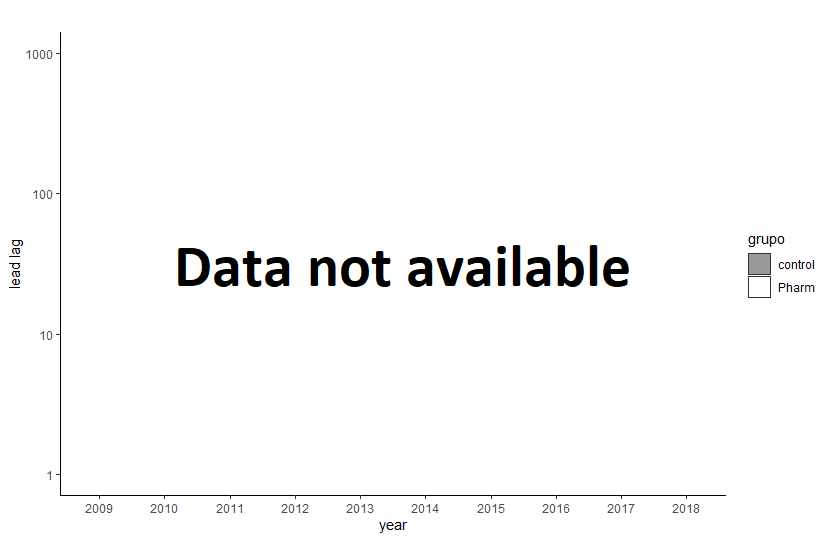 |
| --- | --- |
| 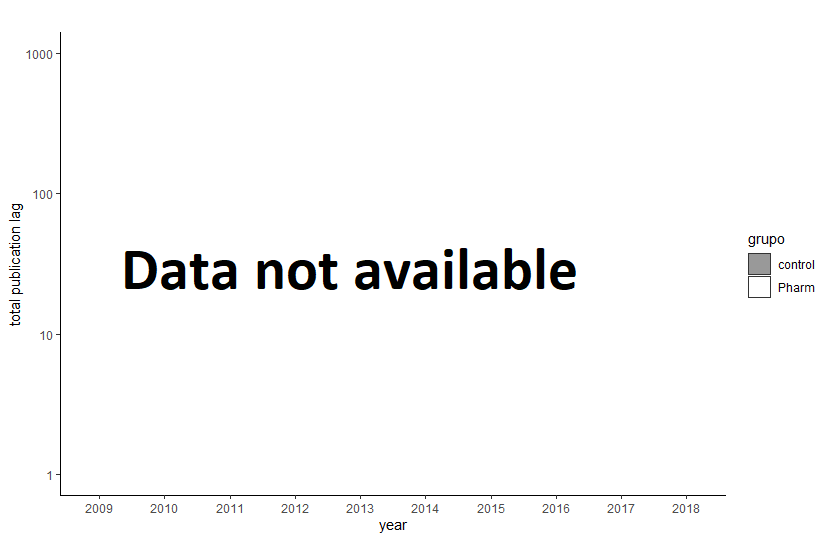 | 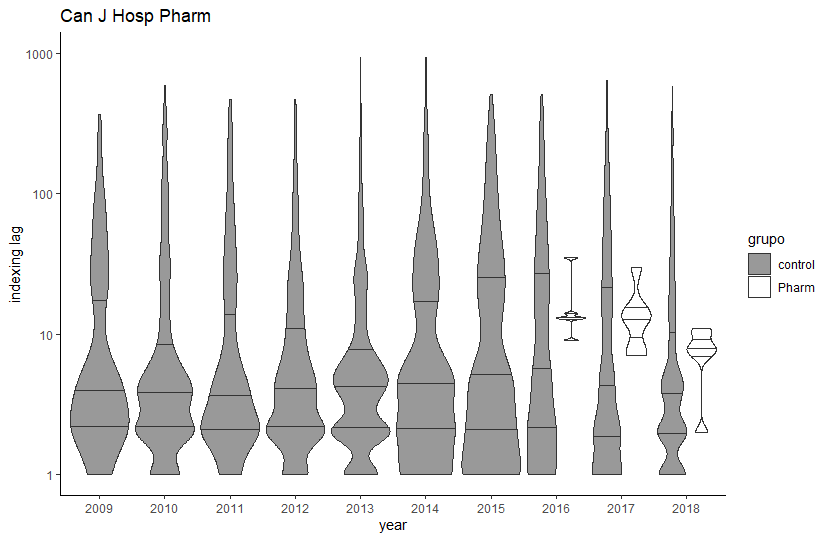 |

| **Can Pharm J (Ott)** | **2009** | **2010** | **2011** | **2012** | **2013** | **2014** | **2015** | **2016** | **2017** | **2018** | **TOTAL** |
| --- | --- | --- | --- | --- | --- | --- | --- | --- | --- | --- | --- |
| Articles in PubMed | 0 | 0 | 0 | 114 | 106 | 64 | 65 | 61 | 71 | 71 | 552 |
| Submission date; n(%) | - | - | - | 0 | 0 | 0 | 0 | 0 | 0 | 0 | 0 |
| Acceptance date; n(%) | - | - | - | 0 | 0 | 0 | 0 | 0 | 0 | 0 | 0 |
| Online publication date; n(%) | - | - | - | 0 | 0 | 0 | 0 | 52 (85.2) | 71 (100) | 71 (100) | 194 (35.1) |
|  |  |  |  |  |  |  |  |  |  |  |  |
| Acceptance lag | - | - | - | NR | NR | NR | NR | NR | NR | NR | NR |
| Lead lag | - | - | - | NR | NR | NR | NR | NR | NR | NR | NR |
| Total lag | - | - | - | NR | NR | NR | NR | NR | NR | NR | NR |
| Indexing lag | - | - | - | NR | NR | NR | NR | 49 (36-70) | 57 (38-98) | 52 (34-69) | 51 (37-81) |

| 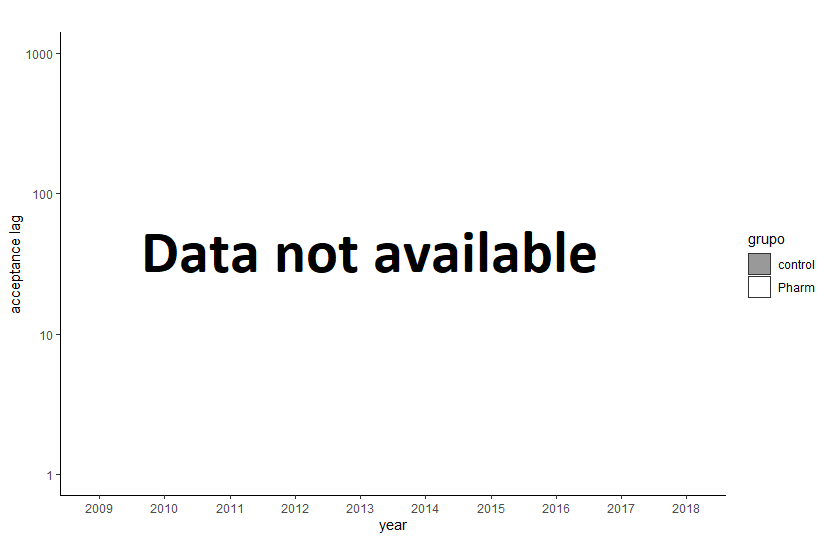 | 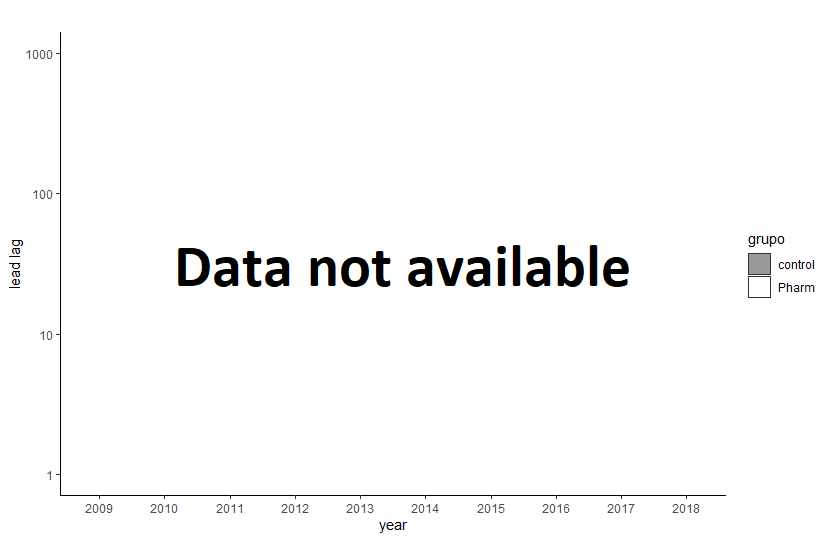 |
| --- | --- |
| 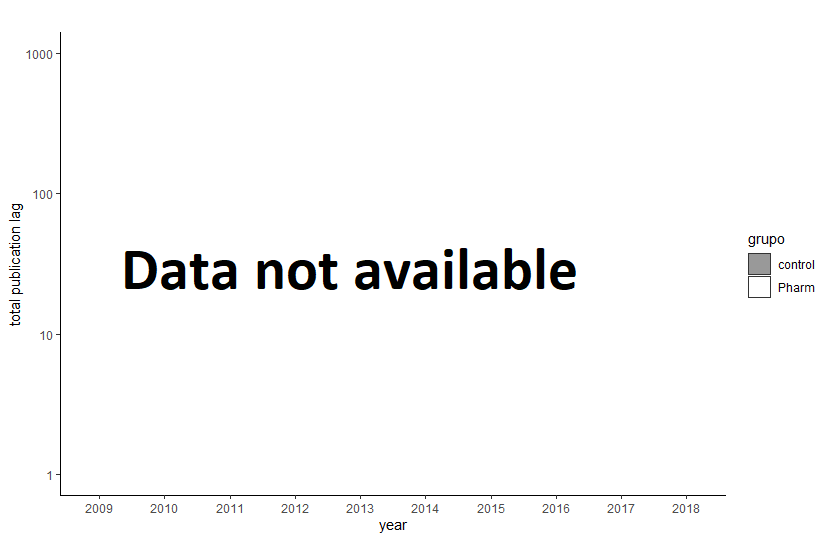 | 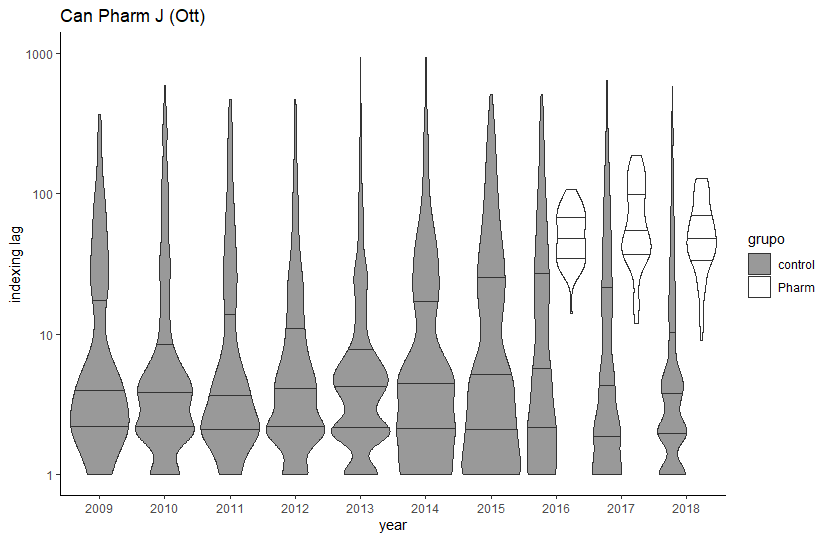 |

| **Consult Pharm** | **2009** | **2010** | **2011** | **2012** | **2013** | **2014** | **2015** | **2016** | **2017** | **2018** | **TOTAL** |
| --- | --- | --- | --- | --- | --- | --- | --- | --- | --- | --- | --- |
| Articles in PubMed | 77 | 79 | 67 | 70 | 66 | 87 | 93 | 89 | 114 | 87 | 829 |
| Submission date; n(%) | 0 | 0 | 0 | 0 | 0 | 0 | 0 | 0 | 0 | 0 | 0 |
| Acceptance date; n(%) | 0 | 0 | 0 | 0 | 0 | 0 | 0 | 0 | 0 | 0 | 0 |
| Online publication date; n(%) | 0 | 0 | 0 | 0 | 0 | 0 | 0 | 0 | 0 | 0 | 0 |
|  |  |  |  |  |  |  |  |  |  |  |  |
| Acceptance lag | NR | NR | NR | NR | NR | NR | NR | NR | NR | NR | NR |
| Lead lag | NR | NR | NR | NR | NR | NR | NR | NR | NR | NR | NR |
| Total lag | NR | NR | NR | NR | NR | NR | NR | NR | NR | NR | NR |
| Indexing lag | NR | NR | NR | NR | NR | NR | NR | NR | NR | NR | NR |

| 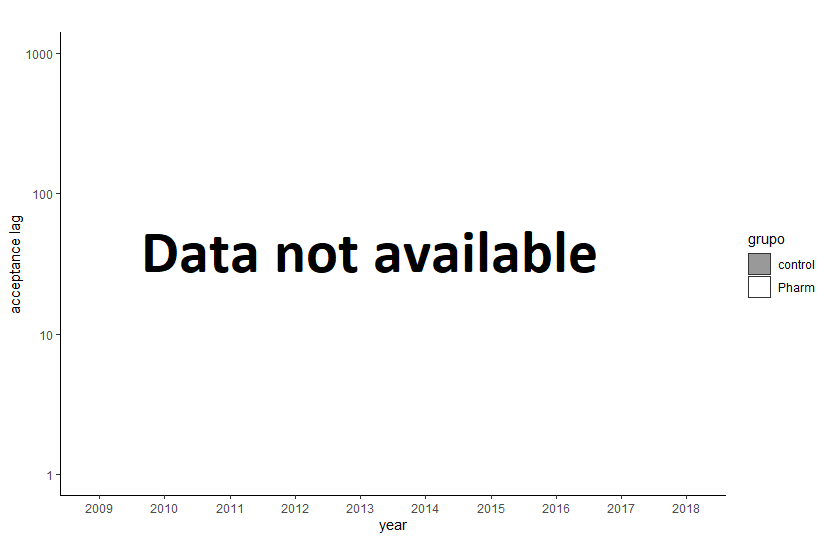 | 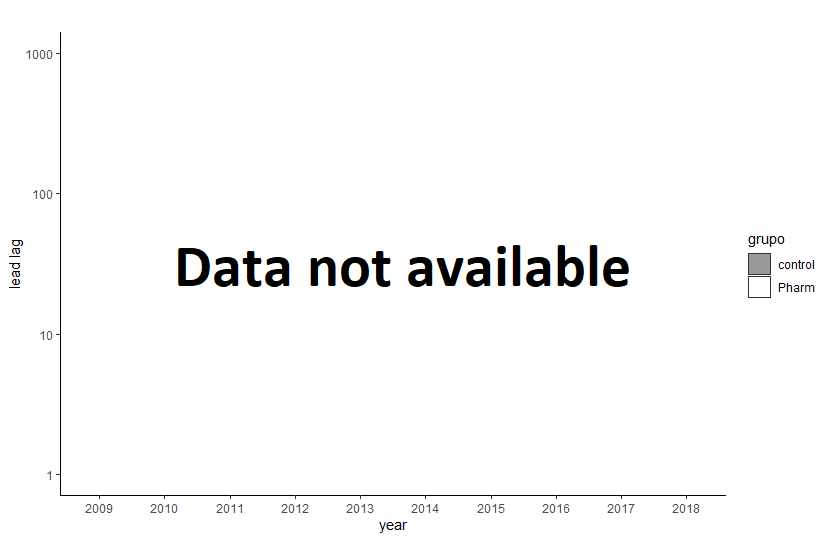 |
| --- | --- |
| 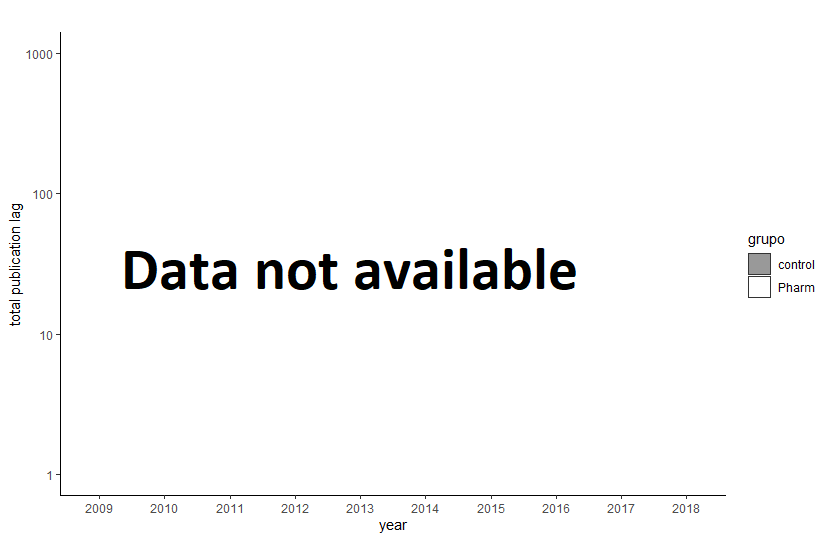 | 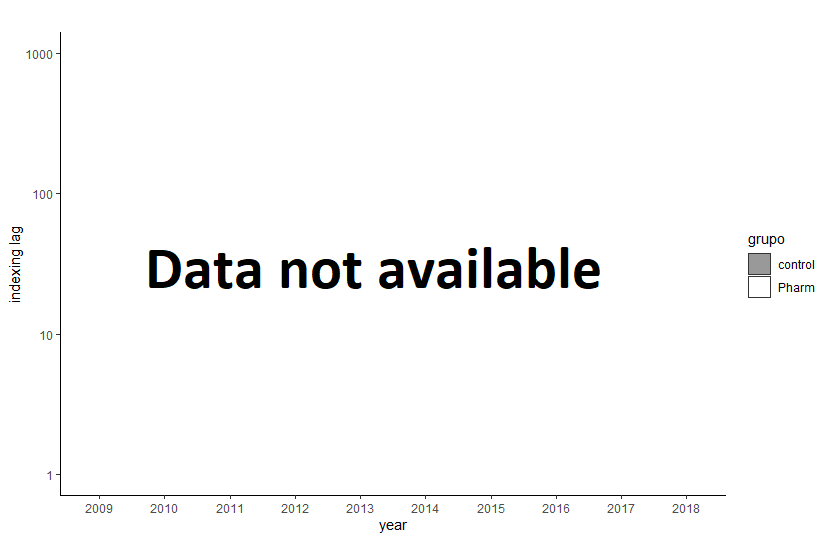 |

| **Curr Pharm Teach Learn** | **2009** | **2010** | **2011** | **2012** | **2013** | **2014** | **2015** | **2016** | **2017** | **2018** | **TOTAL** |
| --- | --- | --- | --- | --- | --- | --- | --- | --- | --- | --- | --- |
| Articles in PubMed | 0 | 0 | 0 | 0 | 0 | 0 | 4 | 22 | 164 | 222 | 412 |
| Submission date; n(%) | - | - | - | - | - | - | 0 | 20 (90.9) | 164 (100) | 221 (99.5) | 405 (98.3) |
| Acceptance date; n(%) | - | - | - | - | - | - | 0 | 0 | 164 (100) | 221 (99.5) | 385 (93.4) |
| Online publication date; n(%) |  |  |  |  |  |  | 1 (25.0) | 0 | 163 (99.4) | 221 (99.5) | 385 (93.4) |
|  |  |  |  |  |  |  |  |  |  |  |  |
| Acceptance lag | - | - | - | - | - | - | NR | 258 (219-318) | 313 (237-360) | 271 (226-342) | 290 (230-349) |
| Lead lag | - | - | - | - | - | - | NR | NR | 66 (46-76) | 24 (15-34) | 35 (21-66) |
| Total lag | - | - | - | - | - | - | NR | NR | 365 (302-425) | 304 (246-370) | 332 (268-399) |
| Indexing lag | - | - | - | - | - | - | 72 (72-72) | NR | 197 (118-315) | 127 (104-149) | 144 (108-196) |

| 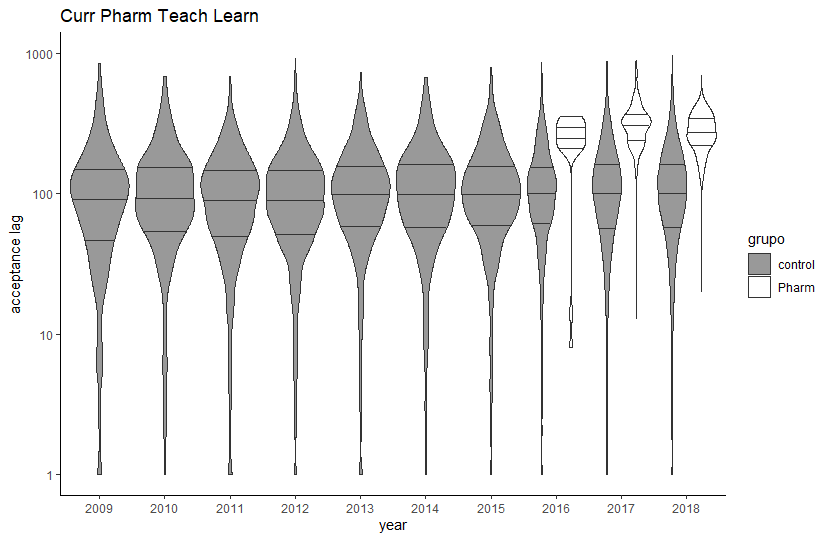 | 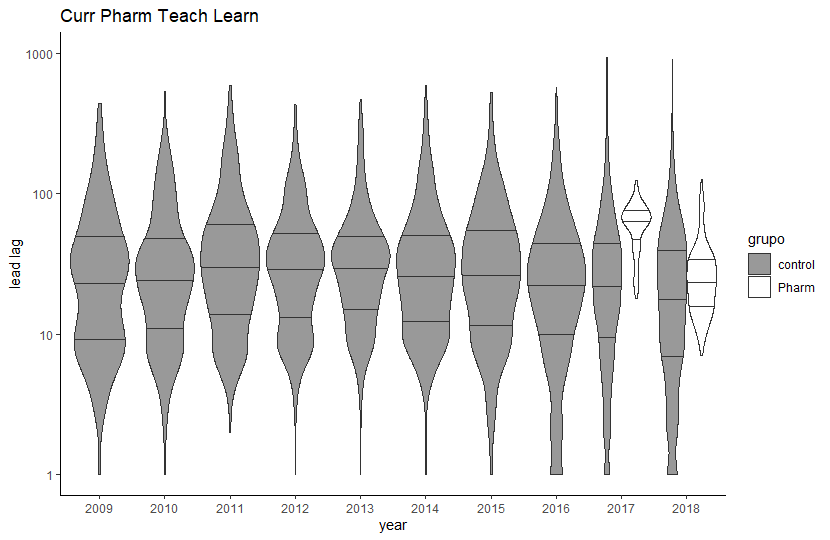 |
| --- | --- |
| 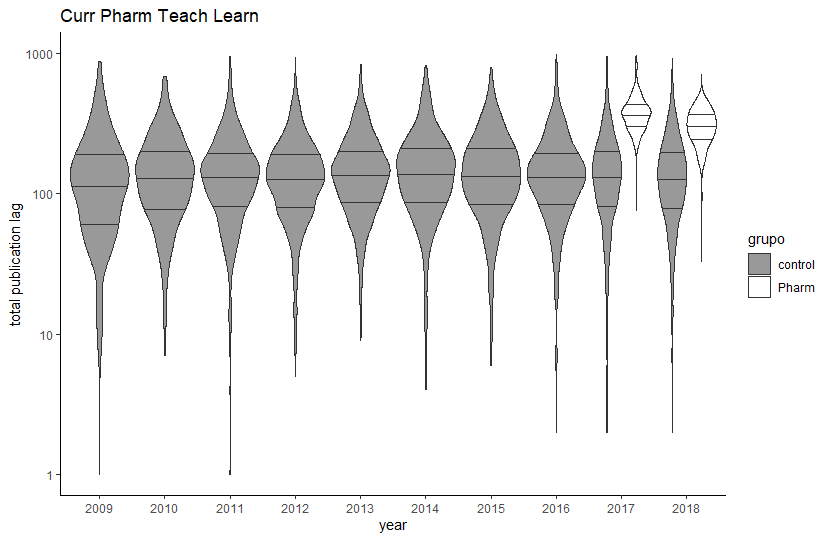 | 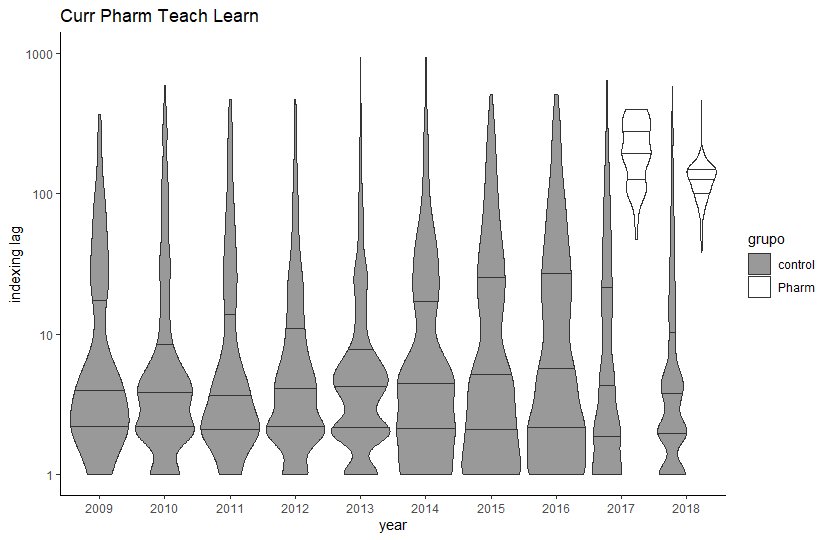 |

| **Hosp Pharm** | **2009** | **2010** | **2011** | **2012** | **2013** | **2014** | **2015** | **2016** | **2017** | **2018** | **TOTAL** |
| --- | --- | --- | --- | --- | --- | --- | --- | --- | --- | --- | --- |
| Articles in PubMed | 0 | 1 | 0 | 0 | 157 | 165 | 163 | 114 | 138 | 74 | 812 |
| Submission date; n(%) | - | 0 | - | - | 0 | 0 | 0 | 0 | 0 | 0 | 0 |
| Acceptance date; n(%) | - | 0 | - | - | 0 | 0 | 0 | 0 | 0 | 0 | 0 |
| Online publication date; n(%) | - | 0 | - | - | 0 | 0 | 89 (54.6) | 0 | 83 (60.1) | 74 (100) | 246 (30.3) |
|  |  |  |  |  |  |  |  |  |  |  |  |
| Acceptance lag | - | NR | - | - | NR | NR | NR | NR | NR | NR | NR |
| Lead lag | - | NR | - | - | NR | NR | NR | NR | NR | NR | NR |
| Total lag | - | NR | - | - | NR | NR | NR | NR | NR | NR | NR |
| Indexing lag | - | NR | - | - | NR | NR | 175 (121-295) | NR | 119 (106-149) | 113 (82-211) | 135 (102-184) |

| 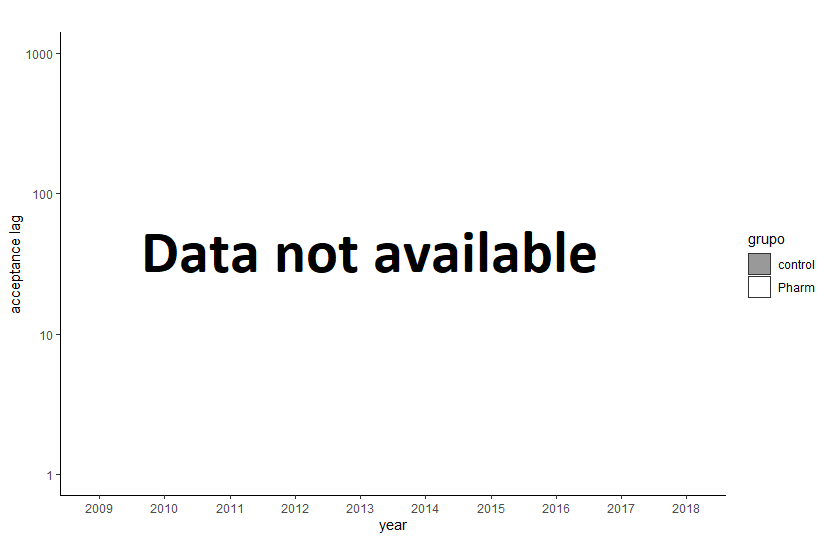 | 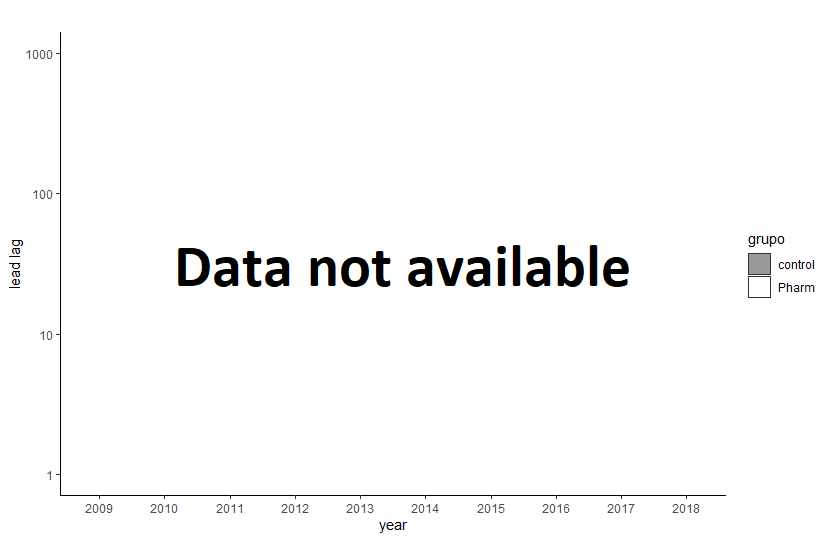 |
| --- | --- |
| 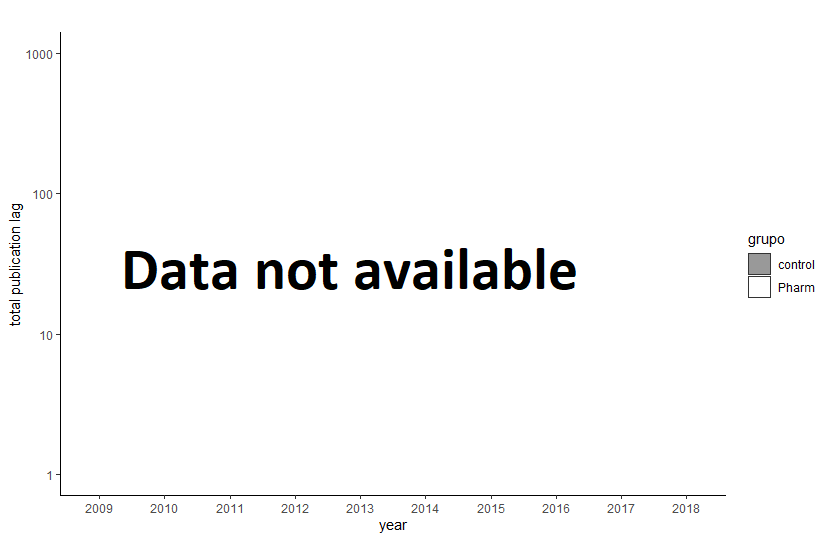 | 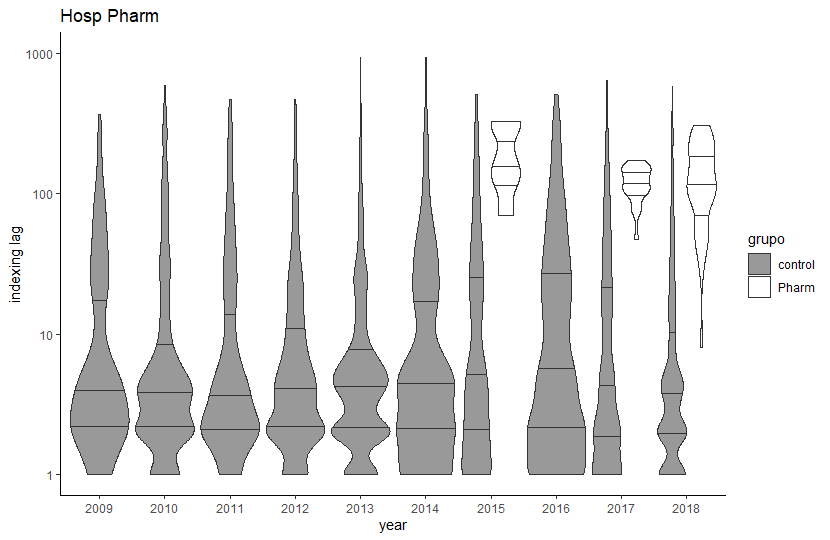 |

| **Int J Clin Pharm** | **2009** | **2010** | **2011** | **2012** | **2013** | **2014** | **2015** | **2016** | **2017** | **2018** | **TOTAL** |
| --- | --- | --- | --- | --- | --- | --- | --- | --- | --- | --- | --- |
| Articles in PubMed | 0 | 0 | 119 | 113 | 151 | 166 | 161 | 186 | 152 | 189 | 1237 |
| Submission date; n(%) | - | - | 108 (90.8) | 107 (94.7) | 146 (96.7) | 157 (94.6) | 150 (93.2) | 172 (92.5) | 146 (96.1) | 175 (92.6) | 1161 (93.9) |
| Acceptance date; n(%) | - | - | 108 (90.8) | 107 (94.7) | 146 (96.7) | 157 (94.6) | 150 (93.2) | 172 (92.5) | 146 (96.1) | 175 (92.6) | 1161 (93.9) |
| Online publication date; n(%) | - | - | 108 (90.8) | 107 (94.7) | 141 (93.4) | 153 (92.2) | 150 (93.2) | 163 (87.6) | 146 (96.1) | 175 (92.6) | 1143 (92.4) |
|  |  |  |  |  |  |  |  |  |  |  |  |
| Acceptance lag | - | - | 172 (111-226) | 158 (119-233) | 176 (122-241) | 159 (123-211) | 162 (115-208) | 170 (135-241) | 193 (149-272) | 179 (138-244) | 171 (128-234) |
| Lead lag | - | - | 25 (16-45) | 17 (13-24) | 15 (11-22) | 18 (13-25) | 14 (11-21) | 15 (11-21) | 15 (10-23) | 13 (8-22) | 16 (11-24) |
| Total lag | - | - | 211 (144-273) | 181 (134-260) | 197 (141-261) | 180 (147-232) | 179 (130-226) | 189 (149-272) | 213 (163-304) | 198 (151-272) | 192 (147-261) |
| Indexing lag | - | - | 2 (1-26) | 1 (1-3) | 1 (1-3) | 1 (1-2) | 1 (1-1) | 2 (2-2) | 2 (2-2) | 2 (2-2) | 2 (1-2) |

| 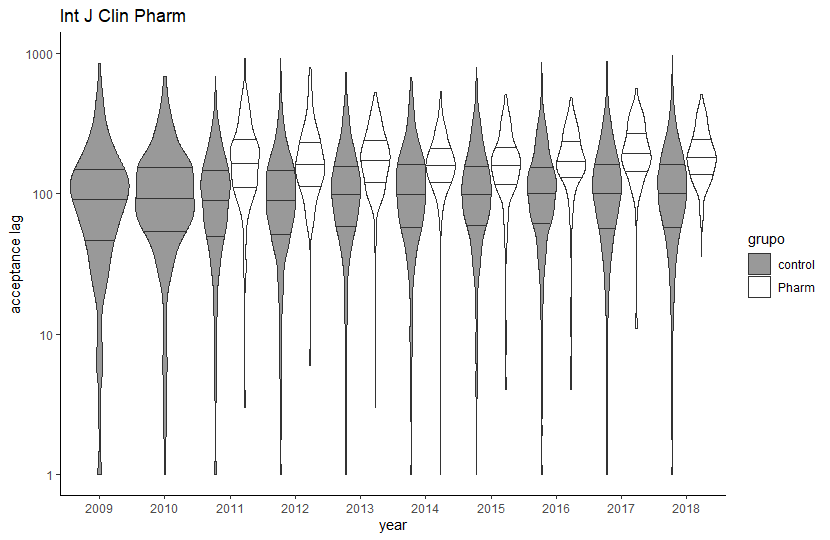 | 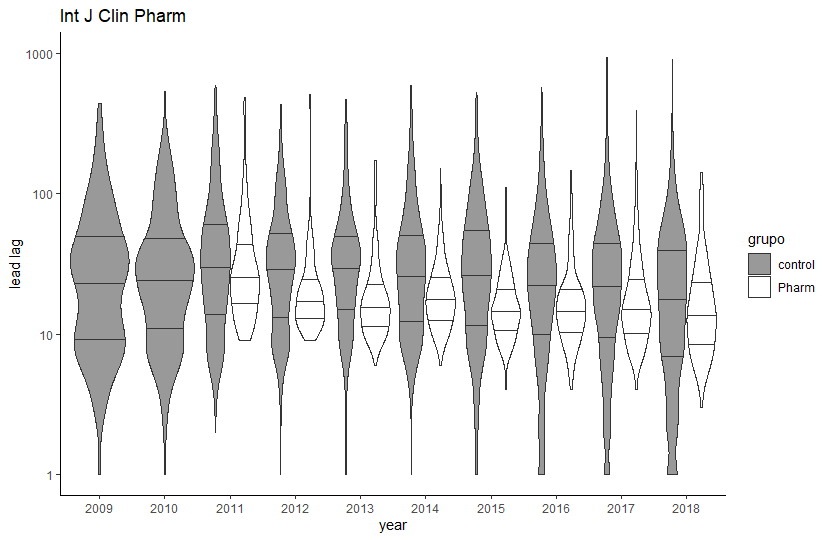 |
| --- | --- |
| 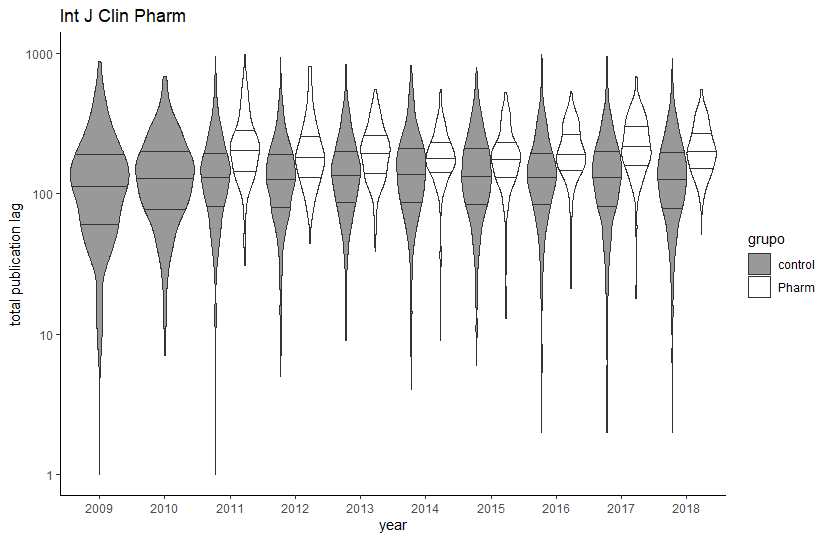 | 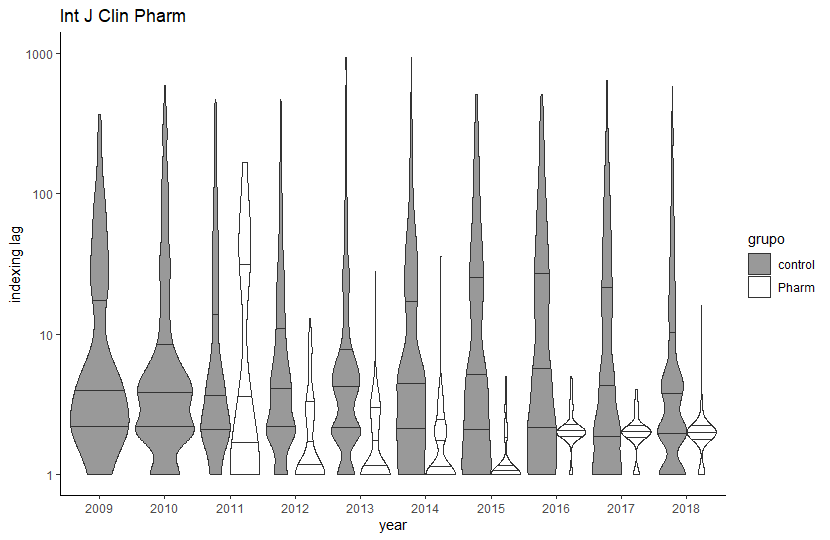 |

| **Int J Pharm Compd** | **2009** | **2010** | **2011** | **2012** | **2013** | **2014** | **2015** | **2016** | **2017** | **2018** | **TOTAL** |
| --- | --- | --- | --- | --- | --- | --- | --- | --- | --- | --- | --- |
| Articles in PubMed | 77 | 77 | 22 | 75 | 75 | 79 | 73 | 75 | 69 | 63 | 685 |
| Submission date; n(%) | 0 | 0 | 0 | 0 | 0 | 0 | 0 | 0 | 0 | 0 | 0 |
| Acceptance date; n(%) | 0 | 0 | 0 | 0 | 0 | 0 | 0 | 0 | 0 | 0 | 0 |
| Online publication date; n(%) | 0 | 0 | 0 | 0 | 0 | 0 | 0 | 0 | 0 | 0 | 0 |
|  |  |  |  |  |  |  |  |  |  |  |  |
| Acceptance lag | NR | NR | NR | NR | NR | NR | NR | NR | NR | NR | NR |
| Lead lag | NR | NR | NR | NR | NR | NR | NR | NR | NR | NR | NR |
| Total lag | NR | NR | NR | NR | NR | NR | NR | NR | NR | NR | NR |
| Indexing lag | NR | NR | NR | NR | NR | NR | NR | NR | NR | NR | NR |

| 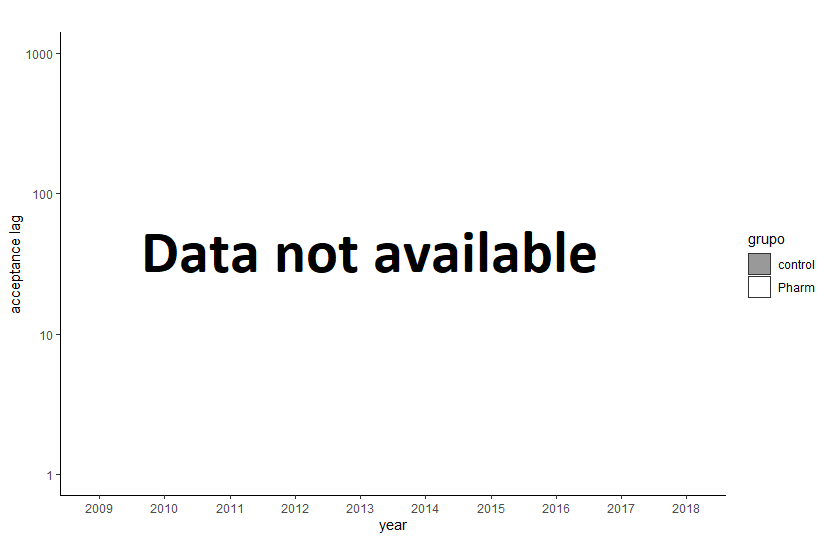 | 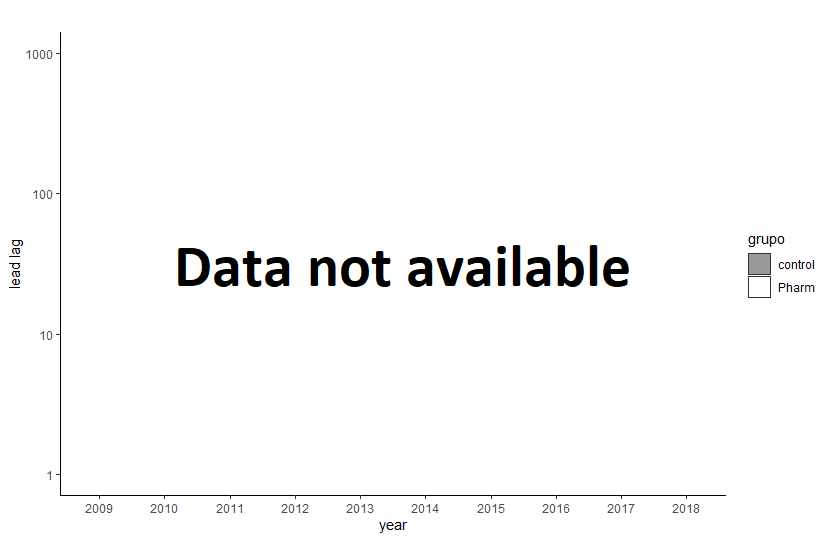 |
| --- | --- |
| 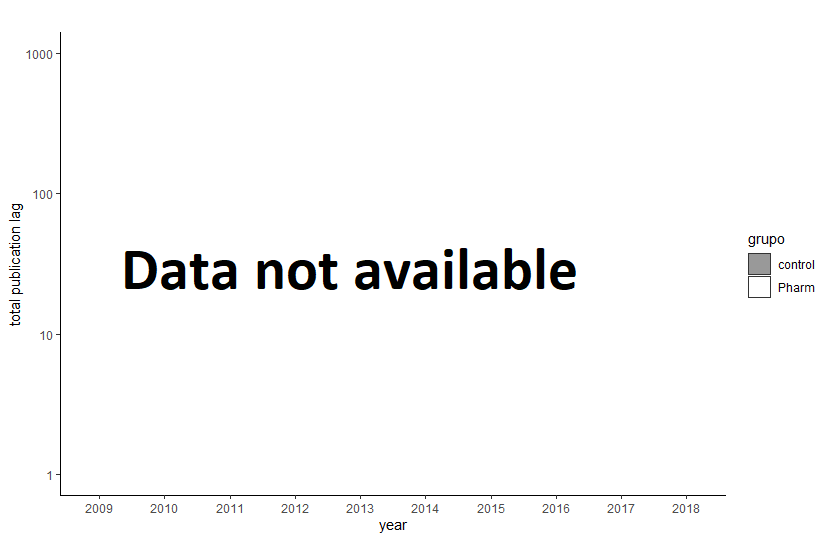 | 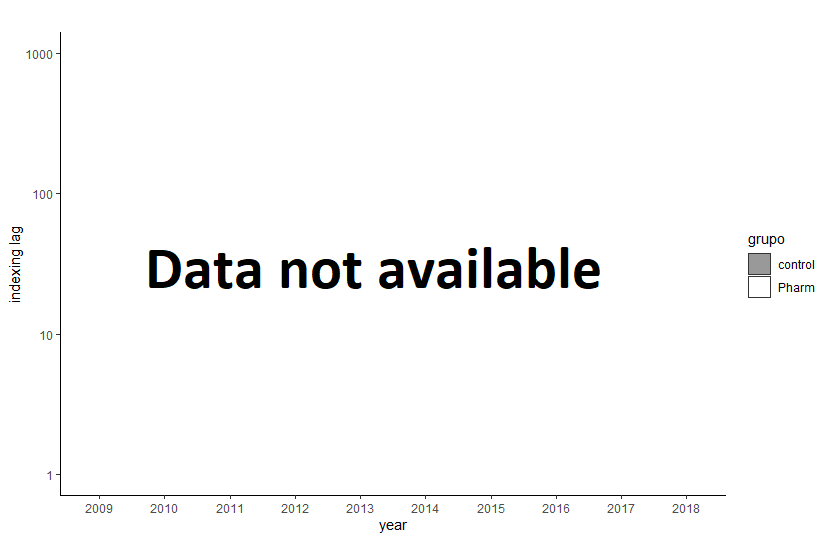 |

| **Int J Pharm Pract** | **2009** | **2010** | **2011** | **2012** | **2013** | **2014** | **2015** | **2016** | **2017** | **2018** | **TOTAL** |
| --- | --- | --- | --- | --- | --- | --- | --- | --- | --- | --- | --- |
| Articles in PubMed | 52 | 57 | 62 | 62 | 59 | 66 | 75 | 63 | 64 | 106 | 666 |
| Submission date; n(%) | 0 | 0 | 0 | 6 (9.7) | 49 (83.1) | 54 (81.8) | 59 (78.7) | 49 (77.8) | 53 (82.8) | 95 (89.6) | 365 (54.8) |
| Acceptance date; n(%) | 0 | 0 | 0 | 6 (9.7) | 49 (83.1) | 54 (81.8) | 59 (78.7) | 49 (77.8) | 53 (82.8) | 96 (90.6) | 366 (55.0) |
| Online publication date; n(%) | 0 | 5 (8.8) | 43 (69.4) | 53 (85.5) | 49 (93.1) | 53 (80.3) | 59 (78.7) | 49 (77.8) | 48 (75.0) | 97 (91.5) | 456 (68.5) |
|  |  |  |  |  |  |  |  |  |  |  |  |
| Acceptance lag | NR | NR | NR | 178 (136-248) | 250 (216-297) | 253 (196-329) | 269 (199-345) | 262 (193-368) | 192 (140-252) | 238 (182-292) | 238 (182-304) |
| Lead lag | NR | NR | NR | 71 (44-73) | 62 (47-79) | 57 (45-74) | 47 (36-62) | 50 (40-83) | 49 (33-69) | 45 (37-59) | 50 (39-70) |
| Total lag | NR | NR | NR | 250 (188-294) | 310 (280-363) | 310 (268-391) | 329 (259-421) | 332 (255-434) | 229 (191-290) | 287 (230-347) | 295 (243-366) |
| Indexing lag | NR | 35 (19-42) | 92 (26-133) | 155 (140-177) | 103 (48-170) | 2 (1-5) | 2 (1-2) | 1 (1-2) | 1 (1-1) | 1 (1-2) | 2 (1-85) |

| 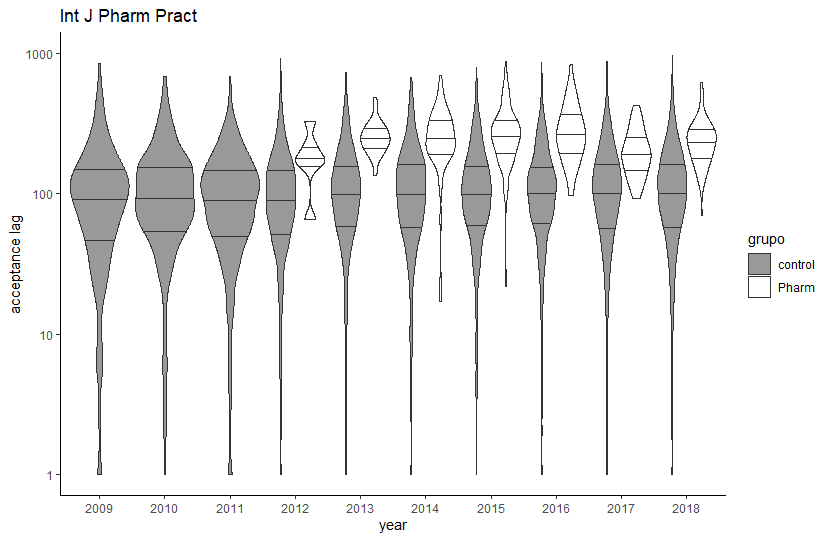 | 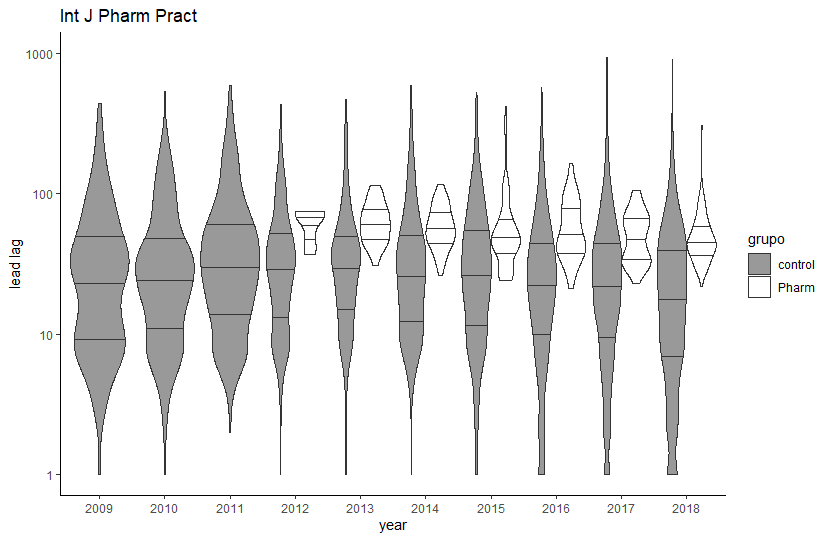 |
| --- | --- |
| 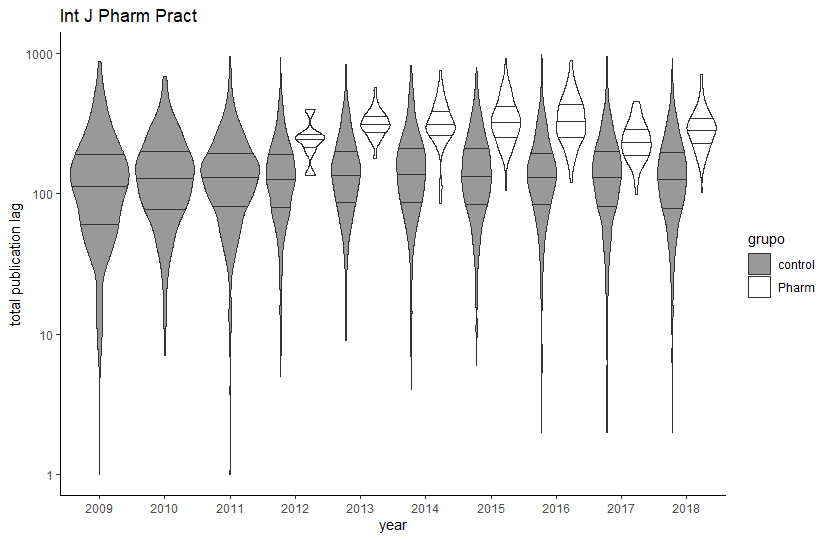 | 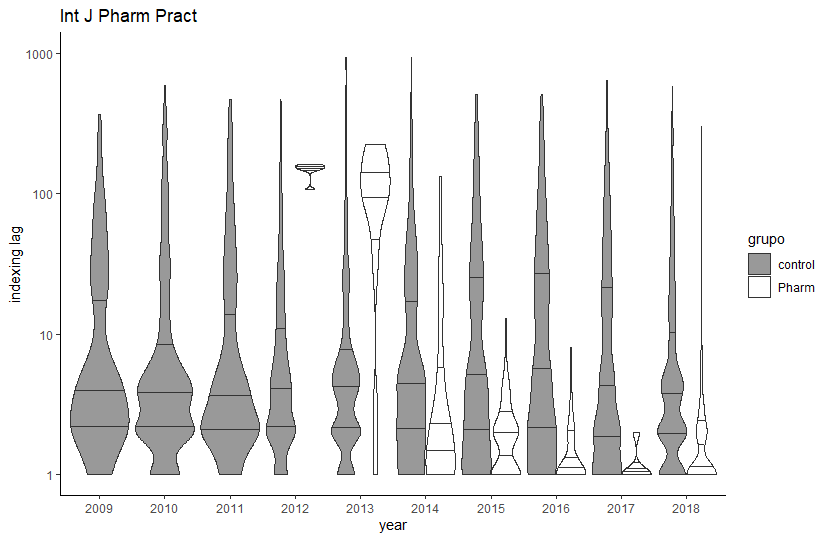 |

| **J Am Pharm Assoc (2003)** | **2009** | **2010** | **2011** | **2012** | **2013** | **2014** | **2015** | **2016** | **2017** | **2018** | **TOTAL** |
| --- | --- | --- | --- | --- | --- | --- | --- | --- | --- | --- | --- |
| Articles in PubMed | 124 | 125 | 111 | 132 | 106 | 114 | 114 | 121 | 196 | 158 | 1301 |
| Submission date; n(%) | 0 | 0 | 0 | 0 | 0 | 1 (0.9) | 0 | 74 (61.2) | 158 (80.6) | 122 (77.2) | 355 (27.3) |
| Acceptance date; n(%) | 0 | 0 | 0 | 0 | 0 | 1 (0.9) | 0 | 88 (72.7) | 158 (80.6) | 122 (77.2) | 369 (28.4) |
| Online publication date; n(%) | 1 (0.8) | 0 | 0 | 0 | 5 (4.7) | 0 | 1 (0.9) | 45 (37.2) | 138 (70.4) | 115 (72.8) | 305 (23.4) |
|  |  |  |  |  |  |  |  |  |  |  |  |
| Acceptance lag | NR | NR | NR | NR | NR | 113 | NR | 148 (101-208) | 140 (94-191) | 156 (112-210) | 147 (103-206) |
| Lead lag | NR | NR | NR | NR | NR | NR | NR | 94 (66-118) | 49 (42-58) | 41 (32-53) | 48 (39-63) |
| Total lag | NR | NR | NR | NR | NR | NR | NR | 245 (205-317) | 192 (144-241) | 194 (149-246) | 199 (153-255) |
| Indexing lag | 7 (7-7) | NR | NR | NR | 2 (2-2) | NR | 4 (4-4) | 6 (5-7) | 5 (4-5) | 5 (4-5) | 5 (4-5) |

| 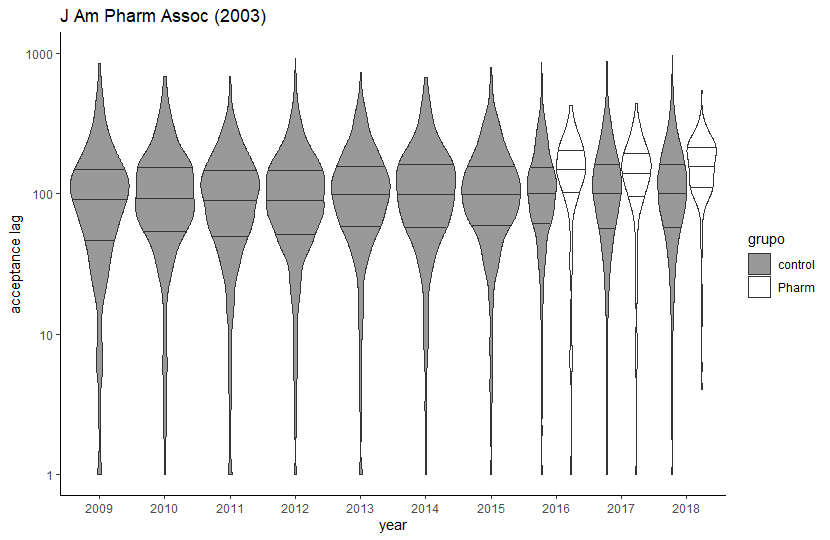 | 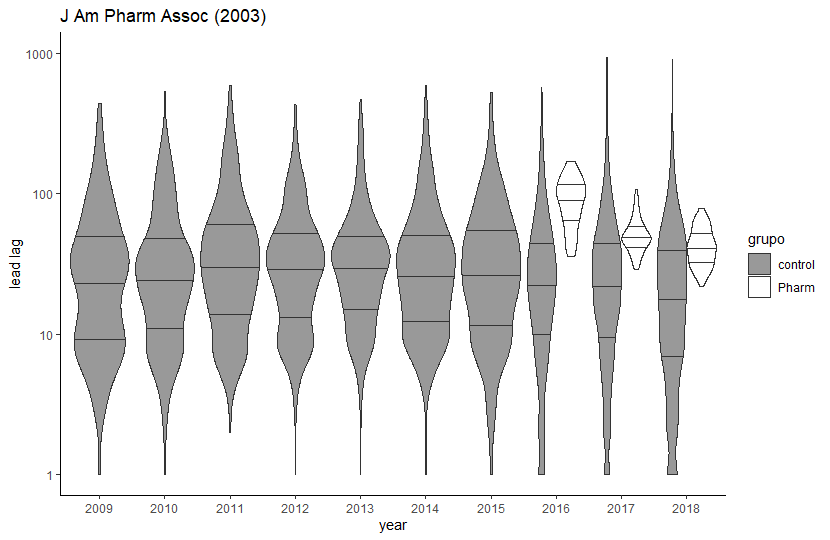 |
| --- | --- |
| 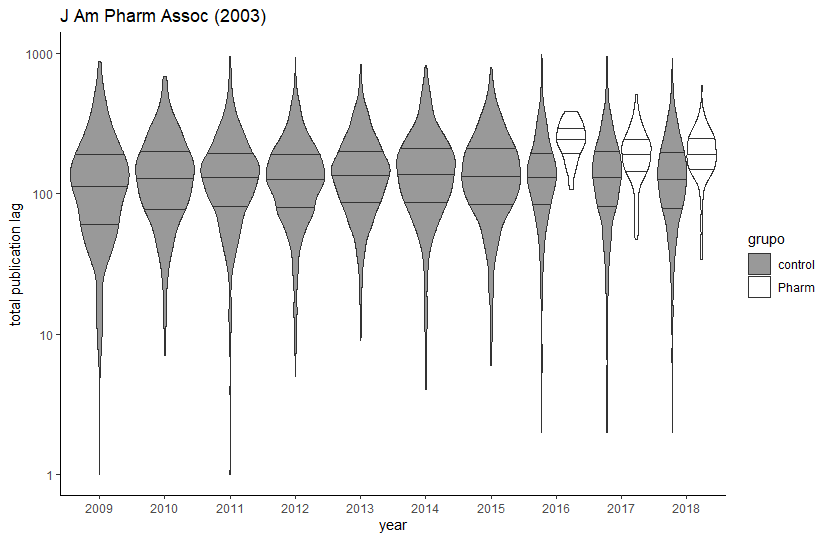 | 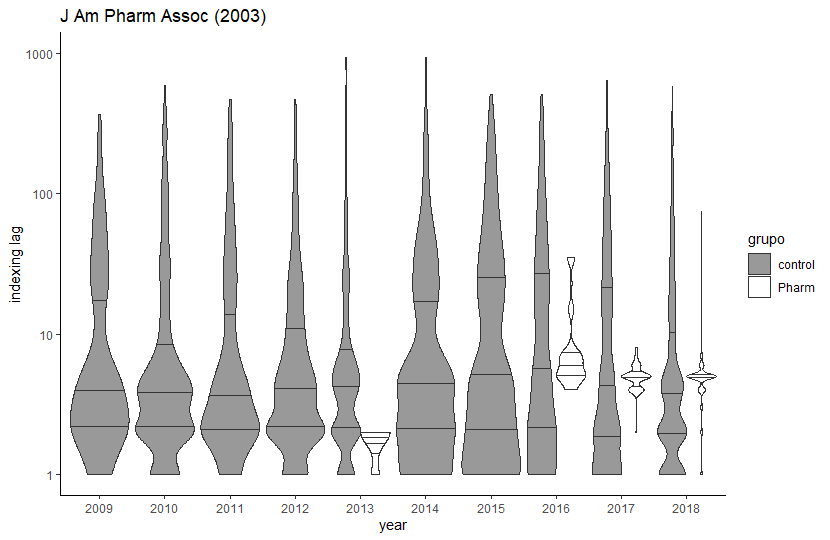 |

| **J Basic Clin Pharm** | **2009** | **2010** | **2011** | **2012** | **2013** | **2014** | **2015** | **2016** | **2017** | **2018** | **TOTAL** |
| --- | --- | --- | --- | --- | --- | --- | --- | --- | --- | --- | --- |
| Articles in PubMed | 9 | 38 | 27 | 26 | 19 | 28 | 22 | 26 | 0 | 0 | 195 |
| Submission date; n(%) | 9 (100) | 36 (94.7) | 27 (100) | 13 (50.0) | 0 | 0 | 0 | 0 | - | - | 85 (43.6) |
| Acceptance date; n(%) | 8 (88.9) | 36 (94.7) | 27 (100) | 13 (50.0) | 0 | 0 | 0 | 0 | - | - | 84 (43.1) |
| Online publication date; n(%) | 9 (100) | 36 (94.7) | 26 (96.3) | 13 (50.0) | 0 | 0 | 0 | 0 | - | - | 84 (43.1) |
|  |  |  |  |  |  |  |  |  |  |  |  |
| Acceptance lag | 20 (15-22) | 61 (41-122) | 50 (27-94) | 35 (15-58) | NR | NR | NR | NR | - | - | 46 (27-80) |
| Lead lag | 13 (11-20) | 37 (24-66) | 61 (18-84) | 13 (11-26) | NR | NR | NR | NR | - | - | 30 (15-66) |
| Total lag | 36 (27-41) | 123 (82-226) | 113 (61-210) | 53 (23-103) | NR | NR | NR | NR | - | - | 88 (51-154) |
| Indexing lag | NR | NR | NR | NR | NR | NR | NR | NR | - | - | NR |

| 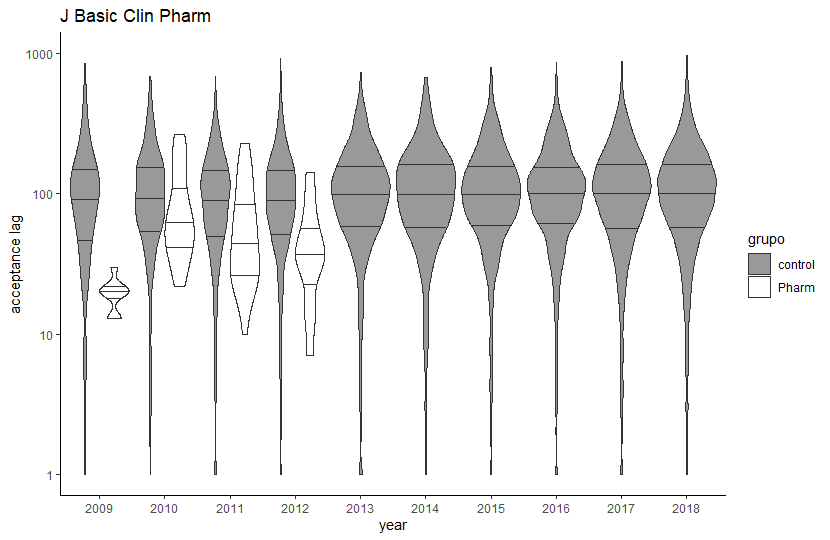 | 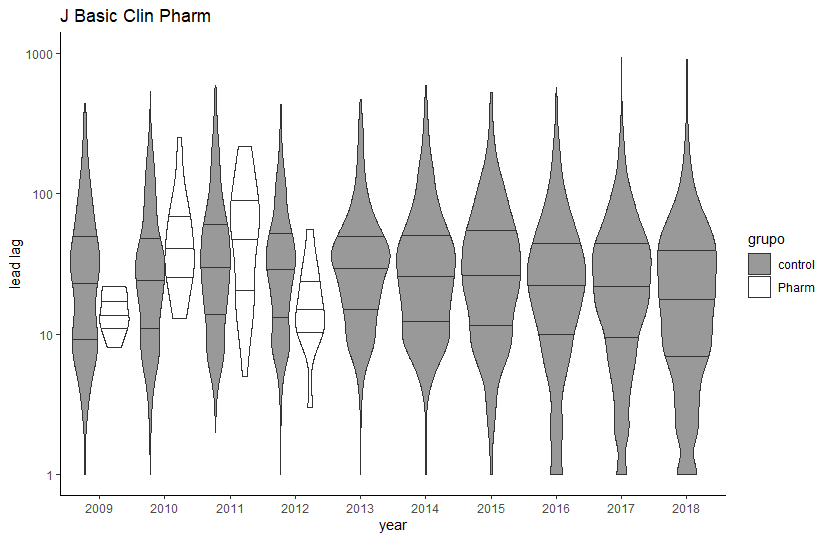 |
| --- | --- |
| 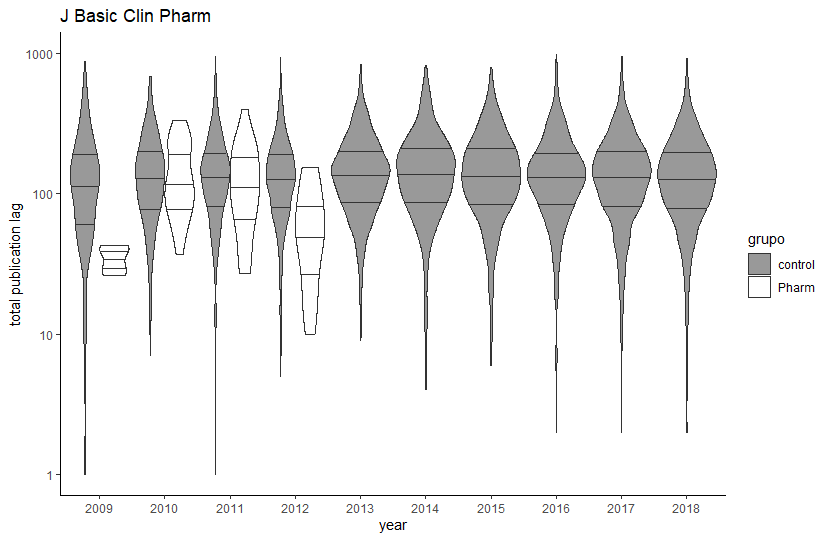 | 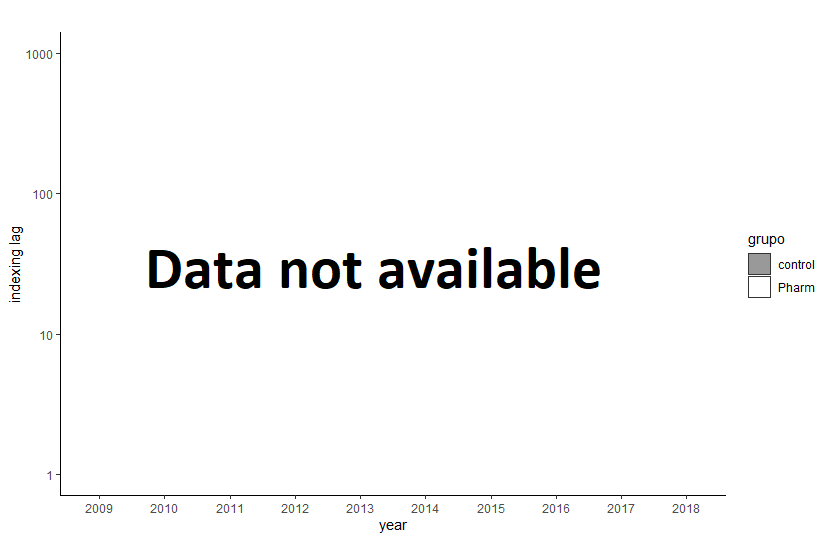 |

| **J Manag Care Spec Pharm** | **2009** | **2010** | **2011** | **2012** | **2013** | **2014** | **2015** | **2016** | **2017** | **2018** | **TOTAL** |
| --- | --- | --- | --- | --- | --- | --- | --- | --- | --- | --- | --- |
| Articles in PubMed | 0 | 0 | 0 | 0 | 0 | 101 | 114 | 152 | 151 | 146 | 664 |
| Submission date; n(%) | - | - | - | - | - | 0 | 0 | 0 | 0 | 0 | 0 |
| Acceptance date; n(%) | - | - | - | - | - | 0 | 0 | 0 | 0 | 0 | 0 |
| Online publication date; n(%) | - | - | - | - | - | 0 | 0 | 13 (8.6) | 21 (13.9) | 21 (14.4) | 55 (8.3) |
|  |  |  |  |  |  |  |  |  |  |  |  |
| Acceptance lag | - | - | - | - | - | NR | NR | NR | NR | NR | NR |
| Lead lag | - | - | - | - | - | NR | NR | NR | NR | NR | NR |
| Total lag | - | - | - | - | - | NR | NR | NR | NR | NR | NR |
| Indexing lag | - | - | - | - | - | NR | NR | 30 (17-103) | 64 (23-99) | 1 (1-2) | 27 (1-81) |

| 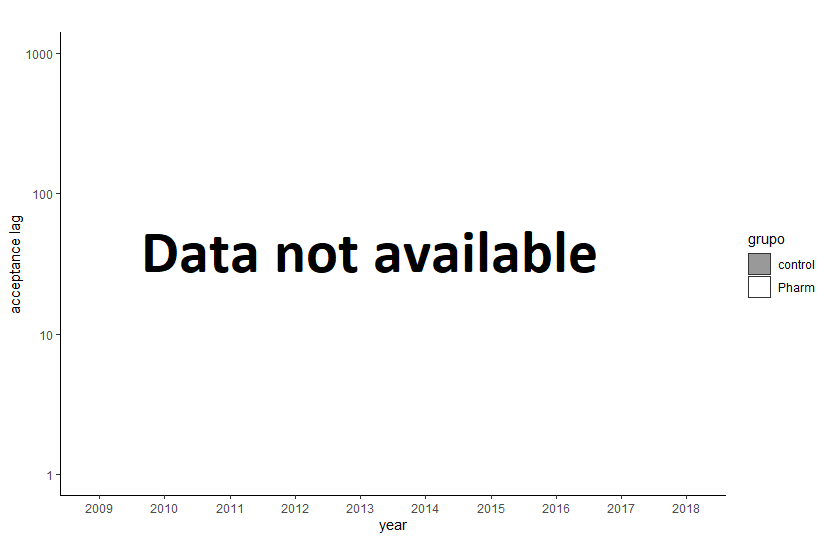 | 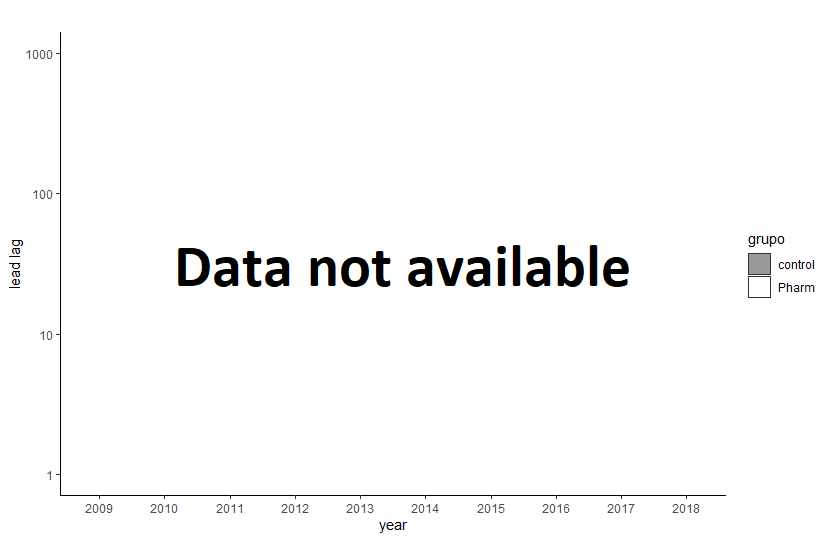 |
| --- | --- |
| 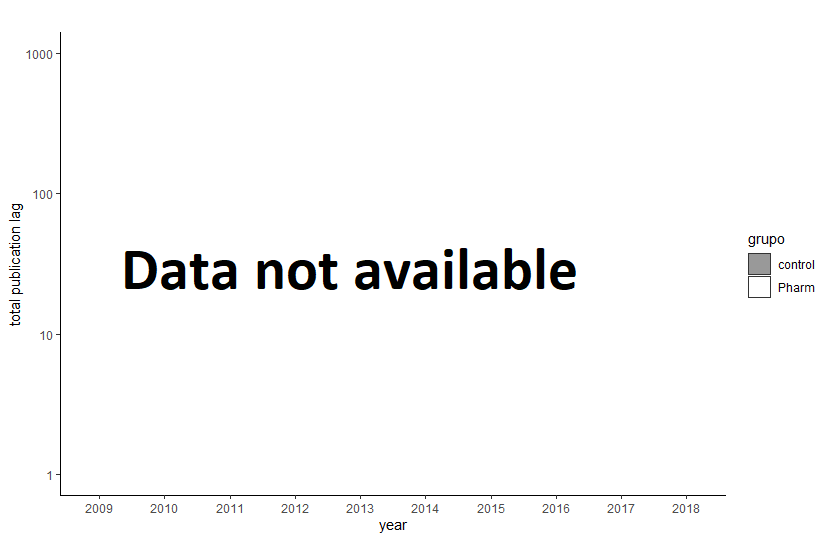 | 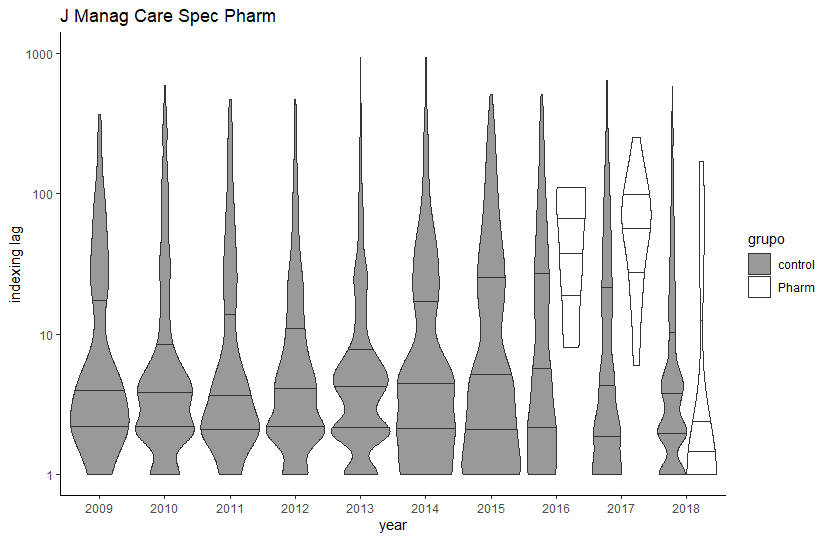 |

| **J Pain Palliat Care Pharmacother** | **2009** | **2010** | **2011** | **2012** | **2013** | **2014** | **2015** | **2016** | **2017** | **2018** | **TOTAL** |
| --- | --- | --- | --- | --- | --- | --- | --- | --- | --- | --- | --- |
| Articles in PubMed | 51 | 58 | 51 | 65 | 63 | 95 | 70 | 71 | 42 | 20 | 586 |
| Submission date; n(%) | 0 | 0 | 0 | 0 | 0 | 0 | 0 | 0 | 0 | 0 | 0 |
| Acceptance date; n(%) | 0 | 0 | 0 | 0 | 0 | 0 | 0 | 0 | 0 | 0 | 0 |
| Online publication date; n(%) | 0 | 0 | 10 (19.6) | 9 (13.8) | 46 (73.0) | 65 (68.4) | 34 (48.6) | 62 (87.3) | 34 (81.0) | 19 (95.0) | 279 (47.6) |
|  |  |  |  |  |  |  |  |  |  |  |  |
| Acceptance lag | NR | NR | NR | NR | NR | NR | NR | NR | NR | NR | NR |
| Lead lag | NR | NR | NR | NR | NR | NR | NR | NR | NR | NR | NR |
| Total lag | NR | NR | NR | NR | NR | NR | NR | NR | NR | NR | NR |
| Indexing lag | NR | NR | 2 (2-2) | 2 (2-2) | 2 (2-3) | 1 (1-2) | 1 (1-1) | 1 (1-2) | 1 (1-20) | 1 (1-1) | 1 (1-2) |

| 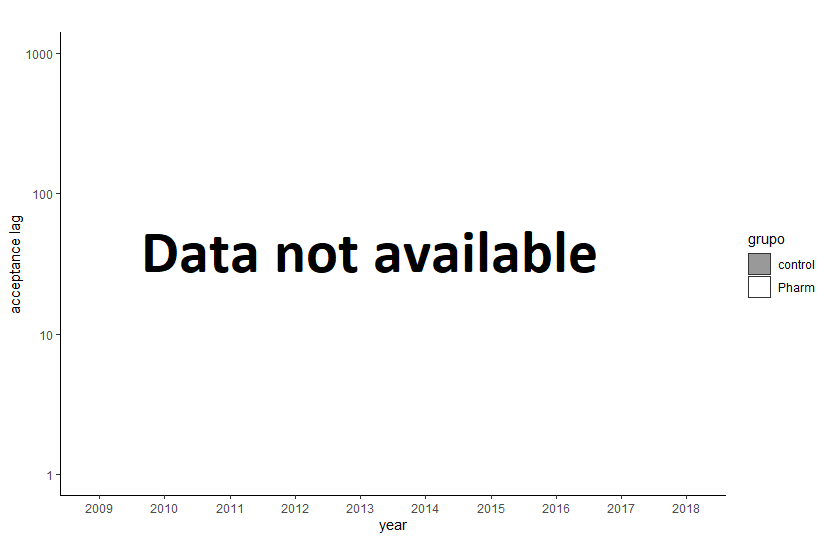 | 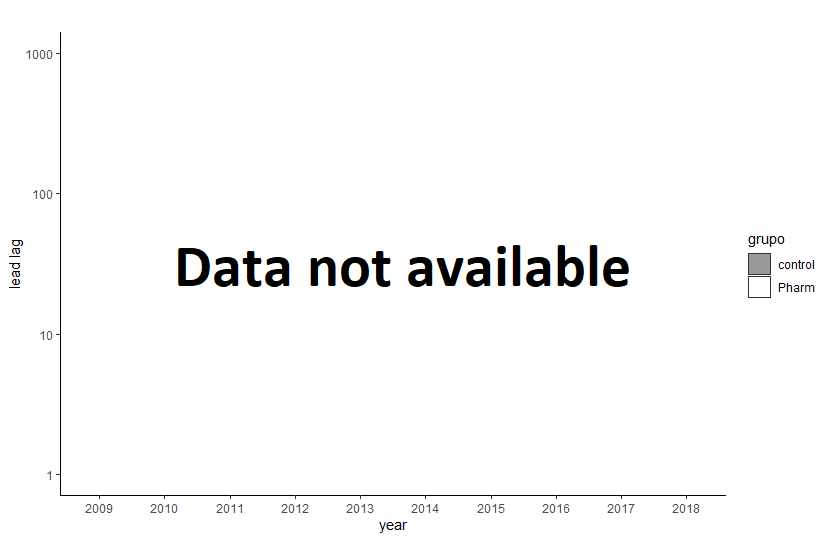 |
| --- | --- |
| 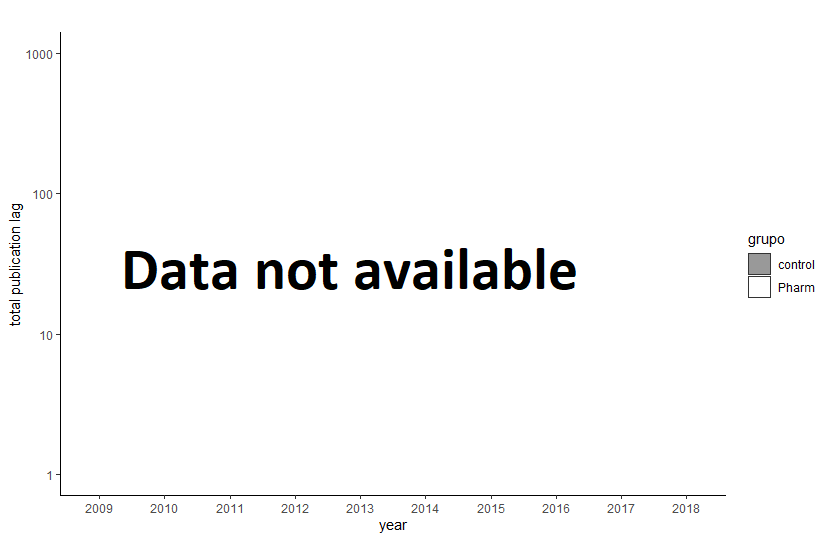 | 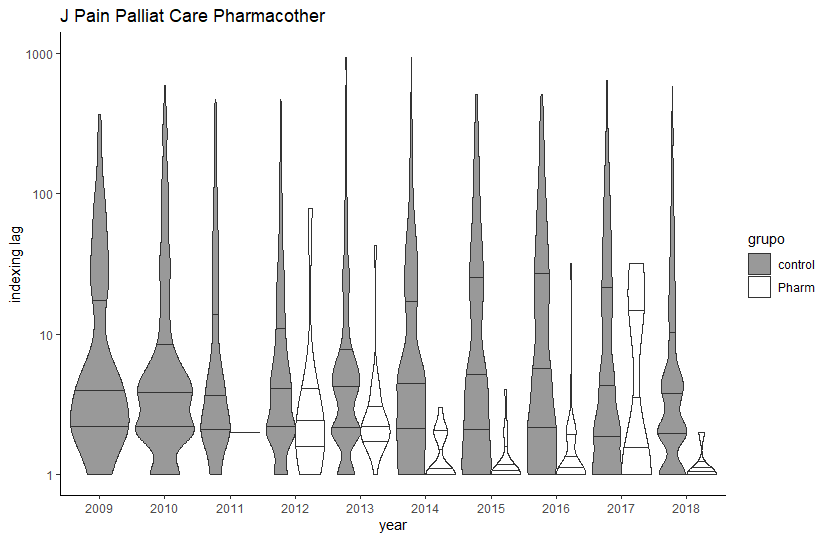 |

| **J Pharm Bioallied Sci** | **2009** | **2010** | **2011** | **2012** | **2013** | **2014** | **2015** | **2016** | **2017** | **2018** | **TOTAL** |
| --- | --- | --- | --- | --- | --- | --- | --- | --- | --- | --- | --- |
| Articles in PubMed | 0 | 71 | 89 | 189 | 109 | 97 | 261 | 107 | 106 | 34 | 1063 |
| Submission date; n(%) | - | 59 (83.1) | 67 (75.3) | 123 (65.1) | 93 (85.3) | 89 (91.8) | 121 (46.4) | 0 | 0 | 0 | 552 (51.9) |
| Acceptance date; n(%) | - | 59 (83.1) | 67 (75.3) | 123 (65.1) | 93 (85.3) | 89 (91.8) | 121 (46.4) | 0 | 0 | 0 | 552 (51.9) |
| Online publication date; n(%) | - | 0 | 0 | 0 | 0 | 0 | 0 | 0 | 0 | 0 | 0 |
|  |  |  |  |  |  |  |  |  |  |  |  |
| Acceptance lag | - | 51 (21-80) | 75 (47-101) | 56 (56-71) | 8 (2-151) | 10 (10-108) | 9 (9-68) | NR | NR | NR | 56 (9-91) |
| Lead lag | - | NR | NR | NR | NR | NR | NR | NR | NR | NR | NR |
| Total lag | - | NR | NR | NR | NR | NR | NR | NR | NR | NR | NR |
| Indexing lag | - | NR | NR | NR | NR | NR | NR | NR | NR | NR | NR |

| 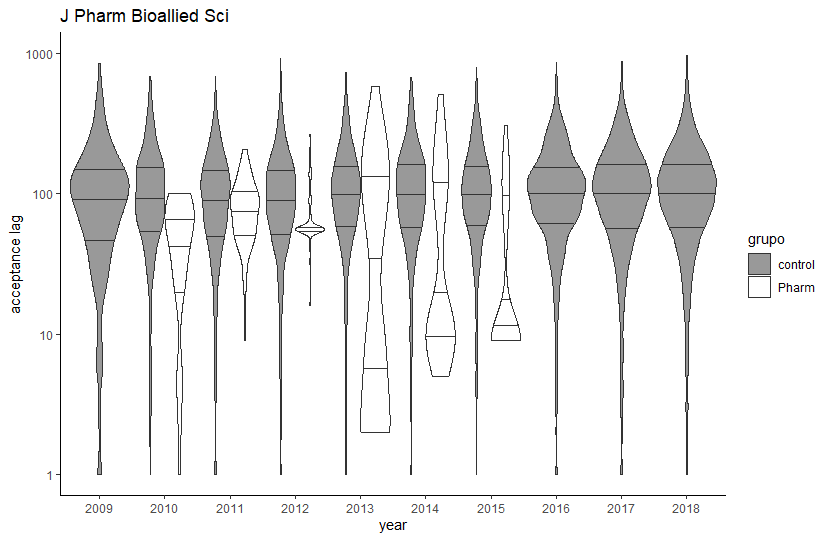 | 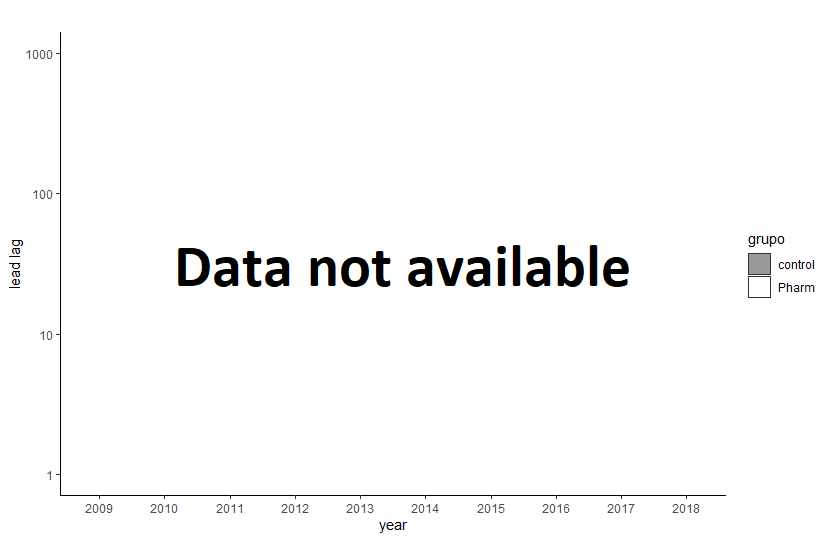 |
| --- | --- |
| 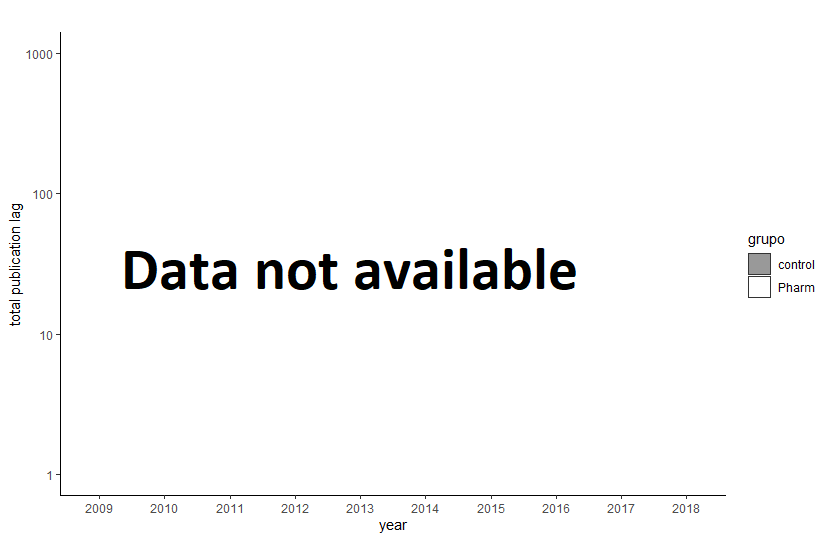 | 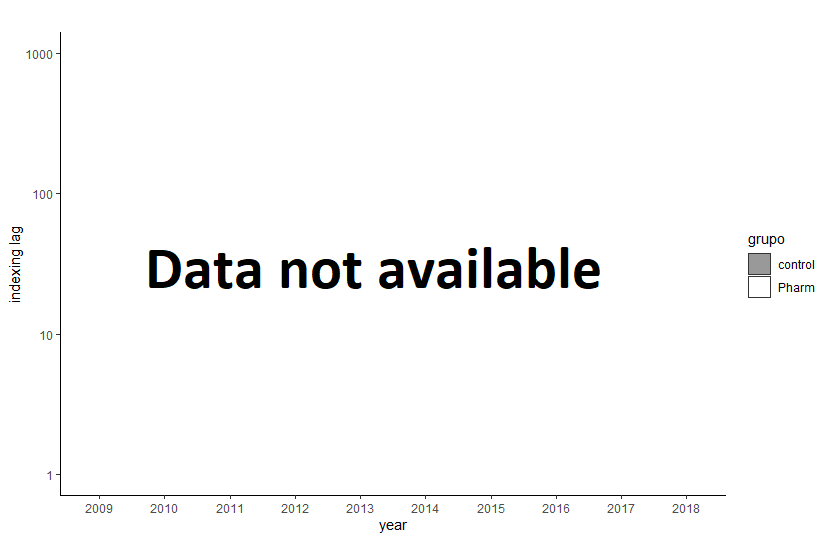 |

| **J Pharm Policy Pract** | **2009** | **2010** | **2011** | **2012** | **2013** | **2014** | **2015** | **2016** | **2017** | **2018** | **TOTAL** |
| --- | --- | --- | --- | --- | --- | --- | --- | --- | --- | --- | --- |
| Articles in PubMed | 0 | 0 | 0 | 0 | 11 | 19 | 31 | 39 | 39 | 30 | 169 |
| Submission date; n(%) | - | - | - | - | 11 (100) | 18 (94.7) | 29 (93.5) | 38 (97.4) | 39 (100) | 30 (100) | 165 (97.6) |
| Acceptance date; n(%) | - | - | - | - | 11 (100) | 18 (94.7) | 29 (93.5) | 38 (97.4) | 39 (100) | 30 (100) | 165 (97.6) |
| Online publication date; n(%) | - | - | - | - | 11 (100) | 19 (100) | 30 (96.8) | 39 (100) | 39 (100) | 30 (100) | 168 (99.4) |
|  |  |  |  |  |  |  |  |  |  |  |  |
| Acceptance lag | - | - | - | - | 63 (6-140 | 184 (69-254) | 117 (95-159) | 115 (75-153) | 111 (68-149) | 86 (65-127) | 111 (69-160) |
| Lead lag | - | - | - | - | 45 (35-51) | 22 (10-36) | 25 (19-37) | 12 (9-22) | 18 (10-31) | 36 (22-55) | 22 (13-36) |
| Total lag | - | - | - | - | 68 (41-184) | 194 (103-276) | 144 (113-193) | 134 (83-184) | 126 (97-187) | 133 (103-172) | 138 (97-188) |
| Indexing lag | - | - | - | - | NR | 24 (19-52) | 15 (4-43) | 4 (2-8) | 7 (4-8) | 8 (6-11) | 7 (4-12) |

| 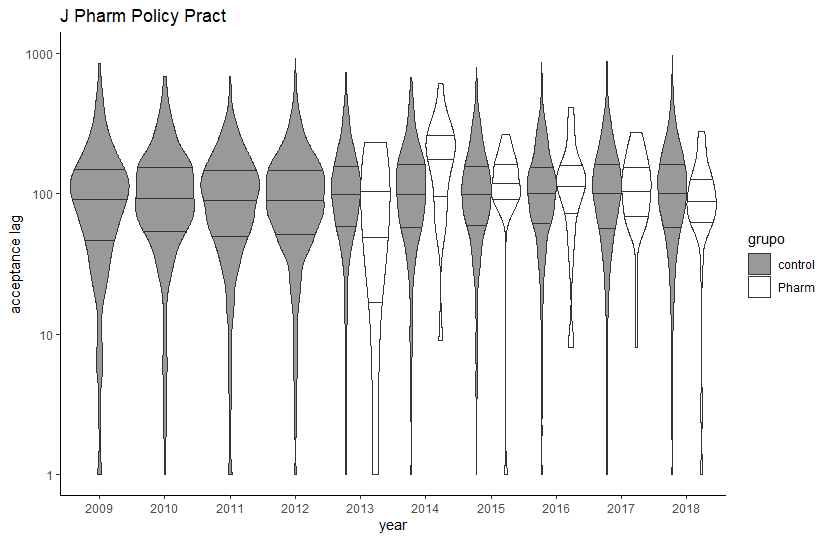 | 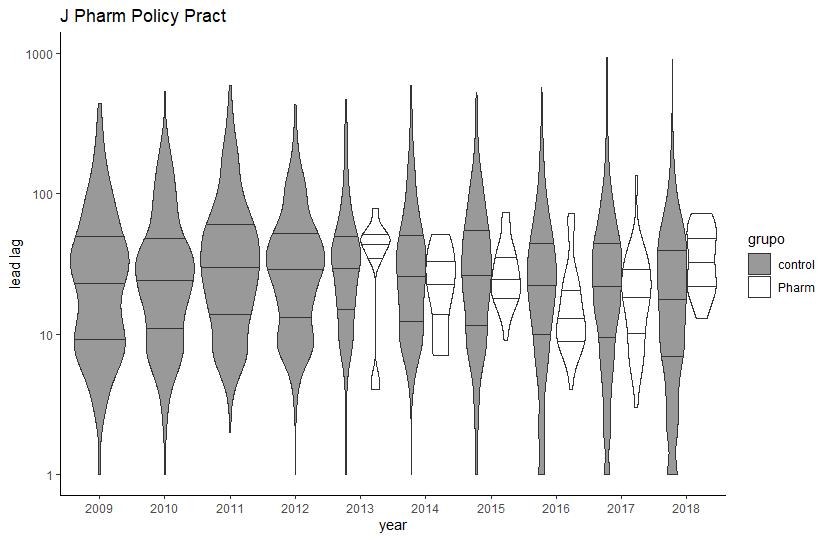 |
| --- | --- |
| 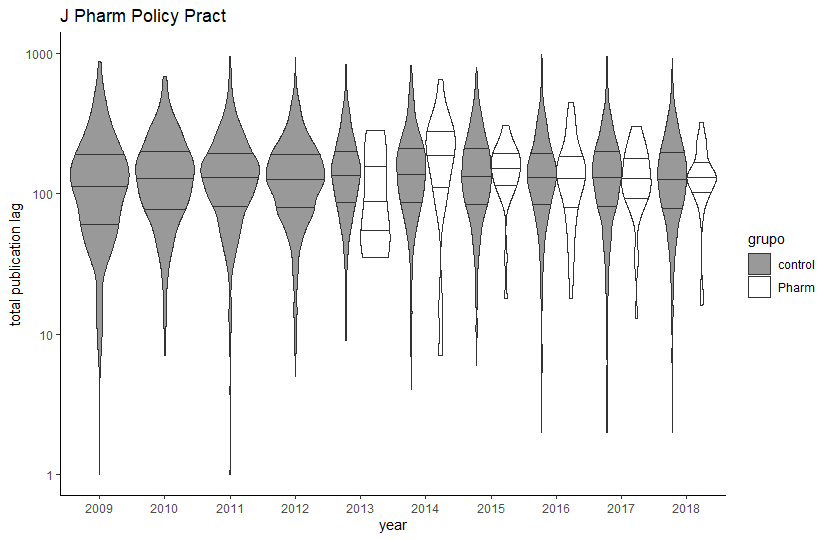 | 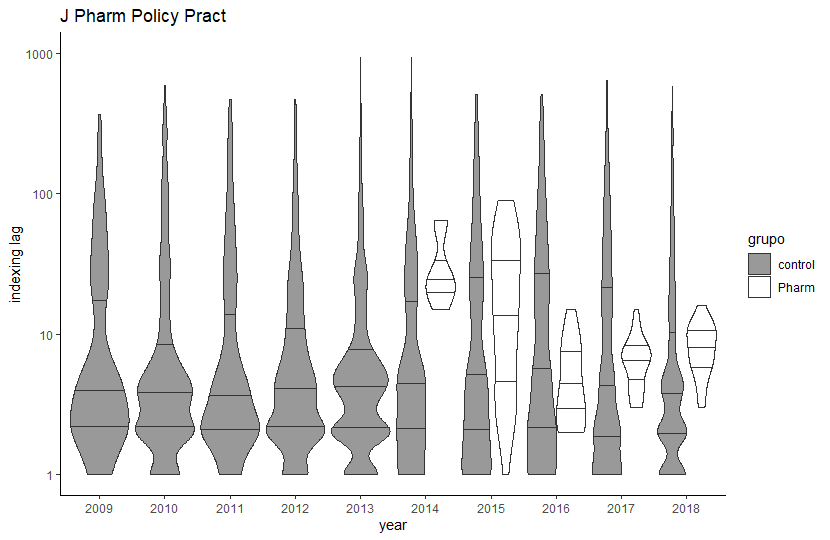 |

| **J Pharm Pract** | **2009** | **2010** | **2011** | **2012** | **2013** | **2014** | **2015** | **2016** | **2017** | **2018** | **TOTAL** |
| --- | --- | --- | --- | --- | --- | --- | --- | --- | --- | --- | --- |
| Articles in PubMed | 0 | 72 | 67 | 76 | 80 | 76 | 81 | 78 | 146 | 201 | 877 |
| Submission date; n(%) | - | 0 | 0 | 0 | 0 | 0 | 0 | 0 | 0 | 0 | 0 |
| Acceptance date; n(%) | - | 0 | 0 | 0 | 0 | 0 | 0 | 0 | 0 | 0 | 0 |
| Online publication date; n(%) | - | 54 (75.0) | 45 (67.2) | 54 (71.1) | 64 (80.0) | 60 (78.9) | 61 (75.3) | 66 (84.6) | 100 (68.5) | 198 (98.5) | 702 (80.0) |
|  |  |  |  |  |  |  |  |  |  |  |  |
| Acceptance lag | - | NR | NR | NR | NR | NR | NR | NR | NR | NR | NR |
| Lead lag | - | NR | NR | NR | NR | NR | NR | NR | NR | NR | NR |
| Total lag | - | NR | NR | NR | NR | NR | NR | NR | NR | NR | NR |
| Indexing lag | - | 350 (263-379) | 91 (2-147) | 2 (2-4) | 3 (2-4) | 2 (2-3) | 3 (2-4) | 2 (2-3) | 3 (2-306) | 2 (2-64) | 3 (2-59) |

| 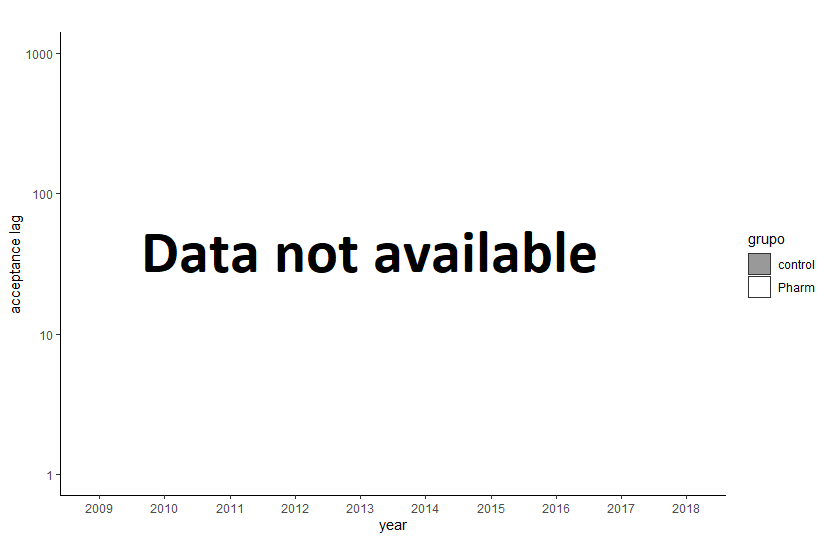 | 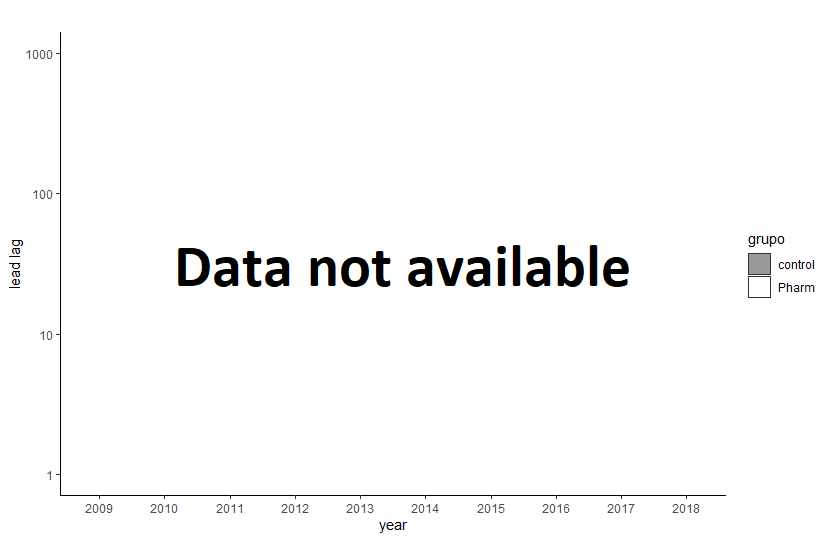 |
| --- | --- |
| 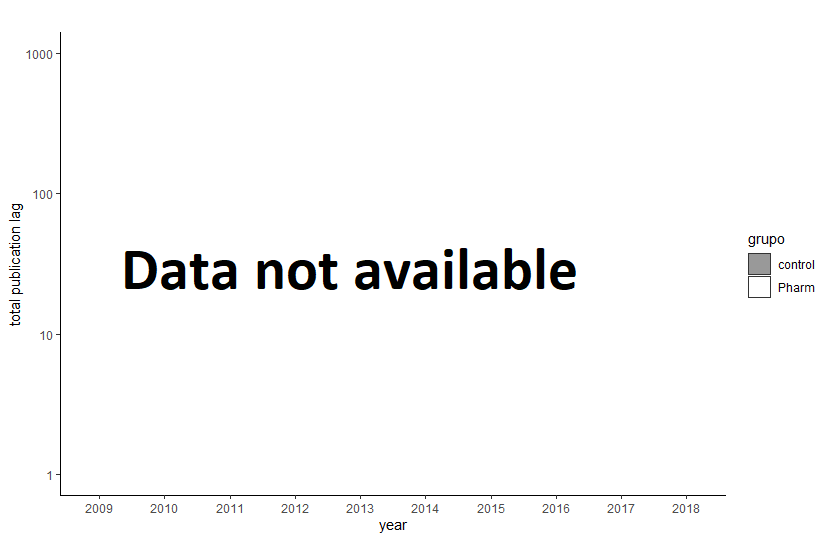 | 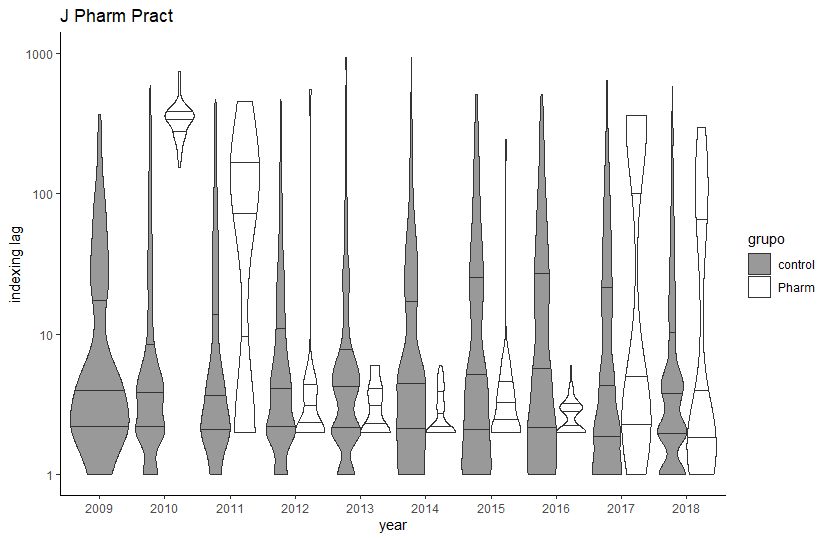 |

| **J Pharmacol Pharmacother** | **2009** | **2010** | **2011** | **2012** | **2013** | **2014** | **2015** | **2016** | **2017** | **2018** | **TOTAL** |
| --- | --- | --- | --- | --- | --- | --- | --- | --- | --- | --- | --- |
| Articles in PubMed | 0 | 31 | 91 | 97 | 95 | 70 | 70 | 47 | 40 | 0 | 541 |
| Submission date; n(%) | - | 0 | 0 | 0 | 0 | 44 (62.9) | 43 (61.4) | 0 | 0 | - | 87 (16.1) |
| Acceptance date; n(%) | - | 0 | 0 | 0 | 0 | 44 (62.9) | 39 (55.7) | 0 | 0 | - | 83 (15.3) |
| Online publication date; n(%) | - | 0 | 0 | 0 | 0 | 0 | 0 | 0 | 0 | - | 0 |
|  |  |  |  |  |  |  |  |  |  |  |  |
| Acceptance lag | - | NR | NR | NR | NR | 103 (68-165) | 154 (98-184) | NR | NR | - | 127 (87-174) |
| Lead lag | - | NR | NR | NR | NR | NR | NR | NR | NR | - | NR |
| Total lag | - | NR | NR | NR | NR | NR | NR | NR | NR | - | NR |
| Indexing lag | - | NR | NR | NR | NR | NR | NR | NR | NR | - | NR |

| 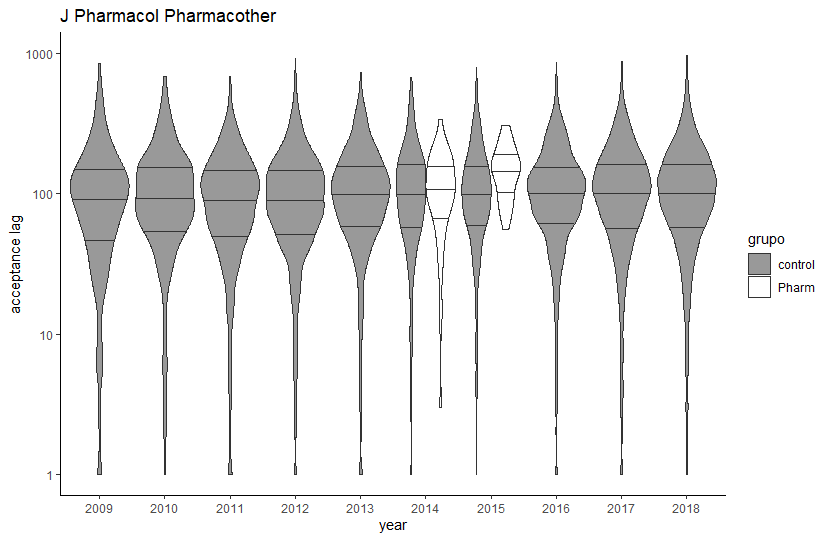 | 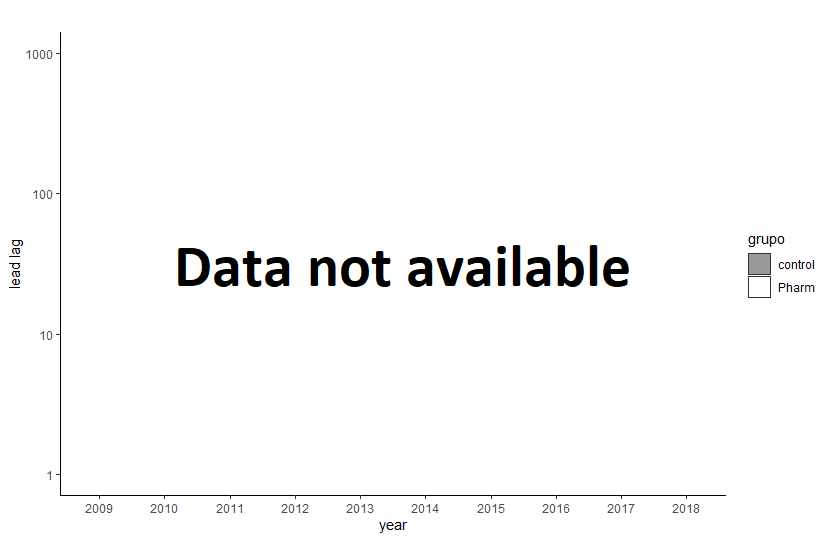 |
| --- | --- |
| 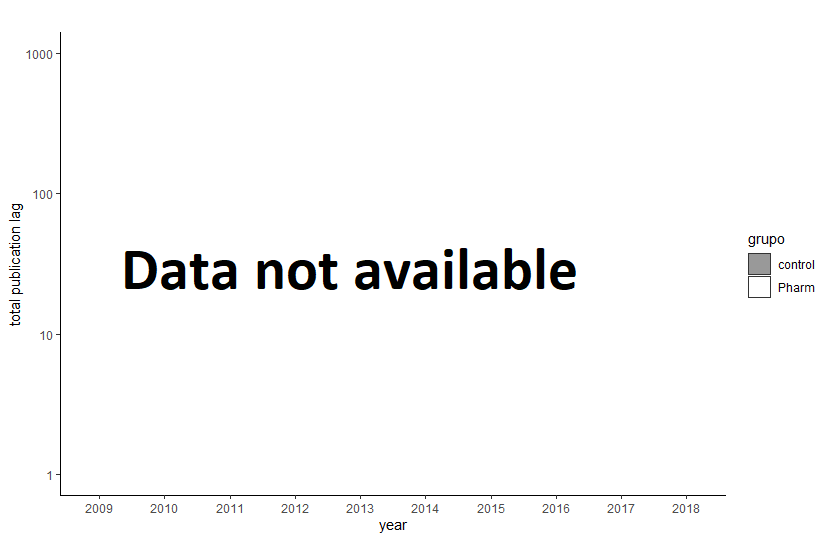 | 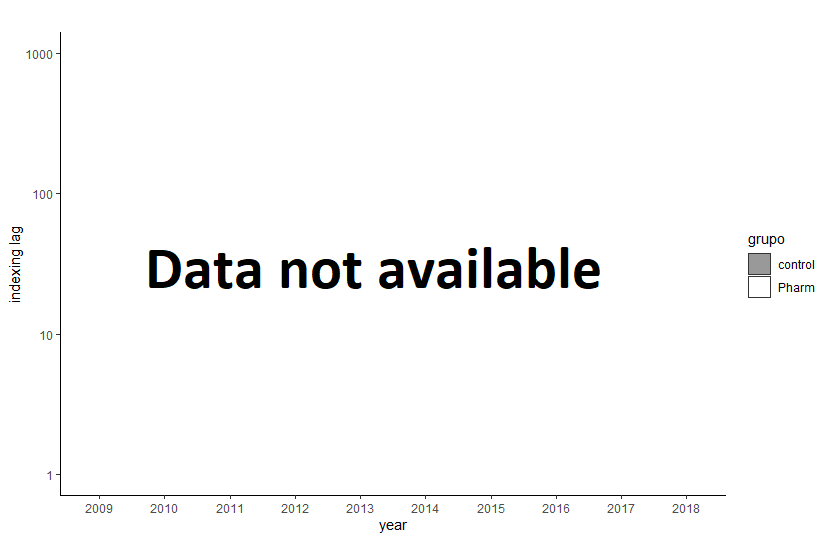 |

| **J Res Pharm Pract** | **2009** | **2010** | **2011** | **2012** | **2013** | **2014** | **2015** | **2016** | **2017** | **2018** | **TOTAL** |
| --- | --- | --- | --- | --- | --- | --- | --- | --- | --- | --- | --- |
| Articles in PubMed | 0 | 0 | 0 | 17 | 32 | 31 | 38 | 54 | 45 | 35 | 252 |
| Submission date; n(%) | - | - | - | 0 | 0 | 0 | 0 | 0 | 0 | 0 | 0 |
| Acceptance date; n(%) | - | - | - | 0 | 0 | 0 | 0 | 0 | 0 | 0 | 0 |
| Online publication date; n(%) | - | - | - | 0 | 0 | 0 | 0 | 0 | 0 | 0 | 0 |
|  |  |  |  |  |  |  |  |  |  |  |  |
| Acceptance lag | - | - | - | NR | NR | NR | NR | NR | NR | NR | NR |
| Lead lag | - | - | - | NR | NR | NR | NR | NR | NR | NR | NR |
| Total lag | - | - | - | NR | NR | NR | NR | NR | NR | NR | NR |
| Indexing lag | - | - | - | NR | NR | NR | NR | NR | NR | NR | NR |

| 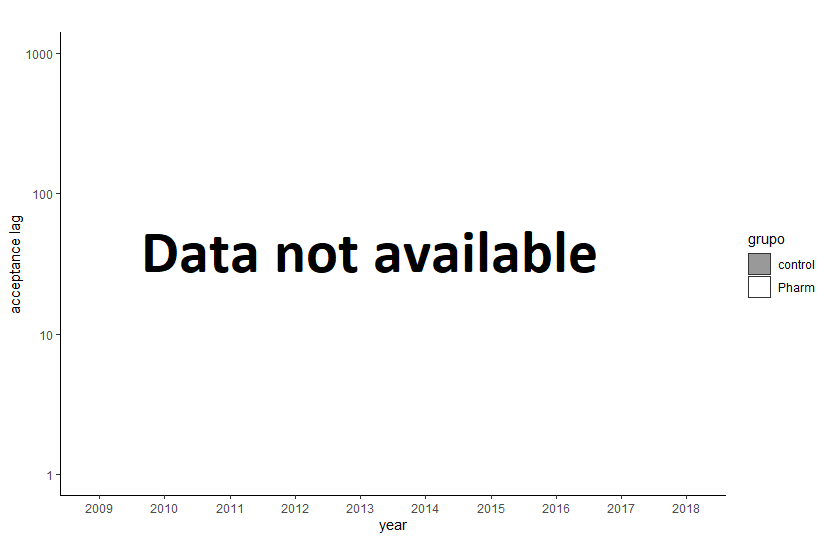 | 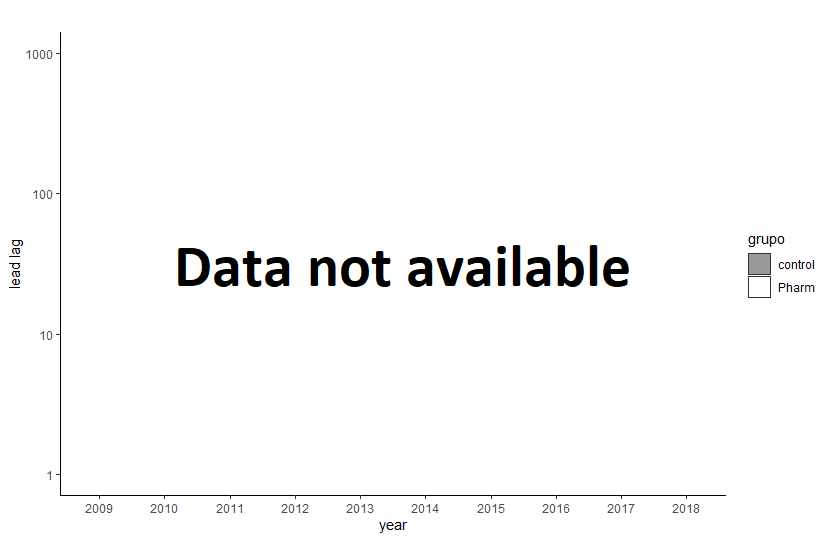 |
| --- | --- |
| 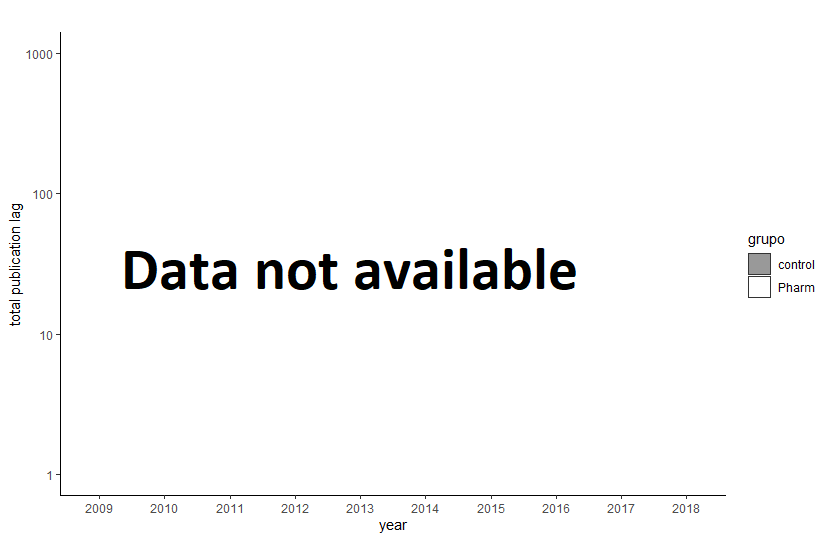 | 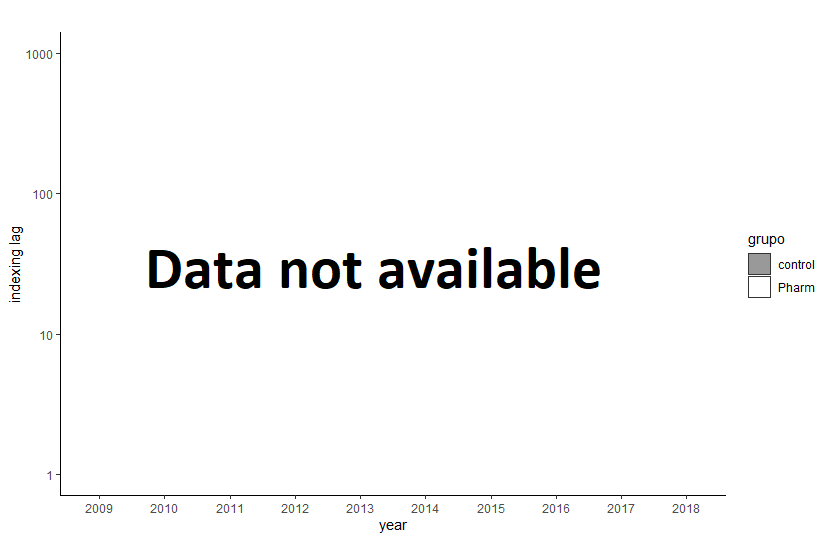 |

| **J Young Pharm** | **2009** | **2010** | **2011** | **2012** | **2013** | **2014** | **2015** | **2016** | **2017** | **2018** | **TOTAL** |
| --- | --- | --- | --- | --- | --- | --- | --- | --- | --- | --- | --- |
| Articles in PubMed | 0 | 84 | 56 | 48 | 44 | 0 | 0 | 0 | 0 | 0 | 232 |
| Submission date; n(%) | - | 0 | 0 | 0 | 42 (95.5) | - | - | - | - | - | 42 (18.1) |
| Acceptance date; n(%) | - | 0 | 0 | 0 | 39 (88.6) | - | - | - | - | - | 39 (16.8) |
| Online publication date; n(%) | - | 0 | 0 | 0 | 42 (95.5) | - | - | - | - | - | 42 (18.1) |
|  |  |  |  |  |  |  |  |  |  |  |  |
| Acceptance lag | - | NR | NR | NR | 60 (45-98) | - | - | - | - | - | 60 (45-98) |
| Lead lag | - | NR | NR | NR | 30 (25-47) | - | - | - | - | - | 30 (25-47) |
| Total lag | - | NR | NR | NR | 94 (82-141) | - | - | - | - | - | 94 (82-141) |
| Indexing lag | - | NR | NR | NR | 96 (66-167) | - | - | - | - | - | 96 (66-167) |

| 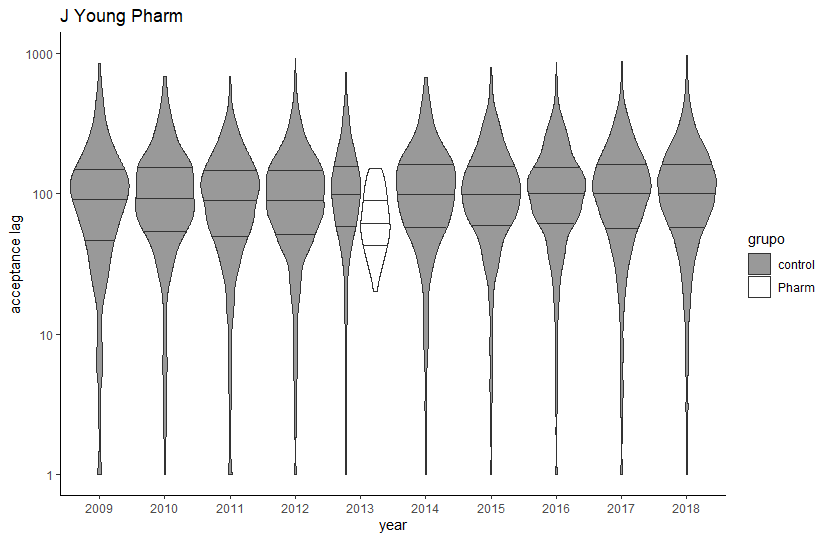 | 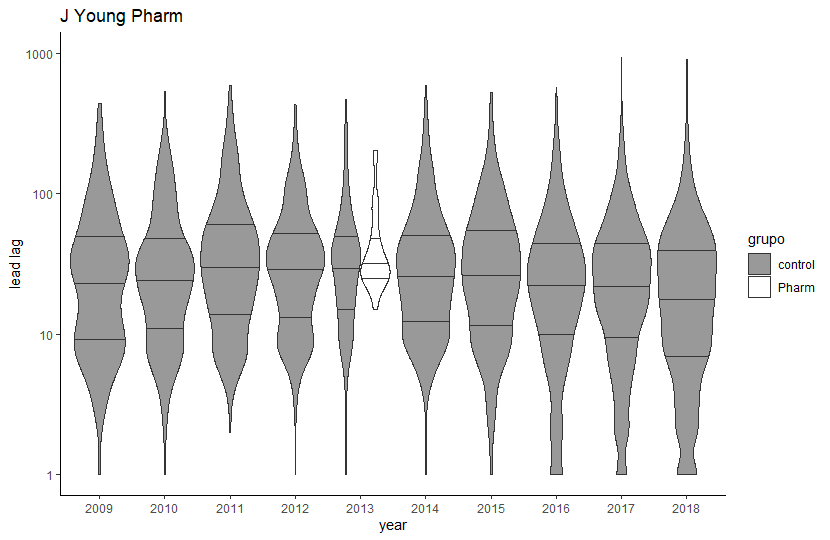 |
| --- | --- |
| 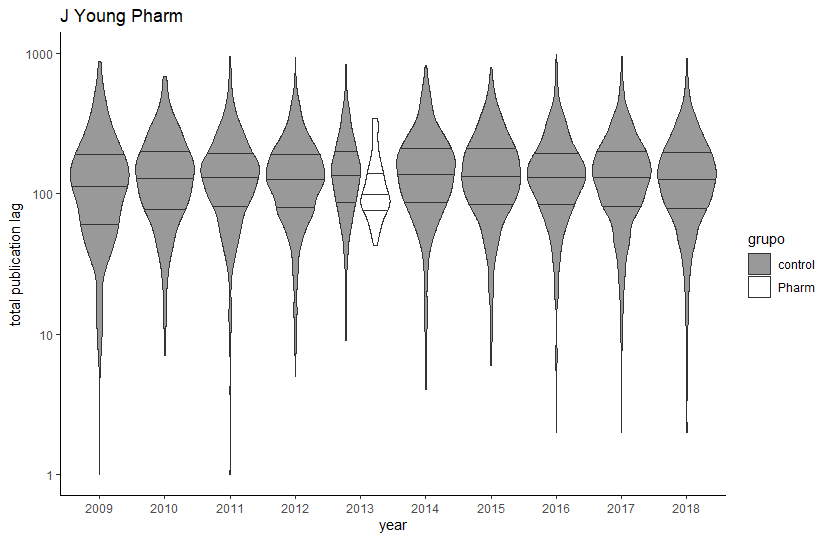 | 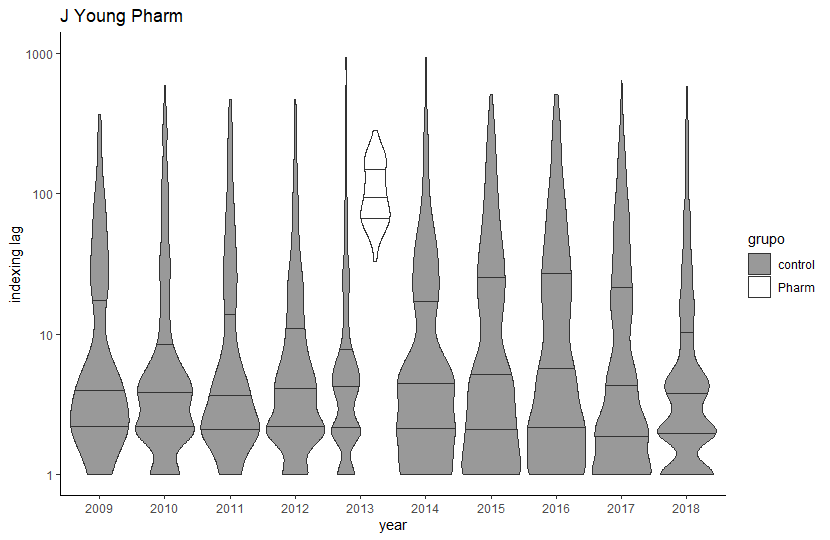 |

| **P T** | **2009** | **2010** | **2011** | **2012** | **2013** | **2014** | **2015** | **2016** | **2017** | **2018** | **TOTAL** |
| --- | --- | --- | --- | --- | --- | --- | --- | --- | --- | --- | --- |
| Articles in PubMed | 90 | 82 | 113 | 107 | 105 | 106 | 119 | 139 | 139 | 132 | 1132 |
| Submission date; n(%) | 0 | 0 | 0 | 0 | 0 | 0 | 0 | 0 | 0 | 0 | 0 |
| Acceptance date; n(%) | 21 (23.3 | 16 (19.5) | 15 (13.3) | 16 (15.0) | 7 (6.7) | 0 | 0 | 0 | 0 | 0 | 75 (6.6) |
| Online publication date; n(%) | 0 | 0 | 0 | 0 | 0 | 0 | 0 | 0 | 0 | 0 | 0 |
|  |  |  |  |  |  |  |  |  |  |  |  |
| Acceptance lag | NR | NR | NR | NR | NR | NR | NR | NR | NR | NR | NR |
| Lead lag | NR | NR | NR | NR | NR | NR | NR | NR | NR | NR | NR |
| Total lag | NR | NR | NR | NR | NR | NR | NR | NR | NR | NR | NR |
| Indexing lag | NR | NR | NR | NR | NR | NR | NR | NR | NR | NR | NR |

| 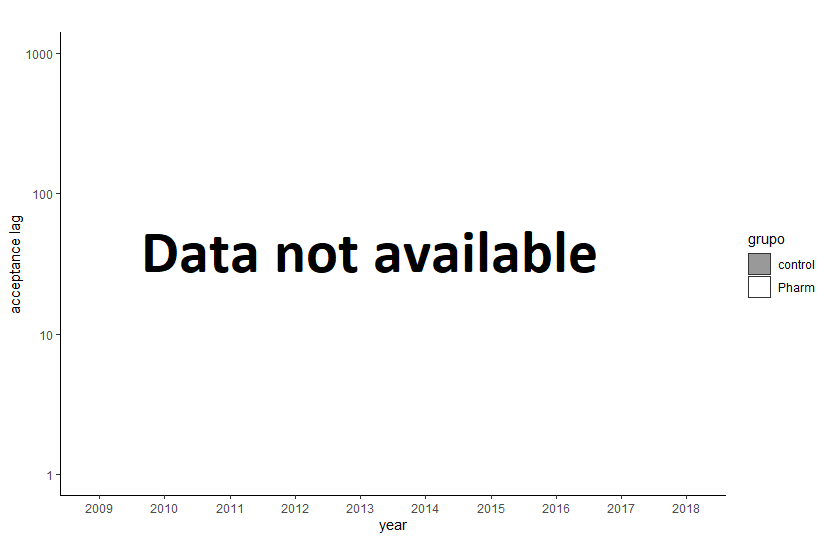 | 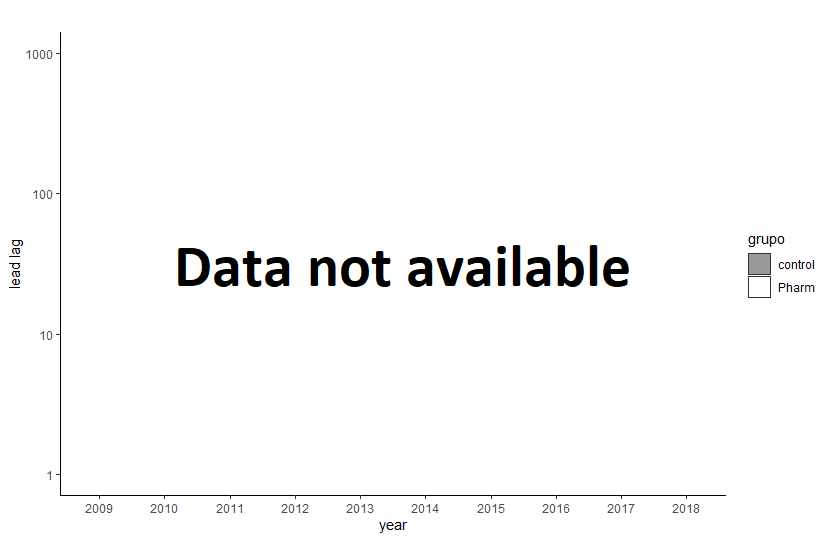 |
| --- | --- |
| 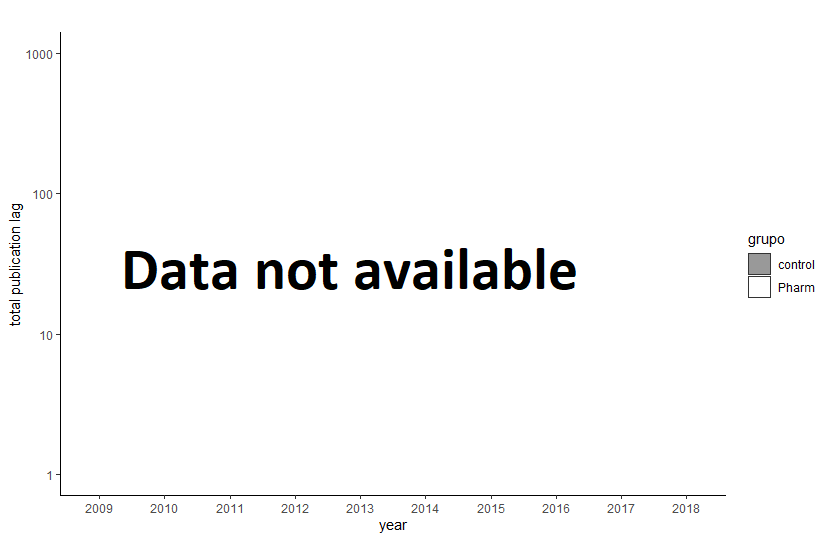 | 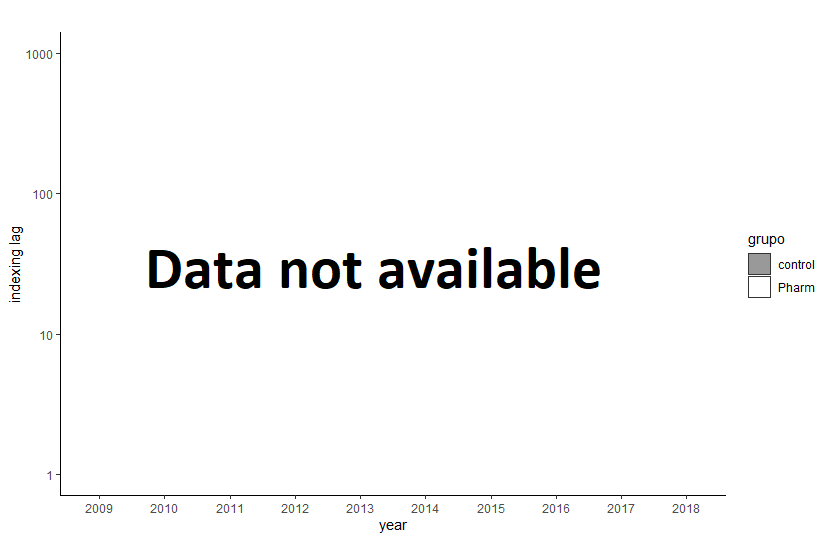 |

| **Pharm Hist** | **2009** | **2010** | **2011** | **2012** | **2013** | **2014** | **2015** | **2016** | **2017** | **2018** | **TOTAL** |
| --- | --- | --- | --- | --- | --- | --- | --- | --- | --- | --- | --- |
| Articles in PubMed | 11 | 11 | 10 | 7 | 13 | 7 | 2 | 7 | 0 | 0 | 68 |
| Submission date; n(%) | 0 | 0 | 0 | 0 | 0 | 0 | 0 | 0 | - | - | 0 |
| Acceptance date; n(%) | 0 | 0 | 0 | 0 | 0 | 0 | 0 | 0 | - | - | 0 |
| Online publication date; n(%) | 0 | 0 | 0 | 0 | 0 | 0 | 0 | 0 | - | - | 0 |
|  |  |  |  |  |  |  |  |  |  |  |  |
| Acceptance lag | NR | NR | NR | NR | NR | NR | NR | NR | - | - | NR |
| Lead lag | NR | NR | NR | NR | NR | NR | NR | NR | - | - | NR |
| Total lag | NR | NR | NR | NR | NR | NR | NR | NR | - | - | NR |
| Indexing lag | NR | NR | NR | NR | NR | NR | NR | NR | - | - | NR |

| 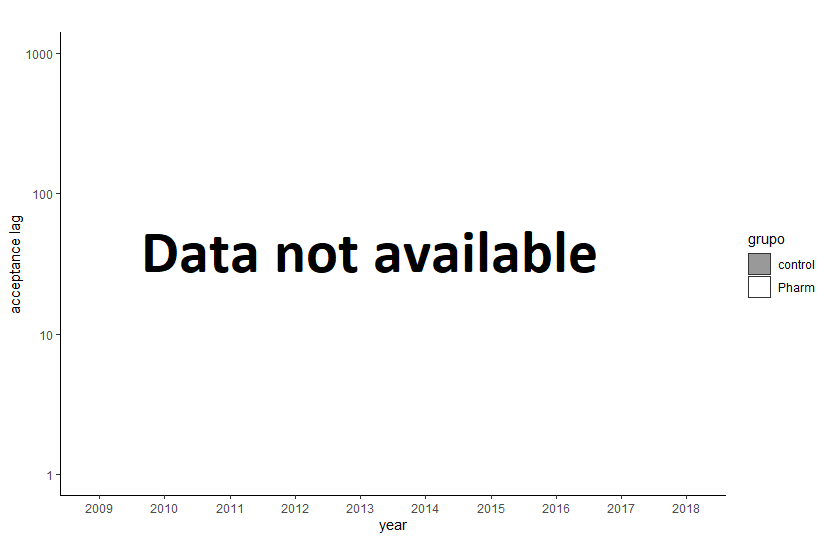 | 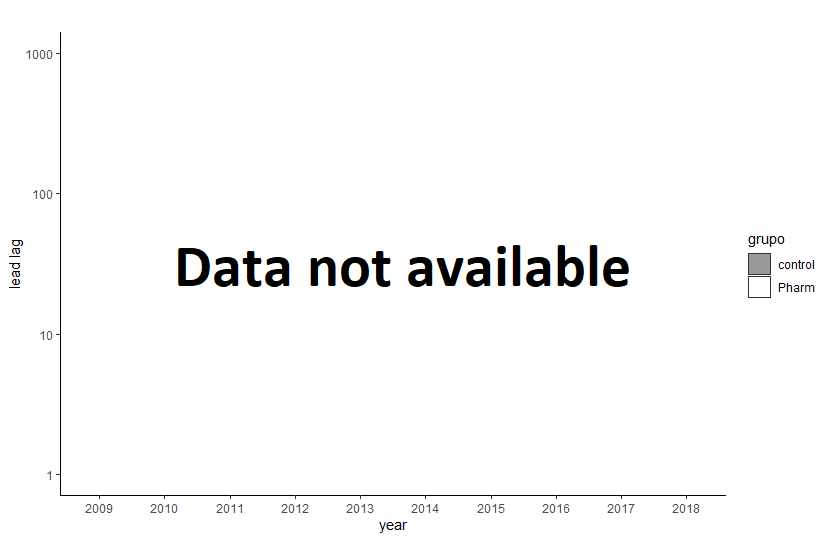 |
| --- | --- |
| 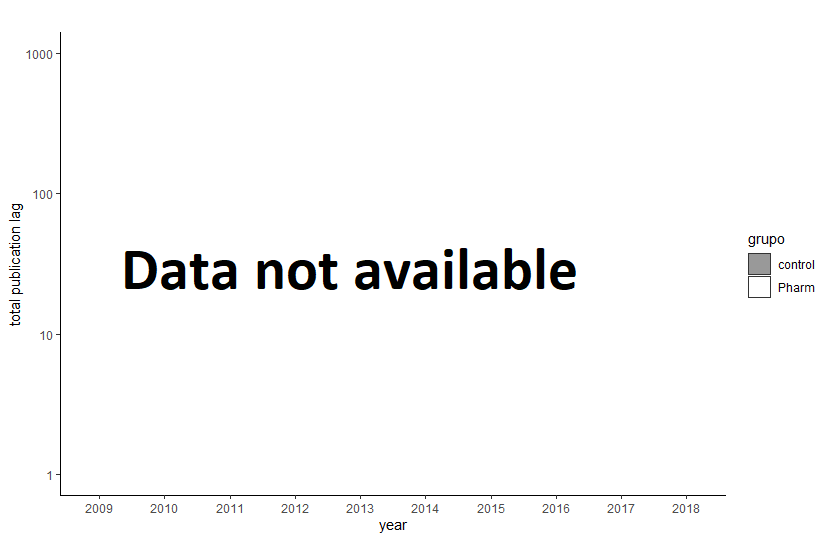 | 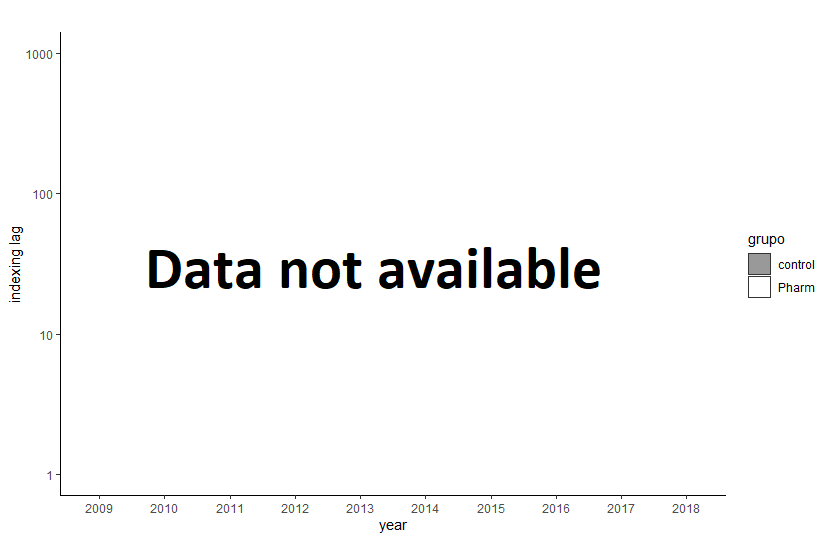 |

| **Pharm Hist (Lond)** | **2009** | **2010** | **2011** | **2012** | **2013** | **2014** | **2015** | **2016** | **2017** | **2018** | **TOTAL** |
| --- | --- | --- | --- | --- | --- | --- | --- | --- | --- | --- | --- |
| Articles in PubMed | 8 | 14 | 9 | 17 | 17 | 20 | 16 | 16 | 0 | 0 | 117 |
| Submission date; n(%) | 0 | 0 | 0 | 0 | 0 | 0 | 0 | 0 | - | - | 0 |
| Acceptance date; n(%) | 0 | 0 | 0 | 0 | 0 | 0 | 0 | 0 | - | - | 0 |
| Online publication date; n(%) | 0 | 0 | 0 | 0 | 0 | 0 | 0 | 0 | - | - | 0 |
|  |  |  |  |  |  |  |  |  |  |  |  |
| Acceptance lag | NR | NR | NR | NR | NR | NR | NR | NR | - | - | NR |
| Lead lag | NR | NR | NR | NR | NR | NR | NR | NR | - | - | NR |
| Total lag | NR | NR | NR | NR | NR | NR | NR | NR | - | - | NR |
| Indexing lag | NR | NR | NR | NR | NR | NR | NR | NR | - | - | NR |

| 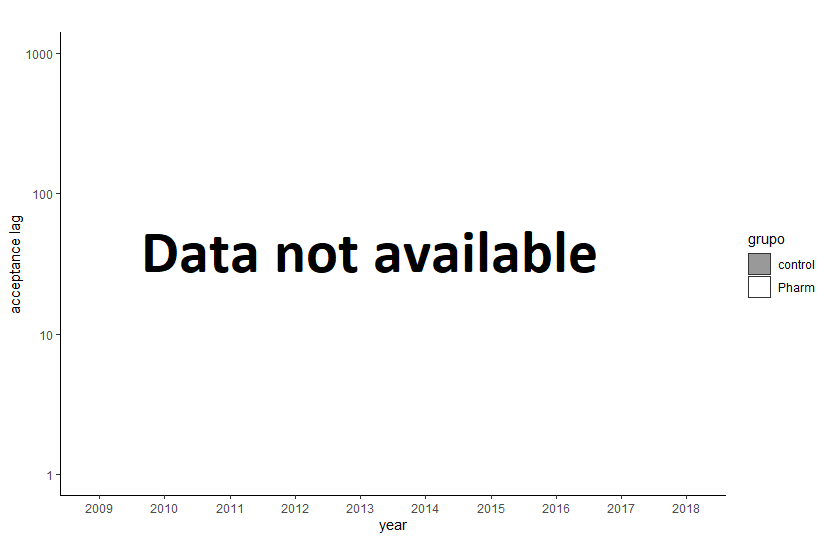 | 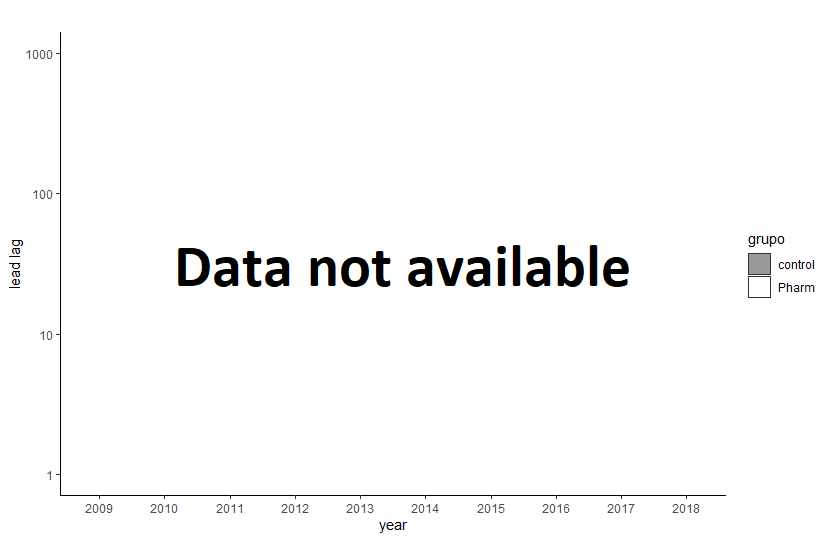 |
| --- | --- |
| 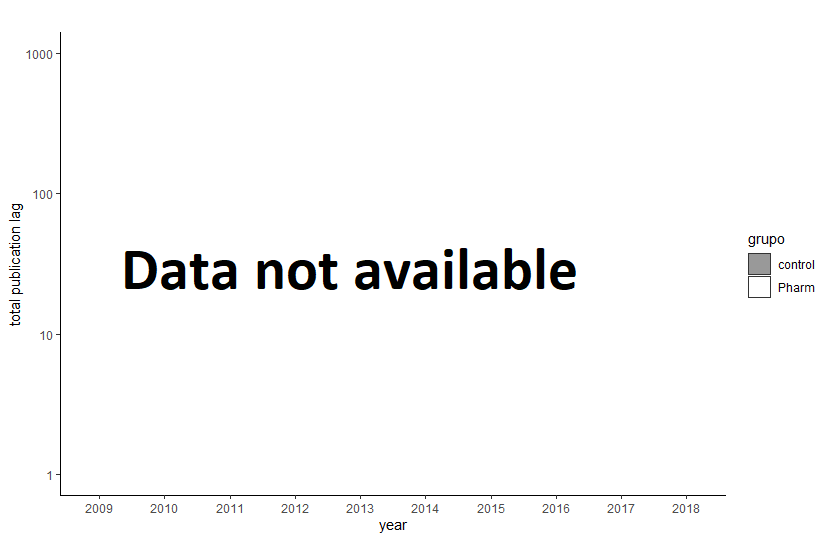 | 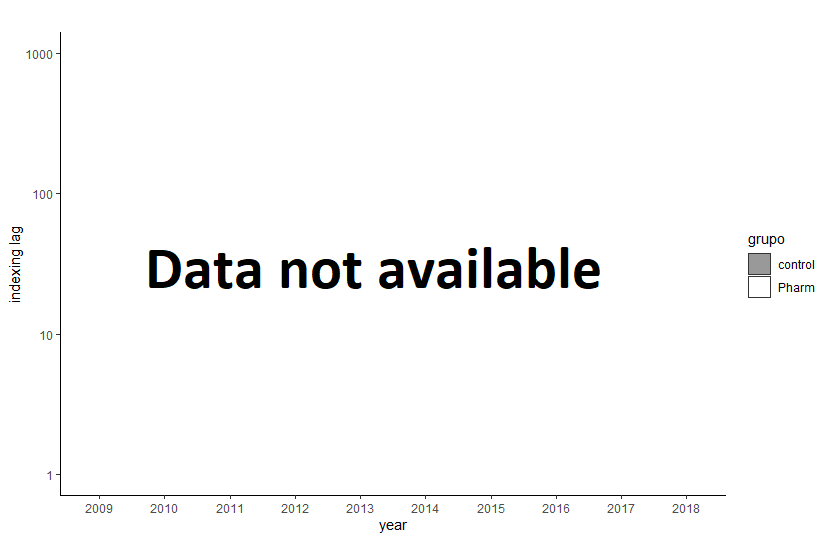 |

| **Pharm Pat Anal** | **2009** | **2010** | **2011** | **2012** | **2013** | **2014** | **2015** | **2016** | **2017** | **2018** | **TOTAL** |
| --- | --- | --- | --- | --- | --- | --- | --- | --- | --- | --- | --- |
| Articles in PubMed | 0 | 0 | 0 | 66 | 72 | 58 | 37 | 47 | 38 | 31 | 349 |
| Submission date; n(%) | - | - | - | 0 | 0 | 0 | 0 | 0 | 0 | 0 | 0 |
| Acceptance date; n(%) | - | - | - | 0 | 0 | 0 | 0 | 0 | 0 | 0 | 0 |
| Online publication date; n(%) | - | - | - | 0 | 0 | 0 | 5 (13.5) | 44 (93.6) | 38 (100) | 31 (100) | 118 (33.8) |
|  |  |  |  |  |  |  |  |  |  |  |  |
| Acceptance lag | - | - | - | NR | NR | NR | NR | NR | NR | NR | NR |
| Lead lag | - | - | - | NR | NR | NR | NR | NR | NR | NR | NR |
| Total lag | - | - | - | NR | NR | NR | NR | NR | NR | NR | NR |
| Indexing lag | - | - | - | NR | NR | NR | 1 (1-1) | 1 (1-1) | 1 (1-1) | 1 (1-1) | 1 (1-1) |

|  |  |
| --- | --- |
|  |  |

| **Pharm Pract (Granada)** | **2009** | **2010** | **2011** | **2012** | **2013** | **2014** | **2015** | **2016** | **2017** | **2018** | **TOTAL** |
| --- | --- | --- | --- | --- | --- | --- | --- | --- | --- | --- | --- |
| Articles in PubMed | 35 | 35 | 36 | 33 | 34 | 37 | 37 | 43 | 51 | 50 | 391 |
| Submission date; n(%) | 35 (100) | 35 (100) | 36 (100) | 32 (97.0) | 33 (97.1) | 36 (97.3) | 35 (94.6) | 41 (95.3) | 50 (98.0) | 50 (100) | 383 (98.0) |
| Acceptance date; n(%) | 35 (100) | 35 (100) | 36 (100) | 32 (97.0) | 34 (100) | 36 (97.3) | 35 (94.6) | 39 (90.7) | 49 (96.1) | 49 (98.0) | 380 (97.2) |
| Online publication date; n(%) | 9 (25.7) | 9 (25.7) | 36 (100) | 32 (97.0) | 34 (100) | 17 (45.9) | 28 (75.7) | 43 (100) | 49 (96.1) | 47 (94.0) | 304 (77.7) |
|  |  |  |  |  |  |  |  |  |  |  |  |
| Acceptance lag | 112 (79-145) | 114 (80-148) | 154 (105-206) | 189 (144-234) | 126 (83-223) | 143 (115-171) | 134 (110-145) | 137 (94-190) | 122 (92-147) | 148 (121-167) | 134 (103-171) |
| Lead lag | 64 (36-75) | 57 (57-74) | 39 (16-71) | 54 (25-73) | 47 (19-60) | 29 (18-51) | 21 (13-63) | 23 (14-45) | 26 (11-61) | 28 (16-42) | 33 (16-57) |
| Total lag | 153 (140-201) | 185 (151-202) | 203 (146-235) | 227 (193-275) | 178 (130-274) | 189 (150-212) | 157 (135-195) | 158 (126-217) | 152 (126-188) | 180 (160-195) | 179 (139-218) |
| Indexing lag | NR | NR | NR | 298 (298-298) | 117 (43-211) | 19 (19-19) | 30 (17-34) | 22 (20-43) | 24 (12-62) | 32 (21-56) | 30 (19-55) |

|  |  |
| --- | --- |
|  |  |

| **Pharmacy (Basel)** | **2009** | **2010** | **2011** | **2012** | **2013** | **2014** | **2015** | **2016** | **2017** | **2018** | **TOTAL** |
| --- | --- | --- | --- | --- | --- | --- | --- | --- | --- | --- | --- |
| Articles in PubMed | 0 | 0 | 0 | 0 | 0 | 0 | 30 | 36 | 67 | 136 | 269 |
| Submission date; n(%) | - | - | - | - | - | - | 29 (66.7) | 36 (100) | 67 (100) | 136 (100) | 268 (99.6) |
| Acceptance date; n(%) | - | - | - | - | - | - | 29 (66.7) | 36 (100) | 67 (100) | 136 (100) | 268 (99.6) |
| Online publication date; n(%) | - | - | - | - | - | - | 30 (100) | 36 (100) | 67 (100) | 136 (100) | 268 (100) |
|  |  |  |  |  |  |  |  |  |  |  |  |
| Acceptance lag | - | - | - | - | - | - | 75 (49-118) | 72 (49-118) | 52 (35-80) | 40 (25-55) | 48 (32-71) |
| Lead lag | - | - | - | - | - | - | 8 (6-15) | 6 (3-9) | 5 (3-7) | 4 (2-6) | 5 (3-7) |
| Total lag | - | - | - | - | - | - | 84 (56-135) | 52 (78-125) | 57 (40-85) | 45 (29-59) | 54 (36-78) |
| Indexing lag | - | - | - | - | - | - | NR | NR | 96 (14-186) | 3 (2-4) | 3 (1-28) |

|  |  |
| --- | --- |
|  |  |

| **Regul Toxicol Pharmacol** | **2009** | **2010** | **2011** | **2012** | **2013** | **2014** | **2015** | **2016** | **2017** | **2018** | **TOTAL** |
| --- | --- | --- | --- | --- | --- | --- | --- | --- | --- | --- | --- |
| Articles in PubMed | 123 | 150 | 145 | 174 | 140 | 220 | 260 | 281 | 249 | 274 | 2016 |
| Submission date; n(%) | 118 (95.9) | 149 (99.3) | 144 (99.3) | 174 (100) | 136 (97.1) | 217 (98.6) | 258 (99.2) | 277 (98.6) | 243 (97.6) | 268 (97.8) | 1984 (98.4) |
| Acceptance date; n(%) | 118 (95.9) | 149 (99.3) | 144 (99.3) | 174 (100) | 136 (97.1) | 217 (98.6) | 259 (99.6) | 277 (98.6) | 243 (97.6) | 268 (97.8) | 1985 (98.5) |
| Online publication date; n(%) | 119 (96.7) | 149 (99.3) | 140 (96.6) | 174 (100) | 139 (99.3) | 219 (99.5) | 260 (100) | 280 (99.6) | 248 (99.6) | 273 (99.6) | 2001 (99.3) |
|  |  |  |  |  |  |  |  |  |  |  |  |
| Acceptance lag | 91 (65-141) | 95 (62-155) | 90 (62-132) | 103 (62-136) | 122 (65-165) | 79 (41-133) | 92 (50-141) | 78 (25-120) | 123 (84-185) | 111 (71-159) | 98 (59-147) |
| Lead lag | 8 (5-12) | 6 (5-10) | 8 (7-12) | 9 (7-12) | 10 (8-14) | 8 (7-10) | 7 (5-10) | 3 (2-5) | 2 (2-4) | 3 (1-4) | 6 (3-10) |
| Total lag | 107 (72-150) | 106 (73-167) | 100 (74-141) | 115 (74-152) | 134 (77-176) | 86 (49-142) | 101 (56-150) | 82 (34-126) | 127 (88-189) | 115 (74-163) | 106 (67-155) |
| Indexing lag | 5 (4-14) | 4 (4-5) | 6 (4-11) | 10 (6-12) | 5 (5-6) | 5 (4-6) | 5 (4-5) | 5 (4-11) | 5 (4-5) | 4 (4-5) | 5 (4-6) |

|  |  |
| --- | --- |
|  |  |

| **Res Social Adm Pharm** | **2009** | **2010** | **2011** | **2012** | **2013** | **2014** | **2015** | **2016** | **2017** | **2018** | **TOTAL** |
| --- | --- | --- | --- | --- | --- | --- | --- | --- | --- | --- | --- |
| Articles in PubMed | 40 | 37 | 42 | 58 | 94 | 85 | 87 | 106 | 144 | 283 | 976 |
| Submission date; n(%) | 40 (100) | 36 (97.3) | 42 (100) | 58 (100) | 94 (100) | 85 (100) | 87 (100) | 106 (100) | 144 (100) | 282 (99.6) | 974 (99.8) |
| Acceptance date; n(%) | 40 (100) | 36 (97.3) | 42 (100) | 58 (100) | 94 (100) | 85 (100) | 87 (100) | 106 (100) | 144 (100) | 281 (99.3) | 973 (99.7) |
| Online publication date; n(%) | 31 (77.5) | 26 (70.3) | 40 (95.2) | 51 (87.9) | 85 (90.4) | 84 (98.8) | 87 (100) | 106 (100) | 136 (94.4) | 283 (100) | 929 (95.2) |
|  |  |  |  |  |  |  |  |  |  |  |  |
| Acceptance lag | 103 (42-167) | 103 (53-147) | 97 (76-136) | 94 (58-143) | 105 (64-184) | 93 (60-151) | 83 (4-117) | 1 (0-69) | 7 (0-112) | 104 (4-149) | 85 (1-135) |
| Lead lag | 168 (123-232) | 80 (59-127) | 69 (58-89) | 74 (57-111) | 55 (37-75) | 33 (13-42) | 10 (7-18) | 10 (8-14) | 10 (3-17) | 3 (1-5) | 12 (4-43) |
| Total lag | 284 (222-408) | 205 (154-250) | 176 (140-234) | 189 (152-243) | 168 (115-256) | 133 (87-187) | 102 (49-137) | 16 (9-79) | 36 (11-124) | 108 (21-162) | 119 (29-179) |
| Indexing lag | 220 (136-227) | 257 (215-311) | 184 (57-261) | 3 (2-6) | 4 (3-5) | 6 (5-31) | 33 (28-48) | 30 (23-39) | 24 (13-32) | 11 (8-16) | 17 (7-33) |

|  |  |
| --- | --- |
|  |  |

| **Saudi Pharm J** | **2009** | **2010** | **2011** | **2012** | **2013** | **2014** | **2015** | **2016** | **2017** | **2018** | **TOTAL** |
| --- | --- | --- | --- | --- | --- | --- | --- | --- | --- | --- | --- |
| Articles in PubMed | 16 | 31 | 35 | 48 | 53 | 81 | 97 | 91 | 178 | 162 | 792 |
| Submission date; n(%) | 15 (93.8) | 30 (96.8) | 35 (100) | 48 (100) | 43 (81.1) | 77 (95.1) | 95 (97.9) | 67 (73.6) | 140 (78.7) | 161 (99.4) | 711 (89.8) |
| Acceptance date; n(%) | 15 (93.8) | 30 (96.8) | 35 (100) | 48 (100) | 43 (81.1) | 76 (93.8) | 95 (97.9) | 67 (73.6) | 140 (78.7) | 161 (99.4) | 710 (89.6) |
| Online publication date; n(%) | 15 (93.8) | 30 (96.8) | 35 (100) | 48 (100) | 39 (73.6) | 76 (93.8) | 97 (100) | 91 (100) | 178 (100) | 162 (100) | 771 (97.3) |
|  |  |  |  |  |  |  |  |  |  |  |  |
| Acceptance lag | 108 (75-150) | 95 (63-133) | 104 (63-146) | 48 (24-68) | 48 (37-79) | 46 (41-73) | 59 (41-73) | 28 (19-46) | 107 (63-168) | 76 (55-127) | 65 (40-104) |
| Lead lag | 138 (120-143) | 31 (23-45) | 11 (9-27) | 8 (7-11) | 12 (8-21) | 11 (10-16) | 9 (8-11) | 9 (7-14) | 6 (3-10) | 2 (1-3) | 8 (4-13) |
| Total lag | 254 (203-282) | 128 (99-167) | 117 (85-160) | 60 (37-77) | 64 (50-93) | 60 (46-74) | 70 (49-84) | 39 (27-66) | 113 (66-179) | 79 (57-131) | 76 (51-119) |
| Indexing lag | NR | 46 (31-62) | 73 (62-112) | 249 (187-506) | 452 (334-585) | 358 (315-403) | 311 (280-478) | 430 (47-578) | 246 (167-285) | 148 (114-184) | 253 (126-372) |

|  |  |
| --- | --- |
|  |  |

| **Yakugaku Zasshi** | **2009** | **2010** | **2011** | **2012** | **2013** | **2014** | **2015** | **2016** | **2017** | **2018** | **TOTAL** |
| --- | --- | --- | --- | --- | --- | --- | --- | --- | --- | --- | --- |
| Articles in PubMed | 190 | 233 | 240 | 199 | 175 | 181 | 191 | 223 | 203 | 215 | 2050 |
| Submission date; n(%) | 0 | 0 | 0 | 0 | 0 | 0 | 0 | 0 | 0 | 0 | 0 |
| Acceptance date; n(%) | 0 | 0 | 0 | 0 | 0 | 0 | 0 | 0 | 0 | 0 | 0 |
| Online publication date; n(%) | 0 | 0 | 0 | 2 (1.0) | 12 (6.9) | 6 (3.3) | 4 (2.1) | 7 (3.1) | 11 (5.4) | 10 (4.7) | 52 (2.5) |
|  |  |  |  |  |  |  |  |  |  |  |  |
| Acceptance lag | NR | NR | NR | NR | NR | NR | NR | NR | NR | NR | NR |
| Lead lag | NR | NR | NR | NR | NR | NR | NR | NR | NR | NR | NR |
| Total lag | NR | NR | NR | NR | NR | NR | NR | NR | NR | NR | NR |
| Indexing lag | NR | NR | NR | 1 (1-1) | 3 (1-4) | 3 (2-5) | 4 (2-40) | 1 (1-5) | 2 (1-4) | 2 (1-3) | 2 (1-4) |

|  |  |
| --- | --- |
|  |  |

| **Yakushigaku Zasshi** | **2009** | **2010** | **2011** | **2012** | **2013** | **2014** | **2015** | **2016** | **2017** | **2018** | **TOTAL** |
| --- | --- | --- | --- | --- | --- | --- | --- | --- | --- | --- | --- |
| Articles in PubMed | 9 | 14 | 13 | 0 | 0 | 14 | 19 | 9 | 0 | 0 | 78 |
| Submission date; n(%) | 0 | 0 | 0 | - | - | 0 | 0 | 0 | - | - | 0 |
| Acceptance date; n(%) | 0 | 0 | 0 | - | - | 0 | 0 | 0 | - | - | 0 |
| Online publication date; n(%) | 0 | 0 | 0 | - | - | 0 | 0 | 0 | - | - | 0 |
|  |  |  |  |  |  |  |  |  |  |  |  |
| Acceptance lag | NR | NR | NR | - | - | NR | NR | NR | - | - | NR |
| Lead lag | NR | NR | NR | - | - | NR | NR | NR | - | - | NR |
| Total lag | NR | NR | NR | - | - | NR | NR | NR | - | - | NR |
| Indexing lag | NR | NR | NR | - | - | NR | NR | NR | - | - | NR |

|  |  |
| --- | --- |
|  |  |
