# Supplementary material for: Publication speed in pharmacy practice journals: A comparative analysis
Source: PLoS One. 2021 Jun 29;16(6):e0253713. doi: 10.1371/journal.pone.0253713 (PMC8241115; doi:10.1371/journal.pone.0253713)
Supplement: Attachment — Submitted filename: Mendes et al review_PLOS ONE.docx [file pone.0253713.s006.docx]

The article “Publication speed in pharmacy practice journals: a comparative analysis” by Mendes et al evaluates the duration of the publication process in Pharmacy practice journals compared to other scientific disciplines.

The article provides valuable and significant information with a large sample size of articles and carefully analyzed data. I addition, the article describes some of the pain points of the publication process that add to delays, with peer review as the major process that may significantly add to delays.

The quantification of the acceptance, lead and indexing lags are useful and interesting contributions. Additionally, the comparison of the present results to previous studies such as the Rodriguez data is interesting and sets up a good framework for the need to carry out this particular investigation.

Table 3 with lag times presents useful data to compare the two groups and supports the above notions.

Also interesting is the open access data presented in this study. The magnitude of the indexing lag 92 days vs 5 days for pharmacy (which seems an incredibly large difference…can an 18.4-fold difference be easily explained?)...compared to 4 days vs 3 days in the control seems to ask for further study/data. It would be important to explain this further in the text and perhaps clarify the reasons.

However, the manuscript also has some significant drawbacks. Large sections of text seem opinion based, especially the discussion. The introduction and discussion need to be significantly curtailed and seem to digress significantly from the hard data presented. I would recommend removing large sections of text that are not addressed in the analyses.

During “emergency times”, COVID, the peer review process, pre-prints are all mentioned…all of which are important and currently topical items when exploring publication speed…however, the reader is left with wanting more granular data or at least some *direct* data that addresses these influencing factors and their effect on publication speed. At best, the current dataset is indirect regarding these discussions. The article should be significantly shortened.

In brief, I would support publication after a very significant reduction in length… perhaps as a short note.
