# Supplementary material for: Publication speed in pharmacy practice journals: A comparative analysis
Source: PLoS One. 2021 Jun 29;16(6):e0253713. doi: 10.1371/journal.pone.0253713 (PMC8241115; doi:10.1371/journal.pone.0253713)
Supplement: Attachment — Submitted filename: Response to the editor.docx [file pone.0253713.s008.docx]

Response to the editor:

Thank you for submitting your manuscript to PLOS ONE. After careful consideration, we feel that it has merit but does not fully meet PLOS ONE’s publication criteria as it currently stands. Therefore, we invite you to submit a revised version of the manuscript that addresses the points raised during the review process.

A premise to be considered for publication is a revision of the statistical analyses (compare comment of reviewer 2 regarding the statistical analyses). To facilitate interpretation it would be better to perform the analysis on the original scale, instead of using Cohen’s d. Furthermore, a multivariate analysis should be performed because it can be expected that the univariate analyses are confounded and consequently it is not possible to assess the impact of individual factors.

RE: Thank you very much for the opportunity to improve our manuscript. Following previous recommendations from reviewer #2 we have now included the multivariate analyses by performing a multivariate linear regression for the three lag times. The results, presented in Table 4, confirm the findings reported with bivariate analysis.

| Table 4. Multivariate analyses for the three lag times | | | | |
| --- | --- | --- | --- | --- |
| Acceptance lag | R-square: 0.006; Durbin-Watson= 1.788 | | | |
|  | Beta | 95%IC | p-value | VIF |
| Study group | -3.910 | -7.077 : -0.742 | 0.016 | 1.072 |
| CiteScore (2018) | 1.000 | -0.267 : 2.268 | 0.122 | 2.991 |
| Opean Access journal? | -13.822 | -17.237 : -10.406 | <0.001 | 1.005 |
| journal Impact Factor | -1.861 | -2.653 : -1.069 | <0.001 | 3.041 |
|  | | | | |
| Lead lag | R-square: 0.052; Durbin-Watson= 1.620 | | | |
|  | Beta | 95%IC | p-value | VIF |
| Study group | -16.966 | -18.570 : -15.363 | <0.001 | 1.088 |
| CiteScore (2018) | -5.779 | -6.436 : -5.121 | <0.001 | 3.447 |
| Opean Access journal? | -0.097 | -1.930 : 1.737 | 0.918 | 1.029 |
| journal Impact Factor | 2.795 | 2.339 : 3.251 | <0.001 | 3.550 |
|  | | | | |
| Indexing lag | R-square: 0.152; Durbin-Watson= 0.550 | | | |
|  | Beta | 95%IC | p-value | VIF |
| Study group | 41.721 | 39.374 : 44.069 | <0.001 | 1.057 |
| CiteScore (2018) | -0.242 | -0.959 : 0.475 | 0.508 | 2.913 |
| Opean Access journal? | 63.494 | 60.803 : 66.184 | <0.001 | 1.018 |
| journal Impact Factor | 0.524 | 0.078 : 0.969 | 0.021 | 2.968 |
| 95%IC: 95% confidence interval; VIF: Variance inflation factor | | | | |

Regarding the use of Cohen’d d in table 2, the comment Reviewer #2 provided in the previous round stated: “Table 3: Similar to my above comment, I think instead of reporting Cohen’s d, a point estimate and confidence interval for a difference would be more interpretable and helpful”. To attend this comment, we included the median (as a point estimate) and the IQR (instead the confidence interval, which we cannot use because we are presenting dispersion measure of a non-normally distributed variable). Additionally, we presented the null hypothesis test that that estimated the significance of the differences between the two groups under analysis. These two elements comply with the requirements of the reviewer #2. In addition to these requested elements, and following the recommendations of the American Statistical Association, we insist in providing the effect size measures of these differences, presented as the Cohen’s d. The latter should be understood as additional information after providing the requested information.
